# Identification of *CBL* and *CIPK* gene families and functional characterization of *CaCIPK1* under *Phytophthora capsici* in pepper (*Capsicum annuum* L.)

**DOI:** 10.1186/s12864-019-6125-z

**Published:** 2019-10-25

**Authors:** Xiao Ma, Wen-Xian Gai, Yi-Ming Qiao, Muhammad Ali, Ai-Min Wei, De-Xu Luo, Quan-Hui Li, Zhen-Hui Gong

**Affiliations:** 1 College of Horticulture, Northwest A&F University, Yangling, Shaanxi 712100 People’s Republic of China; 2 Tianjin Vegetable Research Center, Tianjin, 300192 People’s Republic of China; 3 Xuhuai Region Huaiyin Institute of Agricultural Sciences, Huaian, Jiangsu 223001 People’s Republic of China; 4 Qinghai Academy of Agricultural and Forestry Sciences, Xining, Qinghai 810016 People’s Republic of China; 5 State Key Laboratory of Vegetable Germplasm Innovation, Tianjin, 300384 People’s Republic of China

**Keywords:** Pepper, Genome-wide, *CBL* and *CIPK* family, *CaCIPK1*, Abiotic stress, *Phytophthora capsici*

## Abstract

**Background:**

Calcineurin B-like proteins (CBLs) are major Ca^2+^ sensors that interact with CBL-interacting protein kinases (CIPKs) to regulate growth and development in plants. The CBL-CIPK network is involved in stress response, yet little is understood on how CBL-CIPK function in pepper (*Capsicum annuum* L.), a staple vegetable crop that is threatened by biotic and abiotic stressors.

**Results:**

In the present study, nine *CaCBL* and 26 *CaCIPK* genes were identified in pepper and the genes were named based on their chromosomal order. Phylogenetic and structural analysis revealed that *CaCBL* and *CaCIPK* genes clustered in four and five groups, respectively. Quantitative real-time PCR (qRT-PCR) assays showed that *CaCBL* and *CaCIPK* genes were constitutively expressed in different tissues, and their expression patterns were altered when the plant was exposed to *Phytophthora capsici*, salt and osmotic stress. *CaCIPK1* expression changed in response to stress, including exposure to *P. capsici*, NaCl, mannitol, salicylic acid (SA), methyl jasmonate (MeJA), abscisic acid (ABA), ethylene (ETH), cold and heat stress. Knocking down *CaCIPK1* expression increased the susceptibility of pepper to *P. capsici*, reduced root activity, and altered the expression of defense related genes. Transient overexpression of *CaCIPK1* enhanced H_2_O_2_ accumulation, cell death, and expression of genes involved in defense.

**Conclusions:**

Nine *CaCBL* and 26 *CaCIPK* genes were identified in the pepper genome, and the expression of most *CaCBL* and *CaCIPK* genes were altered when the plant was exposed to stress. In particular, we found that *CaCIPK1* is mediates the pepper plant’s defense against *P. capsici*. These results provide the groundwork for further functional characterization of *CaCBL* and *CaCIPK* genes in pepper.

## Background

Plants have evolved tightly regulated signaling pathways to respond to the complex and varying environments that they are exposed to. Calcium (Ca^2+^) is a ubiquitous second messenger that regulates physiological and developmental processes in plants, and Ca^2+^ levels change when plants are exposed to biotic and abiotic stressors [1–3]. Ca^2+^ signatures, which are the distinct set of Ca^2+^ changes that occur in cells, are triggered by different stimuli, and the release of Ca^2+^ activates specific channels, pumps and transporters that are located at the various cellular membranes [4]. Changes in the intracellular concentration of Ca^2+^ is captured by Ca^2+^ sensors, which in turn regulate signaling pathways involved in plant growth and development, including calmodulins (CAMs), calcium-dependent protein kinases (CDPKs), and calcineurin B-like proteins (CBLs) [3].

CBLs have a domain called the EF-hand, which captures intracellular Ca^2+^. The EF-hand is a helix-loop-helix motif containing 12 residues of +X• + Y• + Z•-Y•-X••-Z, where the letters represent the ligands involved in metal coordination, and the dots represent the intervening residues [5]. CBL-interacting protein kinases (CIPKs) belong to the SnRK3 protein family that contain a Ser/Thr protein kinase domain [6]. CIPKs phosphorylate CBLs at a conserved Ser residue in CBL’s PFPF motif [7]. CIPKs frequently have a conserved N-terminal kinase domain, serine-threonine kinases domain, NAF/FISL motif and C-terminal regulatory domain [8, 9]. In addition to these domains, the C-terminus of CIPKs contain a protein-phosphatase interaction (PPI) domain, which interacts with phosphatase 2C (PP2C) proteins [10]. The interaction between the CBL and CIPK is involved in a Ca^2+^-decoding system called the CBL-CIPK network [11].

Previous studies have shown that the CBL-CIPK network is involved in regulating sodium (Na^+^), potassium (K^+^), magnesium (Mg^2+^) and nitrate (NO^3−^) transport across the plasma membrane (PM) or vacuolar membrane (tonoplast) [1, 12–16]. The CBL-CIPK network also plays an important role in regulating auxin, ABA signaling and stomatal movement [12, 17]. Abiotic stresses, such as ionic stress, osmotic stress and extreme temperatures, activate the salt overly sensitive (SOS) pathway and reactive oxygen species (ROS) signals to induce the expression of transcription factors involved in abiotic stress response [18]. The CBL-CIPK pathway was first identified in *Arabidopsis* through the discovery of the SOS pathway, which is comprised of *AtCBL4* (*SOS3*), *AtCIPK24* (*SOS2*) and the plasma membrane-localized Na^+^/H^+^ antiporter (SOS1) [19, 20]. AtCIPK24 interacts with CBLs to confer salt tolerance, and SOS1 is activated by CBL10-CIPK24 in a different salt-tolerance pathway on the tonoplast [21, 22]. The CBL1/CBL9-CIPK23 complexes are localized to the plasma membrane and regulate K^+^ in roots and stomatal guard cells by modulating the K^+^ channel *Arabidopsis* K^+^ Transporter1 (AKT1) [23, 24], and *Arabidopsis* plants overexpressing *CIPK9* and *CBL10* are sensitive to K^+^. In addition, CIPK9 interacts with CBL3 to regulate K^+^ homeostasis under low-K^+^ conditions [25, 26]. In *Arabidopsis*, the expression of *CIPK3* changes in response to ABA, cold, and high salt stress conditions, and *CIPK3* may be a cross-talk “node” that mediates interaction of ABA and abiotic stressors through ABA-dependent and ABA-independent pathways [27, 28]. Moreover, the CBL9-CIPK3-ABR1 (abscisic acid repressor 1) pathway regulates ABA and seed germination [29].

Recent studies have found that *CIPKs* regulate cell signaling across plants species. For instance, the apple CIPK protein kinase MdSOS2L1 interacts with MdCBL1, MdCBL4 and MdCBL10 to increase the levels of antioxidant metabolites and enhance salt tolerance in apple and tomato [30]. Overexpression of *MdCIPK22* increases ABA sensitivity in an *MdAREB2* (ABA responsive element binding factors) dependent manner, and MdCIPK13 phosphorylates MdSUT2.2 (sucrose transporter) to regulate salt tolerance [31, 32]. Overexpressing *SlSOS2* (*SlCIPK24*) increases tolerance against salinity in tomato [33]. In wheat, *TaCIPK23* is involved in ABA and drought stress responses, as well as the crosstalk between ABA signaling and drought [34].

Pepper (*Capsicum annuum* L.) is an important horticultural crop that belongs to Solanaceae, and is a rich source of vitamins, minerals and nutrients that are of great importance for human health [35, 36]. Nevertheless, the cultivation of pepper is threatened by biotic and abiotic stressors, such as pathogens, drought, salinity, and low temperature. *Phytophthora capsici* (*P. capsici*) is a devastating soil-borne pathogen that is causing significant damage to pepper crops worldwide by causing damping-off, seedling blight, and plant death [37, 38]. Infection of plants by pathogens leads to the activation of pattern-triggered immunity (PTI) and effector-triggered immunity (ETI), and infection by microbes leads to micro-associated molecular patterns (MAMPs) of PTI [39]. *OsCIPK14* and *OsCIPK15* regulate MAMPs defense signaling pathways in rice, and *TaCIPK5* positively regulates resistance against stripe rust fungus in wheat with *TaCBL4* [40]. However, whether *CBL* and *CIPK* genes are involved in mediating stress response in pepper remains unclear, and studying the CBL-CIPK network is important for furthering our understanding of how pepper plants respond to biotic and abiotic stressors. Here, we identified two gene families (*CaCBL* and *CaCIPK*) in the pepper genome. Phylogenetic analyses were performed to investigate the evolutionary relationships of the nine CaCBL and 26 CaCIPK members. A comprehensive analysis of gene structure, protein motif conservation, chromosomal location, gene duplication, stress-related *cis*-elements, and prediction of protein-protein interaction networks were conducted to further understand the structure and relationship of *CaCBL* and *CaCIPK* genes. Furthermore, we examined the expression profiles of *CaCBL* and *CaCIPK* genes in pepper plants of various developmental stages and those exposed to biotic and abiotic stressors. We found that *CaCIPK1* expression was induced by exposure to biotic and abiotic stressors, and we utilized the virus-induced gene silencing (VIGS) system to investigate the function of *CaCIPK1* in pepper plants infected with *P. capsici*.

## Results

### Identification of *CaCBL* and *CaCIPK* genes in pepper

To identify *CBLs* and *CIPKs* in pepper, a HMM (Hidden Markov Model) analysis was performed against the CM334 (*Capsicum annuum* Cultivars in Mexico) and Zunla-1 (*Capsicum annuum* Cultivars in China) genomic databases. All putative genes were surveyed to verify the presence of conserved domains. Nine *CBL* and 26 *CIPK* genes were identified and named by chromosomal order (Additional file 1, Additional file 2). CaCBLs had four EF-hands, and CaCIPKs had a protein kinase catalytic domain (PKC), the 24-amino acid NAF/FISL motif and a PPI motif, similar to other genes in these families.

The length of CaCBL proteins varied from 205 (CaCBL3) to 258 (CaCBL5) amino acids, and the predicted molecular weights ranged from 23.55 to 29.73 kDa. The predicted isoelectric points (*p*I) were between 4.63 and 4.95. The length of CaCIPK proteins ranged from 335 (CaCIPK10) to 506 (CaCIPK26) amino acids, and the predicted molecular weights were between 38.27 and 57.26 kDa. The predicted instability indexes revealed some unstable proteins, inducing three CaCBLs (CaCBL5, − 7, − 9) and four CaCIPKs (CaCIPK5, − 10, − 15, − 26) (instability index =40). In the *CaCBL* family, two genes (*CaCBL1* and *CaCBL3*) contained six introns, six genes (*CaCBL2*, *− 4*, *− 6*, *− 7*, *− 8*, *− 9*) had seven introns, and one gene (*CaCBL5*) had eight introns (Additional file 3A). In the *CaCIPK* family, seven *CaCIPK* genes (26.9%) (*CaCIPK2*, − *4*, − *11*, − *13*, − *20*, − *25*, − *26*) contained more than ten introns, while others had zero or one intron (Additional file 3B).

### Phylogenetic and sequence analysis of *CaCBL* and *CaCIPK* genes

**Fig. 1:**
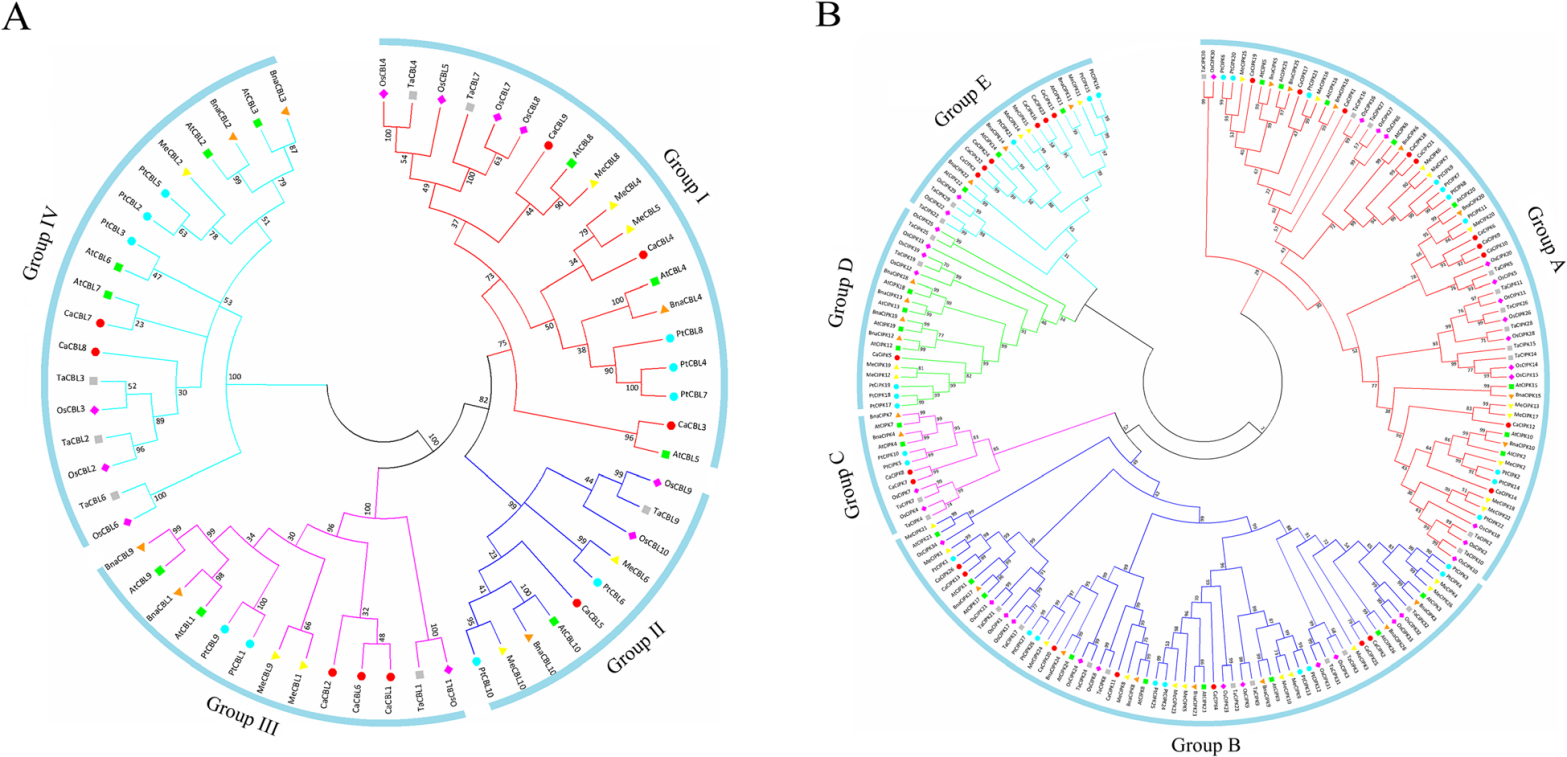
The phylogenetic analysis of *CBL* (**a**) and *CIPK* (**b**) gene families from pepper, *Arabidopsis* cassava, canola, rice, poplar and wheat. Full-length protein sequences were used to construct Neighbor-Joining (NJ) trees by MEGA-X program with pairwise deletion, Poisson correction and bootstrap value 1000. Different species are denoted using different symbols. Subfamilies are distinguished with different colors. CBLs were classified into four groups, and CIPK were classified into five groups

To understand the evolutionary relationship of CBL and CIPK proteins among pepper and other plants, a neighbor-joining (NJ) tree was constructed using amino acid sequences from *Capsicum annuum, Arabidopsis thaliana*, *Manihot esculenta*, *Populus trichocarpa*, *Oryza sativa*, *Triticum aestivum*, and *Brassica napus* (Fig. 1**;** Additional file 4). There were multiple CBL and CIPK sequences from wheat, potentially due to its hexaploidy, therefore only one sequence from each set of homologous gene sequences was used for the analysis. Sixty CBLs were clustered into four different groups (I to VI) with high bootstrap values (Fig. 1a). Three *CBL* genes (*CaCBL3*, *CaCBL4* and *CaCBL9)* were categorized into group I, *CaCBL5* was a part of group II, *CaCBL1, − 2,* and *− 6* belonged to group III, and *CaCBL7* and *CaCBL8* clustered into group IV. *CaCBL7* was orthologous to *AtCBL7*, and *CaCBL8* was orthologous to wheat and rice *CBLs*. There were no sequences available for some *CIPK* genes, such as *TaCIPK6*, *TaCIPK12*, and *TaCIPK13* [41].

A total 188 of *CIPK* genes from seven species were clustered into five subfamilies and assigned names (A to E) (Fig. 1b). Group A contained *CaCIPK1*, *− 6*, *− 9*, *− 10*, *− 12*, *− 14*, *− 17*, *− 18*, *− 19*, and *− 21*, group B included *CaCIPK2*, *− 4*, *− 11*, *− 13*, *− 20*, *− 25*, and *− 26*, group C included *CaCIPK7* and *CaCIPK8*, group D contained only *CaCIPK*5, and group E contained *CaCIPK3*, *− 15*, *− 16*, *− 22*, *− 23*, and *− 24*. *CaCIPK5* and *CaCIPK20* were most similar to poplar and cassava *CIPKs*.

**Fig. 2:**
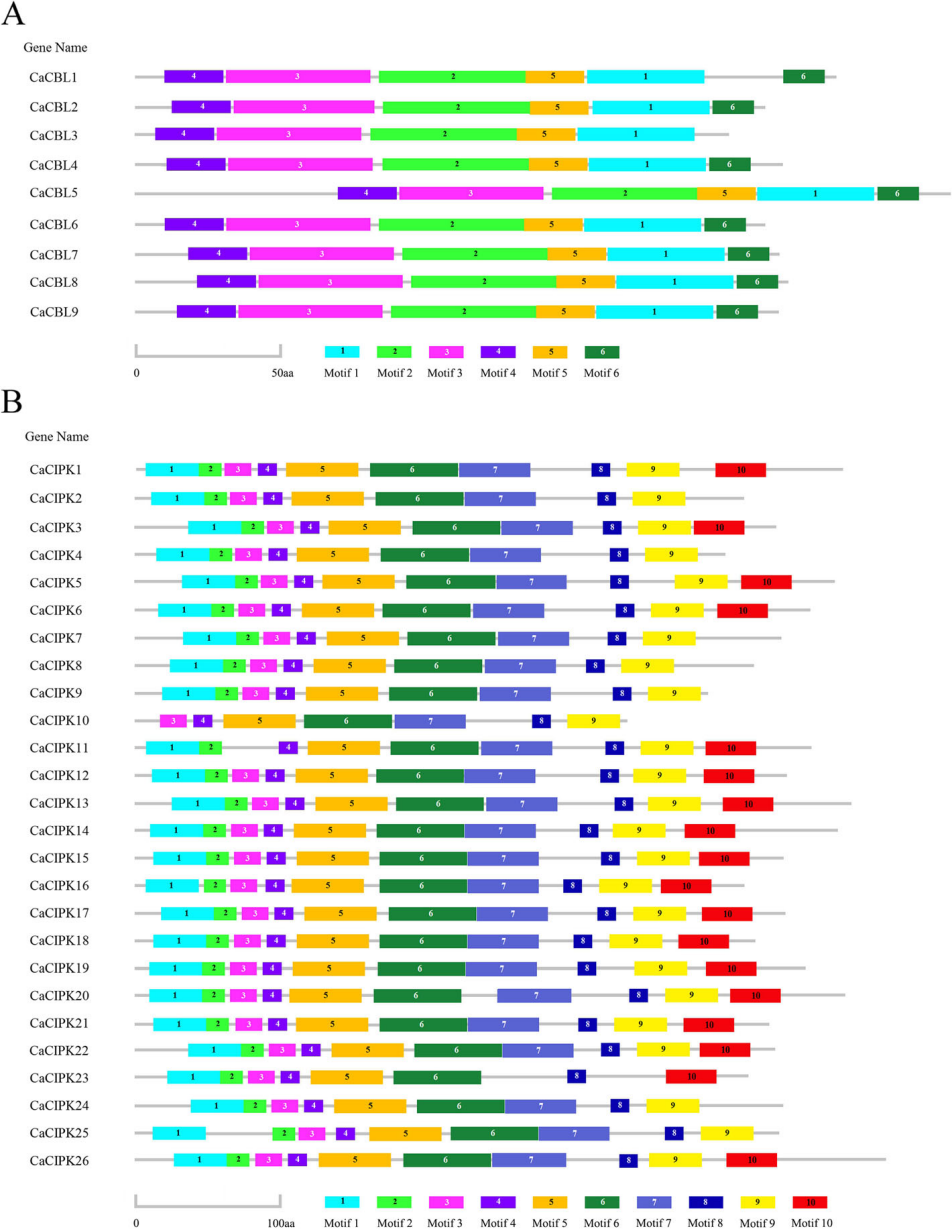
Distribution of conserved motifs in *CaCBL* (**a**) and *CaCIPK* (**b**) families. The MEME program was used to identify the conserved motifs. Different motifs are highlighted with different colors and numbers. For details of motifs refer to Additional files 6 and 7

Multiple sequence alignment (MSA) revealed that CaCBLs have four EF-hand domains and a conserved PFPF motif (Additional file 5A). In addition, five CaCBLs harbored a conserved myristoylation motif (MGXXXS/T) on the N-terminus, including CaCBL1, − 2, − 3, − 4, and − 6. CaCBL5 had an extended N-terminus, which contained a transmembrane helix. CaCBL8 possessed the consensus motif for the tonoplast targeting sequences (TTS, MSQCXDGXKHXCXSXXXCF). MEME analysis showed that CaCBLs have six different conserved motifs, and these were named Motif-1 to Motif-6 (Fig. 2a**;** Additional file 6).

CaCIPKs contained a protein kinase domain, a unique NAF motif and PPI motif (Additional file 5B). MEME analysis revealed 10 conserved motifs for CaCIPKs, which were named motif-1 to motif-10 (Fig. 2b**;** Additional file 7). CaCIPK10 did not have motif-1 and motif-2, CaCIPK11 did not contain motif-3, CaCIPK23 did not contain motif-7 and motif-9, and CaCIPK2, − 4, − 7, − 8, − 9, − 10, − 24, and − 25 did not have motif-10. In addition, motif-8 was very similar to the NAF motif.

### Chromosomal location and gene duplication of *CaCBL* and *CaCIPK* genes

**Fig. 3:**
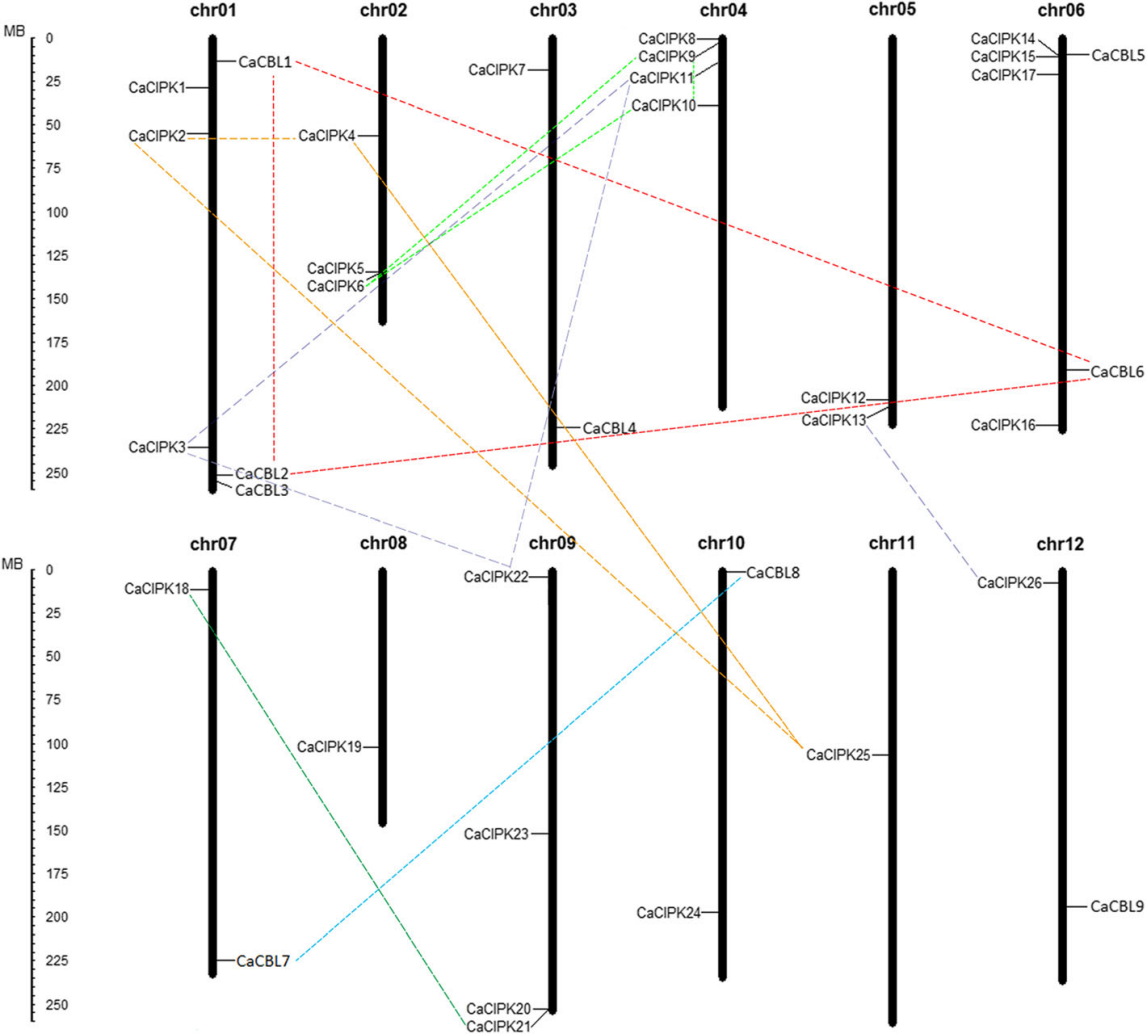
Location and duplications of *CBL* and *CIPK* genes on pepper chromosomes. The scale represents megabases (Mb). Gene pairs that resulted from a segmental duplication are connected by lines of different colors

*CaCBL* genes mapped onto six different chromosomes, including chromosome 1, 3, 6, 7, 10, and 12. Chromosome 1 contained three genes (*CaCBL1*, *− 2*, *− 3*), chromosome 3 had *CaCBL4*, chromosome 6 harbored *CaCBL5* and *CaCBL6*, chromosome 7, − 10 and 12 contained *CaCBL7*, *CaCBL8, CaCBL9*, respectively (Fig. 3). *CaCIPK* genes were distributed across 12 chromosomes. Chromosome 1 and 2 both had three genes (*CaCIPK1*, *− 2*, *− 3*, and *CaCIPK4*, *− 5*, *− 6*), and chromosome 4, 6, and 9 had four genes each. Chromosome 5 contained two genes (*CaCIPK12*, *− 13*), and the other chromosomes had only one *CaCIPKs*.

Two segmental duplication events were predicted for *CBLs* (*CaCBL1*, − *2*, − *6*, and *CaCBL7*, − *8*), and five duplication events were predicted for *CIPKs* (*CaCIPK2*, *− 4*, *− 25*, *CaCIPK3*, *− 11*, *− 22*, *CaCIPK6*, *− 9*, *− 10*, *CaCIPK13*, *− 26*, *CaCIPK18*, *− 21*) (Fig. 3). However, we did not identify any tandem duplication for *CaCBL* and *CaCIPK* genes. To explore the evolutionary constraints on *CaCBL* and *CaCIPK* gene families, the ratios of Ka (nonsynonymous) to Ks (synonymous substitutions) were estimated, and the results were less than 1 (Additional file 8). The chromosomal duplication events likely occurred between 106.34 (*CaCIPK18/21*) and 14.67 (*CaCIPK9/10*) million years ago.

### Interaction network of CaCBL and CaCIPK members

To investigate the relationship of CaCBLs and CaCIPKs, a protein-protein interaction network was built using homologs of the *Arabidopsis* interaction network (Additional file 9). Nine CaCBLs were homologous to five AtCBLs, and 26 CaCIPKs were homologous to 13 AtCIPKs. The co-expression network of CBLs and CIPK was calculated using the Pearson correlation coefficient (PCC), where the red lines indicate PCC > 0, and the green lines indicate PCC < 0. The analysis inferred that CaCBL1, − 2, − 3, − 4, − 6 may interact with CaCIPK1, − 4, − 13, − 15, − 16, − 18, − 21, − 23, − 24, and − 26, and CaCBL7 and CaCBL8 may interact with CaCIPK1, − 2, − 3, − 4, − 5, − 7, − 8, − 13, − 15, − 16, − 18, − 21, − 22, − 23, − 24, − 25, and − 26. The analysis also predicted that CaCBL5 interacted with CaCIPK20, and CaCBL9 interacted with CaCIPK4 and CaCIPK11.

### *Cis*-acting elements of *CaCBL* and *CaCIPK* genes in pepper

**Fig. 4:**
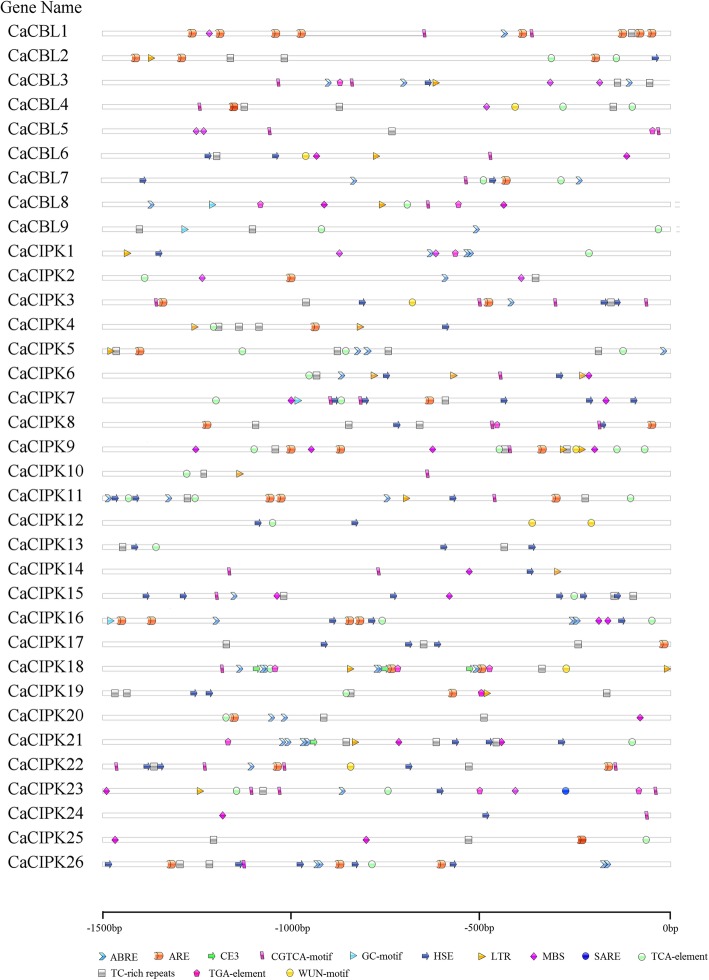
Predicted *cis*-acting regulatory elements in the promoter regions of *CBL* and *CIPK* genes. Promoter sequences (− 1500 bp) were analyzed by Plant CARE (Available online: http://bioinformatics.psb.ugent.be/webtools/plantcare/html/). Different shapes and colors represent different elements

To better understand how *CaCBL* and *CaCIPK* genes are regulated, the 1500 bp upstream sequences of the coding region were detected by PlantCARE (http://bioinformatics.psb.ugent.be/webtools/plantcare/html/) [42] to identify *cis*-acting elements (upstream sequences are shown in Additional file 10). Thirteen *cis*-elements, including ABRE (abscisic acid responsiveness), ARE (anaerobic induction), CE3 (ABA and VP1 responsiveness), CGTCA-motif (MeJA-responsiveness), GC-motif (enhancer-like element involved in anoxic specific inducibility), HSE (heat stress responsiveness), LTR (low-temperature responsiveness), MBS (MYB binding site involved in drought-inducibility), SARE and TCA-element (salicylic acid responsiveness), TC-rich (defense and stress), TGA-box (auxin-responsiveness) and WUN-motif (wound-responsiveness) were mapped onto the promoter regions (Fig. 4). ABRE elements were found in the promoter regions of five *CaCBL* genes (55.6%) including *CaCBL1*, *− 3*, *− 7*, *− 8*, and *− 9*, and 14 *CaCIPK* genes (53.8%) including *CaCIPK1*, *− 2*, *− 3*, *− 5*, *− 6*, *− 11*, *− 15*, *− 16*, *− 18*, *− 20*, *− 21*, *− 22*, *− 23*, and *− 26*. The TC-rich repeats elements were identified in the promoter regions of seven *CaCBL* (77.8%) and 21 *CaCIPK* genes (80.8%), excluding *CaCBL7*, *− 8*, *CaCIPK1*, *− 12*, *− 14*, *− 16*, and *− 24*. Interestingly, the promoter region of some *CIPKs* harbored at least one HSE elements, and the promoter of *CaCIPK15* had the highest number of HSE elements (6). In addition, the WUN-motif was the least common element in these genes, and only in two *CaCBL* (*CaCBL4*, *− 6*) and five *CaCIPK* genes (*CaCIPK3*, *− 9*, *− 12*, *− 18*, *− 22*) contained WUN-motifs upstream of their promoter. All *cis*-elements that were identified in this analysis are involved in stress response and hormone signaling.

### Subcellular localization of CaCIPK proteins

To explore the potential function of CaCIPK, we selected one member from every subfamily to study its subcellular localization. The subcellular localizations were detected in epidermal cells of *Nicotiana benthamian*a by *Agrobacterium*-mediated transient expression. CaCIPK1 was localized to the nucleus, plasma membrane and cytoplasm, CaCIPK5 and CaCIPK20 were localized to the plasma membrane, and CaCIPK7 was localized to the nucleus and plasma membrane. In particular, the CaCIPK15-GFP fusion protein was localized to organelles or other structures in the cytoplasm (Additional file 11)**.**

### Gene expression patterns in response to various stresses and developmental stages of pepper

**Fig. 5:**
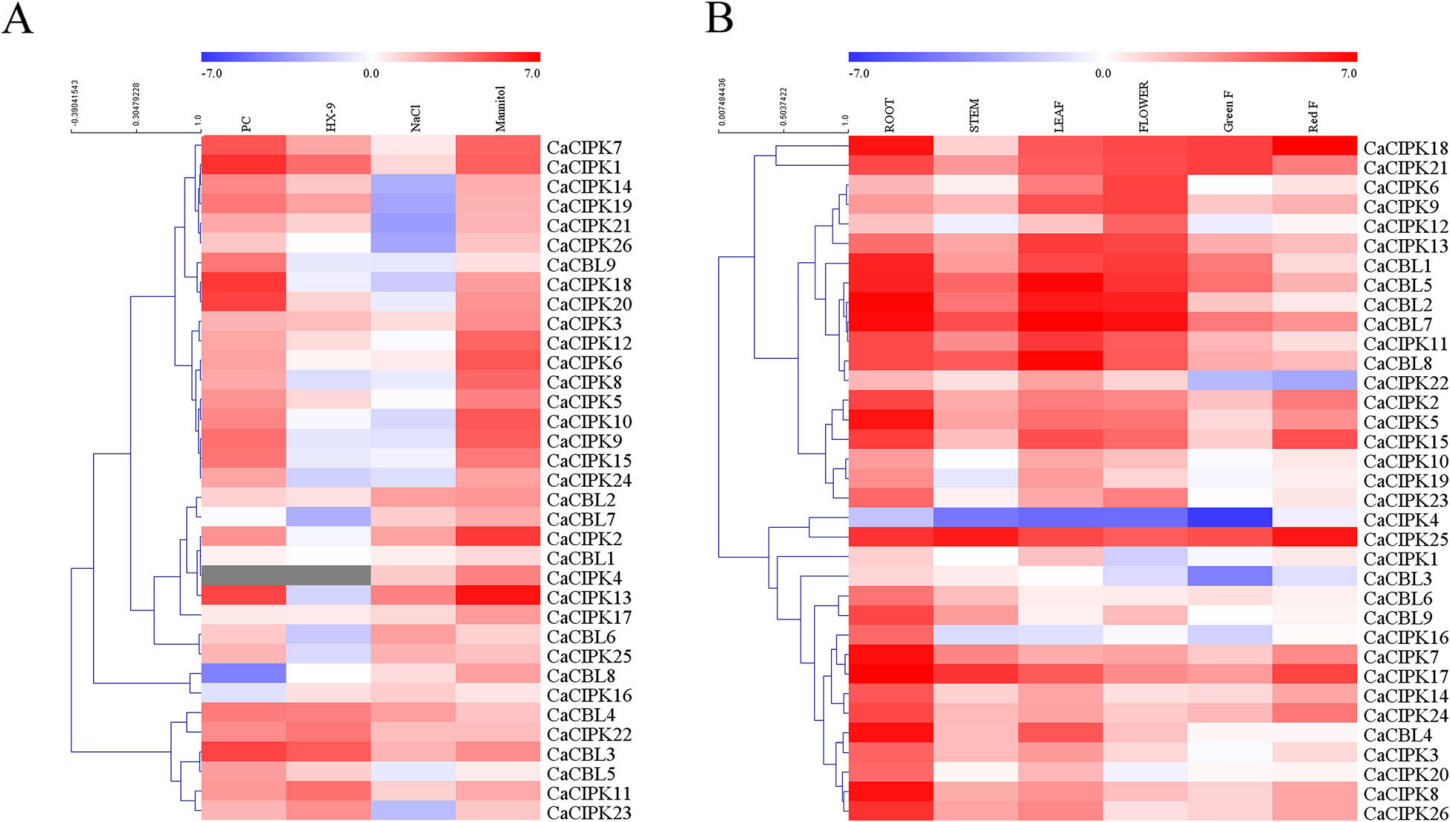
Expression patterns of *CaCBLs* and *CaCIPKs* in plants exposed to *P. capsici*, NaCl, and mannitol (**a**), and in different tissues (**b**). The grey means N/A. The samples in (**a**) were collected at different time points (6 hpt and 12 hpi for abiotic and biotic stresses respectively). “F” in (**b**) represents fruit. Actin (AY572427.1) is used as the internal control. Relative transcript levels were calculated using the comparative threshold (2^−ΔΔCT^) method, and normalized using log2. The heat map was created by MeV

The CBL-CIPK network regulates stress response against biotic and abiotic stressors. In order to explore whether the expression of *CaCBL* and *CaCIPK* genes change when exposed to various stressors, including NaCl, mannitol, incompatible PC strain and compatible HX-9 strain of *P. capsici*, we conducted qRT-PCR analyses to study the expression patterns of *CaCBLs* and *CaCIPKs*. We chose samples at 6 h post treatment (hpt) for abiotic stress and 12 h post-inoculation (hpi) for biotic stress, and compared expression to plants that were collected at the same time that were not exposed to biotic or abiotic stress (Fig. 5a).

Plants exposed to the incompatible PC strain of *P. capsici* showed up-regulation of six *CaCBLs* (*CaCBL2, − 3, − 4, − 5, − 6, − 9*) (log > 1) and down-regulation of one *CaCBL* (*CaCBL8*) (log < 1). *CaCBL1* and *CaCBL7* showed similar expression pattern as the control. When plants were exposed to the HX-9 strain, four *CaCBLs* (*CaCBL2*, *− 3*, *− 4*, − *5*) were up-regulated and three (*CaCBL6*, *− 7*, − *9*) were down-regulated. *CaCBL1* and *CaCBL8* showed similar expression pattern as the control. *CaCBL3* and *CaCBL4* were differentially expressed compared with *CaCBL* genes when plants exposed to *P. capsici*. *CaCBL3* expression was strongly induced by exposure to PC (36.16-fold) and HX-9 (22.74-fold). In the *CaCIPK* family, 23 *CaCIPKs* were up-regulated when exposed to the PC strain. Differentially expressed genes included *CaCIPK1*, *− 7*, *− 9*, *− 10*, *− 13*, *− 15*, *− 18*, *− 19*, and *− 20*. *CaCIPK1* expression was highly induced when exposed to the PC strain (51.68-fold) and HX-9 strain (16.34-fold). Eleven *CaCIPKs* were down-regulated when plants were treated with the HX-9 strain. *CaCIPK8* was up-regulated when exposed to the PC strain and down-regulated when exposed to the HX-9 strain. Interestingly, *CaCBL3/4* and *CaCIPK1* were significantly up-regulated when plants were exposed to *P. capsici*. *CaCBL9* and *CaCIPK9* were up-regulated when exposed to the PC strain, but down-regulated when exposed to the HX-9 strain. Based the same expression patterns, we speculated that *CaCBL3*, *− 4*, and *CaCIPK1*, and *CaCBL 9* and *CaCIPK 9* were involved in the pepper’s resistance against *P. capsici*.

In response to abiotic stresses, five *CaCBL* genes were up-regulated when exposed to NaCl and seven *CaCBL* genes were up-regulated when exposed to mannitol. *CaCBL3* was up-regulated by 4.25-fold when plants were exposed to NaCl and by 8.77-fold when exposed to mannitol. *CaCBL2* and *CaCBL9* were down-regulated when plants were treated with high levels of NaCl and mannitol. In the *CaCIPK* family, *CaCIPK1*, *− 2*, *− 3*, *− 4*, *− 6*, *− 7*, *− 11*, *− 13*, *− 16*, *− 17*, *− 22*, and *− 25* showed differential expression levels when treated with NaCl or mannitol. For instance, *CaCIPK1* was up-regulated by 2.13-fold when treated with NaCl and by 20.90-fold with mannitol. *CaCIPK5*, − *6*, *− 8*, *− 9*, *− 10*, *− 12*, *− 14*, *− 15*, *− 18*, *− 19*, *− 20*, *− 21*, *− 23*, *− 24*, and *− 26* were up-regulated in plants treated with mannitol (value ranged from 2.95 to 25.49-fold), but were not affected by NaCl stress. The same pattern was found in *CaCBL8*, indicating that they are co-regulated in response to osmotic stress.

To further investigate the spatial expression patterns of *CaCBLs* and *CaCIPKs* in pepper, qRT-PCR analysis was conducted on root, stem, leaf, flower, green fruit and red fruit. Expression levels of all genes were calculated relative to the expression levels of *CaCIPK1* in the stem (Fig. 5b). *CaCBL* and *CaCIPK* genes were constitutively expressed in different tissues. Most *CaCBL* and *CaCIPK* genes showed higher expression in the root and leaf tissues compared with other tissues. For example, *CaCBL4* expression was enriched in the root (95.80-fold) and leaf (24.98-fold), but was lower in stem (3.59-fold), green fruit (1.21-fold) and red fruit (1.19-fold). Expression levels of *CaCBL1*, − *5*, − *7*, and − *8*, and *CaCIPK17*, and − *25* were high in all tissues. For instance, *CaCIPK17* had the highest expression levels in all tissues compared to the other *CaCBL* and *CaCIPK* genes, especially in the root (662.41-fold). *CaCIPK18* and *CaCIPK25* were expressed in the red fruit (227.27-fold and 85.61-fold, respectively). These differential expression patterns of *CaCBLs* and *CaCIPKs* suggest that they may be involved in development and have tissue-specific functions.

### Expression pattern of *CaCIPK1* during stress and hormone treatment

**Fig. 6:**
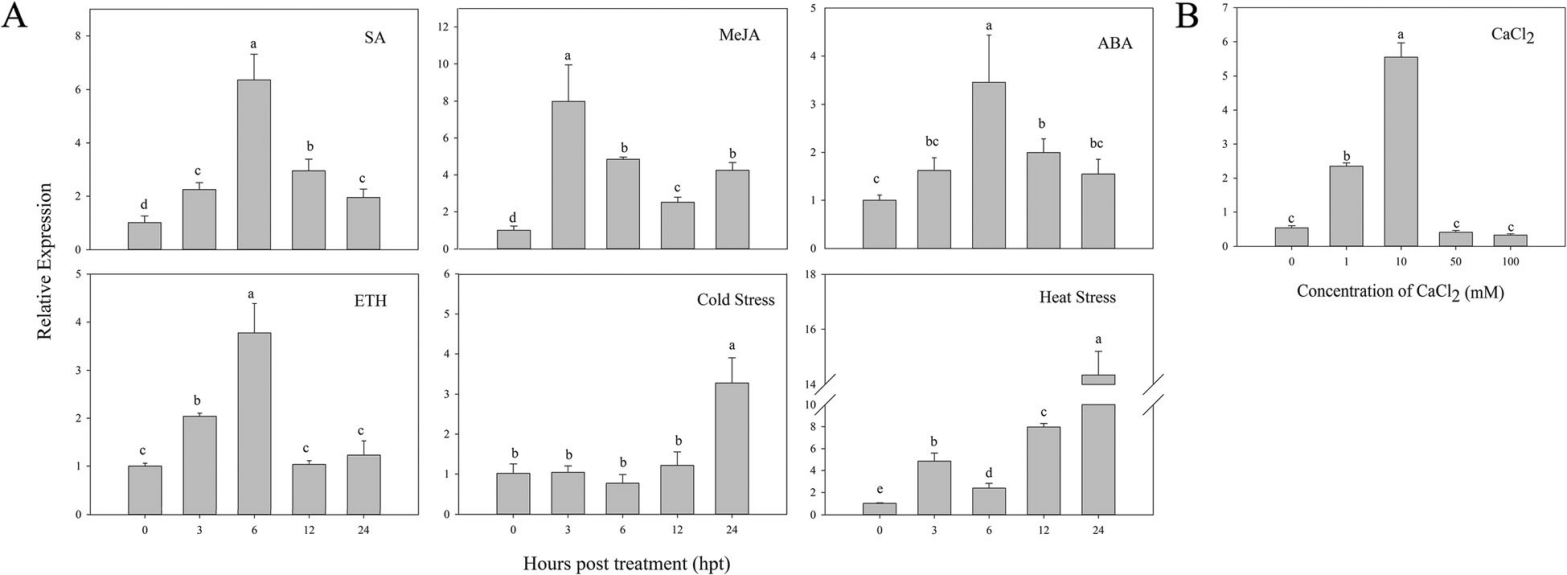
Expression patterns of *CaCIPK1* in plants exposed to SA, MeJA, ABA, ETH, cold (4 °C) and heat (42 °C) stress (**a**), and different concentration of CaCl_2_ (**b**). The samples in (**a**) were collected at different time points (0, 3, 6, 12, 24 hpt), and in (**b**) were at 6 hpt. Actin (AY572427.1) is used as the internal control. The error bars indicate the standard error (SE) for three replicates are showed. Letters (a–d) represent significant differences (LSD, *p* < 0.05)

Our results above showed that *CaCIPK1* expression increased under different treatments, especially when plants were infected with *P. capsici* and exposed to high levels of mannitol (Fig. 5). To obtain better insight into the role of *CaCIPK1* in mediating cell signaling and other stress response mechanisms, *CaCIPK1* expression levels were measured in plants treated with SA, MeJA, ABA, ETH, and low and high temperatures. *CaCIPK1* is a member of the *CIPK* family, which interacts with Ca^2+^ sensors. Therefore, leaves were sprayed with different concentrations of CaCl_2_ to explore whether *CaCIPK1* was involved in the plant’s response to Ca^2+^ stress (Fig. 6).

*CaCIPK1* expression was induced when leaves were sprayed with SA, MeJA, ABA and ETH. When sprayed with SA, *CaCIPK1* was up-regulated and reached to a peak (6.36-fold) at 6 hpt, and was down-regulated at subsequent time points. When leaves were sprayed with ABA and ETH, the highest *CaCIPK1* expression levels (3.46-fold and 3.77-fold respectively) were recorded at 6 hpt. Plants treated with MeJA showed highest *CaCIPK1* expression level at 3 hpt (7.98-fold). In response to cold stress, *CaCIPK1* expression steadily increased and reached highest expression level (3.28-fold) at 24 hpt. After heat stress, *CaCIPK1* expression changed dynamically, and was significantly up-regulated (14.34-fold) at 24 hpt. In addition, expression of *CaCIPK1* varied under different concentration of CaCl_2_ at 6 hpt. *CaCIPK1* expression increased with exposure to higher concentrations of CaCl_2_, and reached a 5.55-fold up-regulation at 10 mM. *CaCIPK1* expression was down-regulated when exposed to CaCl_2_ concentrations above 50 mM.

### VIGS of *CaCIPK1* increased pepper sensitivity to *P. capsici*

**Fig. 7:**
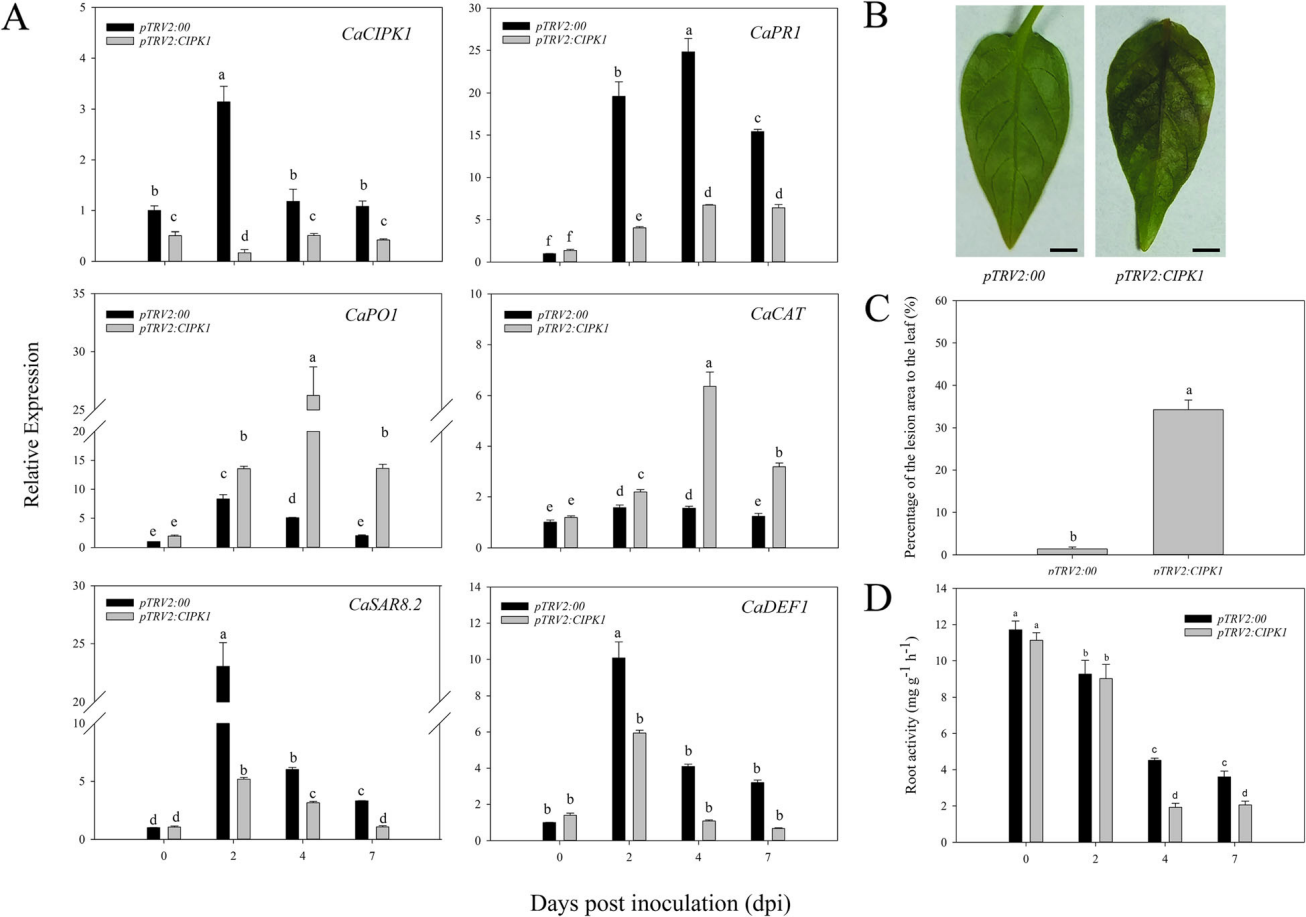
Knock-down of *CaCIPK1* reduces pepper’s defense response to PC strain. (**a**) The relative expression of *CaCIPK1*, *CaPR1*, *CaPO1*, *CaCAT*, *CaSAR8.2* and *CaDEF1* in silenced and control plants; (**b**) Disease symptoms grow on the detached leaves of silenced and control plants at 4 day post inoculation (dpi). The scale bar represents 5 mm; (**c**) Percentage of the lesion area of silenced and control plants; (**d**) Root activity in *CaCIPK1* silenced and control plants after inoculation with *P. capsici*. Error bars represent the mean ± SD of three independent biological replicates. Different letters (a–f) represent significant differences (LSD, *p* < 0.05)

To further explore the function of *CaCIPK1* in the pepper’s response to *P. capsici* infection, *CaCIPK1* loss-of-function peppers were generated by VIGS using the AA3 cultivar. At 5 weeks post-inoculation, the positive control with pTRV2:*CaPDS* (phytoene desaturase gene) showed photobleaching phenotypes. qRT-PCR analysis confirmed that *CaCIPK1* was silenced in the leaves, and *CaCIPK1* expression in the silenced plants (pTRV2*:CaCIPK1*) was 50 to 80% lower than the negative control (pTRV2:00) (Fig. 7a). To ensure the construct specifically targeted *CaCIPK1*, the *CaCIPK1* sequence was aligned with its homologous genes (*CaCIPK17* and − *19*) (Additional file 12A), and we found that the expression of *CaCIPK17* and *CaCIPK19* was not suppressed in pTRV2:*CaCIPK1* plants (Additional file 12B). The sensitivity of *CaCIPK1*-silenced plants to the avirulent *P. capsici* (PC strain) was explored using detached leaves assays. Leaves from negative control and *CaCIPK1-*silenced plants were sampled to match the same region that showed photobleaching in the positive control sample. Leaves were injected with zoospore suspension of the PC strain. Slight disease symptoms were detected at 2-day post inoculation (dpi) in the *CaCIPK1-*silenced plants, and these symptoms gradually became apparent at 4 dpi. In contrast, very few disease lesions were observed in the control (Fig. 7b). Quantitative analysis of the lesion area revealed that *CaCIPK1-*silenced plants had significantly larger lesion areas (34.28%) than the control (1.42%) (Fig. 7c).

To understand the molecular mechanisms underlying the increased sensitivity to the PC strain in the *CaCIPK1* knocked down plants, expression levels of defense related genes were measured. Defense genes that were studied included *CaPR1* (pathogenesis-related gene 1) [43], *CaDEF1* (defensin gene) [44], *CaSAR8.2* (systemic acquired resistance gene) [45], *CaPO1* (peroxidase) [46], and *CaCAT* (catalase, highly homologous with *AtCAT*), and their expression patterns were examined at 2, 4, and 7 dpi (Fig. 7a). Knocking down *CaCIPK1* resulted in a significant decline in the expression of *CaPR1*, *CaSAR8.*2 and *CaDEF1*. Control plants had 4.83-fold higher expression of *CaPR1* than the *CaCIPK1-*silenced plants at 2 dpi. *CaSAR8.2* and *CaDEF1* showed 80% decrease in expression in *CaCIPK1-*silenced plants at both 2 dpi and 7 dpi. The expression of *CaPO1* and *CaCAT* were significantly up-regulated in *CaCIPK1-*silenced plants, and the fold change was as high as 5-fold and 4-fold compared to the control at 4 dpi, respectively.

Furthermore, the vigor of the metabolism in the root system was measured by assessing root activity using triphenyltetrazolium chloride (TTC) [47]. TCC was reduced in the control and *CaCIPK1-*silenced plants that were treated with *P. capsici* (Fig. 7d). Root activity was reduced in both plants; however, *CaCIPK1-*silenced plants that were infected with *P. capsici* had significant lower root activity than control plants. The lowest activity was recorded in *CaCIPK1-*silenced plants at 4 dpi, where the activity was reduced by 57.3% compared to the control.

### Transient expression of *CaCIPK1* in pepper leaves

**Fig. 8:**
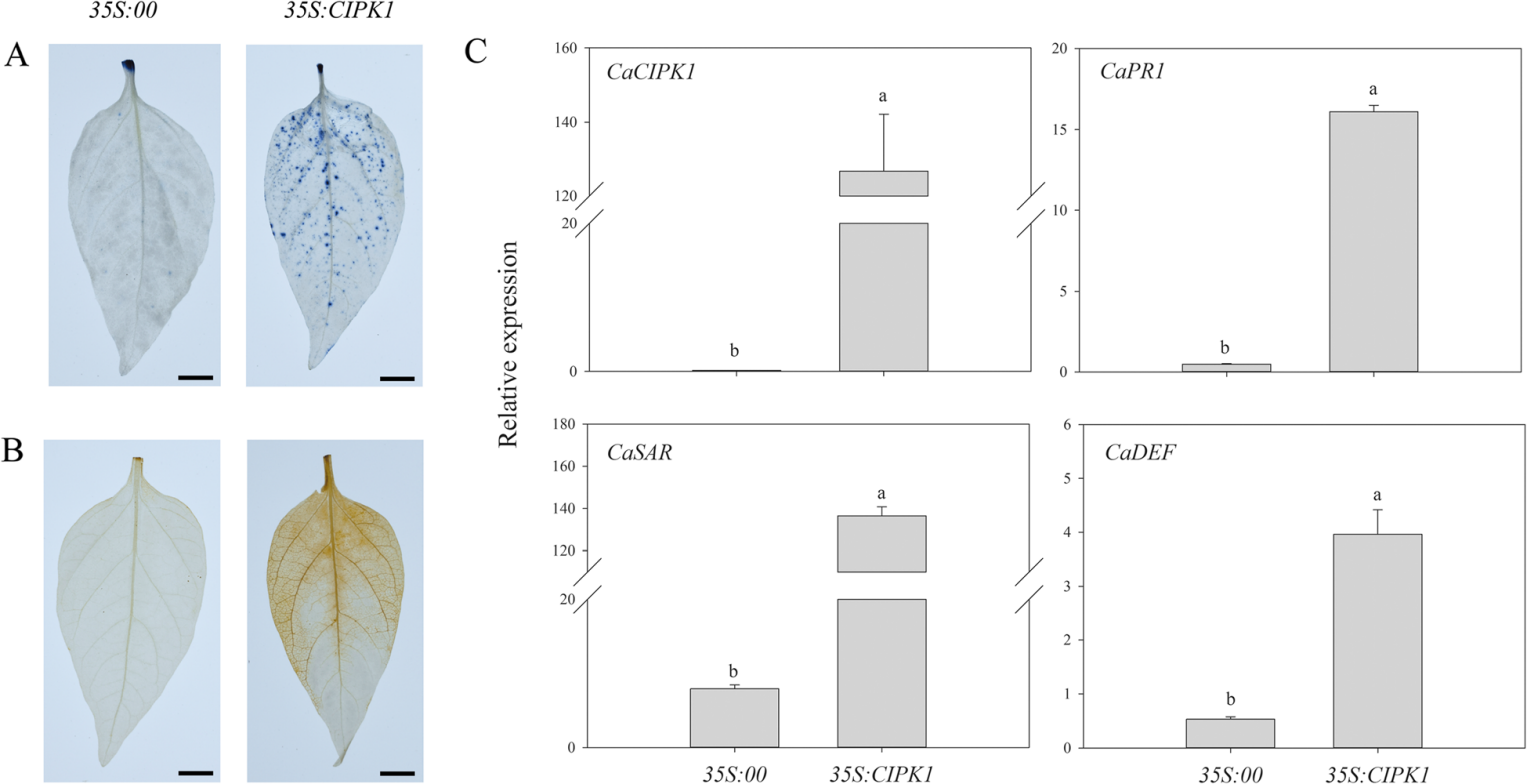
*Agrobacterium*-mediated *CaCIPK1* transient expression in pepper leaves. **a** Trypan blue staining of cell death in leaves; **b** DAB staining of H_2_O_2_ accumulation in leaves after agroinfiltration; (**c**) The relative expression of *CaCIPK1*, *CaPR1*, *CaSAR8.2* and *CaDEF1* in leaves. Data are means ± SD deviations from three independent experiments. Different letters indicate significant differences (LSD, *P* < 0.05). The scale bar represents 5 mm

Knocking down *CaCIPK1* increased the expression of *CaPO1* and *CaCAT*, two genes that regulate ROS levels, therefore we explored whether *CaCIPK1* is involved in ROS accumulation. The vectors 35S:00 and 35S:*CIPK1* were ectopically overexpressed in pepper leaves using *Agrobacterium*. We measured cell death, H_2_O_2_ production, and expression of defense-related genes at 24 h post agroinfiltration. Transient expression of *CaCIPK1* induced cell death and enhanced H_2_O_2_ accumulation compared with samples infected with the empty vector control (Fig. 8a, b). *CaCIPK1* expression increased by 126.76-fold in the infected samples compared to the control, and *CaPR1*, *CaDEF1*, *CaSAR8.2* expression increased by 16.11-fold, 3.96-fold and 136.54-fold, respectively (Fig. 8c).

## Discussion

Calcium is a core regulator of plant development and responses to the environment [3]. The Ca^2+^ sensor CBLs and their target protein kinases CIPKs, comprise a complicated signaling network that allows plants to adapt to developmental and environmental stress. CBL-CIPK modules are involve in ion channel, phytohormones signaling network, growth and development [1, 12–15]. Past studies showed that multiple *CBL* and *CIPK* genes are found in many plant species, such as *Arabidopsis thaliana* (10 *CBLs* and 26 *CIPKs*), *Manihot esculenta* (8 *CBLs* and 26 *CIPKs*), *Oryza sativa* (10 *CBLs* and 34 *CIPKs*), *Brassica napus* (7 *CBLs* and 23 *CIPKs*), *Populus trichocarpa* (10 *CBLs* and 27 *CIPKs*), *Triticum aestivum* (24 *CBLs* and 79 *CIPKs* loci in sub-genomes A, B, and D respectively), and *Vitis vinifera* (8 *CBLs* and 20 *CIPKs*) [14, 40, 41, 48–54]. In this study, nine *CBL* and 26 *CIPK* genes were identified using the CM334 [55] and Zunla-1 [56] pepper genomes. MSA of CaCBLs found that all members contained four EF-hands, which consisted of 12 relatively conserved amino acids. Interestingly, the amino acids numbers between adjacent EF-hand were same. EF1 and EF2 were 23 amino acids apart, EF2 and EF3 were 25 amino acids apart, and 32 amino acids separated EF3 from EF4. The number of amino acids separating adjacent EF-hands were not consistent with findings from *Arabidopsis thaliana*, *Oryza sativa* and *Vitis vinifera*, where there were 22 amino acids between EF1 and EF2 [49, 52]. To further explore the numbers of amino acids between EF-hands in other species, CBLs in *Arabidopsis thaliana*, *Capsicum annuum, Manihot esculenta*, *Populus trichocarpa*, and *Triticum aestivum* were tested again for protein structure. We found there were consistently 23 amino acids between EF-1 and EF-2, unlike previous studies that identified 22 amino acids, and this is potentially due to differences in methods for counting amino acids between the domains (Additional file 13).

To uncover the potential function of CaCBLs and CaCIPKs, we performed phylogenetic analysis and MSA (Fig. 1**;** Additional file 5). Of the genes that belonged to group II (Fig. 1a), CaCBL5 had a transmembrane helix that may target the protein to the plasma membrane, similar to AtCBL10, OsCBL9, OsCBL10, and TaCBL9 [40]. Group IV contained the tonoplast targeting sequences (TTS). However, one member, CaCBL7, did not have the tonoplast targeting motif, similar to AtCBL7 [57, 58]. Compared with *Arabidopsis*, CaCBL8 and AtCBL2/3 contained TTS, suggesting that it is localized to the tonoplast [40, 59]. Additionally, five CaCBLs (CaCBL1, − 2, − 3, − 4 and − 6) had a myristoylation motif in the N-terminus, and this motif is required for binding to the membrane and interacting with SOS3 under salt stress [7, 60]. In addition, there was a PFPF motif at the C-terminus, and the motif contained a conserved serine residue known to interact with CIPKs. Interestingly, *CIPKs* in group B, such as *CaCIPK2*, *− 4*, *− 11*, *− 13*, *− 20*, *− 25*, and *− 26*, contained more than 10 introns, making this an intron-rich clade (Fig. 1b; Additional file 3). *CaCIPKs* in other groups contained zero or one intron, suggesting that they are an intron-poor clade. *CIPK* genes were also classified into intron-rich and intron-poor clades, such as in *Arabidopsis thaliana*, *Triticum aestivum*, and *Oryza sativa* [41, 52]. However, the number of introns in *CBLs* did not show a specific pattern. Hence the classification method using intron numbers is unique to the *CIPK* family. CaCIPKs had the conserved N-terminal kinase domain, C-terminal regulatory domain and NAF/FISL motif (Additional file 5B). The NAF domain consists of the conserved amino acids asparagine (N), alanine (A), and phenylalanine (F), and mediates the interaction with CBL. Next to the NAF motif, there was the PPI domain, which interacts with PP2C [8–10]. The N-terminal catalytic kinase domain has an ATP binding site and an activation loop [61].

In the gene duplication analysis, we found that the *CaCBL* family underwent two segmental duplication events (*CaCBL1*, − *2*, − *6*, and *CaCBL7*, − *8*) in group III and group IV. The *CaCIPK* family harbored two segmental duplication events (*CaCIPK6*, *− 9*, *− 10*, and *CaCIPK18*, *− 21*) in group A, and three (*CaCIPK2*, *− 4*, *− 25*, *CaCIPK3*,*-11*,*-22*, and *CaCIPK13*, *− 26*) in group B. Tandem duplication events were not detected in *CaCBL* and *CaCIPK* genes. These results suggested that segmental duplications may have contributed to the complexity and diversity of both gene families. Additionally, the Ka/Ks ratio of *CaCBL* and *CaCIPK* genes (less than 1) inferred that the duplicated genes were maintained by purifying selection. The earliest duplication was for *CaCIPK18* and *CaCIPK21*, and it was estimated to have occurred around 106.34 million years ago (Additional file 8).

The role of CBL-CIPK complexes in regulating plant development and response to environmental stress has been studied in different plant species, such as *Arabidopsis* and wheat [1, 3, 61]. To better understand the function of *CaCBLs* and *CaCIPKs*, we compared genes belonging to these families from pepper and *Arabidopsis* using a phylogenetic analysis (Additional file 14). *CaCBL3* and *CaCIPK20* were orthologous to *AtCBL5* and *AtCIPK24*, respectively. AtCBL5-AtCIPK24 proteins are localized to the plasma membrane. Overexpression of *AtCBL5* enhances salt and drought stress tolerance, and *AtCIPK24* modulates cellular responses to salt stress and activate the Na^+^/H^+^ antiport activity of SOS1 [19, 21, 61, 62]. In pepper, *CaCBL3* was up-regulated when exposed to salt (4.25-fold) and mannitol (8.77-fold) stress, and *CaCIPK20* was also up-regulated when plants were exposed to mannitol (7.90-fold). MBS elements regulate gene expression under drought conditions, and these elements were found in the promoter region of *CaCBL3* and *CaCIPK20*, suggesting that *CaCBL3* may interact with *CaCIPK20* to regulate drought stress tolerance (Figs. 4, 5a). PAT10 (Protein S-Acyl Transferase10)-CBL2/3-CIPK9/17 complexes regulate ABA signaling during stomatal movement in *Arabidopsis* [15, 17]. *CaCBL7* and *CaCBL8* were orthologous to *AtCBL2* and *AtCBL3*, respectively. They contained ABRE elements in the promoter regions, suggesting that they may be regulated by PAT in ABA signaling. In wheat, TaCBL4 interacts with TaCIPK5 to positively modulate wheat resistance against fungus [40]. The genes orthologous to *TaCBL4* and *TaCIPK5*, *CaCBL9* and *CaCIPK9/10*, were up-regulated when plants were exposed to the PC strain, and down-regulated when exposed to the HX-9 strain. We speculate that CaCBL9-CaCIPK9/10 may regulate the pepper plant’s resistance against *P. capsici*.

The expression levels of *CaCBLs* and *CaCIPKs* were detected by qRT-PCR in plants exposed to pathogen, salt, and mannitol (Fig. 5a). However, expression patterns of *CaCBL* and *CaCIPK* genes were variable within the different subfamilies. For instance, *CaCBL3* and *CaCBL4* were up-regulated when exposed to stress, but *CaCBL9* was up-regulated in plants treated with mannitol and incompatible *P. capsici*. *CaCIPK13* was up-regulated by exposure to salt (10.87-fold), mannitol (89.38-fold) and incompatible *P. capsici* (35.20-fold), while *CaCIPK26* was slightly up-regulated by exposure to mannitol (3.17-fold) and incompatible *P. capsici* (2.94-fold). *CaCIPK9* was derived from a segmental duplication of *CaCIPK10*, and these two genes showed similar expression patterns. Nevertheless, other paralogs did not show similar patterns. In contrast, many *CaCBLs* and *CaCIPKs* genes were expressed at higher levels during different developmental stages (Fig. 5b). For instance, *CaCIPK18* was expressed at the highest level in all stages, specifically in the red fruit (227.27-fold), inferring that it may have vital functions during different developmental stages. The expression level of *CaCIPK4* was lower than other genes in all tissues, and its expression levels did not change when plants were exposed to *P. capsici*. Therefore, CBL-CIPK may play an important role in the response to biotic and abiotic stimuli, rather than development in pepper.

Five genes from each subfamily in *CaCIPKs* were chosen for subcellular localization assay. CaCIPK1 was localized to the nucleus, plasma membrane and cytoplasm, similar to TaCIPK14 and TaCIPK23 [34, 63]. Overexpression of TaCIPK14 enhances cold and salt stress tolerance in tobaccos. CaCIPK1 and TaCIPK14 belong to the same clade in Group I, therefore CaCIPK1 may also be involved in regulating tolerance against abiotic stress. CaCIPK5 and CaCIPK20 were localized to the plasma membrane. CaCIPK7 was localized to the nucleus and plasma membrane, similar to AtCIPK21, MeCIPK23 and BnaCIPK24 [64–66]. CaCIPK15 was targeted to organelles in the cytoplasm, deviating from the patterns observed in other CaCIPKs in this study (Additional file 11)**.** In most plants, CIPKs are recruited by CBLs to the plasma membrane or tonoplatst to form a complex [21, 67], and it is likely that the interaction between CBLs and CIPKs influenced the plasma membrane localization of the five CIPKs were analyzed in this study.

While the role of the CBL-CIPK network in regulating response against abiotic stress is well documented, it remains unclear whether this network also regulates responses against biotic stress. *OsCIPK14* and *OsCIPK15* are rapidly induced by MAMPs in rice, and RNAi against these genes reduces sensitivity to *Trichoderma viride*/ethylene-inducing xylanase [68]. The TaCBL4-TaCIPK5 complex positively contributes to the interaction of wheat and *Puccinia triiformis* f. sp. *tritici* through ROS signaling [40]. *CaCIPK1* was strongly up-regulation in the root of plants that were infected with incompatible (51.68-fold) and compatible (16.34-fold) *P. capsici*, indicating that *CaCIPK1* may be involved in resistance against *P. capsici* in pepper. Moreover, the subcellular localization of CaCIPK1 was similar to TaCIPK14 and TaCIPK23. Overexpression of *TaCIPK14* enhances cold and salt stress tolerance and *TaCIPK23* positively regulates drought stress and ABA responses [34, 63], and *CaCIPK1* may have similar functions. Meanwhile, the *CaCIPK1* promoter region contained ABRE, HSE, MBS, LTR, and TCA *cis*-acting elements, which are involved in ABA signaling, heat stress, drought, low temperature and SA (Fig. 4). The presences of these elements suggest that *CaCIPK1* is involved in abiotic and phytohormone stress response, and we tested the expression pattern of *CaCIPK1* to verify this hypothesis (Fig. 6). SA, MeJA, ABA, and ETH are central regulators of defensive signaling and plant innate immunity [69, 70]. The results implied that *CaCIPK1* may play a crucial role in defensive and innate immunity in pepper.

To confirm that *CaCIPK1* is involved in mediating the interaction between pepper and incompatible *P. capsici*, TRV-VIGS was used to successfully knock down *CaCIPK1*. The silenced leaves were detached [71] and inoculated with the PC strain. There were larger lesions in the *CaCIPK1*-silenced plants compared to the control (24.06-fold), indicating that knocking down of *CaCIPK1* increased sensitivity to incompatible *P. capsici* (Fig. 7c). Similar results were observed in wheat, where knockdown of *TaCBL4* and *TaCIPK5* reduced the defense response of wheat against stripe rust fungus [40]. To further study the role of *CaCIPK1* in plants that were infected with the PC strain, root activity assays were conducted to detect the effects of the fungus on *CaCIPK1*-silenced plants. When the inoculation time was prolonged, root activity in *CaCIPK1*-silenced plants was lower than control plants, and there was a significant decrease in root activity at 4 and 7 dpi (Fig. 7b). These results showed that *CaCIPK1*-silenced plants had reduced resistance against incompatible *P. capsici* compared with control plants. In addition, *CaPR1*, *CaSAR8.2*, and *CaDEF1* expression levels were lower in *CaCIPK1*-silenced plants that were exposed to PC (Fig. 7a). *CaPR1* and *CaSAR8.2* are genes involved in pathogenesis and systemic acquired resistance, and are induced by SA signaling when plants are exposed to biotic stress [40, 43]. Interestingly, *CaCIPK1* expression was induced by SA (Fig. 6). Taken together, *CaCIPK1* may be involved in the SA pathway to defend the pepper plant against incompatible *P.capsici*. *CaCIPK1*-silenced plants that were exposed to PC had higher expression levels of *CaPO1* and *CaCAT*, which may lead to lower H_2_O_2_ accumulation. Previous studies have postulated that the accumulation of ROS (e.g., H_2_O_2_) and the release of Ca^2+^ positively regulate each other [72]. To verify whether *CaCIPK1* is involved in H_2_O_2_ signaling, *CaCIPK1* was transiently over-expressed in pepper. Plants over-expressing *CaCIPK1* had higher cell death and H_2_O_2_ accumulation, and up-regulation of defense-related genes *CaPR1* (16.11-fold) and *CaSAR8*.*2* (136.54-fold) (Fig. 8). Thus, we hypothesize that CaCIPK1 is involved in H_2_O_2_ and SA signaling to modulate *P. capsici* tolerance by interacting with CBLs. Nevertheless, more studies are necessary to clarify the molecular mechanisms by which *CaCIPK1* regulates the resistance of pepper against *P. capsici*.

## Conclusions

The CBL-CIPK signaling pathway is a Ca^2+^-related pathway that regulates the plant’s response to environmental stimuli and ion stress [1]. Here, we identified nine *CaCBL* and 26 *CaCIPK* genes in pepper and most genes were highly expressed in different developmental stages. These genes also showed varying responses to biotic and abiotic stressors, suggesting that they may be crossing nodes of different signaling networks. Furthermore, *CaCIPK1* expression levels changed in response to various stresses, including exposure to *P. capsici*, abiotic stress, and phytohormones. Knockdown of *CaCIPK1* decreased the resistance of pepper against *P. capsici*, and changed the expression of defense related genes and root activity. Transient expression of *CaCIPK1* in pepper leaves enhanced H_2_O_2_ accumulation and cell death. In brief, our study establishes a basic foundation for further research on the function of *CaCBL* and *CaCIPK* genes, and report a preliminarily exploration of the role of *CaCIPK1* in pepper’s resistance to *P. capsici*. Further investigations are required to reveal the mechanism by which *CaCIPK1* regulates resistance against *P. capsici* in pepper.

## Methods

### Genome-wide identification of *CBL* and *CIPK* genes in pepper

The proteome of pepper was downloaded from the Pepper Genome Database (CM334, http://peppergenome.snu.ac.kr/) and Zunla-1 (http://peppersequence.genomics.cn/). CBLs contain four unique EF-hands, and the CIPKs have a highly conserved protein kinase domain and NAF domain. The HMM profile of EF-hand (PF00036) and NAF (PF03822) were obtained from the Pfam (http://pfam.sanger.ac.uk/) protein family database. These domains were used as queries to search the pepper genome database with the BLASTP program (E-value ≤1.0E-3) [73]. All candidate CaCBLs were submitted to InterProScan (http://www.ebi.ac.uk/Tools/InterProScan) and SMART (http://smart.embl-heidelberg.de) to ensure the presence of the four EF-hand domains, and the same method was used to ensure CaCIPKs contained the Pkinase domain (IPR000719) and the NAF domain (IPR004041). Sequences were aligned with ClustalX (version 2.1) (http://www.clustal.org) to ensure that the *CBL* and *CIPK* genes that were identified in the analysis aligned to the CM334 and Zunla-1 genome sequences. When the sequences were different in CM334 and Zunla-1 databases, we designed the two sets of primers that were specific to the gene of interest in the different strains, and the PCR products were used to clone the gene. The sequences were aligned with ClustalX to identify the sequence of the gene of interest in cultivar AA3. The deduced amino acid and CDS sequences, theoretical isoelectric point (*p*I), instability index (with a value < 40 regarded as stable) [74] and protein molecular weight (MW) were analyzed by ExPASY (https://web.expasy.org/translate/; https://web.expasy.org/ protparam/). The WoLF PSORT program (http://wolfpsort.org/) was used to predict the subcellular localizations. Nomenclature of the *CaCBL* and *CaCIPK* genes were based on their chromosomal order.

### Phylogenetic analysis

The full-length amino acid sequences of CBL and CIPK protein from *Capsicum annuum*, *Arabidopsis thaliana*, *Manihot esculenta*, *Oryza sativa*, *Brassica napus*, *Populus trichocarpa*, and *Triticum aestivum*, were aligned as an unrooted neighbor-joining phylogenetic tree using MEGA-X [75] with the bootstrap test replicated 1000 times. The classification of CaCBLs and CaCIPKs were based on previous research in *Arabidopsis thaliana* and *Populus trichocarpa* [50] and had high bootstrap values (> 50). The full-length amino acid sequences of genes from the species listed above were acquired from NCBI databases (http://www.ncbi.nlm.nih.gov/), Plant Genome Resource (https://phytozome.jgi.doe.gov/pz/portal.html), and TAIR database (https://www.arabidopsis.org/index.jsp). The sequences are displayed in Additional file 4.

### Sequence analysis

The Multiple Sequence Alignment was executed using ClustalX to detect conserved domains. The position and number of introns in *CaCBL* and *CaCIPK* were visualized using Gene Structure Display Server (GSDS, http://gsds.cbi.pku.edu.cn/index.php) [76]. The conserved motifs of CaCBLs and CaCIPKs were identified and analyzed using protein sequences in the MEME online tool (http://meme-suite.org/tools/meme). The site distribution was selected as any number of repetitions, the optimum width of motifs ranged from 10 to 50 (10 to 200 for CIPK), and the maximum numbers of motifs were identified as 6 for CaCBLs and 10 for CaCIPKs. Motifs with position *p*-values less than 0.0001 are shown.

### Chromosomal location and gene duplication

The chromosomal location of *CaCBL* and *CaCIPK* genes were identified using MapDraw [77]. Tandem duplications were defined as adjacent homologous genes on the same chromosome with a distance of less than 50-kb [78]. If they were paralogs located on duplicated chromosomal blocks, they were defined as a segmental duplication event [79]. The non-synonymous substitutions (Ka) and synonymous substitutions (Ks) were calculated by MEGA-X and DnaSP v6 [75, 80]. The divergence time (Mya, million years ago) was calculated as T = Ks / (2 × 6.1 × 10^− 9^) × 10^− 6^ [81].

### Prediction of protein-protein interaction network

The protein-protein interaction relationships were tested to establish the genome-wide regulation network. Since there were no references about the interaction of CBL and CIPK proteins in pepper, homologous genes from *Arabidopsis* was used to predict the protein-protein interaction network for CBLs and CIPKs from pepper. First, CBL and CIPK proteins from *Arabidopsis* were analyzed using *Arabidopsis* Interactions Viewer (http://bar.utoronto.ca/interactions/cgi-bin/arabidopsis_interac tions_viewer.cgi). The homologs were identified in pepper, and the corresponding interaction network was created by Cytoscape 3.6.0 (National Institute of General Medical Sciences, MD, USA) [82].

### *Cis*-acting elements in the promoters of *CaCBL* and *CaCIPK* genes

Regulatory elements of the promoter sequences can control gene expression [42]. The upstream regions (1500 bp) of *CaCBL* and *CaCIPK* genes were obtained from the PGD and Zunla-1 genomes, and regulatory elements, including ABRE (abscisic acid responsiveness), ARE (anaerobic induction), CE3 (ABA and VP1 responsiveness), CGTCA-motif (MeJA-responsiveness), GC-motif (enhancer-like element involved in anoxic specific inducibility), HSE (heat stress responsiveness), LTR (low-temperature responsiveness), MBS (MYB binding site involved in drought-inducibility), SARE and TCA-element (salicylic acid responsiveness), TC-rich (defense and stress), TGA-box (auxin-responsiveness) and WUN-motif (wound-responsiveness) were identified using the plant promoter database PlantCARE (http://bioinformatics.psb.ugent.be/webtools/plantcare/html/) [42, 83].

### Subcellular localization

The full-length cDNA sequences of *CaCIPK1*, *CaCIPK5*, *CaCIPK7*, *CaCIPK15*, and *CaCIPK20* were cloned from the root tissue of AA3 and inserted into the pVBG2307 vector [84], which was modified with the green fluorescent protein (GFP) under the control of the 35S promoter. Constructs were introduced into the *Agrobacterium* strain GV3101. *Agrobacterium* cultures were grown overnight, and resuspended in 10 mM MES (pH 5.7) with 400 mM acetosyringone (3,5-dimethoxy-4′-hydroxy-acetophenone). Subcellular localization was observed following methods described by Wydro et al. [85]. The infiltrated *Nicotiana benthamian*a leaves were imaged 2 days after agroinfiltration using the OLYMPUS BX63 automated fluorescence microscope (OLYMPUS Corporation, Tokyo, Japan).

### Plant materials, RNA extraction and quantitative RT-PCR

The pepper cultivar AA3 was obtained from the College of Horticulture, Northwest A&F University. AA3 is compatible with the HX-9 strain of *P. capsici* (virulent) and incompatible with the PC strain (avirulent)*.* The plants were grown on soil and vermiculite (1:1), and cultivated in growth chambers (24/20 °C day/night temperature and 16/8 h day/night photoperiod). When plants reached the 6–8 true leaves stage, zoospore suspension of *P. capsici* was inoculated using the root drenching method as described previously [86]. Root samples were collected from treated (*P. capsici*) and control (treated with sterile water) plants, and collected at 0 and 12 hpi and immediately stored at − 80 °C. To induce NaCl and mannitol stress, seedlings at 6–8 true leaves stage were pre-hydroponically cultivated in sterile water for 2 days, and then treated with NaCl (200 mM) and mannitol (300 mM) hydroponically. Root samples were collected at 0 and 6 hpt [84]. Root, stem, leaf, flower, green fruits and red fruits were collected from normal AA3 plants grown in soil, frozen in liquid nitrogen and stored at − 80 °C for tissue-specific experiments.

Total RNA was extracted using the Trizol Reagent (Invitrogen, Carlsbad, CA), and reverse-transcribed using PrimeScript™ RT reagent Kit with gDNA Eraser (Takara, Dalian, China). Primer Premier 5.0 was used to design primer pairs against *CaCBL* and *CaCIPK* genes for Quantitative Real-Time PCR analysis. The primer’s specificity was tested by NCBI Primer BLAST (https://www.ncbi.nlm.nih.gov/tools/primer-blast/index.cgi) and checked by electrophoresis in 1.5% (w/v) agarose gel. All the primers used in this study are listed in Additional file 15. Actin was used as the reference gene [87]. Quantitative real-time PCR (qRT-PCR) was used to detect expression levels by SYBR Green Supermix (Takara, Dalian, China) on IQ5.0 Bio-Rad iCycler thermocycler (Bio-Rad, Hercules, CA, USA). All experiments included three independent biological replicates. The relative expression levels of pepper *CBL* and *CIPK* genes were calculated using the comparative 2^−ΔΔCT^ method [88]. After normalizing dates by log2, a heatmap was drawn by Multi experiment viewer (MeV, http://www.tm4.org/mev.html).

### Expression profile of *CaCIPK1*

The pepper line AA3 was grown to the 6–8 true leaf stage and sprayed with 5 mM SA, 50 μM MeJA, 0.57 μM ABA and 10 mM ETH solutions to detect changes in the expression level of *CaCIPK1*. Control plants were treated with sterile water. Plants that were at the same growth stage were placed at 4 °C and 42 °C for cold and heat stress, and leaves were collected at 0, 3, 6, 12, and 24 hpt. For CaCl_2_ stress, plants were sprayed with 0, 1, 10, 50, 100 mM CaCl_2_ and leaves were collected at 6 hpt. The samples were frozen in liquid nitrogen and stored at − 80 °C for RNA extraction.

### Virus-induced gene silencing (VIGS) of *CaCIPK1* in pepper

To construct the tobacco rattle virus (TRV) vector, primers for *CaCIPK1* were designed by Sol Genomics Network (http://vigs.solgenomics.net/). A 245 bp fragment of the *CaCIPK1* ORF was amplified using the specific primer pair vigs-F and vigs-R from cDNA isolated from the AA3 root grown under normal conditions (Additional file 12). The fragment was inserted into the original TRV vector for gene silencing. *Agrobacterium* strain GV3101 harboring pTRV1 was mixed at a 1:1 ratio with pTRV2 (negative control), pTRV2:*CaPDS* (silencing the phytoene desaturase gene, which induces bleaching) and pTRV2*:CaCIPK1*. The mixture was injected into fully extended cotyledons leaves [89]. Plants were cultivated in growth chambers (22/18 °C day/night temperature and 16/8 h day/night photoperiod). After 4 weeks, when the positive control (pTRV2:*CaPDS*) showed the photobleaching phenotype, leaf samples were collected from pTRV2:*CaCIPK1* and pTRV2 to test the silencing efficiency by qRT-PCR. pTRV2:*CaCIPK1* and control plants were treated with incompatible *P. capsici* (PC strain), and the third to fifth leaf from top of the control and *CaCIPK1* silenced plants were picked. Leaves were washed with sterile water and injected with a 20 μL zoospore suspension (1 × 10^5^ zoospores mL^− 1^) of incompatible *P. capsici*. Leaves were then moved into petri dishes and sealed with parafilm [86].

### *Agrobacterium*-mediated transient expression assays

*Agrobacterium* strain GV3101 carrying 35S:00 (empty-vector) or 35S:*CIPK1* was used for transient expression assays. Fresh pepper leaves were placed into resuspended 10 mM MES (pH 5.7, 200 mM acetosyringone) with 35S:00 and 35S:*CIPK1*, and vacuum infiltrated (OD_600_ = 1.0). Pepper leaves were photographed at 24 h after agroinfiltration using a Nikon D3300 (Nikon Corporation, Tokyo, Japan).

### Histochemical analyses

Root activity was measured using TTC under a modified protocol by Wang et al. [90]. H_2_O_2_ production was detected by staining with 3,3′-diaminobenzidine (DAB). Cell death was visualized using trypan blue staining as described by Kim and Hwang [89].

## Supplementary information

**Additional file 1. **List of the identified *CBL* genes in pepper.

**Additional file 2. **List of the identified *CIPK* genes in pepper.

**Additional file 3. **Phylogenetic relationship and gene structure of *CBL* (A) and *CIPK* (B) in pepper.

**Additional file 4.** Amino acid sequences of phylogenetic analysis.

**Additional file 5. **The multiple sequence alignment of *CaCBL* and *CaCIPK* families.

**Additional file 6.** MEME sequence of CaCBL.

**Additional file 7.** MEME sequence of CaCIPK.

**Additional file 8. **The Ka/Ks ratios and estimated divergence time for duplicated *CaCBL* and *CaCIPK* genes.

**Additional file 9. **The predicative interaction network of *CBL* and *CIPK* genes in pepper according to the orthologs in *Arabidopsis*.

**Additional file 10. **1500 bp promoter sequences of *CaCBL* and *CaCIPK* genes.

**Additional file 11. **Subcellular localization of CaCIPK1, CaCIPK5, CaCIPK7, CaCIPK15 and CaCIPK20 in N. *benthamiana* epidermal cells.

**Additional file 12. **The multiple sequence alignment of *CaCIPK1, CaCIPK17*, and *CaCIPK19* (A) and expression patterns of *CaCIPK17* and *CaCIPK19* in *CaCIPK1*-silenced plants (B).

**Additional file 13. **The multiple sequence alignment of CBLs from pepper, *Arabidopsis*, cassava, poplar and wheat.

**Additional file 14. **The phylogenetic analysis of *CBL* (A) and *CIPK* (B) gene families from pepper and *Arabidopsis*.

**Additional file 15.** Sequences of the primers used in this study.

## Data Availability

All the data in this research have been contained in the tables, figures, and files from the manuscript and additional files already. The sequences of *Arabidopsis thaliana*, *Manihot esculenta*, *Populus trichocarpa*, *Oryza sativa*, *Triticum aestivum*, and *Brassica napus* were acquired from National Center for Biotechnology Information (NCBI, http://www.ncbi.nlm.nih.gov/), the Plant Genome Resource (https://phytozome.jgi.doe.gov/pz/portal.html) and the TAIR database (https://www.arabidopsis.org/index.jsp).

## Abbreviations

ABA: Abscisic acid
ABR: Abscisic acid repressor
ABRE: Abscisic acid responsiveness element
AKT: *Arabidopsis* K^+^ Transporter1
AREB: ABA resonsive element binding factor
CAMs: Calmodulins
CBLs: Calcineurin B-like proteins
CDPKs: Calcium-dependent protein kinases
CIPKSs: CBL-interacting protein kinases
DAB: 3,3′-diaminobenzidine
ETH: Ethylene
ETI: Effector-triggered immunity
GFP: Green fluorescent protein
H_2_O_2_: Hydrogen peroxide
HMM: Hidden Markov Model
LTR: Low-temperature responsiveness
MAMPs: Microbe-associated molecular patterns
MBS: MYB binding site involved in drought-inducibility
MeJA: Methyl Jasmonate
MSA: Multiple Sequence Alignment
NJ tree: Neighbor-Joining tree
*P. capsici*: *Phytophthora capsic*i
PAMPs: Pathogen-associated molecular patterns
PAT: Protein S-acyl Transferase
PCC: Pearson Correlation Coefficient
PDS: Phytoene desaturase
*p*I: isoelectric points
PKC: Protein kinase catalytic domain
PP2Cs: Type 2C protein phosphatases
PPI: Protein-phosphatase interaction
PTI: Pattern-triggered immunity
qRT-PCR: Quantitative real-time PCR
ROS: Reactive oxygen species
SA: Salicylic acid
SARE: Salicylic acid responsiveness element
SnRK3: SNF1-related serine/threonine kinases
SOS: Salt overly sensitive
SUT: Sucrose transporter
TRV: Tobacco rattle virus
TTC: Triphenyltetrazolium Chloride
TTS: Tonoplast targeting sequences
VIGS: Virus-induced gene silencing
