# Supplementary figures and images for: Identification of CBL and CIPK gene families and functional characterization of CaCIPK1 under Phytophthora capsici in pepper (Capsicum annuum L.)
Source: BMC Genomics. 2019 Oct 25;20:775. doi: 10.1186/s12864-019-6125-z (PMC6814991; doi:10.1186/s12864-019-6125-z)

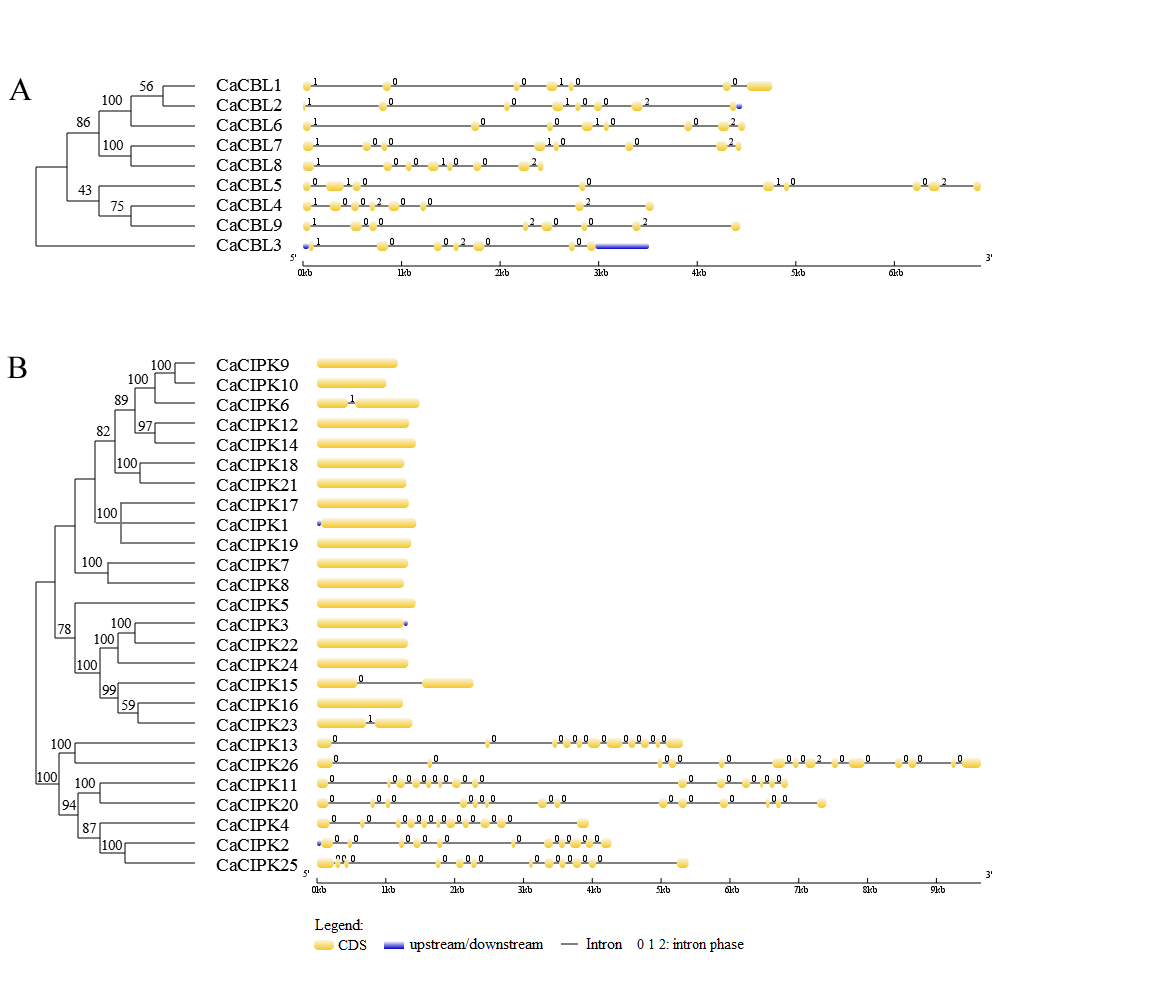

Supplement: Supplementary file 3 — Additional file 3. Phylogenetic relationship and gene structure of CBL (A) and CIPK (B) in pepper. [file 12864_2019_6125_MOESM3_ESM.tif]

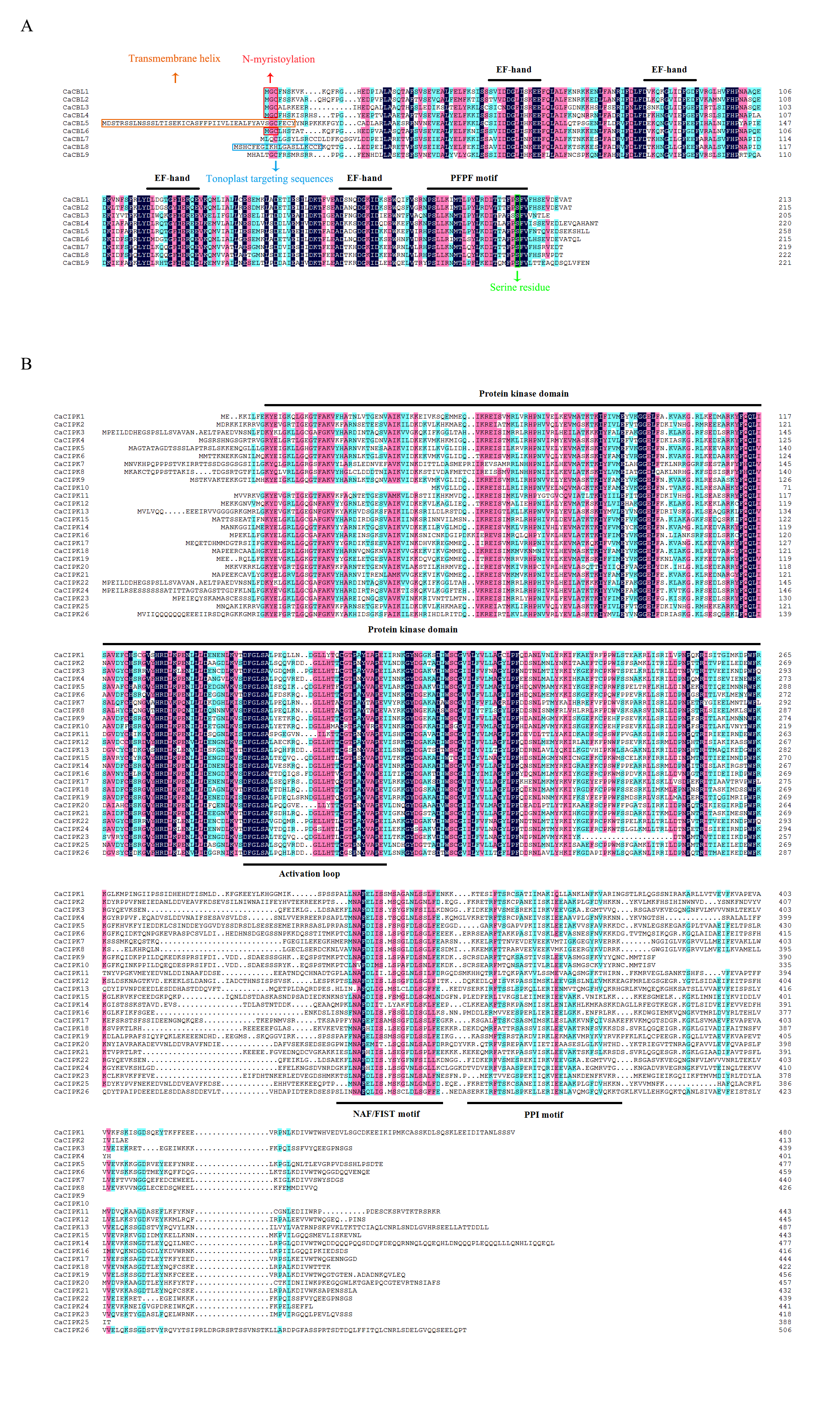

Supplement: Supplementary file 5 — Additional file 5. The multiple sequence alignment of CaCBL and CaCIPK families. [file 12864_2019_6125_MOESM5_ESM.tif]

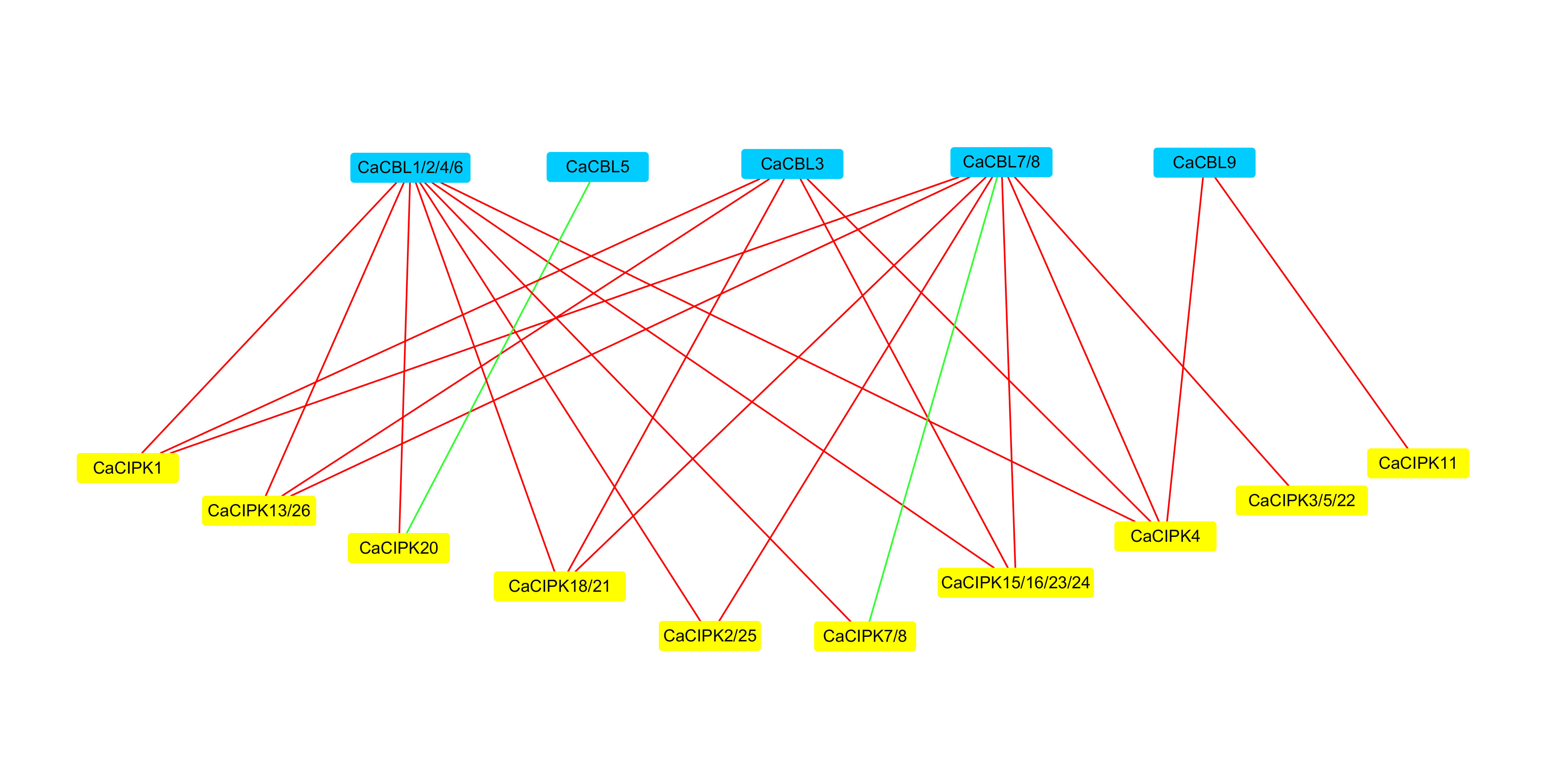

Supplement: Supplementary file 9 — Additional file 9. The predicative interaction network of CBL and CIPK genes in pepper according to the orthologs in Arabidopsis. [file 12864_2019_6125_MOESM9_ESM.tif]

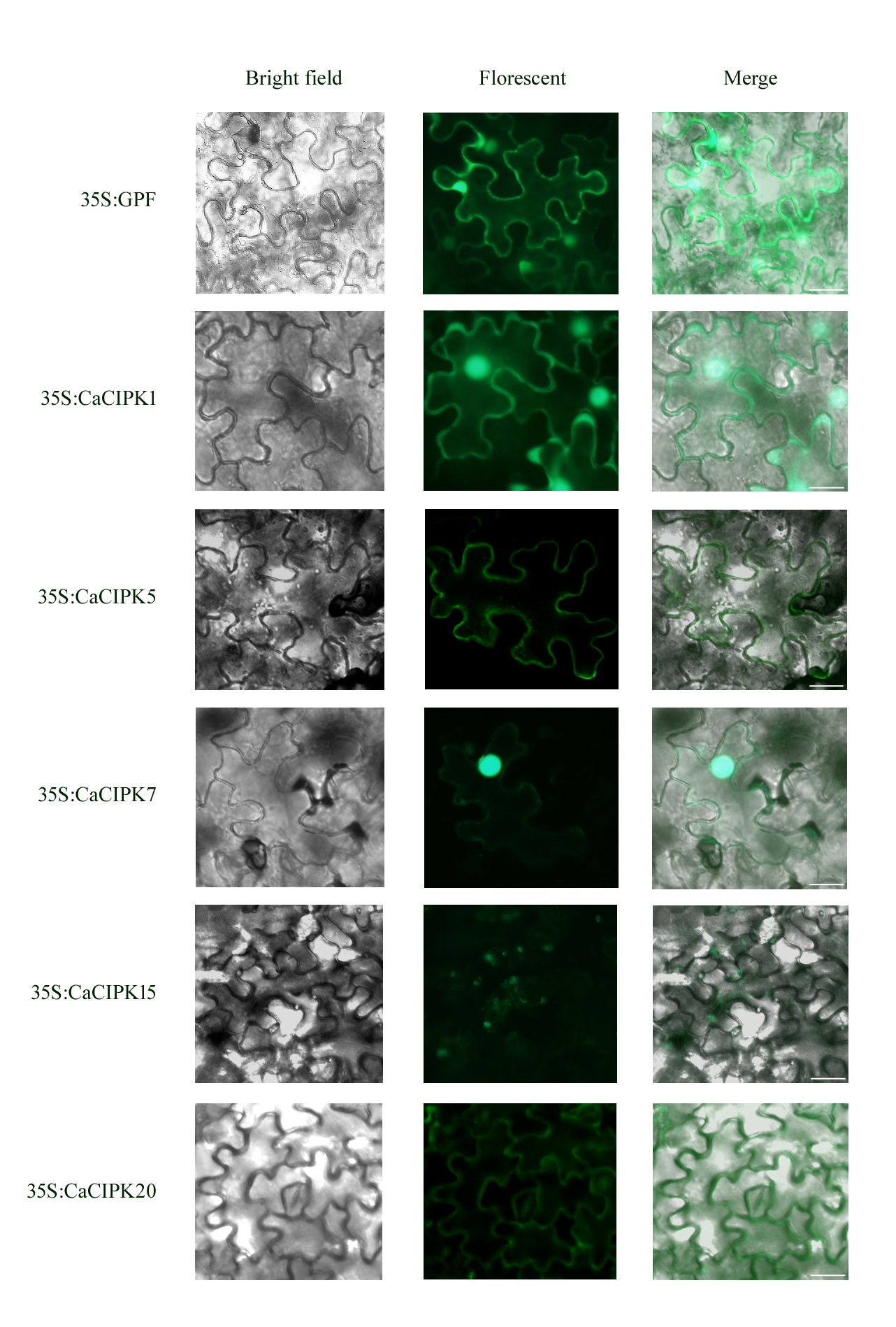

Supplement: Supplementary file 11 — Additional file 11. Subcellular localization of CaCIPK1, CaCIPK5, CaCIPK7, CaCIPK15 and CaCIPK20 in N. benthamiana epidermal cells. [file 12864_2019_6125_MOESM11_ESM.tif]

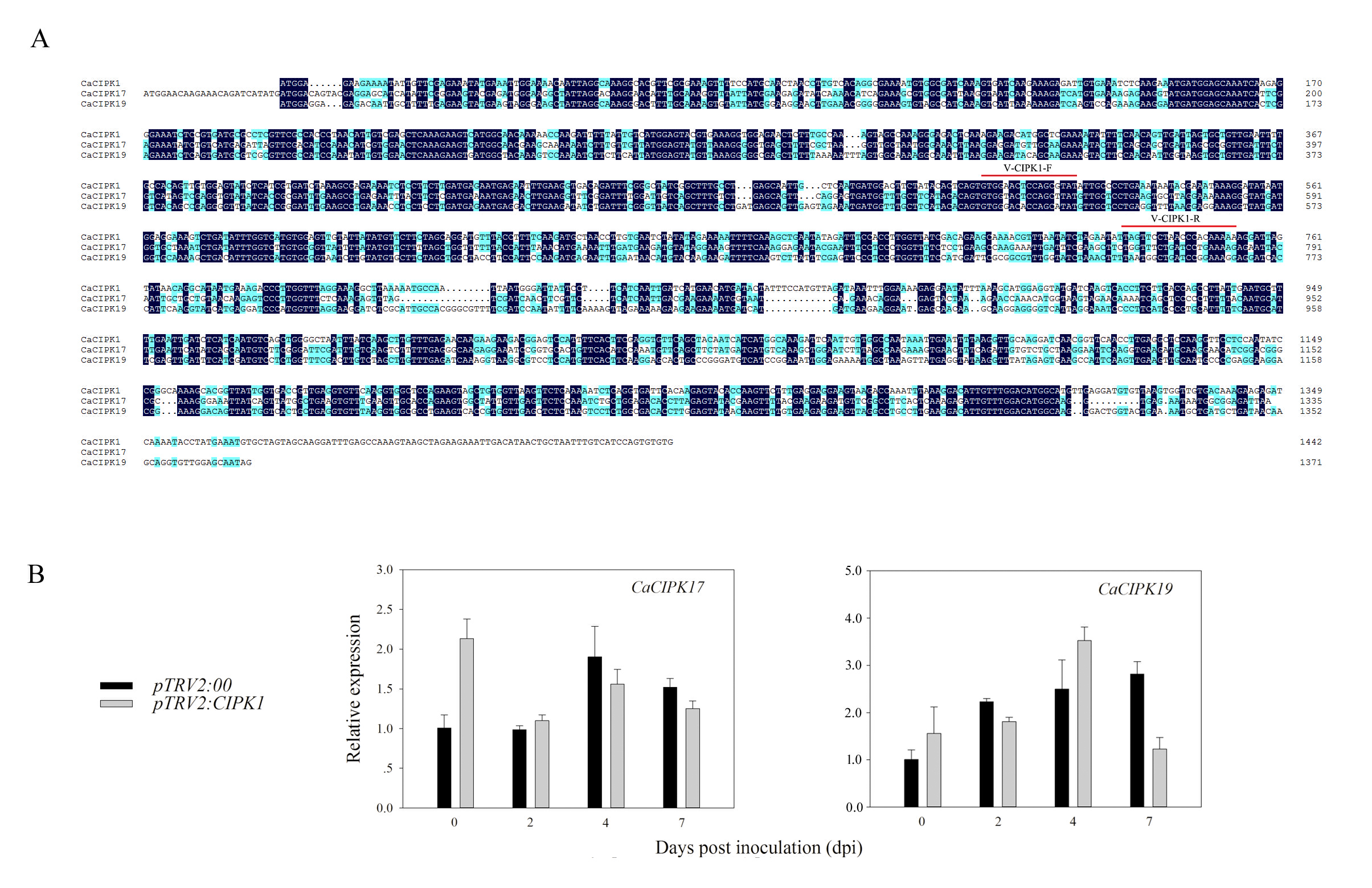

Supplement: Supplementary file 12 — Additional file 12. The multiple sequence alignment of CaCIPK1, CaCIPK17, and CaCIPK19 (A) and expression patterns of CaCIPK17 and CaCIPK19 in CaCIPK1-silenced plants (B). [file 12864_2019_6125_MOESM12_ESM.tif]

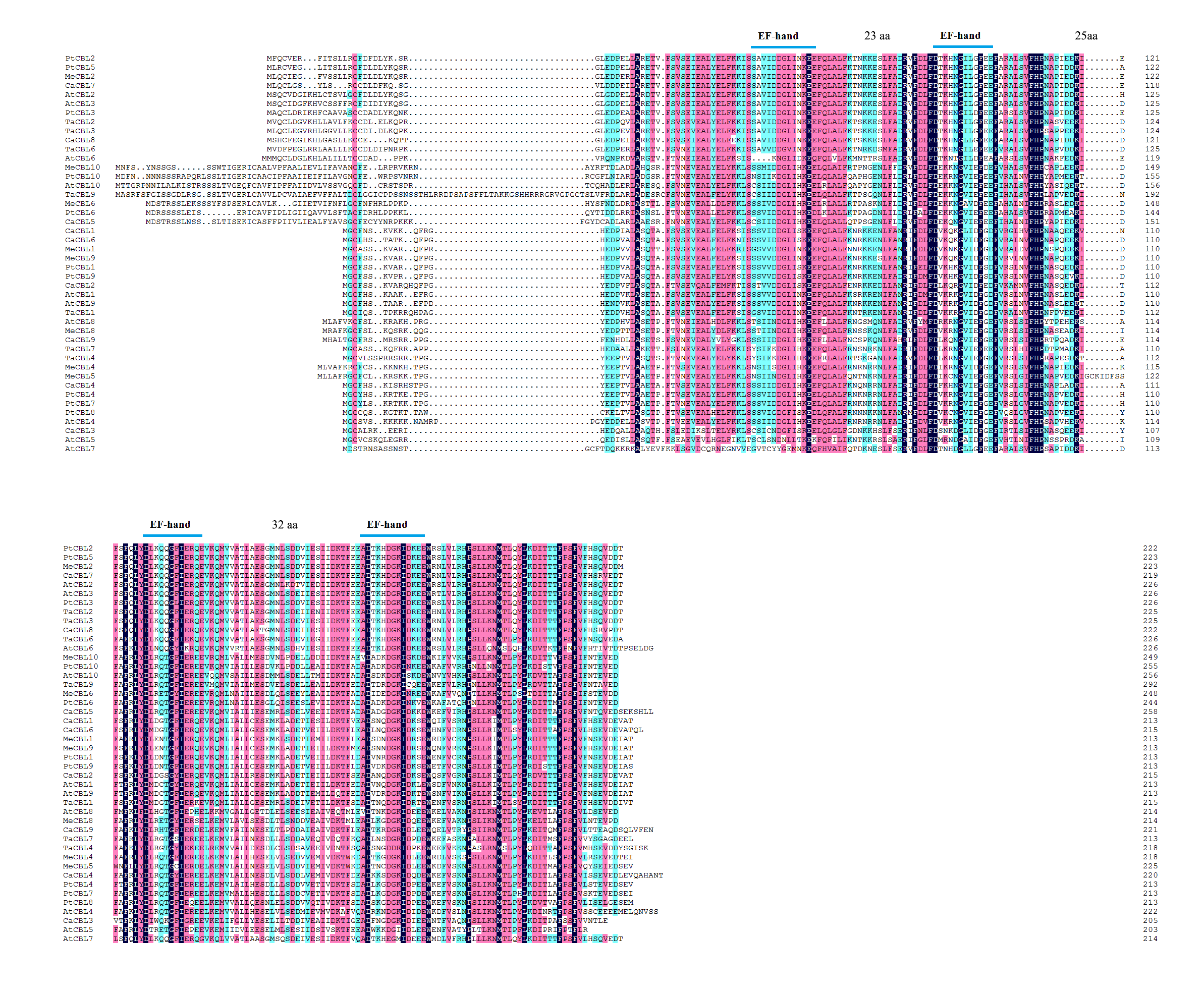

Supplement: Supplementary file 13 — Additional file 13. The multiple sequence alignment of CBLs from pepper, Arabidopsis, cassava, poplar and wheat. [file 12864_2019_6125_MOESM13_ESM.tif]

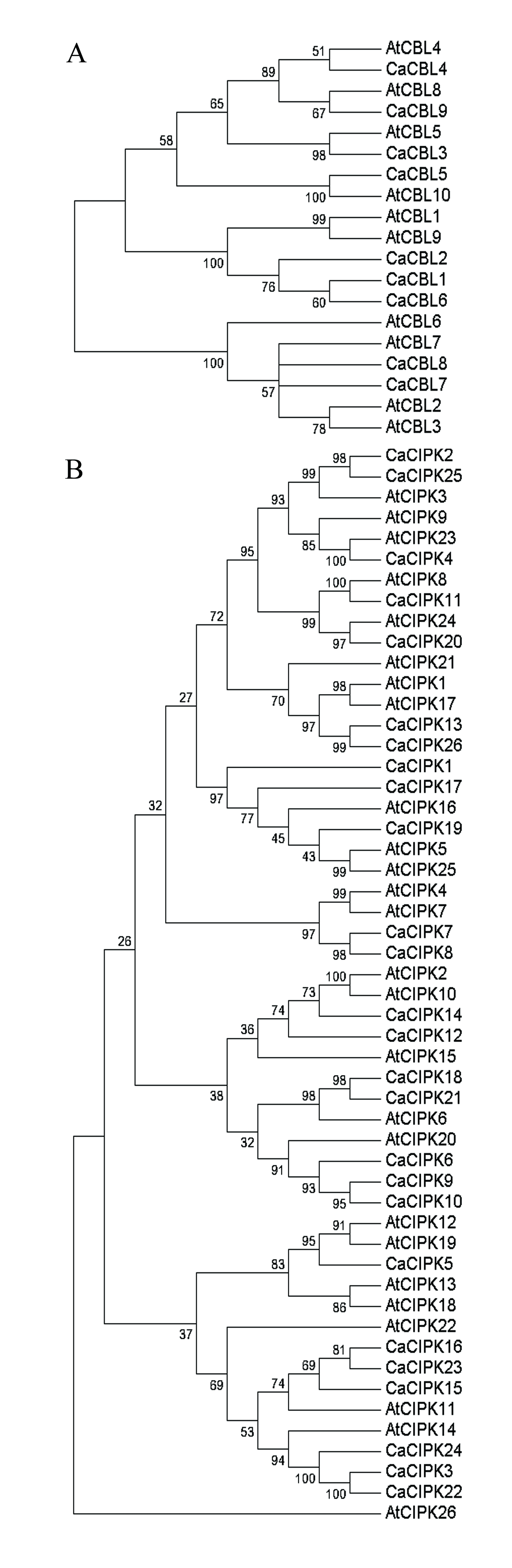

Supplement: Supplementary file 14 — Additional file 14. The phylogenetic analysis of CBL (A) and CIPK (B) gene families from pepper and Arabidopsis. [file 12864_2019_6125_MOESM14_ESM.tif]
