# Supplementary material for: Identification of CBL and CIPK gene families and functional characterization of CaCIPK1 under Phytophthora capsici in pepper (Capsicum annuum L.)
Source: BMC Genomics. 2019 Oct 25;20:775. doi: 10.1186/s12864-019-6125-z (PMC6814991; doi:10.1186/s12864-019-6125-z)
Supplement: Supplementary file 4 — Additional file 4. Amino acid sequences of phylogenetic analysis. [file 12864_2019_6125_MOESM4_ESM.doc]

>CaCBL1

MGCFNSKVKKQFRGHEDPIALASQTAFSVSEVEALFELFKSISSSVIDDGLISKEEFQLALFKNRKKENLFANRIFDLFDVKQKGLIDFGDFVRGLHVFHPNAAQEEKVNFSFRLYDLDGTGFIERQEVKQMLIALLCESEMKLADETIESILDKTFVEADSNQDGKIDKSEWQIFVSRNPSLLKIMTLPYLRDITTTFPSFVFHSEVDEVAT

>CaCBL2

MGCFSSKVARQHQFPGYEDPVFLASQTAFTVSEVQALFEMFKTISSTVVDDGLISKEEFQLALFENRKKEDLLANRIFDLFDLKQRGVIDFEDFVKAMNVFHPNASQEDKLTFSFKLYDLDGSGYIERQEVKQMLIALLRESDMKLADETIEIILDKTFSEADANQDGKIDKSEWQSFVGRNPSLLKIMTLPYLRDVTTTFPSFVFHSEVDEVAT

>CaCBL3

MGCALRKEERIHEDQALLAAQTHFSLEDIKSLTELYRKLSCSICNDGFISREELQLGLFGDNKKHSLFSERIFNLFDSNKDGLIDFGEFIRTLSIFHPNASQEEKIYVTFKLYDIWQKGFIGREEVKELIFGLLYESELILTDDIVEAIIDKTIGEADFNGDGKIDIEEWNTFVAQNPSLLKNMTIPYLKDITPAFSSFVVNTLE

>CaCBL4

MGCFHSKISRHSTPGYEEPTVLAAETAFTVSEVEALYELFKKISSSIIDDGLIHKEEFQLAIFKNQNRRNLFADRIFDLFDFKHNGVIEFGEFVRSLSIFHPNAPLADKIAFAFRLYDLRQTGYIEREELKEMVLALLNESDLVLSDDLVEMIVDKTFDEADKKSDGKIDQDEWKEFVSKNPSLLKNMTLPYLKDITLAFPSFVISSEVEDLEVQAHANTR

>CaCBL5

MDSTRSSLNSSSLTISEKICASFFPIIVLIEALFYAVSGCFECYYNRPKKKFGYDCADLARLAAESRFNVNEVEALYELFKKLSCSIIDDGLIHKEELQLALLQTPSGENLFLDRVFDLFDEKQNGVIEFEEFIHALNIFHPYAPIEDKIDFAFRLYDLRQTGFIEREEVKQMVIAILIESEMRLSDELVEEIIDKTFADADADGDGKIDKKDWKEFVIRHPSLLKNMTLPYLKDITTAFPSFVFNTQVEDSEKSHLL

>CaCBL6

MGCLHSTATKQFPGHEDPVALASQTAFSVSEVEALFELFKNISSSVIDDGLISKEEFQLALFKNRKKENLFANRIFDLFDVKQKGVIDFGDFVRALNVFHPNAPQEEKIDFSFRLYDMDGTGFIERQEVKQMLIALLGESEMKLADETVEIILDKTFLEADLNQDGKIDKSEWHNFVDRNPSLLRIMTLSYLRDITTAFPSFVLHSEVDEVATQL

>CaCBL7

MLQCLGSYLSRCCDLDFKQSGVLDDPEILARETVFSVSEIEALYELFKKISSAVIDDGLINKEEFQLALFKTNKKESLFADRVFDLFDTKHNGILGFEEFARALSVFHPNAPIDDKIEFSFQLYDLKQQGFIERQEVKQMVVATLAESGMNLSDDVIESIIDKTFEEADTKHDGKIDKEEWRNLVLRHPSLLKNMTLQYLKDITTTFPSFVFHSRVEDT

>CaCBL8

MSHCFEGIKHLGASLLKCCEKQTTGLEDPEILARETVFSVSEIEALYELFEKISSAVIDDGLINKEEFQLALFKTSKKESLFADRVFDLFDTKHNGLLGFEEFARALSVFHPNAPIDDKIDFSFQLYDLKQQGFIERQEVKQMVVATLAETGMNLSDEIIESIIDKTFEEADTKHDGKIDKEEWRNLVLRHPSLLKNMTLQYLKDITTTFPSFVFHSRVPDT

>CaCBL9MHALTGCFRSMRSRRPPGFENHDLLASETSFSVNEVDALYVLYGKLSSSIIDDGLIHKEEFLLALFNCSPKQNLFAHRLFDLFDLKQNGVIEFGEFVRSLSIFHPRTPQADKIEFAFKLYDLRHTGFIERDELKEMVFAILNESELTLPDDAIEAIVDKTFLEADTKRDGRIDLEEWQELVTRYPSIIRNMTLPFLKEITQMFPSFVLTTEAQDSQLVFEN

>AtCBL1

MGCFHSKAAKEFRGHEDPVKLASETAFSVSEVEALFELFKSISSSVVDDGLINKEEFQLALFKSRKRENIFANRIFDMFDVKRKGVIDFGDFVRSLNVFHPNASLEDKIDFTFRLYDMDCTGYIERQEVKQMLIALLCESEMKLADETIEIILDKTFEDADVNQDGKIDKLEWSDFVNKNPSLLKIMTLPYLRDITTTFPSFVFHSEVDEIAT

>AtCBL2

MSQCVDGIKHLCTSVLGCFDLDLYKQSGGLGDPELLARDTVFSVSEIEALYELFKKISSAVIDDGLINKEEFQLALFKTNKKESLFADRVFDLFDTKHNGILGFEEFARALSVFHPNAPIDDKIHFSFQLYDLKQQGFIERQEVKQMVVATLAESGMNLKDTVIEDIIDKTFEEADTKHDGKIDKEEWRSLVLRHPSLLKNMTLQYLKDITTTFPSFVFHSQVEDT

>AtCBL3

MSQCIDGFKHVCSSFFRCFDIDIYKQSGGLGDPELLARETVFSVSEIEALYELFKKISSAVIDDGLINKEEFQLALFKTNKKESLFADRVFDLFDTKHNGILGFEEFARALSVFHPNAPIEDKIDFSFQLYDLKQQGFIERQEVKQMVVATLAESGMNLSDEIIESIIDKTFEEADTKHDGRIDKEEWRTLVLRHPSLLKNMTLQYLKDITTTFPSFVFHSQVEDT

>AtCBL4

MGCSVSKKKKKNAMRPPGYEDPELLASVTPFTVEEVEALYELFKKLSSSIIDDGLIHKEEFQLALFRNRNRRNLFADRIFDVFDVKRNGVIEFGEFVRSLGVFHPSAPVHEKVKFAFKLYDLRQTGFIEREELKEMVVALLHESELVLSEDMIEVMVDKAFVQADRKNDGKIDIDEWKDFVSLNPSLIKNMTLPYLKDINRTFPSFVSSCEEEEMELQNVSS

>AtCBL5

MGCVCSKQLEGRRQEDISLLASQTFFSEAEVEVLHGLFIKLTSCLSNDNLLTKEKFQFILIKNTKKRSLSAERIFGLFDMRNDGAIDFGEFVHTLNIFHPNSSPRDKAIFAFRLYDTRETGFIEPEEVKEMIIDVLEESELMLSESIIDSIVSKTFEEADWKKDGIIDLEEWENFVATYPLTLKNMTIPFLKDIPRIFPTFLR

>AtCBL6

MMMQCLDGLKHLALILLTCCDADPPKVRQNPKDVARGTVFTVNEIEALYELFKSISKNGLIDKEQFQLVLFKMNTTRSLFADRVFDLFDTKNTGILDFEAFARSLSVFHPNAKFEDKIEFSFKLYDLNQQGYIKRQEVKQMVVRTLAESGMNLSDHVIESIIDKTFEEADTKLDGKIDKEEWRSLVLRHPSLLQNMSLQHLKDVTKTFPNFVFHTIVTDTPSELDG

>AtCBL7

MDSTRNSASSNSTGCFTDQKKRKALYEVFKKLSGVDCQRNEGNVVEGVTCYYGEMNKEQFHVAIFQTDKNESLFSERVFDLFDTNHDGLLGFEEFARALSVFHPSAPIDDKIDLSFQLYDLKQQGFIERQGVKQLVVATLAASGMSQSDEIVESIIDKTFVQADTKHEGMIDEEEWMDLVFRHPLLLKNMTLQYLKDITTTFPSFVLHSQVEDT

>AtCBL8

MLAFVKCFSLKRAKHPRGYEDPHVLASETPFTVNEIEALHDLFKKLSTSIINDGLIHKEEFLLALFRNGSMQNLFADRVFYMFDRKRNGVIEFGEFVRSLSIFHPYTPEHEKSAFMFKLFDLHGTGFIEPHELKKMVGALLGETDLELSEESIEAIVEQTMLEVDTNKDGKIDEEEWKELVAKNPSILKNMTLPYLKEVTLAFPSFVLDSEVED

>AtCBL9

MGCFHSTAAREFPDHENPVKLASETAFSVSEVEALYELFKSISSSVVDDGLINKEEFQLA LFKNRKKENLFANRIFDLFDVKRKGVIDFGDFVRSLNVFHPNASLEEKTDFTFRLYDMDCTGFIERQEVK QMLIALLCESEMKLADDTIEMILDQTFEDADVDRDGKIDKTEWSNFVIKNPSLLKIMTLPYLRDITTTFPSFVFNSEVDEIAT

>AtCBL10

MTTGRPNNILALKISTRSSSLTVGEQFCAVFIPFFAIIDVLVSSVGQCFDCRSTSPRTCQHADLERLARESQFSVNEVEALYELFKKLSCSIIDDGLIHKEELRLALFQAPYGENLFLDRVFDLFDEKKNGVIEFEEFIHALSVFHPYASIQEKTDFAFRLYDLRQTGFIEREEVQQMVSAILLESDMMLSDELLTMIIDKTFADADSDKDGKISKDEWNVYVHKHPSLLKNMTLPYLKDVTTAFPSFIFNTEVED

>BnaCBL10

MDWTKVSSRSSSLTVGEKVCAVFIPLFAIIDFLFSTVGQCFDCRRRRSPQVCHHVDLARLAHGSPFSVNEVEALYELFKKLSCSIIDDGLIHKEELRLALFQAPYGENLFLDRVFDLFDEKKNGVIEFEEFIHALSVFHPYAPIEEKIDFAFRLYDLRQTGYIEREEVHQMVAAILMESEMILSDELLTMIIDKTFADADVDKDGKISKEEWKVYVLKHPTLLKNMTLPYLKDVTTAFPSFIFNTEVED

>BnaCBL9

MGCLHSMAAREYPGHENPVKLASETAFSVSEVEALYELFKSISSSVVDDGLINKEEFQLALFKNRKKENLFANRIFDLFDVKRKGVIDFGDFVRSLNVFHPNASLEEKTDFTFRLYDMDCTGYIERQEVKQMLIALLCESEMKLADDTIEAILDQTFEDADVDRDGKIGKTEWSDFVIKNPSLLKIMTLPYLRDITTTFPSFVFNSEVDEIAT

>BnaCBL4

MGCSLSKKKKIAIPPPGYEDPDLLASVTPFTAAEVEVLYELFKKLSSSIIEDGLIHKEEFQLALLGNRNRNNLFADRIFDVFDVKRNGVIEFGEFVRSLGVFHPNAPVHEKIKFAFKLYDLRQTGFIEREELKEMVIALLHESELVLSEDMIEVMVDKAFTETDRNNDGKIDVDEWKDLVSMNPSLIKNMTLPYLKDIKATFPSFVLSSEDEELELQNLYF

>BnaCBL3

MSQCVDGFKHVCNSLLRCFDIDIGKSSGGPGDPELLARDTVFSVSEIEALYELFKKISSAVIDDGLINKEEFQLALFKTNKKESLFADRVFDLFDTKHNGILGFEEFARALSVFHPNAPIDDKIDFSFQLYDLKQQGFIERQEVKQMVVATLAESGMNLSDEVIESIIDKTFEEADTKHDGRIDKEEWRILVLRHPSLLKNMTLQYLKDITTTFPSFVFHSQVEDT

>BnaCBL2

MAQCIDGFKHLCTSVLGCFDLDLYKQPGGLGDPELLARDTVFSVSEIEALYELFKKISSAVIDDGLINKEEFQLALFKTNKKESLFADRVFDLFDTKHNGILGFEEFARALSVFHPNVPIDDKIHFSFQLYDLKQQGFIERQELKQMVVVTLAESGMNLKDTVIEDIIDKTFEEADTKHDGKIDKEEWRSLVLRHPSLLKNMTLQYLKDITTTFPSFVFHSQVEDT

>BnaCBL1

MGCFHSKVAREFRGHEDPVKLASETAFSVSEVEALFELFKSISSSVVDDGLINKEEFQLALFKNRKKENLFANRIFDMFDVKRKGVIDFGDFVRSLNVFHPNASLEDKIDFTFRLYDMDCTGFIERQEVKQMLIALLCESEMKLADETIEIILDKTFEDADVNQDGKIDKLEWSDFVNKNPSLLKIMTLPYLRDITTTFPSFIFNSEVDEIAT

>MeCBL1

MGCASSKVARQFPRHEDPVVLASQTTFSVSEVEALFELFKRISGSVVDDGLINKEEFQLALFNNRKKENL

FANRIFDLFDVKNKGVIDFGDFVRALDVFHPNSPQEEKIDFAFRLYDLENTGFIERQEVKQMLIALLCES

EMKLSDETIEMIIDKTFLEADSDNDGKIDRSEWRDFVCKNPSLLRIMTLPYLRDITTTFPSFVFNSEVDE

IAT

>MeCBL2

MLQCIEGFVSSLLRCFDLDLYKQSRGLEDPERLARETVFSVSEIEALYELFKKISSAVIDDGLINKEEFQ

LALFKTNKKESLFADRVFDLFDTKHNGILGFEEFARALSVFHPNAPIEDKIEFSFQLYDLKQQGFIERQE

VKQMVVATLAESGMNLSDDVIESIIDKTFEEADTKHDGKIDKEEWRNLVLRHPSLLKNMTLQYLKDITTT

FPSFVFHSQVDDM

>MeCBL4

MLVAFKRCFCSKKNKHTPGYEEPTVLAAETPFTVSEVEALYELFKKLSNSIISDGLIHKEEFQLALFRNR

NRRNLFADRIFDLFDIKRNGVIDFGEFVRSLSVFHPNAPIEDKIKFAFRLYDLRQTGFIEREELKEMVLA

LLHESELVLSEDVVEMIVDKTWKDADTKGDGKIDLEEWRDLVSKSPSLLKNMTLPYLKDITLSFPSFVLR

SEVEDTEI

>MeCBL5

MLLAFRGCFCLKRSKKTPGYEEPTVLAAETPFTVNEVEALYELFKKLSNSIINDGLIHKEEFQLALFQNT

NKRNLFADRIFDLFDIKCNGVIEFGEFVRSLGIFHPNAPVEDKIGCKIDFSSWNFLLYDLRQTGCIERDE

LKEMVLALLNESELVLSDDVIEMIVDKTWKDADTNCDGKIDLEEWKDFVSKNPSLLKNMTLPYLKDITMA

FPSFVQYSEIEDSEV

>MeCBL6

MDSTRSSLEKSSSYFSPSERLCAVLKGIIETVIFNFLGCFNFHRLPPKPHYSFNDLDRIASTTLFSVNEV

EALLDLFKKLSSSIVDDGLLHREELRLALLRTPASKNLFLDRIFDLFDEKKNGAVDFEEFAHALNIFHPR

ASLEDKIDFAFRLYDLRETGYIEREEVRQMLNAIILESDLQLSEEYLEAIIDKTFADADIDEDGKINREE

WKAFVVQNPTLLKHMTLPSLTDITTAFPSFIFSTEVDD

>MeCBL8

MRAFKGCFSLKQSRKQQGYEDPTTLASETPFTVNEIEALYDLFKKLSSTIINDGLIHKEEFQLALFRNSS

KQNLFADRVFDLFDVKRNGVIEFGDFVRSLSIFHPNASEADKIIFAFRLYDLRETGYIERSELKEMVLAV

LSESDLTLSNDDVEAIVDKTMLEADLKGDGKIDQEEWKEFVAKNPSLIKNMTLPYLKELTLAFPSFVLNT

EVPD

>MeCBL9

MGCFSSKVARQFPGHEDPVVLASQTAFSVSEVEALFELFKSISSSVVDDGLINKEEFQLALFKNRKKESL

FANRIFDLFDVKQKGVIDFGDFVRVLNVFHPNAPQEEKIDFSFRLYDLENTGFIERQEVKQMLIALLCES

EMKLADETIEIILDKTFMEADSNNDGKIDRSEWQNFVRKNPSLLKIMTLPYLRDITTTFPSFVFHSEVDE

IAT

>MeCBL10

MNFSYNSSGSSSWTIGERICAALVPFAALIEVLIFAVANCFELRPRVKRNAYRFTDLADLAHQSRFTVNE

VEALYELYKKLSSSMIDDGLIHKEELQLAIFRTPNGENLFFDRVFDLFDEKKNGVIEFDEFVHALSVFHP

CAPLEEKIDFAFRLYDLRQTGFIEREEVRQMLVALLMESDVNLPDELLDDIIDKTFAEVDADKDGKIGKD

EWKIFVVKHPSILKNMTLPYLKDITTVFPSFIFNTEVED

>OsCBL1

MGCFQSTARRPRPGYEDPVGLASETAFSVSEVEALFELFKSISGSVIDDGLINKEEFRLALFKNKRKENL

FANRIFDLFDVKKRGVIDFGDFVRALNVFHPNIPMEEKIDFSFKLYDMDNTGFIERKEVKQMLIALLGES

EMRLSDEIIETILDKTFSDADTNQDGRIDRTEWENFVSRNPSLLKIMTLPYLKDITTTFPSFVFNSEVDD

LVT

>OsCBL2

MVQCLDGVRQLLAVVFKCCDLELKQPRGLEDPQVLARETVFSVSEVEALYELFKKISSAVIDDGLINKEE

FQLALFKTSKKESLFADRVFDLFDTKHNGILGFDEFARALSVFHPSAPLDEKIDFSFQLYDLKQQGYIER

QEVKQMVVATLAESGMNLSDEIIESIIDKTFEEADTKHDGRIDKEEWRNLVLRHPSLLKNMTLQYLKDIT

TTFPSFVFHSQVDDT

>OsCBL3

MLQCLEGVKQLCGVLLKCCDLDLKQPKGLEDPEILARETVFSVSEVEALYELFKKISSAVIDDGLINKEE

FQLALFKTNKKESLFADRVFDLFDTKHNGILGFEEFARALSVFHPNAPLDEKIDFSFQLYDLKQQGFIER

QEVKQMVVATLAESGMNLSDEVIESIIDKTFEEADTKHDGKIDKEEWRNLVLRHPSLLKNMTLQYLKDIT

TTFPSFVFHSQVDDT

>OsCBL4

MGCASSKQFKRPPGYEEPAVLAAQTTFTVNEVEALRELYNKMSYSIIKDGLIHKEEFQLALFRNSRKANL

FADRVFDLFDLKRNGVIEFGEFVRSLSVFHPKAPKSEKTAFAFKLYDLRGTGYIEKEELREMVLALLDES

DLHLSECAVEAIVDNTFSQADSNGDGRIDPEEWEEFVKANPASLRNMSLPYLQDITMAFPSFVMHSEAHD

>OsCBL5

MMGCLPTKQVGRSTHSLNPREAVALAAETSFTVNEVEALYDLFRKLSNSIIKDGLIHKEEFHLALFRNKK

TNLFVDRVFDLFDQKGNGVIEFDEFVRSLSVFHPDAPEEQKAGFAFKLYDLRQTGFIERHELKEMVLALL

DESDLNITSDAVEMIVDRTFDQADTKGDERIDQEEWNEFVKNNPYVLRNMTLPYLKDLTMVFPSFVIHSE

VSEADMVA

>OsCBL6

MVDSSEGLRRLAALLFKCCSLDSSNRPNGLQDPERLARETVFNVNEIEALYELFKKISSAVVDDGLINKE

EFQLALFKTNRKDSMFADRVFDLFDTKHNGILGFEEFARALSVFHPNAPIDDKIDFAFKLYDLKQQGFIE

KQEVKQMVVATLAESGMNLSDEIIEGIIDKTFEEADTKHDGKIDKEEWRNLVLRHPSLLKNMTLPYLRDI

TTTFPSFVFNSQVEDA

>OsCBL7

MGCISSKQFKRAAEHEDPAILAKETTFSVSEVEALYELFKKISHSIFKDGLIHKEEFQLALFRNSNKKNL

FADRIFDLFDLKRNGVIDFGEFVRSLNIFHPETPLAEKIAFAFRLYDLRGTGYIEREELYEMVLAILNES

DLLLSDDAVEQIVDQTFKQADLNSDGKIDPDEWKAFASKNPALLKNMTLPYLKDITMAFPSFVLNSGVDD

EEL

>OsCBL8

MGCVSSKQFKRAAQHEDPAILAKETTFSVSEVEALFELFKKISHSIFRDGLIHKEEFQLALFRNSNKKNL

FANRIFDLFDLKRNGVIDFGEFVRSLSIFHPETPLGDKIAFAFRLYDLRGTGCIEREELHEMVLALLNES

DLFLSEEAVEQIVDQTFKQADLNDDGKIDPDEWKTFASKNPALLKNMTLPYLKDITMVFPSFILNSEVCE

EEL

>OsCBL9

MESGYGFRFSDDDVESASSLTVGERLCAAFLPFVAIAEAVFFALTDCLADLLPPSAAASRHRRSAASSYL

AAVARKWNHQQRGRVGIGCTSLTLRQLARLADESRCFSVNEVEALFELYEKTSCSIIDDGLTHKEEPQPA

LFRTPSGRNLFLDWVFDLFDEKKNGVIEFDEFIHALSVFHPLAPLEDKINFAFRLYDLRQTGFIEREEVM

QMVIAILSESDMKLSEELLEAIIDKTFEDADADRDGKINQQEWKEFVLRHPNLLKNMTLPYLRDITTVFP

SFVFNTAVED

>OsCBL10

MDSSRSSNSLDSGSSLTLGELACAALIPVLALVDAVVFAAAQCFQKRPPGLLPATLAARARRRAGGRLTF

RELADLADESRCFSVNEVEALYELYKKISCSIVDDGLIHKEELQLALFRTPAGKNLFLDRVFDLFDEKKN

SVIEFEEFIHAISVFHPNTPLEDKIDFSFRLYDLRQTGFIEREEVKQMVVATLLESEVQLSDDLVEAILD

KTFEDADTDKDNRISKEEWKAFVLKHPSVIKKMTLPTLKDTTAAFPSFIFNTQVED

>PtCBL1

MGCFSSKVARQFPGHEDPVALASQTAFSVSEVEALFELYKSISSSVVDDGLISKEEFQLA

LFKNRKKENLFANRIFELFDVKHKGVIDFSDFVRSLNVFHPNASQEDKIDFSFKLYDLDN

TGFIERQEVKQMLIALLCESEMKLADETVEIILDKTFLDADVNRDGKIDKSEWENFVCRN

PSLLKIMTLPYLRDITTTFPSFVFNSEVDEIAT

>PtCBL4

MGCYHSKRTKETPGYEEPTVLAAETPFTVSEVEALYELFKKLSSSVIDDGLIHKEELQLA

LFRNKNRRNLFADRIFDLFDVKRNGVIEFGEFVRSLGVFHPNAPVEDKIHFTFRLYDLRQ

TGFIEREELKEMVLAILHESDLLLSDDVVETIVDKTFSDADLKGDGKIDPEEWKEFVSKN

PSLIKNMTLPYLKDITLAFPSFVLSTEVEDSEV

>PtCBL2

MFQCVERFITSLLRCFDFDLYKSRGLEDPELLARETVFSVSEIEALYELFKKISSAVIDD

GLINKEEFQLALFKTNKKESLFADRVFDLFDTKHNGILGFEEFARALSVFHPNAPIEEKI

EFSFQLYDLKQQGFIERQEVKQMVVATLAESGMNLSDDVIESIIDKTFEEADTKHDGKID

KEEWRSLVLRHPSLLKNMTLQYLKDITTTFPSFVFHSQVDDT

>PtCBL7

MGCYLSKRTKKTPGYEEPTVLAAETPFTVNEVEALYELFKKLSSSVIDDGLIHKEELQLA

LFRNKNKRNLFADRIFDLFDVKRNGVIEFGEFVRSLGVFHPNAPVEDKIHFAFRLYDLRQ

TGFIEREELKEMVMALLHESDLLLSDDCVETIVDKTFSDADLKGDGKIDPDEWKEFVSKN

PSLIKNMTLPHLKDITLAFPSFVSKTEVEDSEI

>PtCBL3

MAQCLDRIKHFCAAVASCCDADLYKQNKGLEDPEALARETVFSVSEIEALYELFKKISSA

VIDDGLINKEEFQLALFKTNKKDSLFADRVFDLFDTKHNGILDFEEFARSLSVFHPNAPI

DDKIEFSFQLYDLKQQGLIERQEVKQMVVATLAESGMNLSDDVIESIIDKTFEEADTKHD

GKIDKEEWRSLVLRHPSLLKNMTLQYLKDITTTFPSFVFHSQVDDT

>PtCBL9

MGCFSSKVPRQFPGHEDPVALASQTAFSVSEVEALFELYKSISSSVVDDGLISKEEFQLA

LFKNRKKENLFADRIFDMFDVKHKGVIDFSDFVRSLNVFHPNASQEVKIDFSFKLYDLDN

TGFIERQEVKQMLIALLCESEMKLADETVEIILDKTFLDADVDKDGKIDKSEWETFVCRN

PSLLKIMTLPYLRDISTTFPSFVFNSEVDEIAS

>PtCBL5

MLRCVEGLITSLLRCFDLDLYKQSRGLEDPELLARETVFSVSEIEALYELFKKISSAVID

DGLINKEEFQLALFKTNKKESLFADRVFDLFDTKHNGILGFEEFARALSVFHPNAPIDDK

IAFSFQLYDLKQQGFIERQEVKQMVVATLAESGMNLSDDVIESIIDKTFEEADTKHDGKI

DKEEWRSLVLRHPSLLKNMTLQYLKDITTTFPSFVFHSQVDDT

>PtCBL10

MDFNNNNSSSRAPQRLSSLTIGERICAACIPFAAIIEIFILAVGNCFEWRPSVNRNRCGF

LNIARLADGSRFTVNEVEALYELYKKLSNSIIKDGLIHKEELQLALFQAPHGENLFLDRL

FDLFDEKRNGVIEFEEFVRALNVFHPYAPMEEKTDFAFRLYDLRQTGFIEREEVKQMVIA

ILLESDVKLPDDLLEAIIDKTFADADADKDGKINKEEWKAFVVRHPNLLNNMTLPYLKDI

STVFPSFIFNTEVED

>PtCBL6

MDRSSSSLEISERICAVFIPLIGIIQAVVLSFTACFDRHLPPKKLQYTIDDLRRIASNSL

FTVNEVEALLELFKKLSSSVIDDGLIHKEDLKLALLKTPAGDNLILDRLFALFDEKKNGV

IEFEEFAHALSVFHPRAPMEAKIDFAFRLYDLRQTGFIEREEVRQMLNAILLESGLQISE

ESLEVIIDKTFADADADKDGKINKVEWKAFATQHPNLLKNMTLPYLRDITTMFPSFIFNT

EVED

>PtCBL8

MGCCQSKGTKTTAWCKELTVLASGTPFTVSEVEALHELFKKLSSSVIGDGFISKEDLQFA

LFRNNNKKNLFANRMFDLFDVKCNGVIEFGEFVQSLGVFHPNAPVEEKIYFAFRLYDLRQ

TGFIEQEELKEMVVALLQESNLELSDDVVQTIVDKTFSDADSKGDGKIDPEEWKEFVSKN

PSIIKNMTLPYLKDVTVAFPSFVLISELGESEM

>TaCBL2

MVQCLDGVKHLLAVLFKCCDLELKQPRGLEDPQVLARETVFSVSEVEALYELFKKISSAVIDDGLINKEE

FQLALFKTSKKESLFADRVFDLFDTKHNGILGFEEFARALSVFHPNASVEEKIDFSFQLYDLKQQGFIER

QEVKQMVVATLAESGMNLSDEIIENIIDKTFEEADTKHDGKIDREEWHNLVLRHPSLLKNMTLQYLKDIT

TTFPSFVFHSQVDDT

>TaCBL7

MGCASSKQFRRAPPHEDAALLAKETTFSLNEVEALYELFKKISYSIFKDGLIHKEEFQLALFRNSNRKNL

FADRIFDLFDLKRNGVIEFEEFVRSLHIFHPDTPMADKIAFAFRLYDLRGTGSIEREELKEMVLAILNES

DLLLSDDAVEQIVDQTFKQADLNSDGRIDPDEWKEFASKNPALLKNMTLPYLKDITMSFPSFVVYSGAGD

EEL

>TaCBL1

MGCIQSTPKRRQHPAGYEDPVHLASQTAFSVSEVEALFELFKSISGSVIDDGLINKEEFQLALFKNTRKE

NLFANRIFDLFDVKKRGVIDFGDFVRALNVFHPNFPVEEKIDFSFKLYDMDGTGFIERKEVKQMLIALLG

ESEMRLSDEIVETILDKTFSDADTNQDGKIDRTEWENFVSRNPSLLKIMTLSYLKDITTTFPSFVFHSEV

DDIVT

>TaCBL6

MVDFPEGLRRLAALLLKCCDLDIPNRPKGLEDPERLARETVFSVNEIEALYELFKKISSAVVDDGVINKE

EFQLALFKTNRKDSMFADRVFDLFDTKHNGILEFEEFVRALSVFHPNAPVDDKIDFAFKLYDLKQQGFIE

KQEVKQMVVATLAESGMNLSDEVIEGIIDKTFEEADTKHDGKIDKEEWRNLVLRHPSLLKNMTLPYLRDI

TTTFPSFVFNSQVEDA

>TaCBL3

MLQCLEGVRHLGGVLLKCCDIDLKQPKGLEDPEVLARETVFSVSEVEALYELFKKISSAVIDDGLINKEE

FQLALFKTSKKESLFADRVFDLFDTKHNGILGFEEFARALSVFHPSAPPEEKIDFSFQLYDLKQQGFIER

QEVKQMVVATLAESGMNLSDEVIESIIDKTFEEADTKHDGKIDKEEWRNLVLRHPSLLKNMTLQYLKDIT

TTFPSFVFHSQVDDT

>TaCBL9

MASRFSFGISSGDLRSGSSLTVGERLCAVVLPCVAIAEFVFFALTDCLGGICPPSSNSSTHLRRDPSAPS

FFLTAKKGSHHRRRGRVGPGCTSLVFRDLARLADESRCFSVNEVEALFELYKKISCSIIDDGLIHKEELQ

LALFKTPSGQNLFLDRVFDLFDEKKNGVIEFEEFIHALSVFHPLAPVEDKINFAFRLYDLRQTGFIEREE

VMQMVIAILMESDVELSDELLEAILDKTFEDADTDRDGKICQEEWKEFVLRHPNLLKNMTLPYLRDVTTA

FPSFVFNTAVED

>TaCBL4

MGCVLSSPRRSRRTPGYEEPTVLASQTSFTVNEVEALYELYKKLSYSIFKDGLIHKEEFRLALFRTSKGA

NLFADRVFDLFDLKRNGVIEFGEFVRSLSIFHPRAPESDKTAFAFKLYDLRGTGYIEKEELREMVVALLD

ESDLCLSDSAVEEIVDNTFSQADSNGDDRIDPKEWEEFVKKNPASLRNMSLPYLQDITTAFPSFVMHSEV

DDYSGISK

>CaCIPK1

MEKKILFEKYEIGKQLGKGTFAKVFHATNLVTGENVAIKVIKKEIVKSQEMMEQIKREISVMRLVRHPNIVELKEVMATKTKIFIVMEYVKGGELFAKVAKGRLKEDMARKYFQQLISAVEFCHSCGVSHRDLKPENLLLDENENLKVTDFGLSALPEQLLNDGLLYTQCGTPAYIAPEIIRNKGYNGGKSDIWSCGVVLYVLLAGCLPFQDANLVNLYRKIFKAEYRFPPWLSTEAKRLISRILVPNPQKRISITGIMKDPWFRKGLKMPINGIIPSSIDHEHDTISMLDKFGKEEYLKHGGMIKSPSSPALLNAFELISSMSAGANLSSLFENKKKTESIFTSRCSATIIMAKIQLLANKLNFKVARINGSTLRLQGSSNIRAKARLLVTVEVFKVAPEVAVVKFSKISGDSQEYTKFFEEEVRPNLKDIVWTWHVEDVLSGCDKEEIKIPMKCASSKDLSQSKLEEIDITANLSSSV

>CaCIPK2

MDRKKIKRRVGKYEVGRTIGEGTFAKVKFARNSETEESVAIKILDKDKVLKHKMAEQIKREIATMKLIRHPHVVQLYEVMGSKTKIFIVLEFVTGGELFDKIVNHGRMREEEARKYFQQLINAVDYCHSRGVYHRDLKPENLLLDAAGDLKVSDFGLSALSQQVRDDGLLHTTCGTPNYVAPEVLNDRGYDGATADLWSCGVILFVLLAGYLPFDDSNLMNLYNKITAAEFTCPPWISFSAMKLITRILDPNPTTRITVPEILEDEWFKKDYRPPVFNEIEDANLDDVEAVFKDSEVSILNIWNAIIFEYHVTEKREEKPTSMNAFELISMSQGLNLGNLFDEQGFKRETRFTSKCPANEIISKIEEAAKPLGFDVHKKNYKVLMKFHSIHINWNVDYSNKFNDVYVIVILAE

>CaCIPK3

MPEILDDHEGSPSLLSVAVANAELTPAEDVNSNLFDKYKLGKLLGCGAFGKVYHARDINTAQSVAIKVVGKQKIFKGGLTAHVKREISIMRRLRHPHIVRLHEILATKKKIYFALEFAKGGELFSKLAKGRFSEDLSRRYFQQLISAVGYCHSRRVYHRDLKLENLLLDENCDLKVTDFGLSAVGDQMRPGELLHTLCGTPAYVAPEILAKKGYDGAKVDIWSCGIILFVFNAGYLPFNDTNLMTIYRKIYKGEFRCPKWTSPELKRLLTRLLDTNPVTRITVEEIKNDPWFQRGYQEVKSENQFEIKLGLDSAEKFLNAFDIISYSYGFNFSILLKDNGGFIDKERFVSMESREKIIRKVEEVGKAEGMTVVQRSGASVKVEGQNGNFVLMVVVNRLTEKLVIVEIEKRETEGEIWKKKFKPQISSFVYQEEGPNSGS

>CaCIPK4

MGSRSHNGSGRTRVGRYELGRTLGEGTFAKVKFARNVETGDNVAIKILDKEKVMKHKMIGQIKREISTMKLIRHPNVIRMYEVMASKSKIYIVLEFVTGGELFDKIASKGRLKEDEARKYFQQLINAVDYCHSRGVFHRDLKPENLLLDANGVLKVSDFGLSALPQQVREDGLLHTTCGTPNYVAPEVINNKGYDGAKADLWSCGVILFVLMAGYLPFEESNLMALYKKIHKAEFTCPPWFSSNAKKLIKRILDPNPQMRITISEVIENEWFKKGYRPPVFEQADVSLDDVNAIFSESAVSVLDSSNLVVERREERPSAPLTMNAFELISTSQGLNLSSLFEKQMGLVKRETRFTSRCPANEIVSKIEEAAVPLGFNVRKNNYKVNGTSHSRALALIFFYH

>CaCIPK5

MAGTATAGDTSSSLAPTRSLSKKENQGLLLGRYEIGKLLGHGTFAKVYHARNVKTNESAAIKVIDKEKILKVGLIDHIKREISILKRVRHPNIVELYEVMATKSKIYFVMEFVKGGELFNKVAKGRLKEEVARKYFQQLVSAVAFCHARGVYHRDLKPENILLDEDGNVKVSDFGLSAISEQIKQDGLFHTFCGTPAYVAPEVLARKGYDAAKVDIWSCGVILFVLMAGYLPFHDQNVMAMYKKIYKGEFRCPRWFSPELTRFLKHLLDINPEKRITIQEIMNNRWFKKGFKHVKFYIEDDKLCSINDDEYGGVDYSSDRSDLSESESEMEIRRRSASLPRPASLNAFDIISFSRGFDLSGLFEEGGDGARFVSGAPVPKIISKLEEIAKVVSFAVRKKDCKVNLEGSKEGAKGPLTVAAEIFELTPSLRVVEVKKKGGDRVEYEEFYNRELKPGLQNLTLEVGRPVDSSHLPSDTE

>CaCIPK6

MMTTKNEKKGNILMQKYEIGKLLGQGTFAKVYHARNLKTGLSVAIKVIDKEKVMKVGLIDQTKREISVMRLIKHPNVVQLYEVMASKSKIYFAMEYVRGGELFNKVAKGRLKEDAARKYFQQLIAAVDFCHSRQVYHRDLKPENLLLDEGGNLKVTDFGLSALFDSKRQDGLLHTTCGTPAYVAPEVINKRGYDGEKADIWSCGVVLFVLLAGYLPFHDQNLMEMYKKISKGEFKCPQWFPPEVRKLLSRILDPNPGSRITLVKLMENYWFKKGFKQIDKTQNPGKEVRASPCSVLDIHEDHNSDGEGSSNPKKDQSSTITMKPTCLNAFDIISLSPGFDLSGLFEEEKERRSEARFTAKKPASIIVSKLEEVASNESFNVKKKDGTVTMQSIKQGRKGQLAIDAEIFEITPSFHVVEVSKKSGDTMEYKQFFDQGLKTSLKDIVWTWQGGDQQVENQE

>CaCIPK7

MNVKHPQPPPSTVKIRRTTSSDGSGSGSIILGKYQLGRLLGRGSFAKVYLARSLEDNVEFAVKVINKDITTLDASMEPRIIREVSAMRRLNHHPNILKLHEVMATKTKIYFVMELAHGGELFTKLNRRGGRFSESTARFYFHQLVSALQFCHQNGVAHRDVKPQNLLLDKDGHLKISDFGLSALPEQLRNGLLHTACGTPAYTAPEVVYRKGYDGAKADAWSCGVILFVFLAGRLPFDDSNLPTMYKAIHRREFVFPDWVSKPARRIISRLLDPNPETRYGIEELMNTLWFLKSSSMKQEQSTKQFGEGILEKEGKHMERMNAFDLISMSSGLDLSGLFEARSNKKELRFTTNVEVDEVEEKVMTIGKGEGYRVERRKNGGIGLVKGRVVLLMEIFEVAKLLWLVEFTVVNGGQEFEDCEWEELKIGLKDIVVSWYSDGS

>CaCIPK8

MKAKCTQPPSTTAKISTDGSRTGTFILGKYQLVRLLGRGSFAKVYHGLCLDDDTNIAIKVIDKKSTIVDAFMETCIIREISSMVRLNHHPNIIKLKEVLATKSKIYLVMEIATGGDLQAKLNRHGKFSDSISRFYFHQLVSALHYCHQNGVTHRDIKPQNILLDQNNNVKVSDFGLSALPEQLKNGLLHTACGTPAYTAPEVAYRKGYNGEKADSWSCGVILYTFLSGYLPFDDSNISNMFRLVHLRRLRFPDWVSKSARSVINRLLDPNPSTRLSIEELMKLSWFKKSELKHRQLNLGECLSERDCKNLVAVNAFDIISMSSGLDLSGLFESDMIKKEMKFTTRARVEEVEEKVMKIGEERGYKVDRRKGWGIGLVKGRVVLMVEILKVAMELLLVEVKVVNGGLECEDSQWEELKFEMMDIVVQ

>CaCIPK9

MSTKVAKTEKKGTILMHKYEIGKLLGQGTFAKVYHARNLKTSQNVAVKVIDKEKVMKVGLIDQIKREISVMRLIRHPNVVELYEVMASKTKIYFAMEYVKGGELFNKVAKGRLRESAASKYFQQLIAAVDFCHSRGVYHRDLKPENILLDETGNLKVSDFGLSALYETKRQDGLLHTTCGTPAYVAPEIINKRGYDGEKADIWSCGVILFVLLAGYLPFHDANLMGMYKKISKGEFKCPEHFPSEVKKLLSRILDPNPFSRITLAKLMNNNWFKKGFKQIDKPPILDQKEDKSPRSIFDIVDDSDAESSSGHKEQSPSTMKPTCLNAFDIISLSPAFDLSNLFEKDKSCRSDARFTTQKSASTIVSRLEEVASMGSFKVYYQNCMMTISF

>CaCIPK10

MKVGLLDQIKREISLLRLIRHPNVVELNQVMAGKTKIYFAMEYVKGGELFNTVAKGRLRESAAHKYFQQLIAAVDFFHSRGVYHRDLKPENILLDETGNLKVSDFGLSALYETKRQDGLLHMACRTPAYVRPEVINKRGYDGKKADIWSGGVILFVMLAGYLPFHDANLMVMYKKISKGEFKCPEHFPSEVKKLLSRILDPNPFSRITLAKLVNYTWFKKGFKQINKPPILDQEQDESPRSIFDIVDDSDAESSSRYKEQSPSTMKPTCLNVFDIMSLSPAFDLSNLFEKDKSCRSEARFMTQNSASTIVLRLEEVASMGSCKVYYRNCMMTISV

>CaCIPK11

MVVRKVGKYEVGRTIGEGTFAKVKFAQNTETGESVAMKVLDRSTIIKHKMVDQIKREISIMKLVRHPYGTGVCQVIATLTKIYIILEFITGGELFDKIVHHGRLSEAESRRYFQQLIDGVDYCHIKGVYHRDLKPENLLLDSQGNLKISDFGLSASPGEGVNILKTTCGTPNYVAPEVLSHKGYDGAVADIWSCGVILYVLMAGFLPFDEVDLTTLYAKIDKADFSCPSWFPVGAKSLIHRILDPNPQTRIRIEEIRNDEWFKTNYVPGKVMEYEDVNLDDINAAFDDSEEEATNDQCHNADTGPLALNAFDLIILSQGLNLSILFDRGQDSMKHHQTRFLTQKPAKVVLSSMEVAAQSMGFKTHIRNFKMRVEGLSANKTSHFSVFEVAPTFFMVDVQKAAGDASEFLKFYKNFCGNLEDIIWRPPDESCKSRVTKTRSRKR

>CaCIPK12

MEKKGNVVMQKYDVGRLLGQGNFAKVYYGRNLETGESVAIKVIDKEKVLKAGLIEQTKREISVMALIEHPNILKLYEVMATKSKIYLVIEHAKGGELFKKLAKGRLKEKLARKCFQQLISAVDCCHSRDVYHRDLKPENVLLDENGNLKVSDFGLSALAECKRQDGLLHTTCGTPAYVAPEVISRKGYDGAKADIWSCGVILFVLLAGFLPFQDSNLMEMYRKIRLAEFKFPNWFPSEVRKLISRMLDPNPHTRISIAKIKASSWFKKSLDSKNAGTKVDEKEKLSLDANGIIADCTHNSISPSVSKLESPKPTSLNAFDIISLSSGFDLSGLFITKDQKEDLQFISVKPASSIMSKLEEVGRSLNLEVMKKEAGFMRLEGSGEGRYGTLSIDAEIFEITPSFHLVELKKSYGDKVEYKKMLRQFIRPALEEVVWTWQGEQPINS

>CaCIPK13

MVLVQQEEEIRVVGGGGRKGMRLGKYEVGKTLGEGNFGKVKYAKHVDSGKSFAIKILDKSRILDLRSTDQIKREIGTLKLLKHPNVVRLYEVLASKSKIYMVLEYVNGGELFDRIVSKGKLSEAQGRKLFQQLVDGVCYCHDKGVSHRDLKLENVLIDSKGNIKITDFGLSALPQHFRDDGLLHTTCGSPNYVAPEILSNRGYDGATSDTWSCGVILYVILTGYLPFDDRNLAVLYQKILKGDVHIPKWLSAGAKNLIKRILDPNPHTRITMAQIKEDGWFKQDYIPVNPDEEDLESDDHASTDDQVFTMQETPLDAQRDPESHLINAFQLIGMSLCLDLSGFFEKEDVSERKIRFTSSLSPKQLLERIENMVTQMGFHVQKRHGRLKVMQEQKGHKSATSLLVVAEVFEISPSLYVVELQKSSGDSTVYRQVYLKNILVYLVATRNPSKPVKLTKTTCIAQLCNRLSNDLGVHRSEELLATTDDLL

>CaCIPK14

MANKGGILMERYELGRLLGQGTFAKVYYARNIKTGQSVAIKVVDKEKILRVGLMDQIKREISVMKLVRHPNIVHLYEVMATKTKIYFVMEYCKGGELFNKVAMGRLKEDVARKYFQQLINAVDFCHSRGVYHRDLKPENLLLDDNENLKISDFGLSALVESKRQDGLLHTTCGTPAYVAPEVINRKGYDGAKADIWSCGVVLFVLLAGYLPFQNSNLMEMYRKIGRAEFKCPSWFPPEARRLLSRMLDPNPITRISLAKIRGSTWFRKGISTSSKSTAVDEVSTDLASTNTDDKQEAAQMPKLNAFDIITLYAKFDLSKLFEEPCLKKEAKFTSRKPASMIISKLENIAKHLKMKASKKDAGLLRFEGTKEGKKGTLSIDVEIFEVVEDFHLVEVKKSNGDTLEYQQILNECLRPGLQDIVWTWQDDQQQPQQSDDQFDEQQRNNQLQQEQHLDNQQQPLEQQQLLLQNHLIQQEQLP

>CaCIPK15

MATTSSEATIFNKYELGKLLGCGAFGKVYHARDIRDGRSVAIKIINKSRINNVILMSNIKREISIMRRLRHPHIVKLDEVLATKTKIYFVMEFVKGGELFAKIAKAGKFSEDQSRKIFQQLISAVRYCHYRGVYHRDLKPENLLIDENGDLKVSDFGLSALTEQVQQDGLLHTLCGTPSYVAPDVLTKKGYDGAKADIWTCGIILYVLNAGYLPFHDSNLMGMYNKICNGEFKCPKWMSCELKRFIRRLLDINPMTRITIEEIMNDPWFKKGLKRVKFCEEDGKPQKPIQNLDSTDASKASNDPSADIEDKNKNSYLNAFDIISFSMGLDLSGMLNDGFNPLEDFERLIVKGSLEIIMERIEEVAKKVNIKLKKKKESGMELKKGKLIMNIEIYKVIDDLVVVEVRRKVGDIDMYKELLKNNMKPVILGQQSMEVLISKEVNL

>CaCIPK16

MPEKLLFGKYELGKLLGCGAFAKVYHAREINSGKSVAIKVINKSNICNKDGIPDKKIEREVSIMRQLQHPYIVKLHEVLATKTKIYFIMEYVKGGELFNLIANKSRFSEDLSRKCFQQLISAVNYCHLRGVYHRDLKPENILIDENGDIKVSDFGLSATTDQIQSFDGLLHTVCGTPAYVAPEILTIKGYDGAKTDIWSCGIILYIMIAGYFPFYDQNLMLMYKKIYKGEFRCPKWMSPDVKRILSRLLDVNPGTRITIDEIIRDPWFRKGLKFIKFSGEEENKDSLISNSFNAFDLISFSSGLDISGLLKSNNPMDDLERMVVEESPERLIERIEELGKKENFRLRKKKDWGIEMKVQNGKVTMRLDVYRLTEHLVIMEVQKNDGDGDLYKDVWRNKLKPIILGQQIPKIEDSDS

>CaCIPK17

MEQETDHMMDGTRSIIFGKYEMGRLLGQGTFAKVYYGRDIKTSESVAIKVINKDHVKREGMMEQIIREISVMRLVRHPNIVELKEVMATKQKIFVVMEYVKGGELFAKVANGKLKEDVARKYFQQLISAVDFCHSRGVYHRDLKPENLLLDENENLKVSDFGLSALSEQFRSDGLLHTQCGTPAYVAPEVLRKKGYDGAKSDIWSCGVILYVLLAGFLPFKHENLMKMYRKVFKGEYEFPPWFSPEAKKLISKLLVSDPEKRITIAAVTRVPWFLKEFSRSTSFSSIDEENGNQKQESTKEPNMVSRTKSAPPFYNAFEFISAMSSGFDLSSLFEGKRKSGALFTSKCSASMIMSKLESLAKKVNFQIVSAKEFKVKMQGTSDGRKGKLSVMAEVFEVAPEVAIVEFSKSAGDTLEYTKFYEEDVRPSLKEIVWTWQGENNGGD

>CaCIPK18

MAPEERCAALHGKYELGRLLGHGTFAKVYHARNVQNGKNVAIKVVGKEKVIKVGMMEQIKREISIMKMVKNMNIVELHEVMASKTKIYFAMEYVKGGELFAKIAKGRLREDVARGYFQQLISAIDFCHSRGVYHRDLKPENLLLDDAGNLKVTDFGLSAFTDHLRQDGLLHTTCGTPAYVAPEVIGKKGYDGSKADIWSCGVILYVLLAGFLPFQDENIMAMYKKIYRGDFKCPPWFSSESRKLIMKMLEPNPNSRITASKIMDSSWFKKSVPKTLRHREEEEEFGLASEKVKEVETMNAFHIISLSEGFDLSPLFEEKKRDEKDQMRFATTRSASSVISKLEEVAKTRNFSVKKSDSSVRLQGQEIGRKGKLGIVADIFAITNSFVVVEVNKASGDTLEYNQFCSKELRPALKDIVWTTTK

>CaCIPK19

MEERQLLFEKYEVGKLLGKGTFAKVYYGKELETGESVAIKVIKKDQVQKEGMMEQITREISVMRRVRHPNIVELKEVMATKSKIFFIMEYVKGGELFKNLVAKGKFKEDTARKYFQQLVSAVDFCHSRGVYHRDLKPENLLLDENEDLKISDFGLSALPDEQLSRNDGLLHTQCGTPAYVAPEVLRRKGYDGAKADIWSCGVILYVLLAGYLPFQDENLNNMYKKIFKSYFEFPPWFSMDSRRLVSKLLMADPERRITIQGIMRIPWFRKDLALPRAFSIQYFQKLEKEEENDHDEEGMSSKQGGVIRKSPSSPAFFNAFELISSMSSGFDLSSLFEIKGKASSMFTSRSTARDVIRKLEKMAKVMRYKVYRVKPFKLKLQCPEEGRKGQLLVTAEVFKVAPEVTVVELSKSSGDTLEYNKFCEEEVRPALKDIVWTWQGTGTENADADNKQVLEQ

>CaCIPK20

MKKVKRKLGKYEVGRTIGEGTFAKVKFARNTETGENVAIKVLAKSTILKHRMVEQIEREISVMKIVRHPCIVRLHEVLASQTTIYIIQEFVAGGELYDKIHLGRLSEDEARRYFQQLIDAIAHCHSKGVYHRDLKPENLLLDFQENLKVSDFGLSALPQQGVELLYTTCGTPNYVAPEVLDNQGYDGAAADVWSCGIILYVLMAGYLPFDEADLPTLYTKIKAAEFSCPFWFFPGATSLIRKIIDPNPQTRIKIEGIKRDPWFRKNYIAVRAKADEVVNLDDVRAVFNDIEDAFVSEKSEDSESGPWIMNAFEMITLSQGLNLSALFDRRQDYVKRQTRFVSREPAKVIIETIEAASESLGLKVHTHDYKTRIEGVTTNRAGQFAVVLEVFQVAPSLFMVDVRKAAGDTLEYHKFYKTFCTKIDNIIWKPKEGQGWLKTGAEPQCGTEVRTNSIAFS

>CaCIPK21

MAPEEKCAVLYGKYELGRVLGSGTFAKVYHARNVITRENLAMKVVGKEKVIKVGMMEQIKREISVMKMVKHPNIVELHEVMASKTKIYFAMEFVKGGELFEKVAKGKLREDNARGYFQQLISAIDFCHSRGVYHRDLKPENLLLDEEGNVKVTDFGLSAFSDHLRQDGLLHTTCGTPAYVAPEVIGKNGYDGSTADIWSCGVILYVLLAGFLPFQDGNIMAMYKKIHKGDFKCPPWFSSDARKLITKMLDPNPNTRITASKIMESNWFKKTVPRTLRTKEEEEFGVEDNQDCVGKAKKIESLNAFHIISLSEGFDLSPLFEEKKKKEKEQMRFATTKPASSVISKLEEVAKTSKFSLKRSDSSVRLQGQESGRKGKLGIAADIFAVTPSFLVVEVKKASGDTLEYNQFCSKELRPALKDIVWKSAPENSSLA

>CaCIPK22

MPEILDDHEGSPSLLSVAVANAELTPAEDVNSNLFDKYKLGKLLGCGAFGKVYHARDINTAQSVAIKVVGKQKIFKGGLTAHVKREISIMRRLRHPHIVRLHEILATKKKIYFALEFAKGGELFSKLAKGRFSEDLSRRYFQQLISAVGYCHSRRVYHRDLKLENLLLDENCDLKVTDFGLSAVGDQMRPGELLHTLCGTPAYVAPEILAKKGYDGAKVDIWSCGIILFVFNAGYLPFNDTNLMTIYRKIYKGEFRCPKWTSPELKRLLTRLLDTNPVTRITVEEIKNDPWFQRGYQEVKSENQFEIKLGLDSAEKFLNAFDIISYSYGFNFSILLKDNGGFIDKERFVSMESREKIIRKVEEVGKAEGMTVVQRSGASVKVEGQNGNFVLMVVVNRLTEKLVIVEIEKRETEGEIWKKKFKPQISSFVYQEEGPNSGS

>CaCIPK23

MPEIEQYSKAMASCESSSLFGKYELGKLLGCGAFAKVYHAREIQNGQSVAVKVINKKRIVNTTLMTNIKREISIMRRLYHPNIVKLFEVLATKTKIYFIMEFVKGGELFGKISKGRFSEDLSRKYFQQLISVVRYCHSRGVYHRDLKPENLLVDENGDLKVSDFGLSALTEQVQQDGLLHTLCGTPAYVAPEILTKKGYDGEKVDIWSCGIILFVLNAGYLPFNDPNLMVMYKKIYKDTNPMTRVTIDEIIKDPWFKKCLKRVKFFEVEEIFDHTNKERLEDVEGDSHMKKTSLNAFDLISFSSGLNLSALFNESFNPMEKTVVEGSPEKIIQKVEELANKDENFKVKRMKEWGIEIKGQIIKFTMVMDIYRLTDYLAVVQVEKTYGDASLFQELWRNKIMPVIRGQQLPEVLQVSSS

>CaCIPK24

MPEILRSESSSSSSATITTAGTSAGSTTGDFKLNLFGKYEVGKLLGCGAFAKVYHARDIRTRQSVAIKTISKQKVLKGGFTEHVKREISIMRRLRHPHIVRLHEVLATKTKIYYVMEFAKGGELFTKVTKGRFSEDLSRRYFQQLISAVDYCHSRGVYHRDLKLENLLLDENWDLKVTDFGLSAVTDQIRPDGSLHTLCGTPAYVAPEILEKKGYSGAKVDLWSCGIILFVLNAGYLPFTDSNLMTMYRKIYKGEFRCPKWTSLGLKMLLTRLLDTNPETRISIEEIRNDPWFKKGYKEVKSHLGDEFELKNGSDVNRDGKFLNAFHIISLSSGVNLSGLLKCLGGKDTVDVERFVSAASPERITQRIEEVAKAEGMRVTGKNGADVRVEGRNGKFVLVTEINQLTEKVVIVEVKRNEIGVGPDREIWKQKFKPELSEFFL

>CaCIPK25

MNQAKIKRRVGKYEIGRTIGEGTFAKVKFARNSETGEPVAIKILDKDKVLKHKMAEQVKREIATMKLIRHPHVVQLYEVLASKTKIFIVLEFVTGGELFDKIVNHGRMHEKEARKYFQQLINAVDYCHSRGVYHRDLKPENLLLDASGNLKVSDFGLSALSQQVRDDGLLHTTCGTPNYVAPEVLNDHGYDGTTADLWSCGVILFVLLAGYLPFDDSNLMNLYKKISAAEFSCPPWMSFGAMKLITRILDPNPMTRITIAEILEDEWFKKDYKYPVFNEKEDVNLDDVEAVFKDSEEHHVTEKKEEQPTPMNAFELISMSKGLNLGNLFDEQEFKRETRFTSKCSANEIISKIEEAAKPLGFDVHKKNYKVVMNFKHAFQLACRFLIT

>CaCIPK26

MVIIQQQQQQQEEEIIRSDQRGKKGMRIGKYEFGRTLGQGNFGKVKYAKHIDSGKSFAIKILEKHRIHDLRITDQIKREIRTLKVLKHPNVVRLYEVLASKTKIYMVLEYVNGGELFDRIASKGKLSESQGRKLFQQLIDGVSYCHDKGVFHRDLKLENVLIDGGRNIKITDFGLSALPQHLRDDGLLHTTCGSPNYVAPEVLSNRGYDGATSDTWSCGVILYVILTGFLPFDDRNLAVLYHKIFKGDAPIPKWLSQGAKNLIRRILDPNPQTRITMAEIKEDEWFKQDYTPAIPDEEEDLESDDASSDDEVLTVHDAPIDTERDSESPSLINNAFQLIGMSSCLDLSGFFENEDASERKIRFTSNLSPKELLERIENLAVQMGFQVQKKTGKLKVLLEHKGQKTQANLSIVAEVFEISTSLYVVELQKSSGDSTVYRQVYTSIPRLDRGRSRTSSVNSTKLLARDPGFASSPRTSDTDQLFFITQLCNRLSDELGVQQSEELQPT

>AtCIPK1

MVRRQEEEKKAEKGMRLGKYELGRTLGEGNFGKVKFAKDTVSGHSFAVKIIDKSRIADLNFSLQIKREIRTLKMLKHPHIVRLHEVLASKTKINMVMELVTGGELFDRIVSNGKLTETDGRKMFQQLIDGISYCHSKGVFHRDLKLENVLLDAKGHIKITDFGLSALPQHFRDDGLLHTTCGSPNYVAPEVLANRGYDGAASDIWSCGVILYVILTGCLPFDDRNLAVLYQKICKGDPPIPRWLSPGARTMIKRMLDPNPVTRITVVGIKASEWFKLEYIPSIPDDDDEEEVDTDDDAFSIQELGSEEGKGSDSPTIINAFQLIGMSSFLDLSGFFEQENVSERRIRFTSNSSAKDLLEKIETAVTEMGFSVQKKHAKLRVKQEERNQKGQVGLSVTAEVFEIKPSLNVVELRKSYGDSCLYRQLYERLLKDVGTSSPEQEIVT

>AtCIPK2

MENKPSVLTERYEVGRLLGQGTFAKVYFGRSNHTNESVAIKMIDKDKVMRVGLSQQIKREISVMRIAKHPNVVELYEVMATKSRIYFVIEYCKGGELFNKVAKGKLKEDVAWKYFYQLISAVDFCHSRGVYHRDIKPENLLLDDNDNLKVSDFGLSALADCKRQDGLLHTTCGTPAYVAPEVINRKGYEGTKADIWSCGVVLFVLLAGYLPFHDTNLMEMYRKIGKADFKCPSWFAPEVKRLLCKMLDPNHETRITIAKIKESSWFRKGLHLKQKKMEKMEKQQVREATNPMEAGGSGQNENGENHEPPRLATLNAFDIIALSTGFGLAGLFGDVYDKRESRFASQKPASEIISKLVEVAKCLKLKIRKQGAGLFKLERVKEGKNGILTMDAEIFQVTPTFHLVEVKKCNGDTMEYQKLVEEDLRPALADIVWVWQGEKEKEEQLLQDEQGEQEPS

>AtCIPK3

MLIPNKKLREMNRRQQVKRRVGKYEVGRTIGEGTFAKVKFARNSETGEPVALKILDKEKVLKHKMAEQIRREIATMKLIKHPNVVQLYEVMASKTKIFIILEYVTGGELFDKIVNDGRMKEDEARRYFQQLIHAVDYCHSRGVYHRDLKPENLLLDSYGNLKISDFGLSALSQQVRDDGLLHTSCGTPNYVAPEVLNDRGYDGATADMWSCGVVLYVLLAGYLPFDDSNLMNLYKKISSGEFNCPPWLSLGAMKLITRILDPNPMTRVTPQEVFEDEWFKKDYKPPVFEERDDSNMDDIDAVFKDSEEHLVTEKREEQPAAINAFEIISMSRGLNLENLFDPEQEFKRETRITLRGGANEIIEKIEEAAKPLGFDVQKKNYKMRLENVKAGRKGNLNVATEIFQVAPSLHMVQVSKSKGDTLEFHKFYKKLSNSLEQVVWTNNEVKKETAK

>AtCIPK4

MESPYPKSPEKITGTVLLGKYELGRRLGSGSFAKVHVARSISTGELVAIKIIDKQKTIDSGMEPRIIREIEAMRRLHNHPNVLKIHEVMATKSKIYLVVEYAAGGELFTKLIRFGRLNESAARRYFQQLASALSFCHRDGIAHRDVKPQNLLLDKQGNLKVSDFGLSALPEHRSNNGLLHTACGTPAYTAPEVIAQRGYDGAKADAWSCGVFLFVLLAGYVPFDDANIVAMYRKIHKRDYRFPSWISKPARSIIYKLLDPNPETRMSIEAVMGTVWFQKSLEISEFQSSVFELDRFLEKEAKSSNAITAFDLISLSSGLDLSGLFERRKRKEKRFTARVSAERVVEKAGMIGEKLGFRVEKKEETKVVGLGKGRTAVVVEVVEFAEGLVVADVKVVVEGEEEEEEVESHWSELIVELEEIVLSWHN

>AtCIPK5

MEEERRVLFGKYEMGRLLGKGTFAKVYYGKEIIGGECVAIKVINKDQVMKRPGMMEQIKREISIMKLVRHPNIVELKEVMATKTKIFFVMEFVKGGELFCKISKGKLHEDAARRYFQQLISAVDYCHSRGVSHRDLKPENLLLDENGDLKISDFGLSALPEQILQDGLLHTQCGTPAYVAPEVLKKKGYDGAKADIWSCGVVLYVLLAGCLPFQDENLMNMYRKIFRADFEFPPWFSPEARRLISKLLVVDPDRRISIPAIMRTPWLRKNFTPPLAFKIDEPICSQSSKNNEEEEEDGDCENQTEPISPKFFNAFEFISSMSSGFDLSSLFESKRKVQSVFTSRSSATEVMEKIETVTKEMNMKVKRTKDFKVKMEGKTEGRKGRLSMTAEVFEVAPEISVVEFCKSAGDTLEYDRLYEEEVRPALNDIVWSWHGDNNNTSSEDC

>AtCIPK6

MVGAKPVENGSDGGSSTGLLHGRYELGRLLGHGTFAKVYHARNIQTGKSVAMKVVGKEKVVKVGMVDQIKREISVMRMVKHPNIVELHEVMASKSKIYFAMELVRGGELFAKVAKGRLREDVARVYFQQLISAVDFCHSRGVYHRDLKPENLLLDEEGNLKVTDFGLSAFTEHLKQDGLLHTTCGTPAYVAPEVILKKGYDGAKADLWSCGVILFVLLAGYLPFQDDNLVNMYRKIYRGDFKCPGWLSSDARRLVTKLLDPNPNTRITIEKVMDSPWFKKQATRSRNEPVAATITTTEEDVDFLVHKSKEETETLNAFHIIALSEGFDLSPLFEEKKKEEKREMRFATSRPASSVISSLEEAARVGNKFDVRKSESRVRIEGKQNGRKGKLAVEAEIFAVAPSFVVVEVKKDHGDTLEYNNFCSTALRPALKDIFWTSTPA

>AtCIPK7

MESLPQPQNQSSPATTPAKILLGKYELGRRLGSGSFAKVHLARSIESDELVAVKIIEKKKTIESGMEPRIIREIDAMRRLRHHPNILKIHEVMATKSKIYLVMELASGGELFSKVLRRGRLPESTARRYFQQLASALRFSHQDGVAHRDVKPQNLLLDEQGNLKVSDFGLSALPEHLQNGLLHTACGTPAYTAPEVISRRGYDGAKADAWSCGVILFVLLVGDVPFDDSNIAAMYRKIHRRDYRFPSWISKQAKSIIYQMLDPNPVTRMSIETVMKTNWFKKSLETSEFHRNVFDSEVEMKSSVNSITAFDLISLSSGLDLSGLFEAKKKKERRFTAKVSGVEVEEKAKMIGEKLGYVVKKKMMKKEGEVKVVGLGRGRTVIVVEAVELTVDVVVVEVKVVEGEEDDSRWSDLITELEDIVLSWHNDIM

>AtCIPK8

MVVRKVGKYELGRTIGEGTFAKVKFAQNTETGESVAMKIVDRSTIIKRKMVDQIKREISIMKLVRHPCVVRLYEVLASRTKIYIILEYITGGELFDKIVRNGRLSESEARKYFHQLIDGVDYCHSKGVYHRDLKPENLLLDSQGNLKISDFGLSALPEQGVTILKTTCGTPNYVAPEVLSHKGYNGAVADIWSCGVILYVLMAGYLPFDEMDLPTLYSKIDKAEFSCPSYFALGAKSLINRILDPNPETRITIAEIRKDEWFLKDYTPVQLIDYEHVNLDDVYAAFDDPEEQTYAQDGTRDTGPLTLNAFDLIILSQGLNLATLFDRGKDSMKHQTRFISHKPANVVLSSMEVVSQSMGFKTHIRNYKMRVEGLSANKTSHFSVILEVFKVAPSILMVDIQNAAGDAEEYLKFYKTFCSKLDDIIWKPPDASMRNRVTKAKSKRR

>AtCIPK9

MSGSRRKATPASRTRVGNYEMGRTLGEGSFAKVKYAKNTVTGDQAAIKILDREKVFRHKMVEQLKREISTMKLIKHPNVVEIIEVMASKTKIYIVLELVNGGELFDKIAQQGRLKEDEARRYFQQLINAVDYCHSRGVYHRDLKPENLILDANGVLKVSDFGLSAFSRQVREDGLLHTACGTPNYVAPEVLSDKGYDGAAADVWSCGVILFVLMAGYLPFDEPNLMTLYKRICKAEFSCPPWFSQGAKRVIKRILEPNPITRISIAELLEDEWFKKGYKPPSFDQDDEDITIDDVDAAFSNSKECLVTEKKEKPVSMNAFELISSSSEFSLENLFEKQAQLVKKETRFTSQRSASEIMSKMEETAKPLGFNVRKDNYKIKMKGDKSGRKGQLSVATEVFEVAPSLHVVELRKTGGDTLEFHKVCDSFYKNFSSGLKDVVWNTDAAAEEQKQ

>AtCIPK10

MENKPSVLTDKYDVGRLLGQGTFAKVYYGRSILTNQSVAIKMIDKEKVMKVGLIEQIKREISVMRIARHPNVVELYEVMATKTRIYFVMEYCKGGELFNKVAKGKLRDDVAWKYFYQLINAVDFCHSREVYHRDIKPENLLLDDNENLKVSDFGLSALADCKRQDGLLHTTCGTPAYVAPEVINRKGYDGTKADIWSCGVVLFVLLAGYLPFHDSNLMEMYRKIGKADFKAPSWFAPEVRRLLCKMLDPNPETRITIARIRESSWFRKGLHMKQKKMEKRVKEINSVEAGTAGTNENGAGPSENGAGPSENGDRVTEENHTDEPTNLNAFDLIALSAGFDLAGLFGDDNKRESRFTSQKPASVIISKLEEVAQRLKLSIRKREAGLFKLERLKEGRKGILSMDAEIFQVTPNFHLVEVKKSNGDTLEYQKLVAEDLRPALSDIVWVWQGEKDELTSQQETEYQQQQQQEQQEQEEPLKF

>AtCIPK11

MPEIEIAAGSGDNNDALFGKYELGKLLGCGAFAKVFHARDRRTGQSVAVKILNKKKLLTNPALANNIKREISIMRRLSHPNIVKLHEVMATKSKIFFAMEFVKGGELFNKISKHGRLSEDLSRRYFQQLISAVGYCHARGVYHRDLKPENLLIDENGNLKVSDFGLSALTDQIRPDGLLHTLCGTPAYVAPEILSKKGYEGAKVDVWSCGIVLFVLVAGYLPFNDPNVMNMYKKIYKGEYRFPRWMSPDLKRFVSRLLDINPETRITIDEILKDPWFVRGGFKQIKFHDDEIEDQKVESSLEAVKSLNAFDLISYSSGLDLSGLFAGCSNSSGESERFLSEKSPEMLAEEVEGFAREENLRMKKKKEEEYGFEMEGQNGKFGIGICISRLNDLLVVVEARRRGGDGDCYKEMWNGKLRVQLIRVCDQTSSTNAAI

>AtCIPK12

MAEKITRETSLPKERSSPQALILGRYEMGKLLGHGTFAKVYLARNVKTNESVAIKVIDKEKVLKGGLIAHIKREISILRRVRHPNIVQLFEVMATKAKIYFVMEYVRGGELFNKVAKGRLKEEVARKYFQQLISAVTFCHARGVYHRDLKPENLLLDENGNLKVSDFGLSAVSDQIRQDGLFHTFCGTPAYVAPEVLARKGYDAAKVDIWSCGVILFVLMAGYLPFHDRNVMAMYKKIYRGEFRCPRWFSTELTRLLSKLLETNPEKRFTFPEIMENSWFKKGFKHIKFYVEDDKLCNVVDDDELESDSVESDRDSAASESEIEYLEPRRRVGGLPRPASLNAFDIISFSQGFDLSGLFDDDGEGSRFVSGAPVSKIISKLEEIAKVVSFTVRKKDCRVSLEGSRQGVKGPLTIAAEIFELTPSLVVVEVKKKGGDKTEYEDFCNNELKPKLQNLTADDVVAEPVAVSAVDETAIPNSPTISFLPSDTE

>AtCIPK13

MAQVLSTPLAIPGPTPIQFMAGLLARIVTKNTNKETSTPESPRSPRTPQGSILMDKYEIGKLLGHGSFAKVYLARNIHSGEDVAIKVIDKEKIVKSGLAGHIKREISILRRVRHPYIVHLLEVMATKTKIYIVMEYVRGGELYNTVARGRLREGTARRYFQQLISSVAFCHSRGVYHRDLKLENLLLDDKGNVKVSDFGLSVVSEQLKQEGICQTFCGTPAYLAPEVLTRKGYEGAKADIWSCGVILFVLMAGYLPFDDKNILVMYTKIYKGQFKCPKWFSPELARLVTRMLDTNPDTRITIPEIMKHRWFKKGFKHVKFYIENDKLCREDDDNDDDDSSSLSSGRSSTASEGDAEFDIKRVDSMPRPASLNAFDILSFSDLSGLFEEGGQGARFVSAAPMTKIISKLEEIAKEVKFMVRKKDWSVRLEGCREGAKGPLTIRVEIFELTPSLVVVEVKKKGGNIEEYEEFCNKELRPQLEKLMHYQADEVEEVMCLPPEIEQ

>AtCIPK14

MVDSDPVEFPPENRRGQLFGKYEVGKLVGCGAFAKVYHGRSTATGQSVAIKVVSKQRLQKGGLNGNIQREIAIMHRLRHPSIVRLFEVLATKSKIFFVMEFAKGGELFAKVSKGRFCEDLSRRYFQQLISAVGYCHSRGIFHRDLKPENLLLDEKLDLKISDFGLSALTDQIRPDGLLHTLCGTPAYVAPEVLAKKGYDGAKIDIWSCGIILFVLNAGYLPFNDHNLMVMYRKIYKGEFRIPKWTSPDLRRLLTRLLDTNPQTRITIEEIIHDPWFKQGYDDRMSKFHLEDSDMKLPADETDSEMGARRMNAFDIISGSPGFNLSGLFGDARKYDRVERFVSAWTAERVVERLEEIVSAENLTVAKKETWGMKIEGQKGNFAMVVEINQLTDELVMIEVRKRQRAAASGRDLWTDTLRPFFVELVHESDQTDPEPTQVHTTS

>AtCIPK15

MEKKGSVLMLRYEVGKFLGQGTFAKVYHARHLKTGDSVAIKVIDKERILKVGMTEQIKREISAMRLLRHPNIVELHEVMATKSKIYFVMEHVKGGELFNKVSTGKLREDVARKYFQQLVRAVDFCHSRGVCHRDLKPENLLLDEHGNLKISDFGLSALSDSRRQDGLLHTTCGTPAYVAPEVISRNGYDGFKADVWSCGVILFVLLAGYLPFRDSNLMELYKKIGKAEVKFPNWLAPGAKRLLKRILDPNPNTRVSTEKIMKSSWFRKGLQEEVKESVEEETEVDAEAEGNASAEKEKKRCINLNAFEIISLSTGFDLSGLFEKGEEKEEMRFTSNREASEITEKLVEIGKDLKMKVRKKEHEWRVKMSAEATVVEAEVFEIAPSYHMVVLKKSGGDTAEYKRVMKESIRPALIDFVLAWH*

>AtCIPK16

MEESNRSSTVLFDKYNIGRLLGTGNFAKVYHGTEISTGDDVAIKVIKKDHVFKRRGMMEQIEREIAVMRLLRHPNVVELREVMATKKKIFFVMEYVNGGELFEMIDRDGKLPEDLARKYFQQLISAVDFCHSRGVFHRDIKPENLLLDGEGDLKVTDFGLSALMMPEGLGGRRGSSDDLLHTRCGTPAYVAPEVLRNKGYDGAMADIWSCGIVLYALLAGFLPFIDENVMTLYTKIFKAECEFPPWFSLESKELLSRLLVPDPEQRISMSEIKMIPWFRKNFTPSVAFSIDETIPSPPEPPTKKKKKDLNEKEDDGASPRSFNAFQFITSMSSGFDLSNLFEIKRKPKRMFTSKFPAKSVKERLETAAREMDMRVKHVKDCKMKLQRRTEGRKGRLSVTAEVFEVAPEVSVVEFCKTSGDTLEYYLFCEDDVRPALKDIVWSWQGDDDEDDVTTNDNVDTNDNKINNVS

>AtCIPK17

MVIKGMRVGKYELGRTLGEGNSAKVKFAIDTLTGESFAIKIIEKSCITRLNVSFQIKREIRTLKVLKHPNIVRLHEVLASKTKIYMVLECVTGGDLFDRIVSKGKLSETQGRKMFQQLIDGVSYCHNKGVFHRDLKLENVLLDAKGHIKITDFGLSALSQHYREDGLLHTTCGSPNYVAPEVLANEGYDGAASDIWSCGVILYVILTGCLPFDDANLAVICRKIFKGDPPIPRWISLGAKTMIKRMLDPNPVTRVTIAGIKAHDWFKHDYTPSNYDDDDDVYLIQEDVFMMKEYEEEKSPDSPTIINAFQLIGMSSFLDLSGFFETEKLSERQIRFTSNSLAKDLLENIETIFTEMGFCLQKKHAKLKAIKEESTQKRQCGLSVTAEVFEISPSLNVVELRKSHGDSSLYKQLYERLLNELGSSSQVQELLA*

>AtCIPK18

MAQALAQPPLVVTTVVPDPPPPPPPPHPKPYALRYMADLLGRIGIMDTDKDGNISPQSPRSPRSPRNNILMGKYELGKLLGHGTFAKVYLAQNIKSGDKVAIKVIDKEKIMKSGLVAHIKREISILRRVRHPYIVHLFEVMATKSKIYFVMEYVGGGELFNTVAKGRLPEETARRYFQQLISSVSFCHGRGVYHRDLKPENLLLDNKGNLKVSDFGLSAVAEQLRQDGLCHTFCGTPAYIAPEVLTRKGYDAAKADVWSCGVILFVLMAGHIPFYDKNIMVMYKKIYKGEFRCPRWFSSDLVRLLTRLLDTNPDTRITIPEIMKNRWFKKGFKHVKFYIEDDKLCREDEDEEEEASSSGRSSTVSESDAEFDVKRMGIGSMPRPSSLNAFDIISFSSGFDLSGLFEEEGGEGTRFVSGAPVSKIISKLEEIAKIVSFTVRKKEWSLRLEGCREGAKGPLTIAAEIFELTPSLVVVEVKKKGGDREEYEEFCNKELRPELEKLIHEEVVVEEALYLPSDTE*

>AtCIPK19

MADLLRKVKSIKKKQDQSNHQALILGKYEMGRLLGHGTFAKVYLARNAQSGESVAIKVIDKEKVLKSGLIAHIKREISILRRVRHPNIVQLFEVMATKSKIYFVMEYVKGGELFNKVAKGRLKEEMARKYFQQLISAVSFCHFRGVYHRDLKPENLLLDENGNLKVSDFGLSAVSDQIRQDGLFHTFCGTPAYVAPEVLARKGYDGAKVDIWSCGVILFVLMAGFLPFHDRNVMAMYKKIYRGDFRCPRWFPVEINRLLIRMLETKPERRFTMPDIMETSWFKKGFKHIKFYVEDDHQLCNVADDDEIESIESVSGRSSTVSEPEDFESFDGRRRGGSMPRPASLNAFDLISFSPGFDLSGLFEDDGEGSRFVSGAPVGQIISKLEEIARIVSFTVRKKDCKVSLEGSREGSMKGPLSIAAEIFELTPALVVVEVKKKGGDKMEYDEFCNKELKPKLQNLSSENGQRVSGSRSLPSFLLSDTD

>AtCIPK20

MDKNGIVLMRKYELGRLLGQGTFAKVYHARNIKTGESVAIKVIDKQKVAKVGLIDQIKREISVMRLVRHPHVVFLHEVMASKTKIYFAMEYVKGGELFDKVSKGKLKENIARKYFQQLIGAIDYCHSRGVYHRDLKPENLLLDENGDLKISDFGLSALRESKQQDGLLHTTCGTPAYVAPEVIGKKGYDGAKADVWSCGVVLYVLLAGFLPFHEQNLVEMYRKITKGEFKCPNWFPPEVKKLLSRILDPNPNSRIKIEKIMENSWFQKGFKKIETPKSPESHQIDSLISDVHAAFSVKPMSYNAFDLISSLSQGFDLSGLFEKEERSESKFTTKKDAKEIVSKFEEIATSSERFNLTKSDVGVKMEDKREGRKGHLAIDVEIFEVTNSFHMVEFKKSGGDTMEYKQFCDRELRPSLKDIVWKWQGNNNNSNNEKIEVIH

>AtCIPK21

MGLFGTKKIGKYEIGRTIGEGNFAKVKLGYDTTNGTYVAVKIIDKALVIQKGLESQVKREIRTMKLLNHPNIVQIHEVIGTKTKICIVMEYVSGGQLSDRLGRQKMKESDARKLFQQLIDAVDYCHNRGVYHRDLKPQNLLLDSKGNLKVSDFGLSAVPKSGDMLSTACGSPCYIAPELIMNKGYSGAAVDVWSCGVILFELLAGYPPFDDHTLPVLYKKILRADYTFPPGFTGEQKRLIFNILDPNPLSRITLAEIIIKDSWFKIGYTPVYHQLSDSIKDNVAEINAATASSNFINAFQIIAMSSDLDLSGLFEENDDKRYKTRIGSKNTAQETIKKIEAAATYVSLSVERIKHFKVKIQPKEIRSRSSYDLLSAEVIEVTPTNCVIEISKSAGELRLYMEFCQSLSSLLTAEVS

>AtCIPK22

MSFTIPRLFLFTLFFCSVFFVCLLMAEDSNSSESIIVNVTGDDNKSALFGKYDLGKLLGSGAFAKVYQAEDLQNGGESVAIKVVQKKRLKDGLTAHVKREISVMRRLRHPHIVLLSEVLATKTKIYFVMELAKGGELFSRVTSNRFTESLSRKYFRQLISAVRYCHARGVFHRDLKPENLLLDENRDLKVSDFGLSAMKEQIHPDGMLHTLCGTPAYVAPELLLKKGYDGSKADIWSCGVVLFLLNAGYLPFRDPNIMGLYRKIHKAQYKLPDWTSSDLRKLLRRLLEPNPELRITVEEILKDPWFNHGVDPSEIIGIQADDYDLEENGKILNAFDLISSASSSNLSGLFGNFVTPDHCDQFVSDESTAVIMRKVEEVAKQLNLRIAKKKERAIKLEGPHGVANVVVKVRRLTNELVMVEMKNKQRDVGLVWADALRQKLRRLINQPVYKVPDKP

>AtCIPK23

MASRTTPSRSTPSRSTPSGSSSGGRTRVGKYELGRTLGEGTFAKVKFARNVENGDNVAIKVIDKEKVLKNKMIAQIKREISTMKLIKHPNVIRMFEVMASKTKIYFVLEFVTGGELFDKISSNGRLKEDEARKYFQQLINAVDYCHSRGVYHRDLKPENLLLDANGALKVSDFGLSALPQQVREDGLLHTTCGTPNYVAPEVINNKGYDGAKADLWSCGVILFVLMAGYLPFEDSNLTSLYKKIFKAEFTCPPWFSASAKKLIKRILDPNPATRITFAEVIENEWFKKGYKAPKFENADVSLDDVDAIFDDSGESKNLVVERREEGLKTPVTMNAFELISTSQGLNLGSLFEKQMGLVKRKTRFTSKSSANEIVTKIEAAAAPMGFDVKTNNYKMKLTGEKSGRKGQLAVATEVFQVAPSLYMVEMRKSGGDTLEFHKFYKNLTTGLKDIVWKTIDEEKEEGTDGGGTNGAMANRTIAKQST

>AtCIPK24

MTKKMRRVGKYEVGRTIGEGTFAKVKFARNTDTGDNVAIKIMAKSTILKNRMVDQIKREISIMKIVRHPNIVRLYEVLASPSKIYIVLEFVTGGELFDRIVHKGRLEESESRKYFQQLVDAVAHCHCKGVYHRDLKPENLLLDTNGNLKVSDFGLSALPQEGVELLRTTCGTPNYVAPEVLSGQGYDGSAADIWSCGVILFVILAGYLPFSETDLPGLYRKINAAEFSCPPWFSAEVKFLIHRILDPNPKTRIQIQGIKKDPWFRLNYVPIRAREEEEVNLDDIRAVFDGIEGSYVAENVERNDEGPLMMNAFEMITLSQGLNLSALFDRRQDFVKRQTRFVSRREPSEIIANIEAVANSMGFKSHTRNFKTRLEGLSSIKAGQLAVVIEIYEVAPSLFMVDVRKAAGETLEYHKFYKKLCSKLENIIWRATEGIPKSEILRTITF

>AtCIPK25

MGSKLKLYPLLNHSSVFHPDSRYQSAPTMEEEQQQLRVLFAKYEMGRLLGKGTFGKVYYGKEITTGESVAIKIINKDQVKREGMMEQIKREISIMRLVRHPNIVELKEVMATKTKIFFIMEYVKGGELFSKIVKGKLKEDSARKYFQQLISAVDFCHSRGVSHRDLKPENLLVDENGDLKVSDFGLSALPEQILQDGLLHTQCGTPAYVAPEVLRKKGYDGAKGDIWSCGIILYVLLAGFLPFQDENLMKMYRKIFKSEFEYPPWFSPESKRLISKLLVVDPNKRISIPAIMRTPWFRKNINSPIEFKIDELEIQNVEDETPTTTATTATTTTTPVSPKFFNAFEFISSMSSGFDLSSLFESKRKLRSMFTSRWSASEIMGKLEGIGKEMNMKVKRTKDFKVKLFGKTEGRKGQIAVTAEVFEVAPEVAVVELCKSAGDTLEYNRLYEEHVRPALEEIVWSWHGDNHNNNIVKSNGNYVSDENSGSDC

>AtCIPK26

MNRPKVQRRVGKYEVGKTLGQGTFAKVRCAVNTETGERVALKILDKEKVLKHKMAEQIRREICTMKLINHPNVVRLYEVLASKTKIYIVLEFGTGGELFDKIVHDGRLKEENARKYFQQLINAVDYCHSRGVYHRDLKPENLLLDAQGNLKVSDFGLSALSRQVRGDGLLHTACGTPNYAAPEVLNDQGYDGATADLWSCGVILFVLLAGYLPFEDSNLMTLYKKIIAGEYHCPPWLSPGAKNLIVRILDPNPMTRITIPEVLGDAWFKKNYKPAVFEEKEEANLDDVDAVFKDSEEHHVTEKKEEQPTSMNAFELISMSRALDLGNLFEEEEGFKRETRFAAKGAANDLVQKIEEASKPLGFDIQKKNYKMRLENVTAGRKGNLRVATEIFQVSPSLHMIEVRKTKGDTLEFHKFYKKLSTSLNDVVWKSGESSGLSK

>BnaCIPK1

MVRKHEEEVRKEMRLGKYELGRTLGEGNFGKVKFAKDTVSGQPFAVKIIDKSRISHLNFSLQIKREIRTL

KMVKHPNIVRLHEVLASKTKIYMVMECVMGGELFDRIVSKGKLSETEGRKMFQQLIDGISYCHSKGVFHR

DLKLENVLLDANGHIKITDFGLSALPQHFRDDGLLHTTCGSPNYVAPEVLANRGYDGAASDIWSCGVILY

VILTGCLPFDDRNLAVLYQKICKGDPPIPRWLSPGARTMVKRMLDPNPATRITVTGIKASEWFKQDYTPS

VPDDDDDEEEEVDTDDDSFSVQELGSEEGKGSESPTIINAFQLIGMSSFLDLSGFFEQEDVSERRIRFTS

NSSARYLLEKIETAVTEMGFSVQKKHAKLKVKQEETNHKGQVGLLVTAEVFEIKPSLNVVELRKSYGDSC

LYRQLCERLLKDVGTSSPEQELVT

>BnaCIPK3

MNRRQQVKRRVGKYEVGRTIGEGTFAKVKFARNSETGEPVALKILDKEKVLKHKMSEQIRREIATMKLIK

HPNVVQLYEVMASKTKIFIILEYVTGGELFDKIVNDGRMKEDEARRYFQQLVHAVDYCHSRGVYHRDLKP

ENLLLDAYGNLKISDFGLSALSQQVRDDGLLHTSCGTPNYVAPEVLNDGGYDGATADMWSCGVILYVLLA

GYLPFDDSNLMNLYKKISSGEFNCPPWLSLGAMKLITRILDPNPMTRVTPQEVFEDEWFKKDYKPPVFEE

KDDSNMDDVDAVFKDSEEHHVTEKKEEQPAAINAFEIISMSRGLNLENLFDPEQEFKRETRITLRGGANE

IIDKIEEAAKPLGFDVQKKNYKMRLENVKAGRKGNLNVATEIFQVAPSLHMVQVSKSKGDTLEFHKFYKK

LSNSLENVVWTNNEVKKAES

>BnaCIPK4

MQSMENSFFPYKSRKHSLQNLYNSPQTSSTKNILRLPPPATPAMGFPSPASPEKNPGTILLGKYELGRRL

GSGSFAKVHVARSIEAGELVAVKIIDKKKTADAGMEPRIIREIEAMRRLHNHPNVLKIHEVMATKTKIYL

VMELAAGGELFTRIRRFGCIQESAARRYFQQLASALTFCHREGIAHRDVKPQNLLLDKQGNLKVSDFGLS

ALPEHRSSTGLLQTSCGTPAYTAPEVIAQKSYDGAKADSWSCGVFLFVLLAGYVPFEDSNVVTMYRKIQG

RDYKFPNWISKPARSIIYKLLDPNPETRMSIEAVTETKWFKKSLETSEFKSSVLESDDRLGKLTSHTITA

FDLISLSAGLDLSGLFERRKRKETRFTATVSAEGVVEKAKTIGEKLGFRVEKKKEAMALGLGKGRTTVMV

EAVELVEGLVVAEVKVVEGEEEESYWSELIVEFEEIVLSWHSDVSVKVESDLA

>BnaCIPK5

MEEERRVLFGKYEMGRLLGKGTFAKVYYGKEITSGESVAIKVINKDHVLKRQGMMEQIKREISIMKLVRH

PNIVELKEVMATKTKIFFVMEFVRGGELFDKISKEGKLHEDAARRYFHQLISAVDFCHSRGVSHRDLKPE

NLLLDENGDLKISDFGLSALPEQILHDGLLHTQCGTPAYVAPEVLRKKGYDGAKADIWSCGVVLYALLAG

CLPFRDENLMNMYRKIFRADFEFPPWFSPESRRLVSRLLVVDPDRRISVTEIMRTPWLRENFNPPLAFEI

DDREETEAVSPKFFNAFEFISSMSSGFDLSNLFQSKRKVQSVFTSRFSAAEITEKIETVTKQMDMNVKTT

KDFRVKIEGKAEGRKGRLSMTAEVFEVAPEMSVVEFCKSAGDTLEYNRFYEEVRPALNDIVWSWHGDNTA

VNQIPDGHGTV

>BnaCIPK6

MVGAKPVMKTEENGSDGGANTSTGLLHGRYELGRLLGHGTFAKVYHARNVTTGKSVALKVVAKEKVIKGG

MEEQIKREISVMKMVKHPNIVELHEVMASKTKIYFAMELVRGGELFAKVAKGRLREDAARVYFQQLISAV

DFCHSRGVYHRDLKPENLLLDEQGNLKITDFGLSAFAEHLRQDGLLHTTCGTPAYVAPEVILKRGYDGAK

ADLWSCGVILFVLLAGYLPFQDDNLVNMYKKIYRGDFKCPGWFSSDARRLVVKLLDPNPNTRITIEKVMD

STWFKKRSKNEPEVQDLDLSVHKFKEETETLNAFHIIALSEGFDLSPLFEEKKKEENREMRFATSRPASS

VISSLEEAARVGDKFDVRKSESRVRMEGKLSGRKGKLAVEAEIFAVAPSFVVVEVKKDHGDTLEYNNFCS

TALRPALKDIFWTSTPA

>BnaCIPK7

MDSLPQPQNQSSPAKILLGKYELGRRLGSGSFAKVHLARSFETNELVAIKIIDKKKTIDSNMEPRIIREI

DAMRRLRDHPNILKIHEVMATKSKIYIVMELASGGELFSKLLRRGRLPESTARRYFQQLASALQFSHRDG

VAHRDVKPQNLLLDKEGNLKVSDFGLSALPEHLRNGLLHTACGTPAYTAPEVISRRGYDGAKADAWSCGV

ILFVLLVGEVPFDDSNIASMYRKIQRRDYKFPSWISKQAKSIIYQMLDPNPATRMGIETVMKTTWFKKSL

ETSQFNFESEPVLEKSSSITAFDLISLSSGLDLSGLFESKKKKERRFTAKVSVVEVEEKAKAIGERLGYT

VRKTKKEVVLGSGRTAIAVEAVELAVGVVVVEVKVVDREEGDSRWSDLITELEVIVLSWHNDVM

>BnaCIPK8

MVVRKVGKYELGRTIGEGTFAKVKFAQNTETGESVAMKIVDRNTILKRKMVDQIKREISIMKLVRHPCVV

RLYEVLASRTKIYIILEYITGGELFDKIVRNGRLSEAEARKYFHQLIDGVDYCHSKGVYHRDLKPENLLL

DSQGNLKISDFGLSALPEQGVTILKTTCGTPNYVAPEVLSHKGYNGAVADVWSCGVILYVLMAGYLPFDE

MDLPTLYSKIDNADFSCPSYFALGAKALIHRILDPNPETRITIAEIRKDEWFAKDYTPVQVIDYEHVNLD

DVYAAFDDPEERKEAQVGTGDTGPLTLNAFDLIILSQGLNLATLFDRGKESMKHQTRFISHKPANVVLSS

MEVVSQSMGFKTHIRNYKMRVEGLSANKSSHFSVILEVFKVAPTFLMVDIQNAAGDAEEYLKFVNVLQFY

KTFCGKLDDIIWKPPDASVRNRVTKTKSKRR

>BnaCIPK9

MIGSRKRTTPASRTRVGNYEMGRTLGEGSFAKVKYARNTVTGDIAAIKILDRDKVLRHKMVEQLKREIST

MKLIKHPNVVEIIEVMASKTKIYIVLELVNGGELFDKIAQQGRLKEDEARRYVQQLINAVDYCHSRGVYH

RDLKPENLLLDANGVLKVSDFGLSAFSLQVREDGLLHTACGTPNYVAPEVLSDKGYDGAAADVWSCGVIL

FVLMAGYLPFDEPNLMTLYKRICKAEFNCPPWFSPGAKNVIKRILDPSPITRISIAELLEDEWFKQGYKT

PSFEQDDEDITIDDVDAAFSNSKECLVTEKKEKPESMNAFELISSSNEFSLENLFEKQAQLVKKETRFTS

NRPASEIMSKMEETAKPLGFNVRKDKYKIKMKGDKSGRKGQLSVATEVFEVAPSLHVVELRKTGGDTLEF

HKFYKTFSSGLKDVVWNTDGAAQDHKV

>BnaCIPK10

MENKPSVLTDKYEVGRLLGQGTFAKVYYGRSVHTNQSVAIKMIDKDKVMKVGLMEQIKREISVMRIAKHP

NVVELYEVMATKTRIYFVMEYCKGGELFNKVAKGKLRDDVAWKYFHQLINAVDFCHSRQVYHRDIKPENL

LLDDNENLKVSDFGLSALADCKRQDGLLHTTCGTPAYVAPEVINRKGYDGTKADIWSCGVVLFVLLAGYL

PFHDSNLMEMYRKIGKGDFKAPSWFAPEVRRLLCKMLDPNPETRITIAKIKESSWFRKGLHMKQKKMEKR

VRETNPLEASAAGPSENGGRVTEENNTDQPTSINAFDIIASSSGFDLTGLFGDVYDKRESRFTSQKPASV

IISKLEEVAQRLKLSIRKREAGLFKLETSKEGRKGKLSMDAEIFQVTPTFHMVEMKKSNGDTLEYQKLVK

EDLRPALADIVWVWQSDKDEQLIPDSNQETEQQQQQEEEEPL

>BnaCIPK11

MPEIEIVAEDGDNRNNNALFGKYELGKLLGCGAFAKVFHARDRRSGQSVAVKILNKKKLLANPALANNIK

REISIMRRLSHPNIVGLHEVMATKTRIFFAMEFVKGGELFNKISKHGRLSEDLSRRYFQQLIAAVGYCHA

RGVYHRDLKPENLLIDESGNLKVSDFGLSALTDHVRPDGLLHTLCGTPAYVAPEILSKKGYDGAKFDVWS

CGIVLFVLAAGFLPFNDPNLMNMYKKIYKGTYRCPRWMSQDLKRFISRLLDINPETRITIDEILKDPWFV

KGGLKTIKFHDEVDLDNGAVKGKEDGGGGESEAVKSLNAFDLISFSSGLDLSGLFDGCSNSVGEPERFLS

QKSPEKLAEEVEEFAKEEKLRVNKMKKEEYELEMEGQNGKFTIGIYISRLNDLLVVVEARRRGGEVDCYK

EMWNDKLRLQLIRFTDQTPNASI

>BnaCIPK12

MAELTKETSLPKERSSPQALILGRYEMGKLLGHGTFAKVYLARNVKTNESVAIKVIDKEKILKGGLIAHI

KREISILRRVRHPNIVQLFEVMATKSKIYFVMEYVRGGELFNKVAKGRLKEDVARKYFQQLISAVTFCHA

RGVYHRDIKPENLLLDENGNLKVSDFGLSAVSDQIRQDGLFHTFCGTPAYVAPEVLARKGYDAGKVDIWS

CGVVLFVLMAGYLPFHDRNVMAMYKKIYKGEFRCPRWFSPELTRLCSRLLETNPEKRFTFPQIMENSWFK

KGFKHVKFYVEDDKLCNVVDDDDELETGSVESDRSSTVSESDVEFFKPARRVGGLPRPASLNAFDIISFS

QGFDLSGLFDDDGEGSRFVSGAPVSKIISKLEEIAKVVSFTVRKKDCRVSLEGSRQGVKGPLTIAAEIFE

LTPSLVVVEVKKKGGDRTEYEEFCNKELKPKLQTLTADEVDDPVAVSAVVDETASGVANSPPVCFLPSDT

E

>BnaCIPK13

MAPVPSPQVLLPRLLGKVITKDVKKETSTPESPKSPKTPQGSILMDKYELGKLLGHGSFAKVYLARNINT

GENVAIKVIDKEKIVKSGLAGHIKREISILRRVRHPYIVHLLEVMATKTKIYIVMEYVRGGDLYTKVSKG

RLREGVARRYFQQLISSVSFCHGRGVYHRDLKLENLLLDDEGSLKVSDFGLSVVSEQLKQEGICQTFCGT

PAYLAPEVFTRKGYDAAKADVWSCGVILFVLMAGYLPFDDKNVMVMYKKIFKAQFKCPHWFSPELTRLMG

RILDTNPDTRITISEIMKHRWFKKGFKDVKFYIENDKLCREGDNDDDDSSSLSSGRSSTASEGDAEFVIK

RVGSMPRPASLNAFDIISFSSGFDLSGLFEEGGQGARFVSAAPVIKIISKLEEIANVAKFTVRKKDWSVR

LEGSREGAKGPLTIKVEIFELTPSLVVVEVKKKGGFIEEYEEFCNKELRPQLEKLMHYQADEVEVAMCLV

PC

>BnaCIPK14

MEEKQRGAVLGKYEVGKLVGCGAFAKVYRGRDTGTGQSVAIKVVSKQRLNKGGLNVKREIAIMHRLRHPY

IVRLSEVLATKSKIFFVMEFAKGGELFAKVSKGRFSEDLSRRYFHQLISAVGYCHSRGVFHRDLKPENLL

LDDKLDLKISDFGLSALAEQIRPDGLLHTLCGTPAYVAPEVLAKKGYDGAKIDVWSCGVILFVLNAGYLP

FNDHNLMVMYRKIYKGEFRIPKWTSPDLRRLLTRLLDTNPLSRITIEEIIRDPWFRQGYDDRMSKFHLED

SDMKLPANEMRMNAFHIISGSPGFNLSGLFSGKFDRVERFVSGWAAARVVEKLEEMAAAENLTVASKEAW

GMKIQGEGGSFAMVVEINQLTDELVMIEVRQRQRAGGDIWSDTLRPFLVEIAHKPDDTVAGLQTTHYQEG

ASSLRDVNTTT

>BnaCIPK15

MEKKRTVLMLRYEVGKLLGQGTFAKVYHARNLISGDGVAVKVIDKDRILKVGGMTDQIKREISVMRLLSH

PNIVHLHEVMATKSKIYFVMEHVKGGELFNQVSTGKLREGVARKYFQQLVRAVHYCHTLGVCHRDLKPEN

LLLDEHGNLKVSDFGLSALSDNRRQDGLLHTTCGTPAYVAPEVINRNGYDGFKADVWSCGVILFVLLAGY

LPFRDSNLIDLYKKIGKAEFKFPNWFAPGARRLLKKILDPNPNTRISTEKIMQSSWFRRGLEEDKDHADA

ETDDTEPMPLNDDCDKAKKQCINLNAFEIISLSTGFDLSGLFEKGEEKEEMRFTSNREASEIMEKLVEVG

RELKMNVRKKEEGWRVKMEGRKAGAGVEAEVFEIAPSFHMVVMKKSGGDTAEYKRLMKESIRPALMDIVL

AWH

>BnaCIPK16

MEESKTVLFDKYTIGKLLGTGNFAKVYHGTEISTGDEVAIKVIKKDLVLKRRGMTEQIEREIAVMRLLRH

PNVVELREMMATKTKIFFIMEYVDGGELFERLDKDGKLPEDLARKYFQQLISAVDFCHSRGVYHRDIKPE

NLLLDGHGDLKVTDFGLSALMMPEGLGGRRGSSDDLLHTRCGTPAYVAPEVLRNKGYDGAMADIWSCGIV

LYALLAGFLPFIDENVMTLYTKIFKAEIDFPPWFSTESQELISKLLLSDPEQRITMSEIKQFPWFRKNFT

PPEAFSIDETLPSPPPEPPSKKNKKHLNVDEEDGGSSPRSFNAFQLISSMSSGFDLSSLFEIKRKPKRMF

TSKLPAIAVKERLEEAAPEMNMRVKHVKDCKMKLQSRTEGRKGRLSVTAEVFEVAPEVSVVEFYKTSGDT

LEYQLFCEDDVRPALKDIVWSWQGDDDDDHDNDNVIN

>BnaCIPK17

MVTKGMRVGKYELGRTLGEGNSAKVKLATDTVSGQSFAVKIIDKSSIKRLNVSFQIKREIRTLKVLKHPN

IVRLHEVLASKTKIYMVLECVTGGDLFDRVVSRGKLSETEGRTLFQQLIDGVSYCHNKGVFHRDLKLENV

LLDAKGDIKITDFGLSALPQHFREDGLLHTTCGSPNYVAPEVLANKGYDGAASDIWSCGVILYVILTGCL

PFDDANLAVLCRNIFKGDPPIPRWLSPGAKTMIKRMLDPNPITRITIAGIKANDWFNHDYTPSSSDDEND

ACSFQEDVSEEEKSPDSPTVINAFELIGMSSFLDLSGLFETEKVLDRQIRFTSNRLAIDVLEKIKTTLME

MGFSVQKKHTMLKAIQQESSRKGQSGLSLTAEVFEIIPLLNVVELRKTHGDSLLYKQLCDRLSNELSTSS

HVQKL

>BnaCIPK18

MSQALAPPPLLVVTTVVPDPPHPPPPPQQKPYALQYVTELLSRIGIKETDKDGNISPQSPRSPRNNILMG

KYELGKLLGHGTFAKVYLALNIKSGENVAIKVIDKEKIMKSGLVAHIKREISILRRVRHPYVVHLFEVMA

TKTKIYFVMEYVPGGELFNKVAKGRLPEDTARRYFQQLISSVSFCHGRGVYHRDLKPENLLLDAKGNLKV

SDFGLSAVAEQLRIDGLCHTFCGTPAYLAPEVLTRKGYDAAKADVWSCGVILFVLMAGHIPFYDKNIMAM

YKKIYKGEFRCPRWFSSDLIRLLTRLLDTNPDTRITIPEIMKSRWFKKGFKHVKFYIEDDKLCREDEEEE

EESYSSGRSSTVSESDAEFDVKRIGSMPRPASLNAFDIISFSSGFDLSGLFEEGGEGTRFLSGAPVSEII

AKLEEIARVVSFTVRKKEWSLRLEGCREGAKGPLTIGAEIFELTPSLVVVEVKQKGGDRDEYEEFCNKKL

RPELEKLMHNKAEEKGEVVVEEEETVCLPSDTV

>BnaCIPK19

MAELLRKVKSMKKKDKSNTQALILGKYEMGRLLGHGTFAKVYLARNATSGESVAIKVIDKEKVLKSGLIA

HIKREISILRRVRHPNIVQLFEVMATKSKIYFVMEYVRGGELFNKVAKGRLKEDIARKYFQQLISAVSFC

HFRGVYHRDLKPENLLLDEHGNLKVSDFGLSAVSDQIRQDGLFHTFCGTPAYVAPEVLARKGYDGAKVDI

WSCGVILFVLMAGFLPFHDRNVMAMYKKIYRGDFRCPRWFPVEINRLLIRMLETKPERRFTMPQIMETSW

FKKGFKHIKFYVEDDHKLSSADDDIESVESVSERSSTVSESEFESVDARRRSMPSMPRPASLNAFDLISF

SAGFDLSGLFDDDGEGSRFVSGAPVNQIISKLEEIAKVVSFTVRKKDCRMSLEGSREGSVNGPLTIAAEI

FELTPALVVVEMKKKGGDKVEYDEFCNKEVKPKLQNLSADNGEAVSASRSLPAYMLSDTD

>BnaCIPK20

MDKNGIVLMKRYELGRLLGKGTFAKVYHARNLKTGESVAIKVIDKEKVTKVGLIDQIKREISVMRLVRHP

NVVFLHEVMASKTKIYFVMEYVKGGELFNKVSKGKLKEDNARKYFQQLIGAIDYCHSRGVYHRDLKPENL

LLDENGDLKISDFGLSALRESRKQDGLLHTTCGTPAYVAPEVICKKGYDGDKADVWSCGVVLYVLLAGFL

PFHQQNLVEMYRKIKNGEFKCPNWFPPEVKKLLSRILDPNPNSRIKINKIMENSWFQKGFKKIETPKSPD

SNQIESLISDVHAAFAVQPMCYNAFDLISSLSQGFDLSGLFEKEERSESMFTTKKEAKEIVSKFEEIAMN

SERFDLKKSNVGVKMEDRREGRKGQLAIDVEIFEVTKSFHMVEFKKSKGDTMEYKQFCDGELKPSLKDIV

WKWQGNNNNTNENVA

>BnaCIPK22

MAEEDSSDSIIVNVDGDDNKSALFGKYDIKKHLGSGAFAKVYEAEDLHNKGQSVAIKVVQKKRLKDGLTA

HVKREISVMRRLRNPHIVLLSEVLATKTKIYFVMELAQGGELFSRVSSNRFTESLSRKYFRQLISAVRYC

HARGVFHRDLKPENLLLDERRDLKVSDFGLSAMKEQIKADGMLHTLCGTPAYVAPELLTKKGYDGSKADI

WSCGVVLFLLNAGYLPFRDPNISGLYRKIRMAQYRMPEWTSSGLRHILSRLLEPDPEKRITVEEILKDPW

FNHGVDPSEMIGMQADDYDLEESGKKLNAFELIASSSTANLAGLFGNFVTPDHCDQFISDENPAEIMVKV

VEVAKKMNLRIAKKKERAVKLEGPQGVANIVVKIRRLTDELVMVEMKNKQRDVGIVWADELRQKLRRLIN

QPVNRVPDKP

>BnaCIPK23

MASRTTPSRSTPSRSTPSSGSSSSSISRTRVGKYELGRTLGEGTFAKVKFARNVDKGESVAIKVIDKEKV

LKNKMIAQIKREISTMKLIKHPNVIRMIEVMASKTKIYFVLELVTGGELFDKISSTGRLKEDEARKYFQQ

LINAVDYCHSRGVYHRDLKPENLLLDANGTLKVSDFGLSALPQQVREDGLLHTTCGTPNYVAPEVINNKG

YDGAKADLWSCGVILFVLMAGYLPFEDSNLTSLYKKIFKAEFTCPPWFSASAKKLIKRILDPKPATRITF

AEVIENEWFKKGYKAPKYENADVSLDDVDAIFDESGESKNLVVERKEEGPKNPVTMNAFELISTSQGLNL

GSLFEKQMGLVKRKTRFTSKCSANDIVTKIEAAAGPLGFDVKKNNYKMKLLGEKSGRKGQLAVATEVFQV

APSLYMVEMRKSGGDTLEFHKFYKNLTTGLKDIVWKTIDEEKEEGTEGKVSGGTTNGTVAAS

>BnaCIPK24

MDQKKRITTKKTRKLGKYEVGRTIGEGSFAKVKFARNTDTGENVAIKIMAKSTILKNKMADQIKREISIM

KIVRHPNIVRLYEVLASPSKIYIVLEFVTGGELFDRIVHKGRLEESEARKYFQQLIDAIAHCHCKGVYHR

DLKPENLLLDNNGNLKVSDFGLSALPQQGVELLRTTCGTPNYAAPEVLNGQGYDGSAADIWSCGVILFVI

MAGFLPFSETDLPSLYRKISAAEFSCPQWFSADVKSLIQRILDPNPNTRIQIQGIRKHSWFRINYMPTRA

KEEEEVNLDDVRAVFDGIEGSYVAENIERRYEGPLMMNAFEMITLSQGLNLSALFDRRQDFVKRQTRFVS

RREPSVIIANIEAVATSMGFRAHTRNFKTRLEGLSSIKAGQFAVVIEVYEVAPSLFMVDVRKAAGETLEY

HKFYKKLCEKLENIIWRATEGMPKPELFRTITF

>BnaCIPK25

MEEERRVVLFGKYEIGRLLGKGTFAKVYHAKHITTGESVAIKIINKDHVMKRPGMMDQIKREISIMRLVR

HPNIVELKEVMATKTKIFFVMEFVRGGELFAKVVKGKLKEDAARRYFQQLISAVDFCHSRGVSHRDLKPE

NLLVDENGDLKVSDFGLSALPEQLLQDGLLHTQCGTPAYVAPEVLRKKGYDGAKADIWSCGVVLYVLLAG

YLPFQEENLMNMYRKIFKAEFDFPPWFSPEARRLISKLLVVDPEKRISIPAITRTPWFRKTFKAPMIAAI

NSPGNELEEEEDGAAVSPKFFNAFQFISSMSSGFDLSSLFESKRKVRSMFTSRWSAAEIVAKIEGIGKEI

GMKVKRTKDFKVRMQGKTEGRKGQISVTAEVFEVAPEVAVVELCKSAGDTLEYNKLYEEQVRPALKDIVW

SWHGDSNNNNNIIIDDSNINNYISDENSGSDCERI

>BnaCIPK26

MNRPKVQRRVGKYEVGKTIGQGSFAKVRYAKNTETGESVALKILDKEKVLKNKMSEQIRREISTMKLINH

PNVVRLYEVLASKTKIYIVLEFGVGGELFDKIVHDGRLQEDAARKYFQQLINAVDYCHSRGVYHRDLKPE

NLLLDAQGNLKVSDFGLSALSRQVRGDGLLHTACGTPNYAAPEVLNDQGYDGATADLWSRGVILFVLLAG

YLPFDESNLMTLYKKITAAEYTCPPWLSPGAKKLIDRILDPSPVTRISIQEILEDAWFKKNYKRPVFEEK

EEANLDDVEAVFKDSEEHHVTEKKEEQPTSMNAFELISMSRALDLGNLFEEEEEGYKRETRFAATGPANE

LVQKIEEASKPLGFDIQKKNYKMRLENVNAGRKGNLKVATEIFQVSPSLHMVEVRKTKGDTLEFHKFYKK

LSTTLNDVVWKSGESSGLNNK

>MeCIPK1

MRLGKYELGRTLGEGNFGKVKLAKNIESGQPFAVKILEKNRIIHLNITDQIKREIATLKLLKHHNVVRLYEVLASKSKIYMVLEYVNGGELFDRIASKGKLSEAQGRKLFQQLIDGVSYCHNKGVFHRDLKLENVLIDTKGNLKISDFGLSALPQHFRDDGLLHTTCGSPNYVAPEILSNRGYDGATSDIWSCGVILYVILTGYLPFDDRNLAVLYQKIFKGEAQIPKWLSPGAQNMIRRILDPNPLTRITVAGIEADEWFKQDYTPADPSEEDDDIHIDNEAFSMQEVPLEGERSPGSPVLINAFQLIGMSSCLDLSGFFEKEDVSERKIRFTSNHSLKDLLEKIEDIARGMGFRIQKKNGKLKVVQENKEQRSLRSLYVAAEVFEISPSLHVVELQKSYGDASVYRQLCKKLSNDLSVPSGQGLLTAQV

>MeCIPK2

MEHKPGILTQKYEIGRLLGQGTFAKVYYARSISTNQSVAIKVIDKEKILRAGLVDQIKREISVMRIVRHPNIVHLYEVMATKSKIYFVIEYCKGGELFNKVAKGKLKEDVAHMYFHQLINAVDFCHSRGVYHRDIKPENLLLDENENLKISDFGLSALAESKRQDGLLHTTCGTPAYVAPEVINRKGYDGAKADIWSCGVVLFVLLAGYLPFHDSNLMEMYRKIGKADFKCPNWFPQEARRLLFKMLDPNPNTRISMDKIKESSWFRKVFNPKQKKTEAEVQDILDSNGSGPSENSSVSSEAKQESVKPPRLNAFDIISLSAGFDLSGLFDENSQLREARFTSLQPASVIISKLEDVAKHLRLKIMKKEEGLLKMESLQEGRKGPLCIDAEIFEVAPNFHLVEVKKSNGDTMEYQKILKEDIKPALQDIVSAWQGEQLLQPQQQQQEQETALAASAVMT

>MeCIPK3

MSQPKIKRRVGKYEVGKTIGEGTFAKVKFARNSETGEPVALKILDREKVLKHKMAEQIKREIATMKLIKHPNVVRLFEVMGSRTKIFIVLEFVTGGELFDKIVNHGRMREDEARRYFQQLINAVDYCHSRGVYHRDLKPENLLLDACGNLKISDFGLSALSQQVRDDGLFHTACGTPNYVAPEVLNDRGYDGATADIWSCGVILFVLLAGYLPFDDDNLINLYKKISAAEFTCPPWLSFGAMKLITRILDPNPVTRITISEILEDEWFKKDYKPPVFEEKDDTNLDDVEAVFKDSKDHLVTEKKEEHPAAMNAFELISMSKGLNLGNLFDSEQVCMLEGLLYVVMLNYIFKIDGALCGCACA

>MeCIPK4

MSQPKIKRRVGKYEVGRTIGEGTFAKVKFARNSETGDPVALKILDKEKVLKHKMAEQIKREVATMKLIKHPNVVRLYEVMGSKTKIFIVLEFVTGGELFDKIVNHGRMREDEARRYFQQLINAVDYCHSRGVYHRDLKPENLLLDANGNLKVSDFGLSALSQQVRDDGLLHTTCGTPNYVAPEVLNDRGYDGATADMWSCGVILFVLLAGYLPFDDPNLMNLYKKISAAEFTCPPWLSFGAMKLITRILDPNPMTRITIPEILEDEWFKKDYKPPVFEEKEDTNLDDVEAVFKDSEEHHVTEKKEEQPTAMNAFELISMSKGLNLGNLFDAEQGFKRETRFTSKRPANEIIHKIEEAAKPLGFDVHKKNYKMRLENMKAGRKGNLNVATEIFQVAPSLHMVEVRKAKGDTLEFHKFYKNLSTCLDDVVWKSEDDMQEMK

>MeCIPK5

MKSRGSAATGNRTRVGKYELGRTLGEGTFAKVKFATNTETGENVAIKILDKENVLKHKMIVQIKHEISTMQLIRHPNVIRMYEVMACKTKIYIVLEFVTGGELFDKIASRGRLKEDEARKYFQQLINAVDYCHSRGVYHRDLKPENLLLDASGVLKVSDFGLSALPQQVREDGLLHTTCGTPNYVAPEVISNKGYDGAKADLWSCGVILFVLMAGYLPFEESSLMALYKKIFKAEFTCPPWFSSSAKKLIKRILDPNPLTRITFAEVIANEWFKKGYKPPIFEQSEVILDNVSSIFNDTGDCHNLVVERQEAPIGPVAPITMNAFELISTSQGLNLGSLFEKKMGLVKRETRFTSKHSANEIVSKIEEAAMPLGFEVKKNNFKMKLQGEKTGRKGQLSVATEIFEVAPSLHMVEVRKSCGDTLEFHKFYKNLSVGLKDVVWKTIDEEKEEEKVNSNGATVVPA

>MeCIPK6

MAERSRDGHPTVLHGRYELGRMLGHGTFAKVYHARNLQTGKSVAMKVVGKDKVIKVGMMEQIKREISVMKMVEHPNIVELHEVMASKSKIYFSMELVRGGELFAKIAKGRLREDVARVYFQQLISAIGFCHSRGVYHRDLKPENLLLDEDGNLKVTDFGLSAFTDHLKQDGLLHTTCGTPAYVAPEVIGKKGYDGAKADLWSCGVILYVLLAGFLPFQDDNIVAMYRKIYRGDFKCPPWFSPEARRLITKLLDPNPSSRITISKIMDSSWFKKSVPKTVRTKEEMEFDAFNGEEDGNNGGKSKQPETLNAFHIISLSEGFDLSPLFEEKKRDEEEELRFATTRPASSVISRLEEVAKAGKFSVKKSETKVRMQGQESGRKGKLGIAAEIFAVTPSFLVVEVKKDNGDTLEYKQFCSKELRPALKDLVWTSPAGTSTIA

>MeCIPK7

MGERRRDGQSTLLHGRYELGRMLGHGTFAKVYLARNLQSGKSVAMKVVGKEKVIKVGMMEQIKREIAVMKMVKHPYIVDLHEVMASKSTIYFAMELVRGGELFSRISKGRLREDVARVYFQQLISAVDFCHSRGVYHRDLKPENLLLDEDGNLKVTDFGLSAFSEHLKQDGLLHTTCGTPAYVAPEVIGKKGYDGAKADLWSCGVILYVLLAGFLPFQDDNIVAMYRKIYRGDFKCPPWFSPEARRLITKLLDPNPSTRIAISKVMDSSWFKKSMPKTIRSKEEMEFEAFNCEEESKNGGKSKQPETLNAFHIISLSEGFDLSPLFEEKKREEKEELRFATTRPASSVISRLEEVAKAGKFSVKKSETKVRMQGQESGRKGKLAIAAEIFAVTPSFLVVEVKKDNGDTLEYKQFCSKELRPALKDIVWTSPADNSTIA

>MeCIPK8

MVVRKVGKYEIGRTIGEGTFAKVKFAQNTETGESVAMKVLDRSTIIKHKMVDQIKREISIMKLVRHPYVVRLHEVLASRTKIYIILEFITGGELFDKIVHHGRLSEAEARRYFQQLIDGVDYCHSKGVYHRDLKPENLLLDSQGNLKISDFGLSALPEQGVSLLRTTCGTPNYVAPEVLSHKGYNGAVADVWSCGVILYVLMAGYLPFDELDLTTLYGKIENADFSCPSWFPVGAKSLIHRILDPKPETRITIEQIRNDEWFNKGYVPARLIEYEDVNLDDVDAAFNDPEEQKTDEQCGNEDTGPLILNAFDMIILSQGLNLATLFDRGKDSMKYQTRFISRKPARVVLSSMEVVAQSMGFKTHIRNYKMRVEGLSANKTAHFSVILEVFEVAPTFLMVDIQKAAGDASDYLKQS

>MeCIPK9

MSVKVPSKQTSTRVGKYELGRTLGEGTFAKVKFAKNVETGDSVAIKILDREQVLRLKMVEQLKREISTMKLIKHPNVIKIYEVMASKRKIYIVLEFVDGGELFDKIATQGRLKEDEARRYFHQLINAVDYCHSRGVSHRDLKPENLLLDSLGVLKVSDFGLSAVLSNQIRGDGLLHTACGTPNYVAPEVLKDKGYDGTGSDVWSCGVILFVLMAGYLPFDEPSFMGLYGKICSADFTFPSWFSSGARKLIKRILDPNPVTRITIPEMLEDEWFKKGYKPPQFQQEDDVNLDDVDAAFDDSKEHLVTERKGKPVSMNAFELISKTQGFSLENLFAKQAGIVKRETRFASHSPAKEIMSKIEEAAKPLGFNVDKRNYKMKLKGDKNGRKGQLSVSTEVMS

>MeCIPK10

MSVKAPSTRTRVGKYELGRTLGEGTFAKVKFAKNVETGENVAIKIIDRDQILRLKMVEQLKREISTMKLIKHPNVIQIYEVMASKRKIYIVLEFVDGGELFDKIARNGRLKEDEARRYFHQLIDAVDYCHSRGVSHRDLKPENLLLDSYGTLKVSDFGLSAVLSKQVQGDGLLHTACGTPNYVAPEVLKDKGYDGTGSDIWSCGVILYVLMAGYLPFDEPSLMGLYKKICSADFTFPSCFSSGSRKLIKRILDPNPVTRITIPQILEDEWFKKGYKPPQFEQGDDVNLDDVDAAFNDSKEHLVTERKEKPVSMNAFELISKTQGFSLDNLFEKQAGLVKRETRFASRTPANEIMSKIEDAAKPLGFNVDKRNYKMKLKGDKNGRKGQLSVATEVFEVAPSLHIVELRKIGGDTLEFHKFYKTFSTGLKDVVWKSDGT

>MeCIPK11

MPPEIEQVAENALFGKYELGKLLGCGAFAKVYHARNIRTGQSVAIKIINKKKIANTTLMSNIKREISIMRRLNHPHIVKLYEVLASKTKIYFVMEFVKGGELFAKVAKGRFSEDLSRKYFQQLISAVAYCHARGVFHRDLKPENLLLDESGNLKVSDFGLSAVTDQIRTDGLLHTLCGTPAYVAPEILAKKGYDGAKVDIWSCGVILFVLTAGYLPFNDPNLMVMYKKIYKGEFRCPKWMSPDLKRFLSRVLDTNPQTRITVDEILKDPWFKRGGLKEIKFYDDYVGIDDTDKTDKQEPDVTNLNAFDLISFSSGLDLSGLFDDSYNAVEDGDRFVSSESPEKLVQKVEEFAKAERLRAKRKKEWAFEIEGRNGNFGMEVEVYRLTENLAVVEARRRGGE

AGCFKQMWKNKLKPELSGLTVSQPGTQVAGNC

>MeCIPK12

MATKIHSNSNSNQSPLLGRYEIGKLLGHGTFAKVYHARNVKTSESVAIKVIDKEKILKGGLIAHIKREISILRRVRHPNIVQLFEVMATKAKIYFVMEYVRGGELFNKVAKGRLKEEVARKYFQQLISAVGFCHARGVFHRDLKPENLLLDENGDLKVSDFGLSAVSDQIRQDGLFHTFCGTPAYVAPEVLARKGYDAAKVDIWSCGVILFVLMAGYLPFHDQNVMVMYKKIYKGEFRCPRWFSPELIRLLSKLLDTNPETRITIPEIMENKWFKRGFKHIKFYIEDDKVFSVDVEGQQDDAGSSSDQSQCESEPEMETRRRITSLPRPASLNAFDIISFSPGFDLSGLFEEGGEGARFVSGAPVSKIISKLEEIAKVVSFTVRTKDYRVSLEGSREGIKGPLTIAAEIFELTPKLVVVEVKKKGGDKGEYEEFCNKELKPGLQKLMQEESETADAASSQLPTEPLQLPTEPLQLPTESLQIPTEPLPIDTTHLPSDTE

>MeCIPK13

MEHKGNVLMQRYDFGRLLGQGNFAKVYYARNLQTGQSVAIKVIDKEKILKVGLINQIKREISVMKLVKHPNVLQLYEVMASKSKIYFAIEYAKGGELFKKVAKGRLKEDVARNYFQQLISAVDFCHSRGVYHRDLKPENLLLDENGVLKVSDFGLSALVESKSQDGLLHTTCGTPAYVAPEVIKSKGYDGAKADIWSCGVILYVLLAGYLPFHDANLISLYRKITKAEYKCPNWFLPEVRKLVSKMLDPNPSTRISIEKIMENPWFRKGFNPKAAETKIDVKKLDPLVADTTLDPYENAFIFAEDKKELNRPISLNAFDIISLSNGLDLSGFFTKSQQEKETKFTSMHSASTIISKLEDTAKHLHLEVKKKDRGLLKLKGSEEGRKGALSIDAEIFEFTPSFHMVEVKKSAGDTLEYTQILEQGLRPALKDIVWVWQGEQQQKQQQAHSS

>MeCIPK14

MAEDPSNPSPAIAEITPDINLFDKYELGKLLGCGAFAKVYHARNVVTGQSVAIKAVSKQKVMKGGFIGQIKREISIMRRLHHPRVVKLLEVLATKTKIYFVMEFAKGGELFAKVAKGRFSEDLSRKYFQQLITAVGYCHARGVFHRDLKPENLLLDENWDLKVSDFGLSAVNDQIQSDGLLHTLCGTPAYVAPEILGKKGYDGAKADVWSCGVILYVLIAGYLPFNDTNLMVMYRKIYRGQFRFPKWTSPDLRRFLSRLLDANPETRITVDEILRDPWFKQDFKGIEFHLEDSDLKRQENHKSLNAFDIISFSSGFDLSGLFNDCDISACNERFVSSESPAKIIDRIEEIAEAENVKVTKNRDCGAKLEGIDGTFVMAIEIYQLTEQLVVVEVKGKEMNAKPGQEIWKDKLRPKLGSLVYEAAPTASGE

>MeCIPK15

MADQEPTNPLPDVNLFEKYDLGKLLGSGAFAKVYHARNVRTGQSVAIKAISKQKVVKGGFMGQVKREISIMRRLHHPNVVKLLEVLATKTKIYFVLEFAKGGELFTRVAKGRFSEGLSRRYFQQLITAVEYCHSRGVFHRDLKLENLLLDDNWDLKVTDFGLSAVKDQIEPDGLLHTLCGTPAYVAPEILGKKGYDGAKVDVWSCGIILYVLIAGYLPFNDTNIMSMYRKIYRGQFRFPKWTSPDLRRFLSRLLDTNPKTRITVDEIIQDSWFKKDYKGMKFQLEGFDMKVQENVQNQKSLNAFDIISFSSGFDLSCLFNECDVSASSERFVSCESPAKIIKRVEEIARTENIKLMKNKGWGAKLEGCDGSFAMAIEIYKLTDQLVVVEVKGKEMNAIPNQEIWKHKLRPQLDSLVHKPATPAPSVTDYPISS

>MeCIPK16

MEVETERVAHKDCHINGAKHVLFGKYEMGKLLGQGTFAKVYKAKNLVTQERVAIKAIHKDHVKKKGLIEQIKREISVMHLVKHPNIVELKEVMATKSKVFFVMEYVRGGELFAKLQQGKLKEDLARKYFQQLISAVDFCHSRGVSHRDLKPENLLLDENGDLKVSDFGLSALPEQHWNDGLLHTQCGTPAYVAPEVLRKKGYDGAKADIWSCGVILFVLLAGYLPFQNANLMKMYVKIFKAEYEFPPWISPEARRLISKHLVVDPERRITIPHIRQNPWFRRGLTTPITISNDLEENEKGRELIGEEITKLSETNHSPPFYNAFEFISAMSSGFDLSSLFENKKKSGSMFTSKCAASVILTKLGSAAKKLNFRVLSDSEFKVKMQGKEEGRKGKLAVTAEVFEVAPEVAVVEFSKSAGDTLEYTKFCEEDVRPALKDIVWSWQGEDNCHQLERTSGN

>MeCIPK17

MEHKGNVLMQRYDFGRLLGQGNFAKVYYARNLETGQSVAIKVIDKEKILKVGLINQIKREISIMKLVKHPNVLQLDEVMA

SKSKIYFVIEYAKGGELFKKVAKGRFNEDVARNYFHQLISAVDFCHSRGVYHRDLKPENLLLDENGVLKVSDFGLSALVESKAQDGMLHTTCGTPAYVAPEVISRKGYDGAKADIWSCGVILYVLLAGYLPFHDANLISLYKKISKAEYKCPGWFSSEVRKLVSKMLDPNPRNRISIAKIMETSWFKKGFNSKSVKAKTDGKNSEILVTDLSFHPSEESPSFSEDKKELGKPTSLNAFDIISLSDGFNLSGFFAKSNKEKEAKFTSMHTASTIITKLEDTAKNLQLEVKQKDGGLLKLEGSEEGRKGALSIDAEIFEFTPSFHLVEVKKSAGDTLEYTQVLEQGIRPALKDIVWAWQGEKQQQQEEQQQENSS

>MeCIPK18

MENKGHVLMRKYEMGKLLGQGTFARVHHARNLQTGVSVAIKIIDKEKVLKVGMMEQIKREISVMRLIRHPNVVELYEVMATKSKIYFVMEYVKGGELFNKVAKGKLKEDIGRKYFQQLISAVDYCHSRGVSHRDLKPENLLLDENGNLKVSDFGLSALAECKQQDGLLHTTCGTPAYVAPEVINRKGYDGSKADIWSCGVILYVLLAGYLPFHDTNLMEMYRKIGKAEFRFPNWFAPEVRKLLSKILDPKPSTRISMVKIMENSWFCKGLEPKSLIVETNREEHAFADCDAVFNVDESNSAVTQSKQESAKPCNLNAFDIISYSAGFDLSGLFEEKEKKKEVRFTANKPSSIIISKLEDIGKRLKLKMKKKDAGMLKFEGSKAGRKGALGIDAEIFEITPCFHLVEMKKSSGDTLEYQTVLNQEIRPALKDIVWTWQGEQQQQQQQQQILKQQQEQQELQPSHVCALQAASPYATS

>MeCIPK19

MAARIQANANSNQALLLGRYEIGKLLGHGTFAKVYLARNVKTNESVAIKVIDKEKILKGGLIAHIKREISILRRVRHPNIVQLFEVMATKAKIYFVMEYVRGGELFNKVAKGRLKEEVARKYFQQLISAVSFCHARGVFHRDLKPENLLLDENGDLKVSDFGLSAVSDQIRQDGLFHTFCGTPAYVAPEVLARKGYDAAKVDIWSCGVILFVLMAGYLPFNDQNIMAMYKKIYKGEFRCPRWFSPELVRLLWRLLDTNPETRGTMPQIMENKWFKKGFKHIKFFIEDDKVFSFETEGQLDDADSSSDQSLSESEPEMETRRRITSLPRPASLNAFDIISFSPGFNLSGLFEEGGEEGARIVSGAPVSKIISKLEEIAKLVSFTVRKKDYRVSLEGSREGAKGPLTIAAEIFELTPKLVVVEVKKKGGDQGEYEEFCSKELKPGLQMLTQEEPEIVATASSQSLPEHSHLFSATSDTE

>MeCIPK20

MEKKGAILMNRYELGRLLGQGTFAKVYHARNLQSGQSVAIKIINKEKILKGGLIDQIKREISVMRLVRHPNIVQLYEVMASKNKIYFAMEYVRGGELFNKVAKGKLNEDVARKYFQQLIGAVDFCHSRGVYHRDIKPENLLLDENGNLKVSDFGLSALWDSRKQDGLLHTTCGTPAYVAPEVITKNGYDGAKSDIWSCGVVLFVLLAGYLPFNHQNIMELYRKIIKGEFKCPTWINSGARKLISSILHPNPNSRIAIDDITSNSWFKKGYKQIEAPPSPQGQARCTLLKDVHEAFNSSLPSENKNSSQKEVIAADTRSPVRPTNYNAFDIISRSKGFDLSGLFEEDRYQRLEARFTSKKPASTIVSKFQEIATMESFNFNKKDGTVKLLGCKEGRKGQLEIDAEIFEVTPSFFVVELTKASGDTIEYKNFCNQELRPSLKDIVWAWQGNDQQQS

>MeCIPK21

MGFVNNIGKYHLGKTIGEGTFAKVKLAVDSNNASYVAVKIIDKQMVVESNLKYQVVREIKTMKILNHPNIVRIHEVIGTKRKIYIVMEYVSGGQLSDKLSYSQKLKESEARKMFQQLIDAVDYCHNRGVFHRDLKPENLLLDSKGNMKVSDFGLSALRKHGDVLTTACGSPCYVAPELLANKGYDGAAADVWSCGVILFELLSGYLPFDDRNLVVLYKKICAAEYTFPLWFTESQKKLISRILDPNPKRRITIAEMIEDEWFQTDYVPSFGYECDEKIYLDDVNAAFDVIEDDAAETKMPKPSSFINAFKLIAMSHDLDLSGLFQEQEDKKETTRLGSKHTVNETIKKIEAAALDLSLTVERMNNFRLKMHPKQKMTRCTRSCFDLSAEVIEVAPTNCVVEISKSAGELRLFSEFCKSLSTTLTEKSNTSFQVLDSEAPSIIDNNNQETIRSEKQIDREKNELGGYSSS

>MeCIPK22

MENKGNVLMHKYEMGRLLGQGTFAKVHYARDIRTGTSVAIKIIDKEKIMKVGLMDQIKREISVMKLVRHPNIIQLYEVIATKTKIYYVLEYVKGGELFNKVAKSKLKEDVARKYFQQLISAVDFCHSRGVYHRDLKPENLLLDEDGNLKVSDFGLSALAECTRQDGLLHTTCGSPAYVAPEVIKRKGYDGPKADIWSCGVILYVLLAGYLPFYDSNLMEMYRRIAKADFKSPDWFAPEACRLLSKILDPNPRTRISIAEIIESPWYWYHRDLESQSPMVETDTKGPAYLDCDAVNESSCAVTESKQELCYLNAFDIISYS

AGFDLSGLFEEKEKKKEMRFTAKKTASIIISKLEDTAKRLRLKIKKKDAGLLKFEGSNEGRKGALGIDAQIFEITPYFHLVEMKKSSGDTIEYQALLKQEIRPALKDVIWTWQGEQQQQLLKQEEQQEL

>MeCIPK23

MASRGSAATGSRTRVGRYELGRTIGEGSFAKVKFARNTETGENVAIKILDKEKVLKHKMIGQIKREISTMKLIRHPNVIRMYEVMASKTKIYIVLEFVTGGELFDKIASKGRLKEDEARKYFQQLINAVDYCHSRGVYHRDLKPENLLLDASGVLKVSDFGLSALPQQVRDDGLLHTTCGTPNYVAPEVINNKGYDGAKADLWSCGVILFVLMAGYLPFEESNLVALYKKIFKADFTCPPWFSSSAKKLIKRILDPNPSTRITIAEVIENEWFKKGYKPPAFEQAEVSLDDVNSIFNESGDCQNLVVEKRESPIGPVAPLTMNAFELISTSQGLNLSSLFEKQMGLVKRETRFTSKRSANEIISKIEEAATPLGFEVKKNNFKMKLQGEKTGRKGHLSIATEIFEVAPSLYMVELRKSGGDTLEFHKFYKNLSAGLKDIVWRTIDEEKEEGQPEISGSSVVPA

>MeCIPK24

MKKVTRKVGKYEVGRTIGEGTFAKVKFAQNMETGGSVAMKVMAKSTILKHRMVDQIKREISIMKIVRHPNIVMLHEVLASRTKIYIILEFVTGGELFDKIVHQGRLSENEARRYFQQLIDAVAHCHSKGVYHRDLKPENLLLDSVGNLKVSDFGLSALPQEGVGLLHTTCGTPNYVAPEVLSHQGYDGAAADVWSCGVILYVLMAGYLPFDEADLPTLYRKINAAEYTCPFWFSPGAKALIDKILDPNPKTRIRIEGIRKNPWFAKQYVPVKHSEEGEVNLDDVRAVFDDIEDQYVAEQSENSEGGPLIMNAFEMITLSQGLNLSALFDRRQDYVKRQTRFVSRKPAKVIISTIEAVAESMSLKVHTRNYKTRLEGISANKAGQFAVVLEVFEVAPSLFMVDVRKASGDTLEYHKFYKNFCAKLESIIWKPTDGVSASLLCGCEDKE

>MeCIPK25

MEERHVLFDKYEMGRLLGMGTFAKVYYGKNLATGESVAIKVINKDQVKKEGMMEQIKREISVMRLVRHPNIVELKEVLATKSKIFFVMEYVRGGELFAKVAKGKLKEDVARKYFQQLTSAVDFCHSRGVCHRDLKPENLLLDENGDLKISDFGLSALPEHLRIDGLLHTQCGTPAYVAPEVLRKKGYDGSRADIWSCGVILYVLLAGFLPFRDENVMKMYRQIFKAEFECPPWISTEAKRLISRLLVSDPERRITIPAITRVPWFRKGFTRPLAFSFQAISNPEKTEEEEDEALSAVMAKVSSPKFFNAFEFISSMSSGFDLSNLFENKKKTGSMFTSKFSASAIMNKIEAVAKGLNFKVSKVKDFKMRLQCPLEGRKGRLSITAEVFEVALDVAVVEFSKSAGDTLEYAKFCDEDVRPALKDIVWTWQGDTVCSRNDSGENENQTSL

>MeCIPK26

MSQPKIKRRVGKYEVGRTIGEGTFAKVKFARNSETGDPVALKILDKEKVLKHKMAEQIKREVATMKLIKH

PNVVRLYEVMGSKTKIFIVLEFVTGGELFDKIVNHGRMREDEARRYFQQLINAVDYCHSRGVYHRDLKPE

NLLLDANGNLKVSDFGLSALSQQVRDDGLLHTTCGTPNYVAPEVLNDRGYDGATADMWSCGVILFVLLAG

YLPFDDPNLMNLYKKISAAEFTCPPWLSFGAMKLITRILDPNPMTRITIPEILEDEWFKKDYKPPVFEEK

EDTNLDDVEAVFKDSEEHHVTEKKEEQPTAMNAFELISMSKGLNLGNLFDAEQGFKRETRFTSKRPANEI

IHKIEEAAKPLGFDVHKKNYKMRLENMKAGRKGNLNVATEIFQVAPSLHMVEVRKAKGDTLEFHKFYKNL

STCLDDVVWKSEDDMQEMK

>OsCIPK1

MVNGEAEAECTRASLLGRYEIGRTLGEGNFGKVKYARHLATGAHFAIKILDRNKILSLRFDDQIRREIGT

LKLLKHPNVVRLHEVAASKTKIYMVLEYVNGGELFDKIAVKGKLSEHEGRRLFQQLIDAVSYCHDKGVYH

RDLKPENVLVDRRGNIKISDFGLSALPQHLGNDGLLHTTCGSPNYIAPEVLQNRGYDGSLSDIWSCGVIL

YVMLVGYLPFDDRNLVVLYQKIFKGDTQIPKWLSPSARDLLRRILEPNPMKRINIAGIKEHEWFQKDYTP

VVPYDDDDDNYLDSVLPIKEQIDEAKQEKPTHINAFQLIGMASALDLSGFFEEEDASQRKIRFTSTHSPK

DLFDKIENVVTEMGFQVQRGNSKLKVMKNGRGSKNLRNPSSFLVCTEVVELGPSLYVVELKKSHGDPILY

RQLCERLSDELGVCKTEQIQRTESLEDDLESFDSGSSLPGF

>OsCIPK2

MAEQRGNMLMKKYEMGKLLGQGTFAKVYHARNTETSESVAIKMIDKEKVLKGGLMDQIKREISVMKLVRH

PNIVQLYEVMATKTKIYFVLEHVKGGELFNKVQRGRLKEDAARKYFQQLICAVDFCHSRGVYHRDLKPEN

LLLDENSNLKVSDFGLSALADCKRQDGLLHTTCGTPAYVAPEVINRRGYDGAKADIWSCGVILFVLLAGY

LPFHDKNLMDMYKKIGKAEFKCPSWFNTDVRRLLLRILDPNPSTRISMDKIMENPWFRKGLDAKLLRYNL

QPKDAIPVDMSTDFDSFNSAPTLEKKPSNLNAFDIISLSTGLDLSGMFEESDKKESKFTSTSTASTIISK

IEDIAKGLRLKLTKKDGGLLKMEGSKPGRKGVMGIDAEIFEVTPNFHLVELKKTNGDTLEYRKVLNQEMR

PALKDIVWAWQGEQPKQQQQPTC

>OsCIPK3

MYKAKRTAAQKVRRCLGKYELGRAIGQGTFAKVRFAKNMETGDHVAIKILDKAKVQKHRLVEQIRREICT

MKLIQHPNVVHLHEVMGSKTRIFIVLEYVMGGELHDIIATSGRLKEDEARKYFQQLINAVDYCHSRGVYH

RDLKLENLLLDTAGNIKVSDFGLSAISEQVKADGLLHTTCGTPNYVAPEVIEDKGYDGALADLWSCGVIL

FVLLAGYLPFEDENIVSLYNKISGAQFTCPSWFSAEAKRLIARILDPNPATRITTSQVLQDQWFKKGYES

PVFDDKYYPYFHDVYDAFGDSEEKHVKEAMEEQPTLMNAFELISLNKGLNLDNFFESDKKYKRETRFTSQ

CPPKEIINRIEEAANLLGFNIQKRNYRMRMENIKEGRKGHLNIATEVFQVAPSLHVVELKKAKGDTLEFQ

KFYQTLSTQLKDVVWELEDAAEDMS

>OsCIPK4

MAMAAMDARKKKSGGGGGEPLLGKYELGRMLGRGTFAKVYLARAVAGGEAVAVKVIDKAEVMGTAGMAPR

VLREVAAMRRLRHPHVLRLHEVLATRARIYLVMELATGGDLLSRLAALPRRRLPESAARRVFVQLVDALS

YCHARGVAHRDVKPQNVLLDGDGNLKVSDFGLAALPDTLRDDGRLHTACGTPAYAAPEVLRRRAYDGAKA

DAWSCGVILFVLLAGHLPFDDSNIADMCRKAHRREYELPRWVSQPARRLVSRLLDPNPDTRVAVESLAAH

HPWFKRSLSVDSQLDGLLNGEPERAVAFQAAPPPPLNAFDIISMSPGLDLSGLFGEHDKSLREKRFTTTA

SPEKTLEQLGLAGGKLGYVVVVGKKGVECLPLAGGRLSSGIAAMSVEMSEVAPPLLLVELRLEVAAGDVD

GGDGEVKGFGWEQLRMELGDVVRAWHSCEDLCEI

>OsCIPK5

MEKKASILMNRYELGRMLGQGTFAKVYHARNLASNQSVAIKVIDKEKVLRVGMIDQIKREISIMRLVRHP

NIVQLHEVMASKSKIYFAMEYVRGGELFSRVARGRLKEDAARKYFQQLIGAVDFCHSRGVYHRDLKPENL

LVDENGNLKVSDFGLSAFKECQKQDGLLHTTCGTPAYVAPEIINKRGYDGAKADIWSCGVILFVLLAGYL

PFHDSNLMEMYRKISKGDVKFPQWFTTDVRRLLSRLLDPNPNIRITVEKLVEHPWFKKGYKPAVMLSQPN

ESNNLKDVHTAFSADHKDNEGKAKEPASSLKPVSLNAFDIISLSKGFDLSGLFENDKEQKADSRFMTQKP

ASAIVSKLEQIAETESFKVKKQDGLVKLQGSKEGRKGQLAIDAEIFEVTPSFFVVEVKKSAGDTLEYEKF

CNKGLRPSLRDICWDGQSEHPSLAQSSTLTQSSKSISRHAI

>OsCIPK6

MMAAAAEEGEGKKGGGTVLQGRYEMGRVLGHGNFGRVHVARDLRTGRSVAVKVVAKEKVVRAGMMEQIKR

EIAVMKRVSHPNIVELHEVMATRSKIYLALELVRGGELFGRIVRLGRVREDAARHYFRQLVSAVDFCHSR

GVYHRDLKPENLLLDEAGNLKVVDFGLSALADHARADGLLHTLCGTPGYAAPEVLRDKGYDGAKADLWSC

GVILYVLLAGSLPFPDDNIVTLYRKAQRGDYRCPAWLSTDARRLIPRLLDPNPTTRISVAQLVETPWFKK

TSISRPVSIELPPAFADPAPAKEEAEKDEPETLNAFHLISLSEGFDLSPLFEGDSAKGRRDGGMLFATRE

PASGVISRLEGVAARGGGRMRVTKSGARGVRLEGAERGGAKGRLAVAADIFSVAPSVLVVDVKKDGGDTL

EYRSFCSEELRPALQDIVWGAAADPTPTAAV

>OsCIPK7

MAATKSKAAKKGAPLLGKYELGRLLGRGTFAKVYHARSLAPGADPVAVKVLDKPDLAAAGAGMATRVLRE

VAAMRRLRHPNVLRLHEVLATRSKVYLVMELAPGGDLLSRLASLPSRRLPEHAAQRVFLQLVSALIYCHA

RGVSHRDVKPQNVLLDAHGNLKVSDFGLAALPDSLRDDGRLHTACGTPAFAAPEVLRRKAYDGAKADAWS

CGVILFVLLAGHLPFDDSNIADMCRKAHRREYALPRWVSQPARRLVSRLLDPNPATRLAVAELATHPWFK

RSLSLDSQLGSLLGGQPERELAFQAPPPLNAFDIISMSPGLDLSGLFGESKRRREKRFVTTASPERTVER

LGQAGAKLGYFMVGKKGVERLPLGGLSGLVAMSMEMSEVSPSMMLVELRLEGGDDGDGDGGAEEFGWEEL

RAELGDDVVMAWHGCDGGKKDKEGILL

>OsCIPK8

MVGGGALRRVGKYEVGRTIGEGTFAKVKFAQNTESGESVAMKVVDRSSILKHKMADQIKREISIMKLVRH

PNVVRLHEVLASRKKIFIILEFITGGELFDKIIRHGRLNEADARRYFQQLIDGVDFCHSKGVYHRDLKPE

NLLLDSQGNLKISDFGLSAWPAQGGALLRTTCGTPNYVAPEVLSHKGYDGALADTWSCGVILYVLLAGYL

PFDEVDLTTLYGKIESAEYSFPAWFPNGAKSLIHRILDPNPDKRIRIEEIRNDEWFKKNYEPTREIESEE

VNLDDVNAAFDDPEEDADHTLDDEAGPLTLNAFDLIILSQGLNLAALFDRRQDYDKLQNRFLSRKPAKVI

MSSMEVVAQSMGYKTHIRNYKMRVEGLNANKTSHLAVMLEIFEVAPSIFMIELQRAAGDTSDYNKFINNY

CSKLDDIIWNFPIEKSKSRISRLSKR

>OsCIPK9

MAEAEAEAAGAGAGAGPARRTTRVGRYELGKTIGEGSFAKVKVARDTRTGDTLAIKVLDRNHVLRHKMVE

QIKREISTMKLIKHPNVVQLHEVMASKSKIYMVLEYVDGGELFDKIVNSGRLGEDEARRYFHQLINAVDY

CHSRGVYHRDLKPENLLLDSHGALKVSDFGLSAFAPQTKEDGLLHTACGTPNYVAPEVLADKGYDGMAAD

VWSCGIILFVLMAGYLPFDDPNLMTLYKLICKAKVSCPHWFSSGAKKFIKRILDPNPCTRITIAQILEDD

WFKKDYKPPLFEQGEDVSLDDVDAAFDCSEENLVAEKREKPESMNAFALISRSQGFNLGNLFEKEMMGMV

KRETSFTSQCTPQEIMSKIEEACGPLGFNVRKQNYKMKLKGDKTGRKGYLSVATEVFEVAPSLHMVELRK

TGGDTLEFHNFYNNFSSELKDIVWKSESDAKAAKKR

>OsCIPK10

MVEQKGNILMKRYEIGKLLGQGSFAKVYHGRNIKNSQSVAIKVIDKEKILKCELMDQIRREISVMNLVRH

PCIVQLYEVMATKTKIYFILEYVKGGELFNKVRRGRLKEEVARKYFQQLISAIDFCHSRGVYHRDLKPEN

LLLDENRNLKISDFGLSALAECKRQDGLLHTTCGTPAYVAPEVINRKGYDGAKADVWACGVILYVLLAGY

LPFQDKNVINMYKKICKAEFKWPSWFSSDIRKLLRRILDPNPATRISVSEIMEDPWFRVGLNSDLLNKTI

PTDKVDKVVHVDMDSTFGNLSNNINEGKQEAENLTSLNAFDIISLSSGFDLSAMFEDENSKEESKFTSTN

TATTITKKLEDVAKNLRLKFLKKNGGLLKMEGSKPGRKGVMSINAEIFQITPDFHLVEFTKINGDTLEYQ

KVKQEMRPALKDIVWAWQGEQPQPQSLNEQS

>OsCIPK11

MMDGRSILMGRYEVGKQLGQGTFAKVYYARNLTTGQAVAIKMINKDKVMKVGLMEQIKREISIMRLVKHP

NVLQLFEVMASKSKIYFVLEYAKGGELFNKIAKEGKLSEDSARRYFHQLINAVDYCHSRGVYHRDLKPEN

LLLDENENLKVSDFGLSALAESKRQDGLLHTTCGTPAYVAPEVLSRKGYDGAKADVWSCGVILFVLVAGY

LPFHDPNLIEMYRKICRADFRCPRYFSAELKDLIHKILDSDPSTRISIPRIKRSTWYRKPVEINAKNSEA

ATTNSISSGVATTSGSAECSTSEENQGSLSLPNLNAFDIISLSTGFNLSGFFEDTHGHQEERFTTRQPVT

TVLGKLKELAKRLKLKVKKKDNGVLRLAAPKEGKKGFLELDAEIFEVTPSFLLVELKKTNGDTMEYRKLV

KEDIRPALKDIVWVWQGDEHLNSQSILQGEQQQSPLPPELPQDQLQPSLPQQEKQDMPEPPLLPQVPQEE

VQTSIPAEQTKN

>OsCIPK12

MLMATVSPARREPTPQAVRASPMPSAAAALVRRGGGGSGGTVLGKYELGRVLGQGSFAKVYQARHLETDE

CVAIKVLDKEKAVKGGMVHLVKREINVLRRVRHPNIVQLFEVMASKTKIYFVMEYVRGGELFSRVSKGRL

REDTARRYFQQLVSAVDFCHARGVFHRDLKPENLLVDENGDLKVSDFGLAAGPDQFDPDGLLHTFCGTPA

YVAPEVLRRRGYDGAKADIWSCGVILFALMAGYLPFHDHNIMVLYRKIYNGEFRCPRWFSKDFTRLITRL

LDANPKTRITVPEIIESDWFKKGYKPVKFYIEDDKLYNLSDDVLNLEPADPVPPPLGLAPPVPPPPQGDD

PDGSGSESDSSVVSCPATLSTGESQRVRGSLPRPASLNAFDIISFSKGFNLSGLFEERGNEIRFVSGEPM

SDIVKKLEEIAKVKSFTVRRKDWRVSIEGTREGVKGPLTIGAEIFELTPSLVVVEVKRKAGDNEEYEDFC

NMELKPGMQHLVHQMLPAPNGTPVSEKVERSSSLQAPLTLKLIGTEGSMS

>OsCIPK13

MARMGISKGGSGKEAKKPPLLLGRFEVGKLLGQGNFAKVYHARNVATGEEVAIKVMEKEKIFKSGLTAHI

KREIAVLRRVRHPHIVQLYEVMATKLRIYFVMEYVRGGELFARVARGRLPEADARRYFQQLVSAVAFCHA

RGVFHRDIKPENLLVDDAGDLKVSDFGLSAVADGMRRDGLFHTFCGTPAYVAPEVLSRRGYDAAGADLWS

CGVVLFVLMAGYLPFQDRNLAGMYRKIHKGDFRCPKWFSPELIRLLRGVLVTNPQRRATAEGIMENEWFK

IGFRRFSFRVEDDRTFTCFELDDDAAVDAPTSPPDTPRTVDSGDVGAAPTRPRKAGSLTSCDSAPSLLEG

RFGLGGSSRRRSSLNAFDIISFSPGFDLSGLFDQDDGGGAGAGSIPEQQKHTARFVSAAPVEVIVATLEA

AAAAAGMAVREREDGSISMEGTREGEHGALAVAAEIYELTPELVVVEVRRKAGGAAEYEEFFRARLKPSL

RELVCDDRPCPEDSGELSRSL

>OsCIPK14

MESRGKILMERYELGRLLGKGTFGKVHYARNLESNQSVAIKMMDKQQVLKVGLSEQIRREITTMRLVAHK

NIVQLHEVMATRNKIYFVMEYVKGGELFEKVAKRGKLTEVVAHKYFQQLISAVDYCHSRGVYHRDLKPEN

LLLDENENLKVSDFGLSALSESKRQDGLLHTTCGTPAYVAPEVISKIGYDGAKSDIWSCGVILFVLVAGY

LPFQGPNLMEMYRKIQHGEFRCPGWFSRKLQKLLYKIMDPNPSTRISIQKIKESTWFRKGPEENRILKER

TLNENTTKNVALVLGVRRKKNAHEDVKPMSVTNLNAFEIISFSKGFDLSGMFIVKEWRNEARFTSDKSAS

TIISKLEDVAKALNLRVRKKDNGVVKMQGRKEGRNGVLQFDIEIFEVTTSYHIIEMKQTSGDSLEYRQLL

EEGIRPALKDIVLAWHGDE

>OsCIPK15

MESRGKILMERYELGRLLGKGTFGKVHYARNLESNQSVAIKMMDKQQILKVGLSEQIRREITTMRLVAHK

NIVQLHEVMATRNKIYFVMEYVKGGELFEKVAKRGKLTEVVAHKYFQQLISAVDYCHSRGVYHRDLKPEN

LLLDENENLKVSDFGLSALSESKRQDGLLHTTCGTPAYVAPEVISKIGYDGAKSDIWSCGVILFVLVAGY

LPFQGPNLMEMYRKIQHGEFRCPGWFSRKLQKLLYKIMDPNPSTRISIQKIKESTWFRKGPEENRILKER

TLNENTTKNVAPVLGVRRKKNAHEDVKPMSVTNLNAFEIISFSKGFDLSGMFIVKEWRNEARFTSDKSAS

TIISKLEDVAKALNLRVRKKDNGVVKMQGRKEGRNGVLQFDIEIFEVTTSYHIIEMKQTSGDSLEYRQLL

EEGIRPALKDIVLA

>OsCIPK16

MARRAREEEADQVERKLVLGRYELGRLLGQGTFAKVYYGRDLRSGESVAIKVIDKARLRRTEGMVEQLRR

EISIMRMVRHPNVVGIREVLASRARVFVVMEYARGGELFAKVARGRLTEEHARRYFQQLVAAVGFCHGRG

VAHRDLKPENLLLDEEGRLKVTDFGLAALPEQLRQDGLLHTQCGTPAYVAPEVLRKRGYDGARADLWSCG

VVLYVLLCGFLPFQHENYAKMYQKIFKAEYQVPPWVSGDARRLIVRLLVVDPAKRISIPEIMRTPWFKKG

FVPPVPTSPVSPKKWEEDDVLLDGGDSGAMSPRTCNAFQLISSMSSGFDLSGMFESEQKAATVFTSRAPA

ATVIQKLEAVGRSLGYSATRGKGWKLRLEATADGANGRLAVTVEALEVAADVAVVEFAHDAGDELEFNKF

CAVDVRPGLADIVWAWQGDRPAAPDVAAATVECSPA

>OsCIPK17

MVKGGREALLGGYEMGRTLGEGNFGKVKYARHLATGGHFAVKILDRGRVVSLRAGDQIRREIATLKLLRH

PHVVRLHEVAASKTKIYMVLEFVNGGELFERIAVKGKLSEKEGRRLFQQLIDGVSYCHDRGVYHRDLKPE

NVLVDQKGNIKISDFGLSALPQHLGNDGLLHTTCGSPNYIAPEVLQNKGYDGSLSDIWSCGVILYVMLIG

YLPFDDRNIVVLYQKIFKGDTQIPKWLSHSAQNLLRRILEPNPMKRIDMAGIKSHEWFQKDYIPVLPYDD

DDEDVQFGARLPAKEQINDEPGDKNSHQINAFQLIGMASSLDLSGFFEDEEVSQRRIRFTSTHPPKDAFD

KIESSATELGFQVQRGHSKLKLMRNCKGSKNPESFMVSAEVFELGPSVNVVELRKSNGDPALYRQLCERI

SSDMGARNTEQIFATASLEDDLQNSNAGTPLFAL

>OsCIPK18

MMELEKNGNILLRRYEIGKLLGQGTFAKVYHGRNIVTSQSVAIKVIDKDKIFKVGLMDQIKREISVMKLV

RHPNIVQLYEVMATKSKIYFVLEYVKGGELFNKVAKGRLKEDAARKYFQQLVSAVDFCHSRGVYHRDLKP

ENLLVDENGNLKITDFGLSALAESRRQDGLLHTTCGTPAYVAPEVISRKGYDGVKVDTWSCGVILFVLMA

GYLPFQDSNLMEMYRKIGKAEFKCPAWFSSDVRKLVSRILDPNPRSRMPITKIMETYWFKKGLDSKLILK

NVETNEPVTALADVNVVFSSMGSSSSKKTEEKQDAGKLTNLNAFDIISLSEGFDLSGLFEETDKKKEARF

TSSQSASAIISKLEDVASCSKLTVKKKEGGVLKMEGASEGRKGVLAIDAEIFEVTPSFHLVEIKKNNGDT

LEYQHLWKEDMKPALKDIVWAWQGERQDQQPEDHGQP

>OsCIPK19

MAATPPSSQHRRPLSSSASAASLAGKPRGGGLLLGRYELGRLLGHGTFAKVYQARSADSGEPVAIKVLDK

EKAMRHGLVPHIKREIAILRRVRHPNIVRLFEVMATKSKIYFVMELVRGGELFGRVAKGRLKEDTARRYF

QQLVSAVGFCHARGVFHRDLKPENLLVDEHGDLKVSDFGLSAVADQFHPDGLLHTFCGTPSYVAPEVLAR

RGYDGAKADIWSCGIILFVLMAGYLPFHDQNLMAMYRKIYRGEFRCPRWFSKDLSSLLNRILDTNPETRI

TVKEVMESRWFQKGFRPVRFYVEDDQVHSLADGDNDMPELEPSEPPPPPPFPPPPPQQDDDGEESGWESD

SSVASCPATLSSEERRQRPLGSLTRPASLNAFDIISFSKGFDLSGLFEERGSEVRFISAEPMQTIITKLE

EIAKVKSFFVRRKDWRVSIEGTREGLKGPLTIGAEIFELTPSLVVVEVKKKAGDKEEYDDFCNRELKPGM

QHLVHHMGSVPNIPSDTE

>OsCIPK20

MPEKGTVVMSRYELGRSLGHGTFSKVYQARSLVSGETVAVKVIDKEKALRAGAGMVDQIEREVAVMRLVG

RHPNVVRLHEVMASRSKIYFVMELVRGGELLARLVAGGGRLGEDAARRYFHQLVAAVDFCHSRGVYHRDL

KPENLLVDDDGSGGGGNLKVTDFGLSALSASRRHDGLLHTTCGTPSYVAPEIIGDKGYDGATADVWSCGV

ILFLLLAGYLPFFDSNLMEMYKKITNGEFKVPDWFTPDARSLISRLLDPNPTTRITIDELVKHPWFKKGH

TKRPASSNTMKLNEEEKPANAAMNMKPASLNAFDIISLSQGFDLSGMFCCHGHSSRTQDQLFVTGKPATA

IVSRLEEIAETEHFTVKKKQKKRQEEDGMAVKLQGWKEGRKGQLAIDAEIFEVSPSCYVVEVKKTAGDTL

EYQAFCNRDLRPSLNDICWTSPATAASEKNQLPAVSEVSPLSSPRN

>OsCIPK21

MQKTSMNPVTDPVAAATGRVAIRQLPIKTQPNSQSLTPFLQLPPKPPPNLLFSSPLASVELRHRARARRR

RRPSHPRRAPAMRMGKYEMGRALGEGHFGKVKLARHADTGAAFAIKILDRQRILAMKIDEQIKREIATLK

LLKHPNVVRLHEVSASKTKIYMVLEYVNGGELFDKIALKGKLSEKEGRKLFQQLMDAVSYCHEKGVYHRD

LKPENVLVDAKGNIKVSDFGLSALPQNQRKDGLLHTTCGSPNYIAPEVLLNRGYDGSLSDIWSCGVILYV

MLTGNLPFDDQNTVVLYQKILKGDARIPKWLSPGAQDILRKILDPNPITRLDITGIRAHEWFRQDYTPAM

PFDDDDDNNISDGNLHMTENQDIETSPAISQINAFQLIGMSSCLDLSGFFEKEDVSERKIRFVSNYSPTS

LFEKIESTVTEKGFQVQKNSGKLKVIQVCKEPANPRGHGNLLISAEVFEINESLYVVELKRSSGDCSLYR

QLCASLSEDLGICKRQQLLKKDSMRQDLCRYNSSF

>OsCIPK22

MPPAGDDESPAATGDGYSKKVLQGRYELGRVLGQGASSKVYRARDARTGAHVAVKAIRKQQQPHHHPSCR

SPEAAAAARRCVEVEREVAALRRVRGHPHVVALLDVLATRSTVYLVLELASGGSVLSALDSRGGGHYDEP

AARRLFAQLASAVAHAHSLGVFHRDIKPENLLLDERGDLRLTDFGLSAFADADQHLGATDGLAATHCGSP

AYVAPEILLKRRYDASKADVWSCGVVLFVLTAGYLPFNDGNLMAMYRKICAAKFRCPKWCSQELRSLIGR

MLDPEPDTRIKIGEIFDHPWLQQDGSSSSFGMIQAASSHSKPEVEKWEAELEQAMELNAFDIIGFASGCD

LSGLIGPLPDRVRFVLPGGDSKSVLDKVEKLGREEGLVVRRKEEEWCGGVHVEATSGKFTAYVRVNLLPK

KILMIEAERVIGSEIPKFWHQLQIGNLLVRK

>OsCIPK23

MSVSGGRTRVGRYELGRTLGEGTFAKVKFARNADSGENVAIKILDKDKVLKHKMIAQIKREISTMKLIRH

PNVIRMHEVMASKTKIYIVMELVTGGELFDKIASRGRLKEDDARKYFQQLINAVDYCHSRGVYHRDLKPE

NLLLDASGTLKVSDFGLSALSQQVREDGLLHTTCGTPNYVAPEVINNKGYDGAKADLWSCGVILFVLMAG

YLPFEDSNLMSLYKKIFKADFSCPSWFSTSAKKLIKKILDPNPSTRITIAELINNEWFKKGYQPPRFETA

DVNLDDINSIFNESGDQTQLVVERREERPSVMNAFELISTSQGLNLGTLFEKQSQGSVKRETRFASRLPA

NEILSKIEAAAGPMGFNVQKRNYKLKLQGENPGRKGQLAIATEVFEVTPSLYMVELRKSNGDTLEFHKFY

HNISNGLKDVMWKPESSIIAGDEIQHRRSP

>OsCIPK24

MGGEEGMAAGRKKRVGRYEVGRTIGQGTFAKVKFAVDADTGAAVAMKVLDKDTILNHRMLHQIKREISIM

KIVRHPNIVRLNEVLAGKTKIYIILELITGGELFDKIARQGKLRENEARKYFQQLIDAINYCHSKGVYHR

DLKPENLLLDSRGNLKVSDFGLSTLAQKGVGLLHTTCGTPNYVAPEVLSNNGYDGSAADVWSCGVILYVL

MAGYLPFEEDDLPTLYDKITAGQFSCPYWFSPGATSLIHRILDPNPKTRITIEQIREDTWFKKTYVAIKR

GEDENVDLDDVQAVFDNIEDKYVSEQVTHNDGGPLVMNAFEMITLSQGLDLSALFDRQQEFVKRQTRFVS

RKPAKTIVATIEVVAETMGLKVHSQNYKLRLEGVSSNRMSPFAVVLQVFEVAPSLFMVDVRKVAGDTLEY

HRFYKNLCNKMESIIWRPIEVSAKSALLRTATC

>OsCIPK25

MFRSMGTGTGTKPPAMTTERYEFGPLVGEGNFAKVYLGRHRATGEEVAIKVMDKEKLVRLGATELIKREI

AVMQRLRHPNVVRIHEVMANKRRICVVMEYVRGGALYRYFRRGPSGGAAGLREHEARRFFQQLVSAVAYC

HSRGVFHRDIKLDNLLVDEQGNLKVADFGLSALADMERREAHLQTVCGTPLFLAPEVFKRRGYDGAKADV

WACGVVLYVLLTGRKPFPDEHVSRLYRLIGQNQFQCPPSFSPDLARLVRRLLQPDPDRRITIPEIMEMRW

FKRGFKEVTYYIDSNDRLRSLDGLDGEPELYDSDTDTIESSSSSESPTPVAGTPRGMHTSVSAPALSELD

RMEDSASLPLPLPLPPRPRMPRPKSLNAFDIIASSPSFDLSGLFEERGERMRFVSGAPVADIIAKLQEIA

GMVSFTARTKDCQVSIEATRNGQKGALAISAKVFELTRELVMVQVCKKAGDTAEYRRFCDNELKAGLRGL

VVDALPPPVEGGGHGGAAAAAEAE

>OsCIPK26

MDDRRTILMDRYEIGRQLGQGNFAKVYYARNLTSGQAVAIKMIDKEKVTRVGLMVQIKREISIMRLVKHP

NILQLFEVMASKSKIYFVLEYAKGGELFKKISKGKFSEDVARRYFHQLISGIDYCHSRGVYHRDLKPENL

LLDENESLKVSDFGLSALSESKRHDGLLHTTCGTPAYVAPEVLSRRGYDGAKADIWSCGVILFVLVSGYL

PFHDTNLIEMYRKIAKAEYKCPRSFSAELKDLLYKILDPDPSTRISIPKIKRSAWYRKSSDVNALKSKHE

TGDKVYKGEATTSDTTECSIFEGNRASSRDKVYTNGEATTSDSPECSNSDGKQASLSLPNLNAFDIISLS

TGFDLSNLFEERYGRREERFTTRQPAAAIFAKLNELARRFKLKIKKKENGVLRLVAPKEGIKGLLELDAE

VFELAPSFHLVEFKKSNGDTIEYQKLMKEDIRPALKDIVWAWQGGQHQQPEQSMQGMQGEQQPSRLPSQQ

PQG

>OsCIPK27

MEGKGVLEGRYEMGRVLGHGNFGRVHAARDVRTGRAVAMKVVSKDKVERAGMAEQIKREIAVMKMVSHPS

VVELHEVMATRTKVYLALELVRGGELFDRIARHGRVGEGVARRYFRQLVSAVDFCHGRGVYHRDLKPENL

LLDEAGNLKVADFGLSALACHARPDGLLHTACGTPAYVAPEVLAGNGYDGAKADLWSCGVILYVLLAGAL

PFQDDNLVCMYRKMRRGDFCCPPWVTTDARKLIKSLLDPNPGTRITVAGLLETPWFRKTAPVPRPIIADP

AAAPVDTRGNAGDDKDEPPEVLNAFHLISLSEGFDLSPLFEHDPAASPGRATARAGGTRFATREAASGVV

ARLEALAMGGARVAPSLLMVDVKKDGGDAMEYRPFFSEELRPALKDIVWSPAAT

>OsCIPK28

MEERSVLMERYVIGRQLGQGTFGKVYYARNLSSGQSVAIKMIDKEKILKVGLMEQIKREISIMRLVRHPN

VLQLFEVMATKSNIYFALEYAKGGELFHKMARAKLNEESARNYFQQLISAMDYCHSRGVYHRDLKPENLL

LDENETLKVSDFGLSALAESRRQDGLLHTACGTPAYVAPEVLSRKGYSGSKADVWSCGVILFVLVANYLP

FHDRNIIQMYRKIAKAEYRCPRHFSAELKELLYGILDPDPSTRMSISRIKRSAWYRKPIAISALNNETGK

KSCTSEAPFSGPTICISSERNQEPPNLHNLNAFDIISLSTGFDLSGLFGERYGRRESLFTSRKPAAAVLV

KLKELAKALNLKVTKTDNGVLKLATTKEGRKGRLELDAEVSEVAPFLLVELKKTNGDTLEYQRMMKEDIR

PSLKDIIWTWQGDQQ

>OsCIPK29

MPPSTGSVPPAASTPAAGDEATAAGRVLLGRYELGGLLGRGASAKVYLARDLLTGRDVAIKSFPNPRHGG

GLRGGEEDVLLRPAPIEREAAILPRLRHRHVMRLREILATRKKVHFVLDLAAGGELFSLLDASGRMTEDL

ARHYFRQLISAVRYCHSRGVYHRDIKPENLLLDDAGDLKVADFGLGAVADGALHHTLCGTPAYVAPEILS

RKGYNPAKVDIWSCGVVLFVLAAGYLPFNDASLVNMYRKIYAGKFRCPAWFSPELRCLVRRILDPNPATR

IDTEEIITHPWFRQDASHFAMAQLMQHGHDEEAKFKTEFKEDDMARDMTAFDILACSPGSDLSGLFGAEP

GKERVFVGEPAAAVLSRVEEAGKKEGYMVTREGKKGTGPVYVKGENGGIVAKVCVFKIADAVSVVEVVKG

YGAEAARFWKARLEPAMKPPAAI

>OsCIPK30

MAMETTSQDSQVIMGRYKLGRLLGRGTFAKVYKAYKLATGEAVAIKVFDKEAVQRSGTVEQVKREVDVMR

RVHHRHVIRLHEVMATRSRIYFVMEYASGGELFTRLSRSPRFPEPVARRYFQQLITAVEFCHSRGVYHRD

LKPENLLLDARGDLKVTDFGLSALDGGLRGDGLLHTTCGTPAYVAPEVLLKRGYDGAKADIWSCGVILFV

LLAGYLPFNETNLVILYRNITESNYRCPPWFSVEARKLLARLLDPNPKTRITISKIMDRPWFQQATCPLG

DMSLVASAPSVLLARKEASQQHDDEEDDGFAREKKKRSNVIMSSPVIDVRPSSMNAFDIISRSRGLDLSK

MFDAEERRSEARFSTRETTTAIVSKLEEIAEAGRFSFKLKEKGRVELEGSQDGRKGALAIEAEIFKVAPE

VHVVEVRKTGGDSPDFRDFYKQELKPSLGDMVWAWQGGDSPPLVPAAGRRPITKRS

>OsCIPK31

MYRAKRAALSPKVKRRVGKYELGRTIGEGTFAKVRFAKNTENDEPVAIKILDKEKVQKHRLVEQIRREIC

TMKLVKHPNVVRLFEVMGSKARIFIVLEYVTGGELFEIIATNGRLKEEEARKYFQQLINAVDYCHSRGVY

HRDLKLENLLLDASGNLKVSDFGLSALTEQVKADGLLHTTCGTPNYVAPEVIEDRGYDGAAADIWSCGVI

LYVLLAGFLPFEDDNIIALYKKISEAQFTCPSWFSTGAKKLITRILDPNPTTRITISQILEDPWFKKGYK

PPVFDEKYETSFDDVDAAFGDSEDRHVKEETEDQPTSMNAFELISLNQALNLDNLFEAKKEYKRETRFTS

QCPPKEIITKIEEAAKPLGFDIQKKNYKMRMENLKAGRKGNLNVATEVFQVAPSLHVVELKKAKGDTLEF

QKFYRTLSTQLKDVVWKCDGEVEGNGAAA

>OsCIPK32

MSTTKVKRRVGKYELGRTIGEGTFAKVKFARDTETGDPVAIKILDKEKVLKHKMVEQIKREISTMKLIKH

PNVVRIYEVMGSKTKIYIVLEYVTGGELFDTIVNHGRMREDEARRYFQQLINAVDYCHSRGVYHRDLKPE

NLLLDSYGNLKVSDFGLSALSQQIKDDGLLHTTCGTPNYVAPEVLEDQGYDGAMADLWSCGVILFVLLAG

YLPFEDSNLMTLYKKISNAEFTFPPWTSFPAKRLLTRILDPNPMTRVTIPEILEDEWFKKGYKRPEFDEK

YDTTLDDVYAVFNDSEEHHVTEKKEEPEALNAFELISMSAGLNLGNLFDSEQEFKRETRFTSKCPPKEIV

RKIEEAAKPLGFDVQKKNYKLRLEKVKAGRKGNLNVATEILQVAPSLHMVEVRKAKGDTLEFHKFYKNLS

RTLKDVVWKSDDLQNQLS

>OsCIPK33

MSTTKVKRRVGKYELGRTIGEGTFAKVKFARDTETGDPVAIKILDKEKVLKHKMVEQIKREISTMKLIKH

PNVVRIYEVMGSKTNIYIVLEYVTGGELFDTIVNHGRMREDEARRYFQQLINAVDYCHSRGVYHRDLKPE

NLLLDSYGNLKVSDFGLSALSQQIKDDGLLHTTCGTPNYVAPEVLEDQGYDGAMADLWSCGVILFVLLAG

YLPFEDSNLMTLYKKISNAEFTFPPWTSFPAKRLLTRILDPNPMTRITIPEILEDEWFKKGYKRPEFDEK

YDTTLDDVDAVFNDSEEHHVTEKKEEPEALNAFELISMSAGLNLGNLFDSEQEFKRETRFTSKCPPKEIV

RKIEEAAKPLGFDVQKKNYKICSPCLTTICMNIPFKLRLEKVKAGRKGNLNVATEILQVAPSLHMVEVRK

AKGDTLEFHKFYKNLSRTLKDVVWKSDDLQNQLS

>OsCIPK34

MGFVESIGRYRVGRTIGAGTFAKVRLAVDADTGATVAVKVIDKRMVIRNNLMYQVKREITAMKLLNHPNIVKIYEVIATKTKIC

LVMEYVSGGQLSDKLSYLKRLDEKEAKKYFYQLIDAVDYCHRRGVYHRDLKPENLLVDNQGNLKVSDFGLSVLKKPGQFLSTSCGSPCYVAPEVIQHKSY

DGAAADVWSCGVILFELLAGYLPFQDCSLTNLYRRISRAQFVFPQWLSVPQKKIIIRILDPSPITRAKISDIFDDKWLQDHCNPSARIENDDDCDVIEEA

STDSDSSHNTEVKETEEMTAETDRFINAFQLIARCSDLDLSGLFQEQKTKLASPHPVQETFDKIKVAAKDVSMAVKRMNSSLVEIQDSKLLPRSNLDLTL

SAEVIKVTPAHCVVEVSKSTGDLRSYKEFCRSLSSLLNGGQLSASSSDMECD

>PtCIPK1

MVRKEEEKQKKEKGMQLGKYELGRTLGEGNFGKVKLAKNIETGQPFAVKILEKNRIIDLKITDQIKREIATLKLLKHPNVVRLHEVLASKSKIYMVLEYVTGGELFDRIASKGKLPEAEGRKMFQQLIDGVSYCHSKGVFHRDLKLENVLVDAKGNIKISDFGLSALPQHFRDDGLLHTTCGSPNYVAPEILSNRGYDGATSDIWSCGVILYVILTGYLPFDDRNLAVLYQKIFKGDAQVPKWLSPGAKNMIKRILDPNPVTRITTADIKADEWFKLDYTPMDSAEEQDDVYIDDAAFSIQEAPSEDGSPKSPNLINAFQLIGMSSCLDLSGLFEKEDVSERKIRFTSNHSMSDLLEKIEDIVTEMGFRVQKRNGRFQVIQEHRGKKSSGSLSVATEVFEISPSLYVVELRKSYGDSSAYRQLCTKLSNDLGVPSSPGLLTTEGTSRPLEHMPTTRLPFLKWRNLSLDARTLDR

>PtCIPK2

MENKPSVLTQKYEVGRLLGQGTFAKVYFARNIKTNQSVAIKLIEKEKVLRVGLVDQIKREIYVMRLVRHPNIIKLYEVLATKSKIYFVMEYAKGGELFNKVSKGRLKEDVAWKYFQQLINAVDFCHSRDVYHRDIKPENLLLDENENLKISDFGLSALAESKRQDGLLHTTCGTPAYVAPEVIKRKGYDGAKADIWSCGVVLFVLLAGYLPFHDTNLMEMYRKIDKAEFKCPNWFPTDARKLLRKILDPNPNTRISIAEIKESSWFRKGLPRQSKTETVGREAAALGRNGSGPSENSSVACEAKQESAKPPYLNAFDIISLSAGFDLSGLFDDKYPNREARFTSAQPASVVISKLEDISKCLKLKVMKKDAGLLKMEGMKEGRKGLLAIDAEIFELTPNFHLVELKKTNGDTLEYQKMLNEDIRPALKDIVWLWQGSKNHNSSSCSCSNDIDQTISNSLLEIFS

>PtCIPK3

MSQPKIKRRVGKYEVGRTIGEGTFAKVKFARNSETGEPVALKILDKEKVLKHKMAEQIKREIETMKLIKHPNVVRLYEVMGSKTKIFIVLEFVTGGELFDKIVNHGRMREDEARRYFQQLINVVDYCHSRGVFHRDLKPENLLLDAYGNLKVSDFGLSALSQQVRDDGLLHTTCGTPNYVAPEVLNDRGYDGTTADLWSCGVILFVLLAGYLPFDDSNVMNLYKKISAAEFTCPPWLSFGAMKLITRILDPNPMTRITIPEILVDEWFKKGYKPPVFEEKEDTNLDDVEAVFKDSEEHHVTEKKEEEQPTAMNAFELISMSKGLNLGNLFDGEQEFKRETRFTSRCPANEIIHKIEEAAKPLGFDVHKKNYKMRLENVKAGRKGNLNVATEIFQVAPSLHMVEVRKAKGDTLEFHKFYKSLSTCLDDVVWKTEEDMQETK

>PtCIPK4

MSQPKIKRRVGKYEMGRTIGEGTFAKVKFARNSETGEPVALKILDKEKVLKHKMAEQIKREVATMKRIKHPNVVRLYEVMGSKTKIFIVLEFVTGGELFDKIVNHGRMREDEARGYFHQLINAVDYCHSRGVFHRDLKPENLLLDAYGNLKVSDFGLSALSQQVRDDGLLHTTCGTPNYVAPEVLNDRGYDGAPADLWSCGVILFVLLAGYLPFDDSNLMNLYKKISAAEFTCPPWLSFGAMKLITRILDPNPMTRITVSEILVDEWFKKGYKTPVFEEKEDTNLDDVEAVFKDSEENHVTEKKEEEQPTAMNAFELISMSRGLNLGNLFDGEQEFKRETRFTSKCPANEIIHKIEEAAKPLGFDVHKKNYKMRLENVKAGRKGNLNVATEIFQMAPSLHMVEVRKAKGDTLEFHKFYKSLSTCLDDVVWKTEEDMQETK

>PtCIPK5

MEPPPPSTLQTTTSIPTTLLNKYELGRLLGRGSFAKVYAARSLSDKTQLAAIKIIDKTKTDAAMEPRIISEISAMHRLQHHPTILKIHEVMATKTKIYLVMELASGGDLYSKIRKIGKLKESAARRYFQQLVSALHFCHQNGVSHRDIKPHNLLLDGKGNLKISDFGLSALKNGGGDGVFLLQTACGTPAFTAPEVMSRQGYDGAKADAWSCGVILFFLLSACLPFDDSNLAFLYKRAHKGEYQVPSCISKPVKSIINQLLDPNPNTRMSMEALMKHSWFLKKFELPTQSSVFELDYKKYCKFDQKSAVASITAFDIISLSSGLDLSRLFEVKNRKERRFTSSDTVERVTERVREVGGRLGYRVVEGKGGSAIGLGKGRVGVLFEVFEIAEKLLVVEVMVVEGGEVEFEEVHWGELKDELEDVVLQWHNDVM

>PtCIPK6

MEERHVIFGKYEMGRLLGKGTFAKVYYGKHLVTGESVAIKVISKDQVKKEGMMEQIQREISVMRLVRHPNIVELKEVMATKTKIFFIMEYVRGGELFAKVAKGRLKEEAARKYFQQLISAIDYCHSRGVYHRDLKPENMLLDEDENLKISDFGLSALPEQFRQDGLLHTQCGTPAYVAPEVLRKKGYDGSKADIWSCGVILYVLLAGFLPFQDENVMKMYRKVFKAEYQFPPWFSTDSKRLISRLLVADPEKRITIPAIMRNHWFLKGFSRPVAFSIQESSMDQTGQEQDLDSCSVVNTKVSSPEFFNAFEFISSMSSGFDLSSLFETKRKSGSMFTSKFSASAIMEKIEGVAKGLSYKVAKVKDFKVTLQGPCEGRKGKLAVTAEVFEVAPEVAVVEFSKSSGDTLEYTKFCEEDVRPALKDIVWTWQGDNVCNKDNNNSHVEDREIQML

>PtCIPK7

MAETTSDGHPTLLHGKYELGRLLGHGTFAKVYHARNLQSGKSVAMKVVGKEKVIKVGMMEQIKREISVMKMVKHPNIVELHEVMASKSKIYFAMELVRGGELFSKIEKGRLREDVARVYFQQLISAIDFCHSRGVYHRDLKPENLLLDENGKLKVTDFGLSAFTEHLKQDGLLHTTCGTPAYVAPEVIGKQGYDGAKADLWSCGVILYVLLAGFLPFQDDNIVAMYRKIYRGDFKCPPWFSSEARRLITKLLDPNPSTRITISKVMDSTWFKKSVPKTVKSKEEMEFEAFNGDEDANGDKSKQLETLNAFHIISLSQGFDLSPLFEERKREEKEELRFATTRPASSVISRLEEVGKAGNFSVKKSDSKVRLQGQERGRKGKLAIAADIFAVTPSFLVVEVKKDNGDTLEFNQFCSKALRPALKDIVWISPAENSTLARI

>PtCIPK8

MAETTSDGHPTLLHGKYELGRLLGHGTFAKVYHARNLQSGKSVAMKVVGKEKVIKVGMMEQIKREISVMKMVKHPNIVELHEVMASKSKIYFAMELVRGGELFSKIEKGRLREDVARVYFQQLISAIDFCHSRGVYHRDLKPENLLLDENGKLKVTDFGLSAFTEHLKQDGLLHTTCGTPAYVAPEVIGKQGYDGAKADLWSCGVILYVLLAGFLPFQDDNIVAMYRKIYRGDFKCPPWFSSEARRLITKLLDPNPSTRITISKVMDSTWFKKSVPKTVKSKEEMEFEAFNGDEDANGDKSKQLETLNAFHIISLSQGFDLSPLFEERKREEKEELRFATTRPASSVISRLEEVGKAGNFSVKKSDSKVRLQGQERGRKGKLAIAADIFAVTPSFLVVEVKKDNGDTLEFNQFCSKALRPALKDIVWISPAENSTLARI

>PtCIPK9

MAEKTSDGHPTLLHGKYELGRLLGHGTFAKVYHARNLQSGKSVAMKVVGKEKVIKVGMMEQVKREISVMKMVRHPHIVELNEVMASKSKIYFAMELVRGGELFSKIAKGRLREDVARVYFQQLISAIDFCHSRGVYHRDLKPENLLLDEDGKLKVTDFGLSAFSEHLKQDGLLHTTCGTPAYVAPEVIGKKGYDGAKADLWSCGVILYVLLAGFLPFQDDNIVAMYRKIYRGDFKCPQWFSSEARRLITKLLDPNPSTRITTSKVMDSTWFKKSLPKTVRSKEEMEFEAFNGEEDANGDKAKQPETLNAFHIISLSQGFDLSPLFEEKTREGKEELRFATTRPASSVISRLEEVAKAGNFNVKKSDSKVRLQGQERGRKGKLAIAADIFAVTPSFLVVEVKKDNGDTLEYNQFCSNELRPALKDIVWTSPAENSTLA

>PtCIPK10

MEPPPQTLPPQPLQRTSSVPTTLLNKYEVGRLLGRGSFAKVYAARSLSDKTQLVAIKIIDKTKTDAAMEPRIISEISAMH

RLQHHPNVLKIHEVMATKTKIYLVMELASGGDLFSKIRKMGKLKEPAARRYFQQLVSAIHFCHQNGVSHRDIKPHNLLLDGKGNLKISDFGLSALKNGCVNGGFMLQTACGTPAFTAPEVMAQRGYDGSKADAWSCGVILFFLLSASLPFDDSNLAVMYRKIRKGEYQLPSCLPKSVKSIINQLLDPNPNKRMSIEALMKHPWFLKKFELPTKSSVFESDYKEYCKFDKSAAGGINAFDLISLSSGLDLSGLFEVKHARDRRFTSSETVERVTERVREVGGRLGYIVEEGMVGGAIGLGKGRVGLVFEVWEIVEKLLVVEVKVVERGGVEFEDLHWGELKEGLGDVVLQWNDDAM

>PtCIPK11

MEKMGPILMNRYELGRLLGQGTFAKVYHARNLQSGQSVAIKIIDKGKVLRSGLIDQIKREISVMRLVRHPNIVQLNEVMASRTKIYFVMEFVKGGELFNLVSKGKLKEDVARKYFQQLIGAVDFCHSRGVYHRDLKPENLLLDENGDLKITDFGLSALSESRRQDGLLHTTCGTPAYVAPEVINKKGYDGPKADIWSCGVILFVLLAGFLPFHDQNLMELYRKITKGEFRCPNWFHPEAKKLLSRILDPHPSSRTSIEKITKNCWFRKGYKQIETPPSPQGHARSNLIKDVHSAFDSASDNESNSRENVMVAPRSPLRPTCYNAFDIISLSKGFDLSGLFEKDKNRRQEARFTTTKSASMIMSKFEQIAMAESFSFKKKDGTLMLEGSREGRKGLLAIDAEICEVTPSFYVVEMKKKSGDSFEYKEFCDHELKPSLKDIVWAWQGSEQPQV

>PtCIPK12

MSVKVPAARTRVGKYELGKTIGEGSFAKVKVAKNVQTGDVVAIKILDRDQVLRHKMVEQLKREISTMKLIKHPNVIKIFEVMASKTKIYIVIEFVDGGELFDKIAKHGRLKEDEARRYFQQLIKAVDYCHSRGVFHRDLKPENLLLDSRGVLKVSDFGLSALSQQLRGDGLLHTACGTPNYVAPEVLRDQGYDGTASDVWSCGVILYVLMAGFLPFSESSLVVLYRKICRADFTFPSWFSSGAKKLIKRILDPKPLTRITVSEILEDEWFKKGYKPPQFEQEEDVNIDDVDAVFNDSKEHLVTERKVKPVSINAFELISKTQGFSLDNLFGKQAGVVKRETHIASHSPANEIMSRIEEAAKPLGFNVDKRNYKMKLKGDKSGRKGQLSVATEVFEVAPSLHMVELRKIGGDTLEFHKFYKSFSSGLKDVVWKSDQTIEGLR

>PtCIPK13

MSAKGQGTRTRVGKYELGKTIGEGSFAKVKVAKNVETGDVVAIKILDREQVLRHKMVEQLKREISTMKLIKHPNVIKIFEVMASKTKIYIVIEFVDGGELFDKIAKHGRLREDEARRYFQQLINAVDYCHSRGVFHRDLKPENLLLDSHGVLKVSDFGLSALSQQLQGDGLLHTACGTPNYVAPEVLKDKGYDGTASDVWSCGVILYVLMAGYLPFDETSLMALYLKICSADFTFPSWFSSGAKKLIKRILDPEPLTRITVAEIIEDEWFKKGYRPPQFEQEEHVNVDDVDAVFNDLKEHLVTEKKKKPASMNAFELISKTQGFSLENLFGKQAGFVKRETRIASHSPANEIMSRIEEAAKPLGFNVDKRNYKMKLKGDKGGRKGQLSVATEVFEVAPSLHMVELRKIGGDTLEFHNFYKSFSSGLKDVVWKSDQTIEGLRS

>PtCIPK14

MENKPSVLTKNYEVGRLLGQGTFAKVYFARSIRTNLSVAIKVIDKEKVLKVGLVNQIKREISVMRLVRHPNIVQLYEVLATKSKIYFVMEYAKGGELFDKVAKGRLKEDVAQKYFQQLINAVDFCHSRGVYHRDIKPENLLLDENENLKISDFGLSALTESKQQDGLLHTTCGTPAYVAPEVINRKGYDGTKADIWSCGVVLFVLLSGYLPFHDSNLMEMYRKIGRAEFKCPNWFPTDARRLLRKILDPNPSTRISMAEIKESSWFRKGKEVAALDWNGSGSSENSSVASEAKQESAKPPSLNAFDIISLSAGFDLSGLFEENYQKREARFTSIQPASVIISKLEDIAKRQKLKVIKKDAGLLKLEGMEEGRKGPLSVDAEIFEVTPNIHFVEVKKSNGDTLEYQKILEEAIRPALNDIVCVWQGEQELQQQQQQQSN

>PtCIPK15

MPEIEQVSENALFGKYDVGKLLGCGAFAKVYHARDVQTGKSVAIKIINKKKISSPSLIANIKREISIMRKLNHPYIVKLIEVLASKTKIYCVMEYVKGGELFTKIAKGRFSEDLSRKYFRQLISAVGYCHLRGVFHRDLKPENLLLDENGNLKVSDFGLSAVRDQTQLDGLLHTLCGTPAYVAPEILAKKGYDGAKIDVWSCGVILFVLAAGYLPFNDPNLMAMYKKIYKGEFRCPKWMSTDLKRFLSRLLDTNPETRITIDEILKDPWFKKGKHKEINFYDEEFNKDDGRKDEELASTSLNAFDIISFSSGLNLSGMFDDSYNVVDDGERFVSTESPEDLVKRVEEFAKEERLRVRRRKEWGLEMEGQDGNLMLSVEIRRLTDTLFVVEAKRNGGDAGCYKEIWKNKLKPVIFNGLISTNSVVNDD

>PtCIPK16

MPEIESVSENALFGKYEVGKLLGCGAFAKVYHARDVQTGKSVAIKIINKKKISNPSLMSNIKREISIMRRLNHPHIVKLIEVLATKTKIYFVIEYVKGGELFAKVAKGRFSEDLSRKYFHQLISAVGYCHSRGIFHRDLKPENLLLDENGSLKVSDFGLSAVTDQIRTDGLLHTLCGTPAYVAPEILAKKGYDGAKVDVWSCGVVLFVLTAGFLPFNDPNLMAMYKKIYKGEFRCPKWMSTDLKRFLSRLLDTNPETRITIDEILKDPWFKKGKHKEINFYDEELNKAVDEKKDEELASTSLNAFDIISFSSGLNLSGLFDDSYNVVDDGERFVSTESPENLMKKVEEFAKEERLRVKRRKEWGLEMEGQNGNLMIAVEVHRLTDTLFVVEAKRSGGDAGCFKETWKNKLKPPNYLLG

>PtCIPK17

MATNKTPNNISNQSQSPLLGRYEIGKLLGHGTFAKVYHARNVKTNESVAIKVIDKEKILKVGLMAHIKREISILRRVRHPNIVQLFEVMATKAKIYFVMEYVRGGELFNKVAKGRLKEEVARRYFQQLISAVSFCHARGVFHRDLKPENLLLDENGNLKVSDFGLSAVPDQIRQDGLFHTFCGTPAYVAPEVLAKKGYDAAKVDIWSCGIVLFVLMAGYLPFQDQNIMVMYKKIYKGEFRCPRWFSSELVRLLSKLLDTNPVTRITIPEIMENRWFKKGFKHIKFYIEDDKVCSVQDEDDVGSSSDQSLSESESEFETRRRVTTLPRPASLNAFDIISFSPGFDLSGLFEEGGEGARFVSGAPVTKIISKLEEIAKVVSFTVRKKDCRVSLEGSREGVKGPLTIAAEIFELTPKLVVVEVKKKGGDKGEYEEFCNRELKPGLQKSLSYRCFKYTIGF

>PtCIPK18

MATNKTPNNISNQSQSPLLGRYEIGKLLGHGTFAKVYHARNVKTNESVAIKVIDKEKILKVGLMAHIKREISILRRVRHPNIVQLFEVMATKAKIYFVMEYVRGGELFNKVAKGRLKEEVARRYFQQLISAVSFCHARGVFHRDLKPENLLLDENGNLKVSDFGLSAVPDQIRQDGLFHTFCGTPAYVAPEVLAKKGYDAAKVDIWSCGIVLFVLMAGYLPFQDQNIMVMYKKIYKGEFRCPRWFSSELVRLLSKLLDTNPVTRITIPEIMENRWFKKGFKHIKFYIEDDKVCSVQDEDDVGSSSDQSLSESESEFETRRRVTTLPRPASLNAFDIISFSPGFDLSGLFEEGGEGARFVSGAPVSKIISKLEEIAKVVSFTVRKKDCRVSLEGSREGVKGPLTIAAEIFELTPKLVVVEVKKKGGDKGEYEEFCNRELKPGLQKSLSYRCFKYTIGF

>PtCIPK19

MATNKKLGNKSNNSQSQSPLLGRYEIGKLLGHGTFAKVYLARNIKTNESVAIKVIDKEKVLKGGLIAHIKREISILRLVRHPYIVQLFEVMATKAKIYFVMEYVRGGELFNKVAKGRLKEEVARKYFQQLISAVSFCHARGVFHRDLKPENLLLDENGNLKVSDFGLSAVSDQIRQDGLFHTFCGTPAYVAPEVLARKGYDAAKVDIWSCGIVLFVLMAGYLPFHDQNVMVMYKKIYKGEFRCPRWFSSELVRLLHKLLDTNPVTRITIPEIMENRWFKKGFKNIKFYIEDDKVCSVEEEEDVGSSSDQSLSESESEFETRRRVTSLPRPASLNAFDIISFSPGFDLSGLFEEGGEGARFVSGAPVSKIISKLEEIAKVVSFTVRKKDCRVSLEGSREGVRGPLTIAAEIFELTPKLVVVEVKKKGGDRGEYEEFCNKELKPGLQKLMQEESEAAAAPKVITTVPVPSGSPQFTIDPFPTDSSQLPVDPFPTDSTQLPINPFPTDSTQLPIDPFPILSSHLPSDSE

>PtCIPK20

MEERHVLFGKYEMGRLLGKGTFAKVYYGKHSVTGDSVAIKVINTDQVKKEGMMEQIQTEISVMHLVRHPNIVELKEVMATKTKIFFIMEYVRGGELFAKVAKGRLKEEVARKYFQQLISAIDYCHSRGVYHRDLKPENILLDEDENLKISDFGLSALPEQLLQDGLLHTLCGTPAYVAPEVLRKKGYDGSKADTWSCGVILYVLLAGFLPFQDENVMKMYKKIFKAEYEFQPWFSTDAKRLISRLLVADPERRITIPAIMRNHWFLKGFLRPMAFSIQESIMDKTEEDQDLDSCSVVKPKVSSPRFFNAFEFISSMSSGFDLSSLFETKKKPGSMFTSKVSASAIMEKIEGVAEGLNFKVAKVKDFKVRLQSPCEGRKGKLAVTTEVFEVAPEVAVVEFSKSSGDTLEYATFCKEDVRPALKDIVWTWQGDNVFLIGEIQRFFNNKINNFK

>PtCIPK21

MEVVGETPPASAGEVNLFGKYELGKLLGYGAFAKVYHARNVSTGQSVAIKAVSKAKVMKEGFVAHVKREISIMSRLRHAHIVRLHEVLATKGKVYFVMEYAKGGELFSKISKGRFSEDLSRRYFQQLITAVGYCHARGVFHRDLKPENLLLDENFNLKITDFGLSAVTEQVRPDGLLHTLCGTPAYVAPELLAKKGYDGAKVDIWSCGVVLFVLIAGYLPFNDTNLMAMYRKIYKGQYRCPKWTSPDLKRLLSRLLDANPETRITIDMIINDPWFKKGFKEEKKLYLDEFDFDKGFEEEEDEKSLNAFDIISFSSGYDLSRMFNESDTMILTERFVSAEKPEKVMERIEEVARKEGLEIAKRKNWGAKLEGCNGNFIMIIEVHRLTDHLVMIEVKEKKFKIGPGQETWEDKLKPQLRSLIYQPEQAVSGN

>PtCIPK22

MENKGNVLMQKYEIGRLLGQGTFAKVHHARDLKTGMSVAIKMIDKEKVFKVGMMDQIKREISVMRLIRHPNVVELYEVMATKTKIYFVMEYVKGGELFNKVAKGKLKEDVARKYFQQLISAVDYCHSRGVSHRDLKPENLLLDENENLKVSDFGLSALAESKRQDGLLHTTCGTPAYVAPEVINRKGYDGAKADIWSCGVILYVLLAGYLPFRDPNLMEMYRKIAKGEFKCPNWFAPEVRKLLSKILDPNPNTRISMAKIMENSWVRKDLQSRPCVLEAEVKEPAPLDSEAVFGINEISCAAVEPKQEVAKPCNLNAFDIISFSAGFDLSGLFEEKEQKKEVRFTSNKPASTIISKLEDIAKRLRLKIKKNDGGLLKIEGSKEGRKGVMGIDAEIFEITPFFHLVEMKKSSGDTVEYQKVVKQDIRPALKDIVWSWQGEQQREQLQLQEQQELQPSHASTTQVVSTQNSS

>PtCIPK23

MMETEQVADEDCHVNNGARKILLGKYEMGRLLGQGTFAKVYRGKNLVTQENVAIKVLHKDQVKRTGLMEQIKREISIMHLVKHPNIVELKEVMATKSKVFFVMEFVKGGELFAKIKKGGLEEDLARKYFQQLICAVDYCHSRGVSHRDLKPENLLLDDKEDLKVSDFGFSALPEQKWNDGLLHTRCGTPAYVAPEVLRKKGYDGAKADTWSCGVILFVLLSGHLPFRNENAMKMYVKILKAEYEFPPWISRDAKRLISNLLVVDPEKRITIPEIRANSWFQKGLAQSAVSSNDLEDTENGVRSEDQEEFPGKTGSKHSPPFYNAFELISSMSSGFDLSSLFETKRRRGSMFTSKCPASMILSTLKSVAKKLNFRVAVSDTEFKVKLLGKEEGRKGKLAVTAEVFELAAELVVVELSKCSGDTFEYTKFCEEDVRPALKDIVWSWQGDNIDSHEGQVGTSN

>PtCIPK24

MSSSRSGGSGSRTRVGRYELGRTLGEGTFAKVKFARNVETGENVAIKILDKEKVLKHKMIGQIKREISTMKLIRHPNVVRMYEVMASKTKIYIVLEFVTGGELFDKIASKGRLKEDEARKYFQQLINAVDYCHSRGVYHRDLKPENLLLDASGFLKVSDFGLSALPQQVREDGLLHTTCGTPNYVAPEVINNKGYDGAKADLWSCGVILFVLMAGYLPFEESNLMALYKKIFKADFTCPPWFSSSAKKLIKRILDPNPSTRITISELIENEWFKKGYKPPTFEKANVSLDDVDSIFNESMDSQNLVVERREEGFIGPMAPVTMNAFELISTSQGLNLSSLFEKQMGLVKRETRFTSKHSASEIISKIEAAAAPLGFDVKKNNFKMKLQGEKDGRKGRLSVSTEVFEVAPSLYMVEVRKSDGDTLEFHKFYKNLSTGLKDIVWKTIDEEEEEEAATNG

>PtCIPK25

MSSSRSGGGGGGGGGGSGSKTRVGRYELGRTLGEGNFAKVKFARNVETKENVAIKILDKENVLKHKMIGQIKREISTMKLIRHPNVVRMYEVMASKTKIYIVLQFVTGGELFDKIASKGRLKEDEARKYFQQLICAVDYCHSRGVYHRDLKPENLLMDANGILKVSDFGLSALPQQVREDGLLHTTCGTPNYVAPEVINNKGYDGAKADLWSCGVILFVLMAGYLPFEEANLMALYKKIFKADFTCPPWFSSSAKKLIKRILDPNPSTRITIAELIENEWFKKGYKPPAFEQANVSLDDVNSIFNESVDSRNLVVERREEGFIGPMAPVTMNAFELISTSQGLNLSSLFEKQMGLVKRESRFTSKHSASEIISKIEAAAAPLGFDVKKNNFKMKLQGDKDGRKGRLSVATEIFEVAPSLYMVEVRKSGGDTLEFHKFYKNLSTGLKDIVWKTIDEEKEEEEAATNG

>PtCIPK26

MMKKVTRKVGKYEVGRTIGEGTFAKVKFAQNRETGESVAMKILSKSAILKHRMVDQIKREISIMKLVRHPYIVMLHEVLASRTKIYIILEFVTGGELFDKIVHQRRLEENESRRYFQQLIDAVAHCHCKGVYHRDLKPENLLLDAFGNLKVSDFGLSALPQKGVGLLHTTCGTPNYVAPEVLGHQGYDGAAADVWSCGVILFVLMAGYLPFEETDLPTLYRKINAAEYSCPFWFSPGAKALIDKILNPNPKTRIGIEGIKKHPWFQKNYEPVGHREDEEVNLDDVHAVFDDIEDQYVAEQLENSEDGPLVMNAFEMITLSQGLNLSALFDRRQDYIKRQTRFVSRKPAKDIISAIEAVAESMNLKVHTRNYKTRLEGVSENKAGQFAVVLEVYEVAPSLFMVDARKASGETLEYHKFYKNFCTKLENIIWKPPEGAKLPACFEQ

>PtCIPK27

MMKKVTRMVGKYEVGRTIGEGNFAKVKFAQNRETGESVAMKILAKSTILKHKMVDQIKREISIMKIVRHPNIVRLHEVLSSRTKIYIILEFVTGGELFDKIVHQGRLSENESRRYFQQLIDAVAHCHRKGVYHRDLKPENLLLDAFGNLKVSDFGLSALRQKGVGLLHTTCGTPNYVAPEVLGHQGYDGAAADVWSCGVILFVLMAGYLPFEEIDLPTLCRKYSCPFWFSPVAKALIDKILDPNPKTRIGIEGIKKHPWFRKSYEPVGHSEEEEVNLDDVHAVFDDIEDHYVAEQLENSEGGPLVMNAFEMIALSQGLNLSALFDRRQDYVKKQTRFVSHKPAKVIISAFEAVAESMNLKFHTHNYKTRLEGISENKAGQFAVVLEVYEVGPSLFMVDARKASGETLEYHEFYKNFCAQLENIIWKPPEGC

>TaCIPK2

MGEQKGNILMGKYEMGKMLGQGTFAKVYHARNIETSQSVAIKVTDKEKVLKGGLTDQIKREISVMKLVKHPNIVQMYEVMATKTKIYFVLEHVKGGELFNKVQRGRLKEDAARKYFQQLICAVDFCHSRGVYHRDLKPENLLLDENSNLKVSDFGLSTISECRRLDGLLHTSCGTPAYVAPEVINRKGYDGAKADIWSCGVILFVLLAGYLPFQDKNLMNMYKKIGKAEFKCPSWFSSDIRRLLLRILDPNPGTRISIERIMEHPWFRKGLDAKLLRYNLQAKDAVPAADMTATSDSLSCSNSATEGKEQEAKKLSNLNAFDIISLSTGLDLSGMFEDNDKKRESKFTSTNSASTIVSKIEDIAKCMRLKLVKKDGGMMRMESFKPGRKGVMSIDAEIFE

VTPDFHLVELKKTNGDTIEYQKVLNQEMRPALKDIVWAWQGEQQPQPQPQPQQQPC

>TaCIPK3

MYKARRQASLKVRRRVGKYELGRTIGEGTFAKVRFAKDTESGDPVAIKILDKAKVHKHGLVEQIRREICTMKLIQHPNVVRLHEVMGSKARIFIVLEYVTGGELHDIIAARGSLKEDEARRYFQQLINAVDYCHSRGVYHRDLKLENLLLDTAGNLKVSDFGLSAISEQAKADGLLHTTCGTPNYVAPEVIQDKGYNGALADLWSCGVILFVMLAGYLPFEDDNVSALYKKISGAQFTCPSWFSDGAKRLIPRILDPNPSTRITIPQLLKDPWFKKGYKPPVFDEKHQTSLDDVDAALGDSEEKQVEEEMEGQPASMNAFELISLNKGLNLRNFFEADKKYRRETRFTSQCPPEEIISRIEEAAKPLGFDIQKKNYKMRMKNLEAGRKGNLNVATEVFQVAPSLFVVELKKAKGDTLEFQKFYKTLSAQIKDVVWVCESEAEERGST

>TaCIPK4

MNGKSKKSKGTPLLGKYELGRLLGRGTFAKVYFAHPVTGGEPVAVKVIDKAEVMGMEGMAPRVLREVVAMRRLRHPGVLRLHEVLATRSRIYLVMELAPRGDLQSMLAALPNRRFSEKAARRVFVQLTAALAHCHARGVTHRDVKPQNVLLDSAGNLKVSDFGLSALPDTLRDDGRLHTACGTPAYAAPEVLRHTSYDGAKADAWSCGVMLFVLLAGRLPFDDANIPDMCRKAHRRDYEVPPWVSPPARRLVHRLLDPNPATRVSVEALAATHPWFVKRSLSLDSQLDGLLDGQPERALA

FRAPAVNAFDIISMSQGLDLSGLFGGSKSREKRFMTTASPEQTLEQLSRASGKLGYVVVGKKGVGCQRRPPGRPAISVGISELVPPLMLVEMRLEMDDGDGEVQVFGWDQLRVELGDVVRAWHSCEDLQQVQ

>TaCIPK5

MERKSAILMNRYELGRMLGQGTFAKVYHARSLATNQSVAIKVIDKEKVLRVGMIDQIKREISIMRLVRHPNIVQLHEVMASKTKIYFAMEYVRGGELFARVTKGRLKEDAARKYFQQLIGAVDFCHSRDVYHRDLKPENLLVDEHGNLKVSDFGLSALKESQKQDGLLHTTCGTPAYVAPEIINKKGYDGEKADIWSCGVILFVLLAGYLPFQDSNLMEMYRKISKGDVRHPQWFSSDARKILSRLLDPNPNTRITMDKLIEHPWFKKGYKPAVTLATPRASKDLNDVQAAFSTDQKDGKGNRAEQPNSPLKPASLNAFDIISLSKGFDLSGLFEKDQEQKSNSRFMTQKPASAIVSKLEQIAETESFKVKKQDGLVTLQGSKEGRKGQLAIDAEIFEVTPSFYVVEVKKSAGDTLEYERFCKMGLRPSLKDICWNGPSEEKLPSVSESVPPTPSSKSTKRNVI

>TaCIPK7

MAVAKSKAGKHAAPLLGKYELGRLLGRGTFAKVYHARSLVGGEAVAIKVLDKPELAATAGMDARVLGEVSAMRRLRHPNVLRLHEVLATRSKVYLVMELAPGGDLLSRLAALPKRRLPEHAARRVFLQLVSALIYCHARGVSHRDVKPQNVLIDADGNLKVCDFGLAALPESHRDDGRLHTACGTPAFAAPEVLRRKAYDGVKADAWSCGVILYVLLAGRLPFDDSNIAEMCMKAHRREYTLPEWVSQPARRLVSRLLDPNPATRLTVAELSSHPWFKRSLSLDSQLGSLLGGAAERDLLFQAPPTLNAFDIISMSPGLDLSGLFGENRRSREKRFMTTASPEQMVEQLGHSGAKLGYFMVGKKGVERLPLGGLSGLVAMSMEMSEVAPPLMLVELRLEAGDDEEVQAFGWDELRTELGEVVMAWHGCEEL

>TaCIPK8

MIGGGGGGALRRVGKYEVGRTIGEGTFDKVKFAQNTETGESVAMKVLDRSSILKHKMVDQIKREISIMKLVRHPNVVRLHEVLASRKKIFIILEFITGGELFDKIIRHGRLSEADARKYFQQLIDGVDFCHSKGVYHRDLKPENLLLDSQGNLKISDFGLSAWPAQGAALLRTTCGTPNYVAPEVLSHKGYDGALADTWSCGVILYVLLAGYLPFDEVDLTTLYGKIESAEYSFPAVFPSGAKSLIRRILDPSPDTRIRIKEIRKDEWFKKNYEPAREVENEEVNLDDVNAAFDDPEEDNEHTFDDEAGPLTLNAFDLIILSQGLNLSALFDRRQDYGKLQNRFLSRKPANVILSSMEVVAQSMGLKTHIRNYKMRVEGPNANKTSHLTIMLQIFEVAPSIFMVELERSAGETSEYNKFVNNYCSKLDDIIWKVSAEKGKSRTSRLSKR

>TaCIPK9

MAAAAGRGGGPRRTTRVGPYELGKTVGEGSFAKVKIAKDTRNAATCAIKVLDRNHVLRHKMVEQIKREIATMKLIRHPNVVQLHEVMASKSKIYMVLEFVEGGELFDKIVNSGKLGEDEARRYFHQLINAVDYCHSRGVYHRDLKPENLLLDSYGALKVSDFGLSAFSPQTKEDGLLHTACGTPNYVAPEVLADKGYDGMAADVWSCGIILFVLMAGYLPFDDPNLMTLYKLISRANVSCPPWFSTGARNLIKRILDPNPHTRITIAQILEDEWFKKDYKPPHSEHNEDVSLEDVDAAFDSSEEHLVAERREKPESMNAFALISRSEGFNLGNLFEKEMMGMVKRETSFASQRTPQEIMSKIEEACGPLGFNVRKQNYKMKLKGDKTGRKGHLSVATEVFEVAPTLHMVELRKTGGDTLEFHSFYKNFSSELKDIVWKTESNTIAK

>TaCIPK10

MVEKKGNILMERYEMGRLLGQGSFAKVYYGRSLKTSQSVAIKVIDKEKIFKCGLMDQVRREISVMKLVKHPNIVQLYEVMATKTKIFFVLEYVKGGELFNKVQRGRLKEDVARKYFQQLNSAVDFCHSRGVYHRDLKPENLLLDENRNLKISDFGLSALAECKRQDGLLHTTCGTPAYVAPELISKKGYDGAKADIWACGVILYVLLAGYLPFQDKNLMDMYKKIYKAELKWPSWFSSDARRLLRHILDPNPGTRISFSEILDNSWFRTGLDKGLISYNTPTEGIVAVDMDPTCDPFSSCTTETIQEATELTNLNAFDIISLSSGFDLSGMFEDKSNKESKFTSTNTAATIITKLEDIAKRLRLRLMKRDGGLLKMQSLQPGRKGVMSIDTEIFRIAPNFHLVEIRKTNGDTLEYQKVKHDMRPALKDIVWAWQGEQP

>TaCIPK11

MMDERRTILMGRYEIGKQLGQGNFAKVYYARNLATGQAVAIKMINKDKVTKVGLMEQIKREISVMRLVKHPNVLQLFEVMATKSKIYFVLEYAKGGELFNKIVKEGKLNEDAARRYFHQLISAIDYCHSRGVYHRDLKPENLLLDENENLKVSDFGLSALADCTRQDGLLHTTCGTPAYVAPEVLSRKGYDGAKADVWSSGVILYVLVAGYLPFHEANLIEMYRRISKADFKCPRYFSAELKELLYKILDPDPSTRIPISRIKRSAWYRRPVEVNAKKTEPEATHNTFSAEAASSGSTGCSTSGGNQGSLSLPNLNAFDIISLSTGFNLSGFFEDEYGRREERFTTRQPVTIVLTKLKELAKRLKLKIKKKENGVLKLAAPKEGKKGFLELDAEIFDVAPSFLLVELKKTNGDTFEYQKLVKEEVRPALKDIVWVWQGDQQQRSEPILQGEQHHSPSPTQQPHDELQPPLQQQEGQDQLHPPLQPQEQQNLQEQPPLPPQNGFKHQD

>TaCIPK14

MANRGKILMERYELGRLLGKGTFGKVHYARSLESNQSVAIKMLDKEKVLKVGLSEQIRREVTTMRLVAHKNIVQLHEVMATRNKIYFVMEYVKGGELFDKVAKSGKLTEGAAHKYFQQLISAVDYCHSQGVYHRDLKLENLLLDENENLKVSDFGLSALSESKRQDGLLHTTCGTPAYVAPEVISKTGYDGAKSDIWSCGVILFVLVAGYLPFHGSNLMDMYRKIEQGDFRCPSWFSHKLQKLLCKILDPNPSTRASIQKIKESTWFRKGPRGTLAVKERTPSENVTTNAPPTAGVRPRKNTHEDVQPLTVTNLNAFEIISFSTGFDLSGLFIQEDCRKETRFTSDKPASAIISKLEYVAKALNLRVRKKDNGVVKMQARKEGRNGAVQLDMEIFEITPSHHLIEMKQTSGDPLEYRELLEDIRPALKDIVWAWHGDDHQQQLE

>TaCIPK15

MENSGKIVMGRYELGRLLGKGAFGKVHYAKNLESNRGVAIKILDKEKVLKVGLAEQVRREITTMRLVTHKSIVQLHEVMATRSKIYFVMEYMKGGELFDKVSKAGKLTEGAAHKYFQQLISAVDYCHSRGVYHRDLKPENLLLDGNENLKVSDFGLSALSESKSQDGMLHTTCGSPAYVAPEVISKGGYDGAKSDIWSCGVILFVLVAGYLPFQGQNLIEMYRKIEKGDFRCPGWVSQKLQKLLYKIMDPDPNKRISIQKIKESTWFRKGPGENLTVKERLPSENANTDAVPTLGVRRRKNSHEDAKPLAVTNLNAFEIISFSTGFDLSGLFVEKESKKEARFTSEQPASAIVSKLEAVAKTLNLRVRKKDNGVVKMQVRKEGRNGVLQFDSEIFEISPSYHLIEMKQTSGDSLEYQKLLEESIRPALKNICLGMAWG

>TaCIPK16

MARGREGEVRNLVLGKYELGRMLGQGSFAKVYYGRDLRDGQSVAIKVIDKARLRQTDGMVEQLRREISIMRMVRHPNVVGIREVLASRQRVFVVMEYARGGELFAKVARGRLTEDAARKYFQQLVAAVAFCHSRGVAHRDLKPENLLLDEEGRLKVTDFGLAALPEQLRHDGLLHTQCGTPAYVAPEVLRKRGYDGARADMWSCGVVLYVLLCGFLPFQHDNYVKMYQKIFKGEYQMPPWVSGEARRLIGRLLAVDPAKRISIPEIMLTPWFKRGFVPPVPSSPVTPRKWDDDNAAALIDGSEDSSGNISPRTCNAFQLISSMSSGFDLSGLFESEQKAATVFTSRAPAATVFHKLESAGKALRYNTTRGKGWRIRMEAKADGANGRLAVTAEVFEVAAD

VTVVEFAHDGGDALDFNKFCAEDVRPGLADIVWAWQGDVPALPGAVA

>TaCIPK17

MVATGDAEEAAAGCRARAALLGAYELGRTLGEGSFGKVKHARHRATGDHFAVKILDRGRVLSLRGADDQVRREIATLTMLAHPNVVRLHEVAASKTKIYMVLEFVNGGELFDRIAMKGKLSEREGRRLFQQLIDGVSYCHGKGVYHRDLKPENVLIDRKGNIKISDFGLCALPQHLGKDGLLHTTCGSPNYIAPEVLQNRGYDGSLSDIWSCGVILYIMLIGQLPFDDRNMVVLYQKIFKGDTKVPEWLSPGAQNLLKRILEPNPMKRINMAEIKLHEWFQKDYTPVGPYDDDDEDVRLGAILPVKQQISEAPGDKSTHQMNAFQLIGMASSLDLSGFFEEEGVSQRKIRFTSTLTPKDLFDKIDVSATQSGFHVQRAHSKLKITGNCNGPNNPSPFLVCAEVFVLGPSLHVVELRKSQGDTAVYKKLCDRISSDLGIDNIFGMGSLFDENLPNFDSRAATPLVAL

>TaCIPK19

MAATKPPPRDPSPQAARLPSPSSSSSSAAAAAAKRGGTGSRGLLMGRYELGRVLGKGTFAKVYHARHLQT

GESVAIKVLDREKAVRSGLVSHIKREIAVLRRVRHPNIVHLFEVMATKTKIYFVMELVRGGELFSRVSKG

RLKEDIARRYFQHLISAVGFCHTRGVFHRDLKPENLLVDEAGNLKVSDFGLSAVAEPFQPEGLLHTFCGT

PAYVAPEVLARRGYEGAKADIWSCGVILFVLMAGYLPFHDQNLMAMYRKVYKGEFRCPRWFSKDLTSLIM

RFLDTNPSTRITLPEVMENRWFKKGFRPVKFYIEDDQLYNVIDAENDMLDLGLTDPLPQPLPPPPPSPSP

QEVDGDDSGSESDASVVSCPATSSFEERHRLRGPLPRPASLNAFDIISFSRGFNLSGLFEEKGDEVRFIS

SEPMSDIITKLEEIANVKSFAVRKKDWRVSLEGTREGVKGPLTICAEIFELTPSLVVVEVKKKAGDKEEY

DDFCNKELKPGMQHLVHQMVPVPNTPTISE

>TaCIPK21

MAAAEGAMRMGKYEMGRTLGEGHFGKVRLARHADTGRAFAIKILDRQRILAMKIDEQIKTEIATLKLLKHPNVVRLYEVAASKTKIYMVLEYVNGGELFEKIALKGKLSEKEGRKLFQQLMDAISYCHERGVYHRDLKPENVLVDAKGNIKVSDFGLSALPQHQRKDGLLHTTCGSPNYIAPEVLLNKGYDGSMSDVWSCGVILYVMLTGNLPFDDENMVVLYQKILKGDYRIPKWLSPGAQDILRKLLDPNPITRLGMDGLREHDWFNQSYTPAVPFDDDDDNYVGDDNSHMTKNNGIQDNPAINQMNAFQLIGMSSCLDLSGFFEKEDVSERKTRFASNYPPTYLFEKIESNVINMGFQVQKNNGKLKVIQERKGPTNPRGHGSLLISAEVFEINESLYVVELKRSCGDCSLYRQLCATLSDDLGICKSQQLLKNDSIRQELYRFNSSF

>TaCIPK22

MRLLDVLASRSTVYLVLELARGGTLLSAMDERGRFDEPTSRRLFVQLVSALAHVHSRGVFHRDVKPENLLLDEHGDLKLTDFGLCALADRHLGADGLAATRCGSPAYVAPEILYKKRYDAGKVDVWSSGVALFSLTAGYLPFNDGNLMGMYRKIFSGRFRCPRWFSPELRSLVGRMLDPDAGTRIKMDEIMEHPWLQQDGTSSFDIIRAPSSDPRPEVMKWEAEMEQVRELNAFDIIAFASGCDLSGLFGPLPDRVRFAVVGVVIASVLDKAEEIGREEGFVMRRKEEVGCGGIMFEAIQREIIAMVRVSRLLEEMLMVEVERASSSEAPNLWERLQQGLKFSND

>TaCIPK23

MVDSSAGGKMSAHGGGGGRTRVGRYELGRTLGEGTFAKVKFARNVETGENVAIKILDKDKVLKHKMIAQIKREISTMKLIRHPNVIRMYEVMASRTKIYIVMELVTGGELFDKIASRGRLKEDDARKYFQQLINAVDYCHSRGVYHRDLKPENLLLDANGTLKVSDFGLSALSQQVREDGLLHTTCGTPNYVAPEVINNKGYDGAKADLWSCGVILFVLMAGYLPFEDSNLMALYKKIYKADFSCPSWFSTSAKKLIKKILDPNPNTRITIAEVINNEWFKKGYQPPRFETAEVNLDDINSIFNESGDPAQLVVERREERPAVMNAFELISTSQGLNLGTLFEKQTDSVKRETRFASRLPANEILSKIEAAAGPMGFNVQKRNYKLKLQGENPGRKGQLAIATEVFEVTPSLYMVELRKSNGDTLEFHKFYHSISNGLKDVMWKPEGSIAEGDETRHRRSP

>TaCIPK24

MAGAARKKLVGRYEVGRTIGQGSFAKVKFAVDADTGAPVAMKVLDKATILNHRMLQQIKKEISIMKIVRHPNIIRLNEVLAGQTKIYIIMELITGGELFDKIARQGKLRENEARKYFQQLIDAINYCHSKGVYHRDLKPENLLLDSRGNLKVSDFGLSSLSQNGFLHTTCGTPNYVAPEVLSDGGYDGSAADVWSCGVILYVLMAGYLPFEENDLPTLYDKITAAHFSCPDWFSQGAKSLIQRILDPNPKARITIKEMKADTWFSKNYVGVRHGEDENVSLDDVQAAFDNIEDKYVSEQVTRNDGGPLMMNAFEMITLSQGLNLSSLFDRQQEYVKRQTRFVSRKPAKTIAATIEVVADSMGLKVHSQNYKLRLEGVSSNKMSPFAVVLEIFEVAPALFMVDVRKVAGDSLEYHRFYKSLCSKLESIIWRPIEVSAKSALLRTTTC

>TaCIPK25

MGDRPKLPARYEQVKLLGEGNFAKVYLARHMDTKEEVAIKVMDKEKLIKLGAVQQIKREIAVMRRLRHPNIVQLHKVMACKSRIFVVMEYVRGGPLYRHIPANSGLKEDETRRIFQQLVSALTFCHAQGVYHRDIKPDNLLVDEHGNLKVADFGLSAHADTARREALLHTVCGTPLYVPPEVFARRGYDGAKADAWSCGIVLFVLAAGRKPFRDDDFITLYRTICRGDYRCPRTFSPELVRIVRRLLQPNPARRITLLQIKETDWFKKGFKEVSFYIDNKDCLRSLDGSEEPDLCDSDSEDETAMSSSSSGSSSPVAHGDGGGMHTSVSAPSLVNLEKMHIAAARAPEPRIRRIKSMNAFDIIASSPSFDLSGLFEERGEQLRFVSSAPVNTIISKLEEIAGQVSFTARTKDCQVSFEATRNGHKGALAISTKIFQLTPELVMVQVCKKAGDTAEYRQFCGSELKPGLRGIVDGLPEDGLPPTLNVA

>TaCIPK26

MEDRRTILMDRYEIGRQLGQGNFAKVYYGRNLAGGQAVAIKMIDKEKVSRVGLIVQIKREISIMGLVRHPNVLKLFEVMASKSKIYFVLEYAKGGELFNKITKGKLSEDAARKYFHQLISAVDYCHSRGVYHRDLKPENLLLDENENLKVSDFGLSALAESTRQDGLLHTTCGTPAYVAPEVLSRRGYDGAKADIWSCGVILFVLVAGFLPFHDTNLIEMYRKISRAEYRCPRPFSVELRDLLYKILDPDPSTRASVSRIKRSAWYRKPVDVNGLKIKQETRDKVQKGEPTTSESTEGSNSEVNQEASSSLTNLNAFDIISLSTGFDLSNLFEEKYGRREDRFTTRQPAEAIFAKLNEVANKLKLKIKKKENGVLKLAAPKEGMKGILEFDAEVFEFAPSLHLVELKKTNGDTIEYKQLMKDEIRPALKDVVWAWQGESHPLPEKFIRGEQQQSPLPSQQQQQQQSPLPSQQQQQQSPLPSQQPQE

>TaCIPK27

MEDAAEGKTNVLQGRYELGRVLGHGNFGRVHAARDLHTGRGVAVKVVAKDKVERAGMVEQIKREIAVMKMVSHPNIVELHEVLATRTKIYLALELVRGGELFARISRAGRLREDVARRYFRQLISAVDFCHGRGVYHRDLKPENLLLDEAGNLKVADFGLSALAGHARPDGLLHTACGTPAYVAPEVLGGNGYDGAKADIWSCGVILYVLLVGALPFQDENLMSMYRKMQRGGFLCPSWVSKDARKLIGRLLDPNPSSRITVASLVESPWFKKTTPIPNPLLEPVPSAAARGNGEDKDEPPEALNAFHLISLSAGFDLSPLFDQEPPTGRGTRGGVMRFATREPASGVISRLEGLATGGAMRVTKSGARGVRLEGAERGRKGRLGVAAEFFSVAPSVLVVDVKKDGGDTMEYRSFCSDELRPALKDIVWAAAAAAAAASPGDPPAPT

>TaCIPK28

MEERSVLTQRYEIGRQLGQGTFAKVYYARNLANGQSVAIKILDKDKILKVGLVDQIKREISIMRIVRHPNVLQLFEVMATRSKIYFVLEYAKGGELFNKLAKGKLSEEGARTYFHQLISAIDYCHSRGVYHRDLKPENLLVDEYGTLRVSDFGLSALTKSKWRDGLLHTACGTPAYVAPEVLSRKGYNGAKADVWSCGVILFVLVAGYLPFHERNLMELYRKIAKAEYRCPRYFSTDLKELLSGILDPDPNTRMSIARIKRSPWYRKPVERTPLRKDKTYASEAAASGLPCRKSSDGPASMNAFNIISLTPGFDLSGLFDERYSQREARFASNEPPAAVFVKLEELAQRMKLKVTKKDNGAMKLAAPREGKKGTLEFEAEIYELASSFLLVELKKTNGDTMEYQKLLKEDIRPSLKDIVWSWHGDVQQQAQLPQDPQWPPPQQAQQPQDRQWPPPLPPQRLEHLAANAPPPQ

>TaCIPK29

MPSASSAVPSAAAPGDESQPLAAPKLLLGRYELGGLLGRGASAKVYRARDILTGRDVAIKSFPNPRAGAREGEGSAGSAAIEREAVILSRLRHRHVVRLHEILGTRKKVHFVLDLAAGGELFSLVDSDGRMTEDLARHYFRQLVSAVRYFHSRGVYHRDIKPENLLLDGDGDLKVADFGLGAITDESLHHTLCGTPAYVAPEILSKQGYHPAKVDIWSCGVVLFVLAAGYLPFNDASLINMYRKIYAGRFRCPNWFSPALRHLLRRILDPNPSTRIDTDGIMEHPWFRHGASGDGELEELMRGHEEEAWFKTEFKEDMARDMTAFDILAFSPGSDLSGLFGAGPGTERVFVGEPAAAVLARVEDAGKKQGHRVRREGKGRAGPVYVEAEAGGIVAKVTVFRIADAVSVVEVVKGHGAEAAAFWSDWLEPAVKPQAV

>TaCIPK30

MRRVHHPNVVRLHEVMATRSRIYFVMEYASGGELFARLAESTRFPEPVARRYFQQLITAVEFCHSRGVYHRDLKPENLLLDAHGNLKVSDFGLSALADGASRHRGDALLHTTCGTPAYVAPEVILKRGYDGAKADIWSCGVILFVLLAGRLPFHDTNLVLLYKRIARSDYQCPAWFSIDARKLLARLLDPNPNTRITITKLMARTWSQKDSCPLSDKPLDTSETAVFLCQEAGGHHDDDQPEGATRKRKRSKVTASSPTINLRPSSMNAFDIISRSSVLDLAKMFDAEHKTSEARFSSKETTTVIVSKLGKIAEAGRFSFKLNKEKGRVELEGSQDGRKRALALEAEIFEVAPSVHVVEMRKTGGDSLEFQDFYKQELKPSLGDIVWAWQGGDSPPPTLTTAVPRSNTNS

>TaCIPK31

MYRAKRAALSPKVKRRVGKYELGRTIGEGTFAKVRFAKNTETMEPVAIKILDKEKVQKLRLVEQIRREICTMKLIKHPNVVRLHEVMGSKARIFIVLEYITGGELFDTIYTNGRLKEEEARKYFQQLINAVDYCHSRGVYHRDLKLENLLLDAAGNLKVSDFGLSALTEQVKADGLLHTTCGTPNYVAPEVIEDRGYDGAAADIWSCGVILFILLAGFLPFEDENIIALYKKISEAQFTCPSWFSTGAKKLITRILDPNPATRITIPQILEDPWFKKGFKPPVFDDKYETSFDDVYAAFGDSEDQHVKEETEHKPTSMNAFELISLNQGLNLDNLFEAKEEHKRETRFTSQCPPKEIISKIAEAARPLGFDIQKKNYKMRMENPKAGRKGNLNVATEVFQVAPSLHVVELKKAKGDTLEFQMFYRSLSTQLKDVVWKCGGEVEDNSTVA

>TaCIPK32

MSTTKVKRRVGKYELGRTIGEGTFAKVRFARNTETGDPVAIKILDKEKLLKHKMVEQIKREIATMKLIKHPNVVCIHEVMGSKTKIYIVLEYVTGGELFDTIVNHGRMREDEARRYFQQLINAVDYCHSRGVYHRDLKPENLLLDSCGNLKVSDFGLSALSQQLKDDGLLHTTCGTPNYVAPEVLEDQGYDGAMADLWSCGVILFVLLAGYLPFEDSNLMALYKKISDAEFTFPPWTSFPAKRLLTRILDPNPMTRITVQEMLEDEWFKKGYKRAEFDEKYDTTLDDVDAVFNDSEEHHVTEKKEEEPVALNAFELISMSAGLNLGNLFDSEQEFKRETRFTSKSSPKEILRKIEEAAKPLGFDIHKKNYKLRLEKVKAGRKGKLNVATEILRVGPSLHMVEVRKAKGDTLEFHKFYKNLSNTLKDVVWKSDDLQIQPS
