# Supplementary material for: Identification of CBL and CIPK gene families and functional characterization of CaCIPK1 under Phytophthora capsici in pepper (Capsicum annuum L.)
Source: BMC Genomics. 2019 Oct 25;20:775. doi: 10.1186/s12864-019-6125-z (PMC6814991; doi:10.1186/s12864-019-6125-z)
Supplement: Supplementary file 10 — Additional file 10. 1500 bp promoter sequences of CaCBL and CaCIPK genes. [file 12864_2019_6125_MOESM10_ESM.doc]

**>CaCBL1**

TTTTTTTTCTCTACCAAGAATCCAAGATTTTAGCTTTTTCTTTTGTTGGTATGTTTCAAGATTTNTGGGTGTTTTGGGTA

GATGGATTTTTGAGGGCTGTATTCTTTGTTGGGGAAAAAATTGAGGTTGTATGTGTTTAGTTATGGGAAGTAGTGATATT

TATGAACCCTTTATGGATTTGAGATCAAAATTGAAGCTTTTACATGTTAAAGTTTACAAAATCTTGTTGGTTTTTGTATT

AGGAATTTAAAGTGGAGGTTTTCTTGAAATTTTACTTGGGAGTGGCACAGTTGGAATTTCGAGAGTGGTTTGTTTGGATG

TGGAGGAGGTTTGGGGTGGGTAGGGGGCTAATTGCTAATTAGGTAACGGCCTCTCTGTATACTAGTTGTTTTGTATGTTT

GTGGGATTATGTTGGGTATGTAGTTCTTCGGTCTGCCTCTACCTCGCTTGAAACCATCCCTAACTAACTTCTCACACCTC

TGCACTGGGGCATCCGTGCTCCTCCTCATCACATGCCTAAACCATGTCAACCTCGCTTCCCTCATCTTGGCTTCCACCGA

AGTCACTCCCACCTTATCTCAAATAACTTCATTTCTAATCCTATCTTTCCTAGTACACCCATACATCCATGATCCACCGA

AGCATTCTTATCTCGGCCACCTTCATCTTCTATATGTGGGCTTTTTAATAGGCCAACACTCCGCCCCATACAACAAAGCC

GGTCTAACCACCACTCTGTAGAACTTTCCTTTAAGTTTGGGAGGTACCTTTTTGTCACACAAGATTCCGGAGGCGAGCCT

CCATTTCATCCACCCTACCCCTACCCCAATCTGATGAGTGACATCTTCGTCAATCTCTCCCTTCTCCTGAATCGTAGAGC

CCAAATACTTGAAACTCTCTCTCTTACTGATAGAATGAGTGTCAAGCTTAACAACCACATCGGACTCATGAGACACCTCG

CCGAACTTACACTCCAAATATTCCATCTTGGACCTACTCAACCTAAACCCTTTAGACTCAAGGGTTTGCCTTTAAACCTC

CAATTTAGCATTAACTCTACTACGTGTCTCTACTTGGTTCTTCCATTATCTTTCCTTTTGGTTTTTGTTTGCCATGTTCT

TAGATAGGTGACGATTTTTGAGTAATTACTAAGTTTACTACTTAAAAGAACAATTTATTGCTTAAGCTCGGATTTTTATG

AGATATGGATGAAGTTGCTTAAGAGAGAAACATATCAGTTTGTATAGTACTATCATTTAGTACAGTTCAGCTACTTGTTT

TGTAAGAAATATATGGGATTGATTTCCATTGTTTGTTGTGGAAGGATCAGTCATATTGGATTTACATGAGTTAATGTACT

AATTGTTTTGTGCTTTGGTTTGTGAATTTGCTTCATGTAATTTGCAAGGTTAAGATGGTTTTGATGATCTATTGTAGTGA

AGCTGGTTTCTAGGCTGCTTGTCTAGTCGTGCTTCCTTTTCAGTCACCATTCTCCAGTAA

**>CaCBL2**

CTTCTTGAAAACGGAGACCAAGGAGGAATCACACAACTAGAATGGCGCCTACCGACCAGCTTTACTAGAACTCTGCAATA

TATCATCGAAACCATTTTAGCCTTGCAAATTAGAAAACCGAAATTCACATACCAATACACTATATAATAATTATATCAAC

TCTCAATTACAAAACACAAAACCACTATGGATAGGTAACACCAACATACATCCTCCATATAAGTTTCTTGCTATAGACCA

AACAACTAAATTGCAATAACTCTTAACGATACTATATGAACTCATCGGGCAGGATTTCACTAAACTACATTTCCAACCAC

ACAATTAAAAAGCCTAGGAGAAAATGAAGGAAGTTGATGGAGGAACAGTAATAAATAATGACAAGTTACTTCTTTGATCA

AAATGACATGATTCATGCAGAGGATTTTGGCATTTAAAAGTTAAAAGGCAAGAAAATAATACTACTAGTTTTTTTTGTTC

TTTTCATTTTCTTAATTGTATACTAGCCTAAATGATTCAAAGACCAGAACTTCAACTAAATTTGAAATTGAGGAAAACCT

NNNNNNNNNNNNNNNNNNNNNNNNNNNNNNNNNNNNNNNNNNNNNNNNNNNNNNNNNNNNNNNNNNNNNNNNNNNNNNNN

NNNNNNNNNNNNNNNNNNNNNNNNNNNNNNNNNNNNNNNNNNNNNNNNNNNNNNNNNNNNNNNNNNNNNNNNNNNNNNNN

NNNNNNNNNNNNNNNNNNNNNNNNNNNNNNNNNNNNNNNNNNNNNNNNNNNNNNNNNNNNNNNNNNNNNNNNNNNNNNNN

NNNNNNNNNNNNNNNNNNNNNNNNNNNNNNNNNNNNNNNNNNNNNNNNNNNNNNNNNNNNNNNNNNNNNNNNNNNNNNNN

NNNNNNNNNNNNNNNNNNNNNNNNNNNNNNNNNNNNNNNNNNNNNNNNNNNNNNNNNNNNNNNNNNNNNNNNNNNNNNNN

NNNNNNNNNNNNNNNNNNNNNNNNNNNNNNNNNNNNNNNNNNNNNNNNNNNNNNNNNNNNNNNNNNNNNNNNNNNNNNNN

NNNNNNNNNNNNNNNNNNNNNNNNNNNNNNNNNNNNNNNNNNNNNNNNNNNNNNNNNNNNNNNNNNNNNNNNNNNNNNNN

NNNTATCCCAGATGAAATTTCCCTTCCTTTAAACAGAACTTTAATTTCTGAATAAATACAAAGTTCTTCAAAGAGAAAAA

TAGAGTGTTTTCAAGAATTGATATTTATATAATGTATATAGAAAATTGTATATATTTTTGTGTTTATATATAGGAGAGGA

AAATAGTGGGAGAAACCAAAATTGAAGACACCCCAAGAAGGAGAATGAGAAAGAAGAAGAAAACTAACAGAATTTATATA

AAAATATGGTTGGAGAGTGAAGAACACGCTCGACAAAAGAGCGTGGATGATTAGGAAGTTAGGACGAGGAGTTTACCGCG

GTTTAACCACGGGTAAAAAATTTGTTTTATTGGATAAGGTTAATTACTAATTATGTAATT

**>CaCBL3**

TTTATTATATTATCTTTGTGTTGTAAAGTCTCCCTATCCATGTATATGGAGAGAGAATACTAGTTATATAGTATAATTTT

ATTTATTTTTATTTTATTACGTTACTTGTAATCTATTTGAAAAATAATACTCCTCCTTAGCAAAAAAGTAGTTCGGTGTA

TTAAAGTTTTTGTCATTTACGGTGTTTGGAGAAGAGTCGGGCCACAAAGATATACTGTATGTAGCATCACCTTATATTTC

TGCATAGGGATATTTTCACGGATTGAACTCATGACCTCCTGGTCACACGAGATTTGTAATCAGTTTGAAAAATAATACTC

CCACTAAACAAAAAAGGGCAGCTCGGTATATTAAAATTTTCGTTATGTACAGTGCTCAGTGAAGGGTCGGGCCACAAGGA

TCTACTGTTCGCAGCTTCACCTTACATTTCTGCATGGGAATGTTTTCACGGATTGAACCCATGATCTCCTCGTCACATGA

GATTTGTAATCAGTTTGAAAAATAATACTCCTACTAACTAAATTAAAAAGGGTAGCTCGGTGTATTAAAGTTTCTGCTAT

GTGCGGTGCTCGGTGAAGGGTCGGGCCGCAAGGATCTACTGTACGTAGCCTCATCTTACATTTCTGTAAGCGGTCGTTTC

CACGGCTTGAACCCATGACCTCCTCGTCACATGAGATCTGTAATCTGTTTGAAAAATAATACTCCCACTAAACAAAAAAA

GGGCAGCTCAGTGTATTAAAATTTTCGTTATGCGCAATGCTCGGATAAGGGTCGGGCTATAAGAATCTACTGTACGTAGC

TTCACCTCATATTTCTACAAAGAATTATTTCCATGGCTTGACCACACGACCCCCTGCTAACATGTTAGTAATTTTTTCGG

TTACTTCAATACTCCTGTTAATATCTTTTGAAAATATCTAGAATGCTTGCTTTTGACTTTGAAACAAAGTGTAATTGCTA

ATAATAGCTACATTTTACTAGCCTTAATGACTTTAATAATATTGAAAATTGACAATATGTTAATAAACATTTTTGACAAT

TTGAATTTATGGACAATGTTATACTCCAACTATTTTCATTGTACACCAATATTTATTACATAATCTTAAAAATAAGACAG

ATTAATTATTATTATAGGTAAAAGTAAGTTGATGATGTTAAGAAAAAAAGTTAGTTCAACAAGAAGTAACTGTATTTAAG

TAGTTAATTTACTGAAATTAAATAAAACATTCAATCAATTTTTAGAGAGGTTTGATTGTTTTGTATAGATTAAAAAAGTA

TAATCGGAAATTAAAAAGTTCGTAGTTTTTAAACAGTTAGAGAGATCATACTTTTATAAAATAAAATTATATTTTCTTAA

CATATCCTCACGTGCGAGGTAGAATTTTTATTTTTATTTTTTTTACATTTTTATTAGGGTATAAGGTTAGTCGAGTGGGA

GTGTTGTCTTACTTTTCGAGGGTTTAGGCTCGTTGATGTTTGTCATGATCCTAGTTTGTT

**>CaCBL4**

CAAAAGACGAACAATAAAATGTTGTTAATGAACAATATATCCAAGATCGTTAAACTTCATTGAACAGCACCTGACAATAG

AAATACAAGAGAACTTCAACCCCAGAAGGAATGCATCATGATCATCAGAGTATTGAGCAAATAAGTCATGCATAATAAAT

ATATCGAAGTCAAAATGATACAGCAGAATAGCAGAAAAAAAAGGAAATCATTAAGGACGACTAGATAAAGTCTTAAAAGC

TAAGTAGGTCCATCACCTCGTCAAGCTTTGCACCCTCTTCACAATTTGGGGTGGCCAAACAAAATTGAAGGAGATAACTA

TCCCTAGCTAAGGTCAAATTCTATATCAAACCATTCGTAAAAATCGCAAGTTAGGGAATTGCACCCTTAAGAGTGCTCTG

AGAACTATATGTTTGTGTTACTTTATCAGGTTCAGGATAATCACCGGGACATCAAATGTATCGCTTTCTTAATTAAGCTA

AAAACAAGGATAAAGAAAAAAAAAGGATCAAAGTTCTAGCTCAATAGCAATTGCCTTCACTCATGCTAGCATTAGATGTT

TAAGACTTAAAAGACTGCTGATCTGCATGATCATCAAATATAAGGGTATATAGCAATATGACATTCAAATTTCTTCACTA

CAACTCACCAAAAGCTTACTTCAAAACCTGCACCTTATTTCTTCAGCAGCACTATAACATTCTGTTTTCGCTTAGCCGTG

TGTTCGTGACAATGCAAAGAGATCCTTCTCTTCCTCGCCAGATAAACTGAGCTACTGATATGTATGGAACTAAAATACAG

ATAGAAAGCTTGTTTGCATTTGGAGAGACCAAAGTTTAAGGGCCCAAATGCAACTAGCAAAGGAACATTATTTAAGTCAC

AAATTCATATGGGACTGAGGGCACTAAAGTTAGGGGAGGGCAGGACTACAGACAGCCTTACCCTGCCTTTCCGCAAGAGT

TTGTTTCCACAGCTTGAACGTGTGACCTCTTGGTCACATGACAACAACTTTATCAGTTAATCCAAAGCTCTCCTTCCAAC

AAATTCAGATGGGACATATGTATAAAATGATTATTACCATTACGAAAAGTCTGTACCACAAGGGAAAATTTATTTTAGAA

AAATCCATGGTTATCCACAGAAAGGCAATATATAGTTATTTATCTTTTTTTAGATGAATATCAAGAAGCCAAATACGTAA

GCAAATCTAACCTCTTCTCACAAGTTTGCCTCTTGGAGTATTATGAATATTTAGATAACTTAATCACTGAAAATGTAAAT

ATTTATCTATAAATCAGGAAACTAAAGCTAGTTTTTGTTGCCATTGATACACTGGACTCTATCATCAAAGATCAAGAAAA

TGCTTACAACCAGGTAATAGAAAGACATATATAGAGAGAATAATAAAAGATTTACTTACCTAGTAATTAATGTTGTTGCA

TTGCTGGTTAAATTGTGAAAGAGCAAGTTTACAACTAAGAGCACAGATATATGTAACTTT

**>CaCBL5**

AACATGCCTTAAATTACTCATAATAACTTATTAGAAGTGATATAACCAAATTATTATAAAAAATAATTGTGAAAGAAACT

TAAGTGGTCCTCATGTAAGGTGTCTAGTTAAGTTAAAACATTAATAGTTAATTTATCATCATTTTTTTTTGTTAGCTATT

TTACCTTTTTTGTTAAATATTATTAATTTTTAATACTTAAATGACTTAAATAATTAATCAAGGGAGAAATAATAAAATTG

CAGTTGAAGCAGTTAAAGATTTTTTATTATTATTAAATATTATTAATTTTCTAATACATAAATGACATATAATAATTAAT

TAGGGGTGATATAGTAAAATCACATAGCAAACAGACACGTCGATGAAGAGGTGTCTGCTTTACCGCACTTCTATATAGTA

CTAACGATATATATTATTTTCTTATCTTAATTTATGTGACGCAAATAAAATTTAGATAATCAATTATATTTTTAGATATT

TTAATTGTTAGCTATTGTAATTTACAATACTTTTTTGGGCATTATAATTTATTATTTTAAGTTGTTAACTACTGTAATTT

ATAATACTCTCATTGTCCAAACTTTTGTGGCATTCATACAATCATAGTCAATTATATTTATAAAATAATTTAAGTTTTTT

ATTCTATTCCAATTTCAGTGATACATATAATTTTGACAGTCAATCAAATATGTTAAATTACTCATAATAACTCAATAGAA

GTGACATAACAAAATTACCATAAAAATTAGTGTGAAAAAAATTAAGTGGACCTTATATTAGATGTCAAGTTAATTCAAAA

CATATAGTTAAGTTATCACTTTTTTCTATTTTAGCTTTTTTATTAAATATTATTAATTTTTTAATACTTAATGATTCAAG

TAATTNNNNNNNNNNNNNNNNNNNNNNNNNNNNNNNNNNNNNNNNNNNNNNNNNNNNNNNNNNNNNNNNNNNNNNNNNNN

NNNNNNNNNNNNNNNNNNNNNNNNNNNNNNNNNNNNNNNNNNNNNNNNNNNNNNNNNNNNNNNNNNNNNNNNNNNNNNNN

NNNNNNNNNNNNNNNNNNNNNNNNNNNNNNNNNNNNNNNNNNNNNNNNNNNNNNNNNNNNNNNNNNNNNNNNNNNNNNNN

NNNNNNNNNNNNNNNNNNNNNNNNNNNNNNNNNNNNNNNNNNNNNNNNNNNNNNNNNNNNNNNNNNNNNNNNNNNNNNNN

NNNNNNNNNNNNNNNNNNNNNNNNNNNNNNNNNNNNNNNNNNNNNNNNNNNNNNNNNNNNNNNNNNNNNNNNNNNNNNNN

NNNNNNNNNNNNNNNNNNNNNNNNNNNNNNNNNNNNNNNNNNNNNNNNNNNNNNNNNNNNNNNNNNNNNNNNNNNNNNNN

NNNNNNNNNGATGGAAACCCAAAAAAAAATATATAATAAAATCAAACAATAAGCAGGCACAAATGGCTTTCTCCTTCTGT

ATATTAGAATTAGAATACCAAACGACCCGTCAGTATTAATATTGATATAAATTTTAAGAC

**>CaCBL6**

AATCAGATTTATGGATACATAACCTGTGTTATAATTTCCAAAAAACCCGTGTTATAATTATCCAAATAGAGAAACATAAT

CTTAATTATCCTAATAGATTAACATAATCATAATGTTCAATTAAGTTATAAATGCAAAAATACGTTTTTTCTAAGTTAGT

CAAGTGTAATTTATTAGATTGTTGACCCATAGAATATTCTTTCCTTACAGTGTTAAGTATCTTCCACTTCCACGCTCTAA

AAGAACGCGTGTCATCACTCGCCATACTTGTTTTAGTTTTGCCCCCAATTTTCTCCAAATTTGACCTCTCTTCACTCTAT

TTCTCTCTTACTTCATGTACAAGTATATGTGTATACATACATTTCTTCGAAGTTCACGTTTGAGCTTTACCTTTTACTTC

GGACTTTTCTGTTTTGTCTGTTCATTGGACGGACTGATCAGTGTGTTTCCTTTTTTTCAAAAATTTTCTGTGTTGTTATG

CTCTCTCACATGCATGAATTTTTTCTGTGGTGTTTGTTTTTATACTTCGTTTGCATTCTTAGAATTTAATTTGGTAATGA

GTTCTGAATGTAACTCAACTGATTCAAGAGTGAGGTGTAGTTGTTCTTGATGGGTCATGAAAAGAGAGAAAAAAGTGTGT

GTGTGTGTGTGGTGGTGGGGTTGGGGATAGGGGGTATGGATACAGCAACAACAACAATATACCCTGTGTAATCTGACAAG

TGGGGTTTCGGGAGGGTAGGATGTACATAGAGCTTACCGCTACCTACATTGTAAGGTGGTGGGGCGTTACAACAAGTTTT

GAAATTTGTGTAGTTTGGTNNNNNNNNNNNNNNNNNNNNNNNNNNNNNNNNNNNNNNNNNNNNNNNNNNNNNNNNNNNNN

NNNNNNNNNNNNNNNNNNNNNNNNNNNNNNNNNNNNNNNNNNNNNNNNNNNNNNNNNNNNNNNNNNNNNNNNNNNNNNTG

ATTGGGAGGTAGGGCAAATGGATAAATATAAAATAAGGATATGCAGTCAGACCTCCCTATAGCGTCATTGTACTATCACG

AGCAACTCTTTTAGAGCGAATTGTTCATGTTATGTTGTATTATACGTTCTCTACAACACCCAGACCCCACTTGTGTAATG

TGGGATTACACTGGGTATGTTGTTGTCTCTGTAAGCATTTGAAAGTGACACCGTTATAGAGAAGTTTGACTATACTATTC

GTAAGTTATATAAATTGGAAGTATGAAGTTCTGAATTGTCAACGTTTTCTGTTGTAGCATGACATGCTCATTTTTTTAGG

TTAAGAATGCGCCCATATTATAGATAGTTATGACTTATCCTTAATTGGATTATAAGCATTATTTTCCTTTATCCGGAAGA

TTTAATTCCTAAATTCTCTTGCTTGAATTTGACCGGTACAATTGCTTTGATAAGATGATTTGATGATCTATTGTAGTGAA

GCTGGTTGCTAGGCGCTAGACTAATCGCATTTTTACTCCTTGGCCTCCATTCTTCAGTAA

**>CaCBL7**

ATTTGATGTGGGGCTGTTCTAATTTTGTTGTTTAACCAATAAAATTTTGAAATTTGAGCTAAAATTTGAGTTTTAGATAT

AAAGTTTCAATTTTTAAAGAATTCAATTGAAATTTTTATGTGATGTACAAGTGTGCTTTTGTGTAATTTGATGTGGGGTT

GTTCTAATTTTGTTGTTTAAGTTTTGAAATTTAAGCTGAAAATTGAGTTTTAGATACAAAGTTTCAAATTTTCAAGAATT

GAATTCTGATCATTCAATGAGCCTTGCAGAAGGGATGTGGTGATATACAAGTGTTTTTTTGTTCTCTGTGTGTGTGTGTG

TGTGTGTGTAATTTGATGTGGGGTTTCTTTAATTTTGTTGTTTAGTCAATAAAGTTACGAAATTTGAGTTAAGATATAAA

GTTTCAATTTTTCAAGAATTCGATCCTGATCATTCAATGAGCCCTGCGGAAGGGAATGTGATTTTGGCGCGAACAGATTA

TCTGCAGAAGGGAATGTGATTTTGGCGCGAACAGATTATCCGAGTTGTTTTTGAAAATTCTCGTTTAATTCGTTTACGAA

TTAATCATCAAGGTGGCTGTATTGCTTATTGGGAGTTTAATAGTTGGATTAGGGAATGTGATTTTGTCGCGAACAGATTA

TCCAAGTTGTTTTTGCAAGTTCTCGTTTACGTGTTTACGAATTAATCATCAAGTTGAATGTATTGCTTATTGGGAAATGT

GATTTTGGTGCGACCAGATTATCCGAGTTGTTTTTGTAAATTCTCATTTAATTCGTTTACGAATTAATCATCAAGGTGGA

TGTATTGCTTATTGGGAGGGGATTAGTTGGATTTTTGTTTCCTTTATATTTATAGAAGAATGATCTTTTGGCGATCTTTG

GGGTTAGTATCAGTGTTAAGTTTGCACATTAGTACCGATAAGAAGTTCAGCATGTTGAATCTGAAATCTGTGAACTATAC

GTCAATTAGCAGAATGAAGGATAGGGGAGTGGGATTGCAGAATGATTAGTTGCAGAAGAGGAAAAAGTTTCAATGCTTCA

ATTTTTTGTTCGGATGATGGAACCAGAGTGAAGTGGTTTCATCTTTATTGAAGTTGAAAACATATATTAAGTTGTGATAA

TAACTCTGCGGAATTTATATTGCATGAAAAGAGGATAATCAATACTGATATTTAAGTTGAAAGATTTGACCTTTCTGAAG

GAATTAGTGGGTGTCCTTTTGTGCTTCTTTTATCAGCTTCAATAGACATTTATGGAAATTAATTGTGTATTCACGTGTTT

TCCTTGGCAGATATATCGCTGCATATAGATAGGTTTAAGAAACTCAGTAGTTTGGGTTCATACATAGACAAAACAGAGAA

TGGTCAAAAGGTTAGTTTTGTTGTTCATGTCTTAGAGATATATCGCCGTTACAAATGACCCAATTATAATGGATTTTTGA

CTCCTTCATCTGTCATATTTGTTGAACAGATATGGAGTGTTTTGAAGCTGAGACTCTACC

**>CaCBL8**

CTCTGTGGGTGTGGAGGGCGCGAATTAGTGTAGATGGTTAGTGGGTAGGAGTGTGTCCTGACTAGTAGGGAGGAGTGCTT

TGTTTGTTGTTGTGCCTGTAGTCGTAGTGGTATGCGTGTGTAGACTAGTCCCTTGTTCGTGGCGTTTTGCTGATGGTTGT

GATTACGGATAGCTAGTTTACAGTAGTACTCCGTGTGTGTCTGATTTTCTTTTATTATTTAGTGGTGTGCTATCCCTTTT

TGTTGTCTGTTTTTATGATTTTTGTTTGTTTGTTTGCTATACGGTTAGTTGTCCTGTGTCGGGGGTTTATCGGAAACATC

CTCTCTACTTCATTTGTGGTAGCGGTATGGACTGCGTGCACTTTATCCTCCCCAAACCCTACTTTGCGGGAATACACTGG

GTATGTTGTCGTCGTTGTTGTTGTTGTGGTTAACGTTTCTAAGATATTTTCCGGTGTCAGTTTACTAAGAACCAAATTGA

CCTGAAATACAAGTATGAAGATTGTCTGACTTGGAAAAAGTATAACAATTGTGCCCTTTAATGTGTCCTTTTTCTGATGT

TTACCGTTTAACTAAGCTGTTCAAGAATTCCAGTGTCAGTTGACTCGAAACGTATTTGATATCATAAACAAGGCCGTTTT

ACGGCTGTATAAAGTGGTATACTAAAAGGTAAAGAATCTAGACAATCAACTTATTCCCGACAAACTTCACCCAATCGTCT

ATACAATAGAGGACTTTCAGAGTGCACCGAAAGAAAATAAGGGAACGAAGGCTACTTCTTAACCTACGGAAACTCGACAC

CTTCGAAAGCTCTTCTATTCCTCTCTCTCTCTCTCCAGGCATCCCATCTTAATGTGAGTGGCGTCACGCCATATTCCATC

CCCTCAACACGCATTTTTAGGCTGATGCTACAAGAGTAAAGGTGCCATGTTGCTCGGACTCCTCAAAAATGTCGTTGGGT

GTGTGTCCAAAAATAGTGTATTTTTGAAGGATCCGACACGGGTGCGGCAACATTTTTAGAGATCCGAGCAACTTTTTAGG

TGTCTATTGCAAGTGAAACTCCAGTTGCTTGTGATGGTGTAGCAGAATTTTAAATTGCACAATAAGAGGATAGAGTCATC

GAGATCGAGATATTTAGTAACTTTCTCTTCATATGTTTTGCGTTGGCTGACAGAGTTAGAGGGTCTTTTCACTTGATTTG

CTTATTATCGAGGGGAGGGATGATATTTTAAATGTGTATTGACATGGGATCCTCCTTGTTAGGTGTGTAGCTCCATAATA

GGTAACTTGCAAAAGACGTTGGCGTTCTGCATTCAAATAAGCAAATAGAAAAGAATGGTCACGAGGTTTGTTCAATTCCT

ACGTTTGGCTGCTTTCAGTAACTTCGTTTTTTCTTCCGTATGCATAAAAGACCAAGGAAATTGACCTCTTCACCTCCTCG

TGCGAACCTGCTTTTTTTTGGTTGAACAGCTATGGCGAGCTTTGAAGTACGGAAGATACC

**>CaCBL9**

TATTCCGCACGGGACTTAAAGATCCAGAAATATATTTTTGTTCACCTGCCACATCCAACATGAAAATTATAGTAAAGCAT

TGGCCATAGAAGTTAGAGTATTTAGATAAAGTAATCAGAATCCGAGTACAAGAAAAGAACACTACTGAATTTACGCACAA

GTTTTATCTTCTTACAAACACTAAGTGCATTATCGGGCATCAATTACTTTACTTTACGGGGGATCATAGTCAATGTTAGG

TTTTCCAGTGTTCCGAGGGGGAAATTCAGCATCCCCAATAAAGCAAAGCATATAAACTAGGATAGTTTCTGGTTCTAGAA

AAGAACTTGTTATCGACGATGTTTGATACGAAAATGTTTTTCATGAAAAATGTTTTCTTTATAAGCAAATAAAAATAATT

TTCTTACTTACTTTATAGTGTTTGGCAAATAAGAAAGTGATATTGTTCTAAGAACATTTATATATAATCTTATGCAAACA

TTATGGGGCTCGGTGGGGGTGGGGCAGGACAACCAACATGAAATGTCACTTTTGAAACTTTAGAATTAAGTTATAATCCT

GTTTTCCTTGTAACTATTTTGCTAGAGAAGAATTTCAAAATATTTTGACTAACAAAACATGAGAAAAATCCATACCAGAC

ACACATTCCCAAAGTATTCACAATTTTAACAGTTTTGTAAAATCCATACCAGACACACACTCCCAAAGTATTCACAATTT

TAATAGTTTTGTAAGTACTAAGCCATGAAATACATCTTACCCAAAAATTGCTGCTGAACATACTGATTATAGAGGCGGAT

CTAGAGCAAGTCCAACATGTAAGATTGAACCAAATGCTCCGTTTATAAATGAAAAACTTAAGTATAGATACTATTATCCA

TTTTAGATGGTTGATCTCTTAAATGGTCACTCAACTACTGGTTATTATCTCATATTCCATATTTCTTTGAAAAAAAAGTG

ACTTTGTGAGATACTCTCCTGTCTCAATGTATGTGGCACTTTTTGAATTTCGAGATCCAAATAAGTTTATCTTTGACTAT

TTTTTTTCATATATTTTTAAATATTTTAAAATTATCAATATTGTAACTAATACTACTTTTTAAGTATTTTCCAAACATAT

AAATTTTGTTTCCTAAAAATTGAAGATTCTATGCCCCAATTCATGGTCAAAATTAAACTGTTTGACTCTCGAAATTCATA

AATTAAATAAAGGAATGTAATAACTATTAATTGAGTGCTCTAAATCATGGATTCACCATTGATGTAGGACTCAAGAAAGA

TTGCATTTATCCATATAAAAGAAGCAAATAGGAGAAAACCCAAAATCTTGTAAAGAAGGGGATCCAGAGCAAGTTCAATG

GCATAATTGAGCCAAATACCCAGCGGTACATGAAAAATTAAAAAATATACATTCAGAGAAATCAACATGCATTTTGATCA

CTCAACTAACCATTATTATTCTGAGTCACTTATTTCTTGATTCCAACTTTTTATTCAGCA

**>CaCIPK1**

AACAATGGATGCAATGGACAAGCTTATGATGTTTGTGGACTGATTTACAGCTGATCGCCGAAAAATAACGTCGGAAAAGT

GGCCGGAATCGGAGCTTTTTAGAGGGAAAAATAACCACCTTCTATTTTTAGCTTTTGAAAAAAAAATTTAATATTTTTGG

AAAACCTAGATATTTTTGTATATATAATGGTGGAGGATGGAGTGGGTTGATGTGTATTATTAGAAACTTGTGGGTGAATT

TATTAAGTTGGAAGTATTGGATTAAAGTGAATTATTATAAAAGTTGAGGCTGAAATTGTTAAATTGGAAAAGTTGGTTAT

GAGGTGGAATTATGTGGGTATTTGACCAATATTTTAAAACTTTTGAATATTTGGCAAAATAATTTTGAAAAAGAGGTGTT

TTGACATACTTGCCCAAGTTAAACACTAGCAAGAACATGAAAATACAAAAATACACAGGAAAGAAGGTTTACAATTCATG

CACAATCGGGTAAAACAACAGTACATGCAACAAGAACCATGGACCATTGAGTATGCGGGTGTTTTTACCTCCTTGAGATG

CAATTTAAAATCATATGCTACGAAATCCAACGGAAGAATCATAAATGGTGACACTTCAACTGGAAGTTCAATAATTTGAG

CTGGGTTTATTTAAGGTTTGAAGGGGAAAGGTAAATCTGTCAACTCTTTACTCCATTGTTATTTGACACATAGAGATAAT

TAGAGGCATGGAACAAGTTCTCTCAAACTCTCGTGGGTCATTTGGTATCCCGAATAAGAAAATAATCTCGAGATAAAATT

TGGAATTAATTTTTATATCACAACCCTAAAATTATTTTATCCGAACATTATACTAATGAGTTTACGGTCACATTACATAC

AAAGGGTCAGATAAAATAATATATGGATATCCTACTATACCAAACGACCTCAGTGAAAAGAGTACCACACTTGTGCACGT

GTCAGCAACAAACTACTTTTCCGTCCTACGAACAAAGTCTTACTTTGAAAAAATGAGAGGTGACCCACTAAGTATCATTA

TTAAAAAGATTAAAATGAATAATTCAGTCTGACAGCGCAACAATGCGTAGATGAAGTAACATTATCTACTAACACCATGC

GCATGACCTTAATAAATTTTGGAAATGGTCTTTATCAAATCTAGTTCAACATTCCTTTATATTCTCAGTGTCATCAGGAG

TAAGGTATCATTCATTTGCATTTGTCTTTAGCCTTGTTCAAAAGGCTATTTTACTTTTCTTTCAACAAACAATATTCATC

TTTTTTCTCTAATCAATGTCCTTTCCAAGTTCTAATCTTAGTTCTGGAATCCACAAAAACTATCAACACTACTCGTTCTT

TGATCATCAACTTCTTCCAACTACTTCTCTTTCTCACAATAGGCCGCGCAAAGAATTGATTCTACTAATTCTGTAGCTAG

CAATCATTGTATATAATTTTAAAAAAAGTGAGTCATTAATATTATTCCTGATTCAAAATGATGGAG

**>CaCIPK2**

TTCTTAACAAAATCATAGATTTGAGCTAAAGATACTCAATTTTGTCGAACTCTTAAGTAAGGGGGCTCTGCCCGTGTTCA

CTCCCTACGTTTTATTTTATTCGTCTCAAATTTCTTAATTTAATTTTTCATTTTACTTGTTATTTCTACCAAGATAATTT

TTTTTTTTTTTTACCCGTAGTATTAATTTTTTTTTACAAATTAAAATATAAATATCATTTAATAGGATATTATGGTAAAG

TAATCATGTTATTAACTGTGTTTCTTAATCAATGTCTCAATGTTAAATTGGAACAGTAAAATGAGACGGAGGGAGTAGAT

TCATTTCTAGCTGGATTACTCTAATGATTATAAGCTAAAAAGTTAAAAGACGTGGTTGGATATCATTAATATATTATTTA

TAAGTATCAAATGAATTGTTTAACATAATTATAGGATTTGATATAATACTACTGAATTTTATCGAAGCTGTGACTAAAGG

GTGGTTTATTGCTGATTATAATCCAAAAAGTTAAAAGGTGGTTGGATCATTGATTGCCCCAATAATTCTTTGCTTTTTAT

TCTCTCTAGAATTTTTTAGTACTAATTTCCAATAAATGTATATTATATATGACTTAATTTGCTAAACTGGTGTTGTCACC

TTTGGTTCTCACCTTTAGTTTTTAAAATATTAGGGAAATAAATTAATTCTTTAAAATATTAGGGAAATAAATTAATTAAG

GATTTGGATATGATGGACTCGTGATTGCCATTAACGGCTTTATTTCTCTCTTTTGGAAGGATTAGTTTGAGACCAAAATT

TTTCACAAATTACATTTGTTCCCAACTTGCAATAAGGGGCTTCTTTTATGTGGAATTAGCTAAGTATATATTGGAAGGAT

CTTAATTTCACCATGTACGTAGCAGGATGGAGCCAAGTAGATGAACTTCTTGGTGGAAAATTACACTGTTTATACATTGT

TAAAAGTATCGTTCATGTATATATAAACATATGTTTAACCCCCTTCAGCTTCTTCATTTGTTTACTTCTTCATATTTTGG

ACCCCTTAATGAAAATAGCGGCTGAAAATCCTGGCTCCATCATTCGTTTGCCATTAACTGCTTTATTCTCTTTCTTTTAT

TTTCTTTGGCTTTGGAGAAAACATACTTTTTGGAGGGGTTATTTTGAGATCAAAATTCTTCACAAGTTACATTAGTTCCC

AACTTGCAGTAAGGGACTTTATTGCAAGTATATATTGGAAGGATCTTTATTTCACTATGTAAGTAGCAGGGGTGGAGCCA

AGTAGATGAACCCTGTGGTTAAAATTTATCTTTTATGTATATGCAAAAAATGTTGAACCTCCTTCACCTTCATTGTGTGT

TTACTTTTCATATTTTGAATCCCCTTAATAAAAATTCTGGCTCCGCCAGTATGAAGTAGTAGAGTCATTTGTGTATACAG

TAATCTTCTATCTAACAACACTTTCTACCTTGTACAGAATTGTGCTGGTGTCTCGATAATGGATCG

**>CaCIPK3**

GAAATACCTCAACCCTGGAATCATCCTCATTATTGATTACAACCGTTGCTTGATTTGCTCTAGTCGTCACTAATTATTTC

CTGCTTAATAATTTACATTTTTATTTATAATATCAAAATCATCTTGATACTTTCAAACACTTTATACCCAATTTTCTTAA

ACTAAAGATTGAAATAATTTCTAATTTCTTGATCCTCGTGGGAACGATATCAGACTATCTAAGTCATTATATTACTTGCA

CGATCGCGTACACTTGCGTGTGCGTAGAGACAAAATAATTTGATATATAGTAGACCCTCTAACTCGTCATATTCTAGAAG

TAATAGTCGATGAAAGTTGACCTAAATAAGAGCATCTGCTAAAACTCTATTGATAACAAGTGCTCTACTCATCTTTTAGT

GAAGAGACCTCTATGATCTCAATTCACGTACAAATAGATTCCCACACTACTTAATTGATAAACATACTTATATTCACATA

GGAGTCATTGGTTTAGGGACGAAATGACGATTTAAGCTCTTGCCTGAATAAGCGGGTTCTTACAATGACATAGAATAAAG

TGGGTATTTGACAAATTTTTCTCTAAATGGCAAAATAAGCTTTAAAAGTAGGTATTTTACTAACTTTACCTAGAAATTTA

TGGGTTTTCCACATCACACAAAAATAATCAAACGGTAACAAAGGAAATTTGAACCAAGAATCAAGAGGACAATAAAATTT

TTACAAGTCCCTTTACTTCACTATACCAAACAGCACNTTATAATTATATTATATATTTAATATATTTCTCTATATAAATA

TCAAATCCGCAAGAAAGTTTTTGATTTTTCGAGAACTCCCATCCAACCCCTGGTCTGGCCGTGGTGTTTAGAAAATGAAT

TATTTCATTATACAAAGATAAATTATATTGGATAAAGTGAACGTGCATGTATTCACCTGCTACTGGTACATTATTGTGTA

TATTTTATCCATCGCTAGCTGGAGATCATCCATTAGTTATCAGTGCTTACTTGTTCTGTTTCATTCCCTAGATTATCTGG

TCTATAAATAATCCTAATATTTATTCTTAGGTACGAAACTTCCTCTGACCAAAAAAAAAAAAAGAGATGCGAAACTCCGT

AGAAAAATGGTAGGTAAACAAAGTCTCTTTGTACTATAATACCTGTAGAAAGGGTATAAGAATTCCTCCGTATATCTGAA

TCTTTTTCCTCTTCTCTCCTCTCCTCAAGTCTTTGCTTCAAGAAAAGCCAAATATTATCTTCTTTGTTTTGCAGTTCTAT

TAATCGAATACCCCCATTAGCATCTTTAGCAAGTTCCCCTCATTCCTAAGTGATTTTTTTTTTATTTCTGGTTTTGATTG

ACGAAAAGGAGTACCGGAAAGATTGAAGTGTTAACAATTCAGATATCAAGCAAATTAGGTTTGCTCGGTGTTTTAATGCA

AAGGGTAGAGAACATTCACAAAGCAGGGGTTACTGTTTTACTGGTCTGTGGGTATAGTTATGCCAG

**>CaCIPK4**

ATAAGGTGGGTGTGTCTTCGGTGGAAGCCAAGATAAGAGAAGCGAGGTTGAGATGGTTCAATCATGTGATGAGGAGGAGT

ACGGATGCCCTAGTGCGGAGGTGTGAGAGGTTGGCTAGGGATAGTTTTCAAGCGTGGTAGAGGTAGACTGAAGAAATATT

GGAGGGAGGTGATTAGACATGATATGGAGCAGCTCCAGCTTATCGAGGACATGACCCTGGATAGGAGTTGTGGGGGAGGC

GGATTAGGGTAGTAGGTTAGTCTGAGTTTAGGGTATGGTATCTTTTGATGTGGTACTTGGAGTATTTTGTTTATGGTATT

ATTGAGTTTGCTAATCCCTATTATATCTTCTTCTTTTACTAGTCTTACTATTACTTTTCCTTATCTTAGATTGTTCTATT

TTGAGCCGGGATCTATCGGAAACAACCTCTCTATCTCACTTTTGAGGTAGTGGTATGGACTGCGTACACTTACCCTCCCC

AGACCCACTTGGTGGAAATATACTGGATATGTTGTTGTTGTATAATCAATAATTTAAAATATCTAAAATCGAATAAGAAA

TTTAATTGACTCTCTAAATTATGTGTATTACAAAAAAAATTTAATTGACTCTATCAAATTTATCTATATATACAAAATGA

CATATATAGCACGGATTTTTATGTTTAGTTCTAGCTGTTTTCCCTGTACAATGAGAATGTGACACATATTATTCCTTCTT

AATTACTGAATGAAAATTCAATCAATACTTATTTTCTAAACATGCAGTCCCGGGCAGTAACTTCCTCAGTTCATTTCTTC

TCATTCGCAGCTTCACCCTTCCCCGAAAGCACGCAGCTCTCCAATAATATATCTTTACATTTTCTCTCATAATAAATATT

TTATACTAACTAATAGAAGTGGTACCTTTTTTCTTCTTGCTCAGCCAATTTTACTTTGAAGGAAAATAAACCACCATTGA

AGACATCCCATCAGTTTCGCCGCCTTTCTTGTCCTTTCTTTCTCTCATTCATAATAATTATAATCATTTTTTCCTTATGA

AAAGTAAAGAGNTTTTTTGCCCTTTCACATCACCAAAAAGAGTCATGTAAGAGAATCTAGCTTCCCATTTGAGCAATCCG

ATTTACTATTATCCAAATAATTTTCTTTAATGGATTTTTGTGTTTTCCGTTTCACTTTCCTAAAGGGTAATGGATTCTCA

AACATAGCCTTTTCTGTCCAACTTCTCGTACACTGATCCATTTCAATAGTCTATTCTAATTTCGGGATTCTCTTTACCTT

TATTTTTTGTGTGTGTGTGAAAATTTTAAGATGAGTGTAGCCAAGTCCCAGGTTTGGCAACCTTCTAAAAAGAAGAGGAT

TTAGCTTTAGGCTTATTAAAAGGAAAAAAAAAGATTTTCTGTAGGTAAGAGGGAAAAAAAGTACAAAATATGGATATTCA

TTTAGAGTAGGGGTTTAAAGATCTGTAAGGAGATTTGATTGAATAAGGAGAAGTTTGGAGATG

**>CaCIPK5**

TAAAATGAAGAAACCGAAAGAATCAAGAAAATCGAAAGAATCTACTAAAATCAGAACAGAAAAAAATTGACTTCTTGTTG

ATTTTGGTTTTGGTAATAGATTTAATATAGCGATATAATTGATTTATTAATTCCTTAATGAAAAAATCAAATCAACTCAA

CCCGTGTACCCCTATTATTAATAGTGCTAGCTAATATTTCTTTGTCCTGATCAGGATATGGTACGATCATATCTAGCGTA

TTCATCACTTTGTCCCTTCTCGCCACATCATTGGCGCGTGCCCTTTCACATCCGAGTCACAAGTTTCTGCCTTCTTCCCG

ATGATAATGATTTCTCTAGGTCAATGACTACCTAAGATTATTCTTCCCTAGTCATGTTCTCGAGGTCGCTTAATTTCATT

GAATCCACTTTCTTAGCTATCCTTAGTTATCGCTATGTGTCCATCTTACGCACTACCACATATAAAAACTTCTTTTTATC

CGATACAATTACTGTCTTAAATTTAAGTGGTTGAATAATCGTTATTTAGGTTAATTCTTAAAACAAAAAAATAATAATTA

AAACTCCTTTCTTAGGAGTTAGGACCCAAAAATAGGATATTCCATGGAGAATTTTTCTTCAAAGAAGCACATACCATCTA

TTTTCACATCCAATAAAGTCCATCCACGTAAAAGGCAATCACATGTATATCACGTGAGTCTAGCATCATTCTTTTTATTT

CTTAAAAAGTAGAAAAAGATAAAGGGATTTCTTACTATAACTCCGTTTGCACACTTTTACTTGTCATGTTGTGCTGATCA

AAAGTCGAATTACATAAATTTTAACCAATATTTTAGAATGCATTTTCTACTCTATTAATATGAAAAGGATTTCAAGCTAT

GATGTTTTTTATATAATTCTCAAATATTTAATTTAAAATTAAAATATTAAATAAATTCACTTCAATTTAGCTTTAAAGCT

TAAAATAATAGTATGAAACATATAAATATAATCGCCCTTTTTCCCTCCTTTCCTATACAAGTTACAACCACAGCATCCCA

ATTTACGATTAAATTGACTACTTTTCCAGCTATTGAACACCCCACAACCTTATATAAAAGTTAAAACCTTATACGCCTAA

CGTTAATAACCTACACCACCCAATATAATGGCTCCAGCGTATACATAAATACACATTACACCTCTCTACACAATCATTTT

TCCTCCCTTTACAGAAACCTAAAAAATAGCTTAAAAAAACACAACAACAGCAACAACATCAACATCTCTAACTACACGAC

AGCCCACCATTTGTATGGCGAATTTCTCCATTATTTAGTCTCGACTTTCAGCTCATTCTGATAATATTTTTGGCCTTTTC

CTTTCCCTTCTTTTTCGCAAATCTTGAAACTACTTTTTTTTTTTTTTTGCAAAATCTTGGAACCACCCATTTGATTATAT

TTGCTTCTTATTTAGGAAAAATTTGAGCTTTTTACGTGTTATATAGGTTTGCAAGAATCCATG

**>CaCIPK6**

CGATACTGAATGGGCTCCGATGTTTCTAGAAATAATTTTCTAAAATATGGACCTATCAGACATGAAAAATATTAGCAAGA

AGCATGCACATGCCCCGAAAAGAGGAAACGAATTACAAAAATTCAACCACTAGAGTTGGTGTGGTGGTTCAGCACCTCAT

CCCTTAAGCAAGAGGTTGGAGGTTCGATTCCCATCTCTGGTGAATGGAGTTCCCTACCGCTTAGTGCGCTGACCGAGGAA

CGGAGGATTAGTCTCATGGCTGCCGGCTGTGGGGATACCTTGGAAAACAAAAAAAAAAATTACTAAAATTCATGGTTCTA

GAGGTACATTTCATATCTTATAGATCTAGATACATGCATATGCATGTGGATAATTTCTATCACTACGTATGTACAATTAA

TAATGGCTATCAACTTGTTTCTCTCCGCCACTAACTTCGTAGCAGATATACCGTCACCAGGCAAGAAATGTGATTCACAA

TATATATTTATTATCCTCTTTTAAAATATTTTTTCAAACAAAAAATTGATAGATAATTAGAGCTGTATCTTAACTTAGAT

GAGATAAATCCAATTTCCGAAATAATAATCACCCCATATATATTAGTAAATATGACATATCAATAAGCAAACGAGTTACG

GTGTAATCTTTGATTAATTTATAAGCTGTTTTTTTCACACCATCAAAGTAGAATAACCTCTTTAAATTGAATTTGTATTT

ATTAGACTAAAAATAACTAAAATGTAGAATAGAAAATTTCCATTTATTGTTGGAAGGTTATAAGTTTTTCGGCATGCAAT

TGTAACATTTCACTTGTATGACTAGGGGTGAAAGCTCTTGATTTTTTTATTTTTTTTTGTTTTAAAATATAACTACGTGA

ATTTGATTTAAAAAAATCAAAGTTATATAACCATTCATGACATGGACGACAATGTTGGAGAAGATTAATTAATATCAACT

TAATTAACATTATGAGCAAGGCATGCATGTGGACCTCTGATAATTGCTTACATATAAGTTTGTCTTTTCATATATATATG

ATTGGTAGTTGAGGCATGGGAGAAAAAAAAGGAAAATGAGAGGTGAAAATTGAATCGTGCATCTTAGGGTATGTTCATAT

CTTAACTAACTTTCACCAATTTTCCCCTATGCATCAAACAAAATGTGATTATTGCATCAATATTGCATTATACTATGAGA

CTTTTTGTAGGCATTTCCTCTAACCCTGCCACAAATAAGGCAAGGTTTTAGGTCTTCTTTTCCCTTTGTCTCTCCTCCAT

ATACCTTTAAACTTTCAATGTTACAAGTTCTTCAAGTGAATAAAAAGAGCTTCTTAATCCATACAACAACGGCTATAACT

ACGCCTCCGTTCCAAACATAATGGGATTGGCTATATAAGTCCTCACTGATCACGAGGATATCTTGTTGTTAGGCGCAATG

GCGGTGATTCCCGGACAATACTAATAAAATTGTGTCAATTGTATTGCCAATTTCTTCTCCATG

**>CaCIPK7**

GTGTTTCACGGTCTCCTATTTAAAGTTATATTTTTGATAAGTTGAATTGTGTCATGTTCTTTCTAATTATTTCTTCGATT

TATTTTTATCTTTTTTGAAATTATTTATAATCAGCCTTTCACACATTCACACTGAGGCATCTGGTCATCTCCTCTTCACA

TATTTAAACCTTCTCAGTTAATTTTTAATTTAATTATGAGAATATTTTATTCTAAAAAATTCGAATTTGAAACCTCTAAT

TACGATATTTTGTGGTAAACAATAAACACAAATATAATTATTTATTTGGTACATAGACTAAATTATTTTGAGATTATAAT

TTCTAAACTATTTTATATCCAAAGATGGTGTAAAATAATCTCATAATAGAATAAAATAAGTTATACCAAGACTAAATTTG

GTATCTTTTCTCTTACCTATTGACTTTTCTAAAAAGAAAAAAAAAATTCACATGTATATCTTGCATATATATATTTGTTA

CTTTTAGCAACTTGTTTGGATGGTTATCGTATAGTATAACATTGTTAATTTTAATTATAGTGTTTATTTTGATTGTTTAT

CTAAAGATTGTTTGTATCATAAGTTAAATTGATTGTTAGATAATTGCTAATGACAAAAATTACCTGACAATCAATAGATC

ATATATGGTTTAACGGTTAAAATGATATATCTAAATTTATTGTTACCTGCTATTAATAACGTTGAAAAACATTATATTTA

TTAATATAATATTATTATTATGATCGGACAGAATAAGCAAATCACAAGCAAAAAGAAAAACAAAATGAACACACAGATTT

ACTTGAAAATTCTTGACGGGAAAAATCATGAGCAAAAACGGAGGTATTTCACTATAATTATTGGAGAATACAATGAGAAG

AATGACGTATTTTCTGAACGTTCAAAACGAGCCACTAAATGCACTTATATAACACATGAATACAAATAAATCATAAGCCC

CAAAACATACAAGTCCATACCGCGGAGCTTCGCCCCCGCACCCCCAACTGGATCAGTGGTGGGCTCCGGATACACTACCA

TTATAAATTGCAGCGTGAACTTTTCAGGGCCGAATTTACAAAATTCAAATCACAAACTCTAACAATTATACACTATAGCA

CAATAATAGATATTACAAAACAATATATAACAACCACTCAAACAAGATCGAGATTCACTTGGGCGAAAGAAAAGGAGGCA

GTCGACCTGATTTACAAACTATATTTGAAAAACACGTTTTTCAAATAAATTCTAAGTAATTTTCTATAGTCGTACTTGTA

CACTCCTAAGTCCTAATCCTAAATTATTAGTGAAAAATTAATAAAAATCTGTAAACCCCAATTTTAATAAGAGTCCAAAA

ATATTAAAAAATGACAGATTAGATACAGCCAACTAATCTTTCCTATATAACCCAACCTTTCACCCCTTTTTCTACTTGCC

CATTCCTTTACTTCTGCCACTTCTTCTCCTCCCTTCTCCGCCGCCTTTGCCGTCGCCGGCATG

**>CaCIPK8**

ACAGTGCTAATTAAAGTGCAAAATATCTTATAAAGTCATGCAAGAGGTGGTGCATGTCTATTTTTCAATACTTGATAAAT

ATCTAAAGGAATCAATTCGATGCAGTGCAGTGGATGTAGTTGCTCCACCCTTAATTAGAGGTCGAGGGTTCGGCCCTGGG

TATGAAGGAAACTCTATTGGGAGCGCTGTCATCTTAACGGCCTTGCAACGCGCGATCCGAATTAGTTGGGGCTTCAATAC

AGACATCGGACACCAAATGAAAAACCAAAAATAAAAATACTGGACTCAATGAATCTGGTAATATATGAGGTATCAATTTT

GTACCAACATGGGTTGTGGTGTAGTAATGGAACTGTTCCACCCTTAACCAGAGGTCTCAGGTTCGAGCCCTGGTATGGAG

AAAATCCTGTTGGAAGCGCTACCCCCAGAATGTGTTCTGCAACGCGTGCACCGAATTAGTCAGGCTCCACTGTGGGCACC

GGACACCAGGTGGAAAATCAAGAAAAAAAAAGTGATATCGATTTTGTGCATTGAGAATGAAAGTTTTATAGTTGCGAACA

CCTTTTTTCTTTTAGTACATTATTTCTTATATGTGGATTTTAAATTAGTCGAGTCAGTGAATATCGAACTAAAGATGAAT

ACTTTTCTTACCTGTCTTCAACTTCCTATTGCAAATTGTTTATACCAGAAATTTAAAGTATATGTAAAGTTGGTGATATA

CTTCTTTAAAGAAAAAAAGTGGAAGTGACAGATTTTAAAGAATCTATAAAAAAATATCTATACAGTATAAATTGATGATA

AATTAATTATTAATTTACTTTATGAAAGTGGAGAAGATTTTATATATAACAAATCTATAATAAGATATATATGAGACAAT

CCAAGTNTTAAAAAAAAAAAAAAAAGTAAAGCATCTAGAACATCTTGAACAATGATCAAATTCGCTACGACACACTCTAA

TTTCATGGGAGTTCTATTATCTTTCGAACTCAATTTTAGCGTATTTTTGTCACCCTATTGTGTTGACGTAACATCTTTTG

TCAACCTTTTTAGCTGACATGATATCCTTAATGTGGGCTTCATTTTATGTAATAAAAGTGTCACGTTAGTACAAAAGGGT

GATAAAATACGCTAAAATTGAATTCAGGGATAATAGTACCGCTGTGAAATTAGAGTACACTGGATGCTTATCTCTTTAAA

AAAAAAAAAAATTAAACTTATGATTTGTGACTAAATAAGTTGTTAAATTATATTCAACAAAAAAAAAGTTTCTTGATTTC

CAAGATATGTATATATACTACATACTTATTATGACGATTCGATATAACTTAATTAACAAGACAGATAATAATGAATATAA

CATAAGTTATATCGATTTTTTATTTTTTTTGCATGAAAAAAGTATTCCTCATGCAAAGTAAAACTGATCTACTATAAAAC

CAAGTTAAACACTTCTTGTTTGACAGCCCCCACCCCACCCCTCCGTCTGCCATCGCGAACATG

**>CaCIPK9**

CATGATGTTGGACTCAACTTTATCTCTAATCCTTAACGTACTTTTCCAGGCATGAGAGTCAGAAGAAGGAATGTCTTTAG

AGGCAGGATGAGATCTTTTACAACAATTGGCTCGAAAAAAAGAAGCTCAGAGAGAAGAACAAGTTCTGAACCTCCACCAC

CTCTTCAAGATGAAAGAGTTTGCAATATATTCCTTGACCGATGGTACTTGTCTTTGCCTTCTTTTGGTCCCGAAAGGAAA

TTTCAAAGTGTTTTTCAATAAGAGTTAGAGAGTTCCTTTCGGAGGATTCATAGCGCTGAGAGTATAAGTAGGCGTTGATT

GTAGTACGCTTTTGATCAAAACCATCTTCCCTCCATATGAGAGCATTTTACCCTGCCATGCATTCATGTTTTTAACGATC

TTTGTCATCATGCCGTCAAAGTAGAAGATTTTCTTCTCGCCGTGATAGATTGGGAGCTTTTAAAATGACAGCTCGGTGCA

CCAAAGCTCCCGCTACGCGTAGGATCTGAATAACGGTCCCACCACAAGGGTGTATTGTACGGATCCTTACCTTACATTTC

TGCCGGACGCTGTTTCCAAAGCCTAAACCTGTGACCTCCTGGACACATAGCTGCAACATAACCAGTTACCAAGGCTCCCC

TTCAAAGATTGGGATCTTTTATTCTATTTATTCTACTAGTGGAAGTATTAGAAGCTGCAATGAAAAAACTTTTTTCTTGG

TTCACTTCTGTCCAGACACTTTTTCATATCTTTTAACTTGTTTTAGAACAAGTTGATTGATTTGGAATTTCCACCTATAA

AGATAATAATGTCATCAGCATATGCTAAATGGTTAATGATAAAATTTGAAATATTCATACTGAATGGAGTAAACCATTTT

AGCTTTCTATAATGCAGGGAAAAAAATAATTAACATGATCTTTGCGCAAGGATGACATGCACCGGTCAAGAAATGGTCCC

TTGATGACACCTGTTTTAATAGCTATCGAAATTTCATGACATGGTTTAAGATTACAAGTTTCAAAAATTTAGTTTTTTCT

TCAATTTTATGCTCAATCAAACACTATATAATGTATATCACATAAACTAAAACAGATGGAGTAGCTTCTTATATGTAGTT

TTAAATTCTAACAAAATATATGAAAAAATTATTCGACTTTCTAAACGGAAATAGTGTCATATAAAATGAAGTGGATAGGA

TATGAGTACAAATATGTATAAATATAGGCCTTTTATATTTTTATTATCAGTTATATATACTAACCAACCAAAATCTTGAT

GTCATAAATACCACAAAATTTGTTCAAACACAAAAACACACCTCCTCTAACCCTGGCTAATTTCCCCCTAGCTTCCTTCA

ATCATCCTCATCACTCAAAATTCAAGAAATGTTCCCATGAATCCATGCTACAAAAGAAATTCACAAGGCAAATACGCGAT

GATGATTGGCCATAATTTCTATTTCTCCACGCGCTTTTGATGCATCATCTGTCCTCCACGATG

**>CaCIPK10**

CTTAAACTTTGGGCTTAAACACATATCAATTCATATCAAAACTTCATACTAAATGTGATTCTTGAAATACTTCACAATAA

CAAATCAAGAAAGTGCAATCTCATTCATAATCTAAATCTCAAAAGATAAAACATAAACATAAGCACTTCTTGATCAACTT

AAAAACTTTAAATCTCATAATAATTACATGCTTCAAGAATATGTAAACTTGGGTATAAACCCTACTTCAATACAAGTAAT

TAGAATCATAAAATCATGCAATTTGGGCACAAGGGTGAAAGGATACCCTTGCTCATAACCATACATACCTGATTAGGCCT

AATTGATGTAGAACCTTGACTTTGAACTCCTAAAGATGTTCTTGAAGCCTTCCTCTTGAAGTTCTTGGACTTGGAAACTT

AGATTATTGACTTTCTTGAAGAAGGAGTAATGGAGTTTTGAAGTTCTTGGATAGAGGTTAGGGTCTGTTCTTGAGAGTGG

GAGGCTTTCTTCAAGAGAAATTATGAGTAAAGAGTTAAATATTGCTAGACGGATTCGGGTGAGGGAAAATGTCCAAAGTG

ACCCTAGACCCTTTAGGAACTGAAGCAGTGTGTGAAAAAACTCCACATCGCGTCGGATATCATGTTGGGACAGCACTGCG

TTGCGTCACATATCGCGCGAGAAACTGGTGCGCCGCGTCCCAATCGCGTTGGCTTACTGCTTCACACGAAAAATGCCATA

ACTTTTCACTCGGGTATCGAATTAAGGCGAAATTGGTATCGTTGGAAAACTAACTTAATTATCTACAATTTGGTTGGTTG

TGAGCTGAAAAATTATACGTATATAAAATGTTATACACATTCAATGTAGACCCTTGTAGAATCGAATACAAAGATTTGGC

CGAATCAAGGGCTCTTAGCTCAACTTCGCTCTAAGTGATTCCTATGAACATGTTTCACCTCGAAAACACTTCACATATTA

GGATAATGATAATGAAACTTATGTTCACATGGGAATCATTTGGATTGGAGCTTATACGCGTAGGAACGATGGTAAGAATT

CTAGCGCAAAAACACGGGGTGGTACAATTAGTGACACGGTTTGATACATAAATCAAATCCAACAAGATAAACTAATTCAT

ACCTTATAGATTTTACAACTCCGAAAATCACCTTCCTTAATTGTCTTCCAACCATGAAATTTTAACGCTGTCAGAAATCC

ACATTTACAAAGAAGAGTCATTAACACAGTATGAAGAAAAAGGCTATTATAACGCAAGAAATTTAGCAATTAAAGAAGAG

AAGAAGAACAAGAAAAAATTGGTTCAAACTTAAGGTGTGATTTAATAGGTAGGAAAGTTAAATATGAAGAACAAAGGATG

TGGACAAGAGAGAATTGCTCAGATGGTAAGCACACCTCACCTTCAGACATTAATATTGTGTATTTGAGTCACCAAAGGAA

CAAAAGGGTGGGAGCTCCTAGAGAAGGGTGAAAAAAAAGGTAATTGACAAAGAAAAATTCATG

**>CaCIPK11**

ACCATTTTTTCCCATCACGTGCATTAAAACAATTTCCTATTATGAAGGAACATGTAATAAAGAGGATAAATTATGTACTA

AGCCTAAATTGAAAAGAATAAAAAATTTATTTTTCACCAAATTTATATTAATTCTTCTTTTATACAAGGAAACTCAAAGC

AACGATAATATTTACGTGTACTTTCGATAATAAATCTCATGTTACTAGCTTGATCAAGAAATACATAAAGATAATTTTCT

TCTCAATCAATTCAACTATCACCAATATGACAAATTTTAACATTTAATAATTATAAATTAATGTATATTCTCATCTATAT

ACTCAAATGAATACACACATAGGCATCATTTTCAAGTGGTGCATTCTCCACTAATGAAATCTCTTCTTTTCAACAACTAC

CAAGTTGTGCAATTTTTCTCTATATACTTGTCATTCCTACTTTACCAAACCAAAAATCCCAAACCAAGTTGGGCATTTTC

AAGGGCTCGTTGGTACAAAAAAAATAAAAGAAAATTAATCTCAATATTAATTAATTTTTAGAATAATTTATCCCACGTTT

GTTTAAAAAAACTCAAAAAATAATTTATTCTGAAATTCAGAAGTAAATCTGTAGCTAAACTTTTAGTGCTCCAACATCAT

TAAAGTCGACGCAGATTAAATATAATTATATAAAAAATAAATAAAATTTGATATTATAATATTTAGAAAAGCACCCAGTA

AGTCTAGAAGATAGCCGTGAGTGCTTTAATTTTCACGTGAAACTTTAAATTTTGGGACGTCCATATATGTGTTCGAACCT

TCAATTCCGAAATCTTGGATCCACAATTAGTAAAATTATAATATTATTTTTATCCTAAATTAAAATTTGAATAATAATCC

TAAAATAACTAATCTAGAAATAAATTACTTCCTCCGTCTCAAATTATTTGTCTCAAATTTTCTAATTTGATTTCTCATTT

ACTTGTCTTTTTTATTAATTAAGAAGACATAATTTTTTTTTCATGTTTTACCCTTTGTATTAATTACTTTTTATTTAAAT

TAAAATGTAAGCGTCATTTAATAAGAGTAATATGATAAATTAGGTATGTTATTAATTATCTTTTTAATCAATATGCTATA

TCAGTTTGAGACGAGTAATTTAGGATGGATTGAGTATCTTGCAATAACATGTAACTCACCCAAACAAGCCCCAAAGTCCC

CACAAAACCACTTTCCAAGTTTTCAAAATCCCCAAATCACCTTTTTGTCATTCATTTTCAACTCCTTCACAGTTCATCTT

CCATTTTTTCTTCAATACCCTTTTGCTTTATTACTTAAAATAATAACAAAAGCAGCTAAAAAGATTAAAAAAAAAGCCAA

AATCTTGTTCATCAAGCCCAGTATGTTTTCTTGAAAAAGCAAAAAAAAAAATGGGTTGAGGAAATTAACAACTTCAAAAA

GAAGTAAAAGGGTGTTGTTAGATTCTTGATTTTTTTAGTTAATTTTGTTAAGAAGAAAAAATG

**>CaCIPK12**

AACGCCACGATTCAAATCATGGAAATAGCCTCTTGTATTTTTGAAATGCATGGTAAGGCTGGTTACAATACACCCTTGTG

GTTCAACTCTTTCCAGATTGTGCATCGGACTGTCTTTTGTTTTGTCTTGGGTTTATTTGCATGATGGATTAATTCAAGTG

ATATATCAACTTTGAGTCTTGATATATGAAGTTTTTCTCATATCATTTGTCTTGAATTTGCTTGTAGTGCCTTCCTTTGT

TTAGCCAATATTTGAAATTTCTGGGTTCTGAACTTGCCACCGAATCCATAGCTCGTTTTAGTTATTGGGTTCACGACAAA

TTATATATACATTGGATTTTTCTAATACAAATATAGCGTCTAAGCAAAAGCTATTGTGTTCCTGGAGTATGGAGGTTGGG

TGTTTCCAAAAACATTTCTTTGACTGAATTTGCTAGATTAGCCTTCCTTTTCTTCCTTGAATAGGGGTGTTTATCCGGGA

TTTGAAACTTATGGGTTCTGAACTTGTCACCGAATCCATTAACTTGGTTTACTTATTGGGTTCATACGTAATTGGTTATA

CACCGAACCCATTAGCTCGTTTTACTTATTAGGTTCATAAGTAATTGCACACACATCGGATTTTCTAATACAAATATAGG

GTCTAAGTAAAAGCTATTGTGTTCTTGTAGTCTACGGAGGTTGGGTGTTTCCAAGAACATTTCTTTGTCTATGTGCCCAT

GACCATTCTCAATTTGATATGAGAAAGTATCTTTCATGTCTCATCAGCTCATTTTTATGCATCAAATTTCTGCATCATAT

GTTGTTAAGACTCTTCAAAAATGTCATCGGGTGCGTGTCAAATCCTTCAATAGTAGTGCATTTTTGCGGTATCAAACATG

GGTGCAGCAACATTTTTGGAGAGCCCAAGCAACTTATTAGTCTGCATCTTCTTTCTTTTTGTCTAGTGAAAGACAAAAAA

AGGGTGATTAAATTTGTTTTCCTTTTTTCTTGCTTGATGTATACACATTAGTAATTCTGACCTTGATGAATTAAACTTGT

TTTGAATGTGATGGAACGTGATGCCCCTTTTGTCTGACTTTCATAGTCATCGATTTTTTTGTAAGGCAATAATTGTGAAT

CTTGGATTGAAATTTCCTGACTCTCTTTTTTCTATTGATGTTCAGTGCTTGGTGCATATTTATGAAAGCAAAGTAATTGG

GAATGCAGAGGGATGGGGCTGTACAACAACGATTGCCTTTTGGAGGCTTCTAAATTTCCGTGTGCATTGAAGTTGATCTG

TTTCATTCGTAGTGATCTCGAATATGCAGTTCACAGCACACTTGACTTCTCGTCCCAGATGAACAATGAAGCCACAACCC

TTAAACATCCCGCAATGCTGAAGCACAGTCTTGTTGGAGCCGGTTGAGCGCTGCAACAAGGCCCCTCCTGCCTGTTAAAA

ACGAGTATCATATCAGCAGGAGAAAGTAATTAATAGTGGTTACTAGGATTGTTGGTTCAAATG

**>CaCIPK13**

GTCTTAAATTATTTATCATGAATTTTATAAAATTATTGTCTCAAATTATTCAACTTAATTTCTAAAAAATAATTGAAATA

TTGTTTCAATATTGATAATCCCTTCTCTTAAATTGTCTATCGTGTTTTAAAAATAAATATTTTAAGTTATTTATTTTGTA

GAAATTTAAGACAAATTAATTATTTTTGTATATTCTTATTAGTAACTATTCCTGAATAGTATAGGCATCTAAATAGAGTA

AATATTTAACAAAAAAACAAATATACCATAAGGTATAATGAGGATAAAATAATAAAAACCTCCCACAATTAATATTTTCT

CAGAAAACCGTTAAAAAAGGCATGGAAGATCATTTGAGGCTGAAGTTTTTTAGCTGATTAAAGAAAGATGTAATAAATTG

AAAATTTATAATTTAACAACTTGAATCCAATAAATTGAAAAAAATAACTACTCAATGATAAAAAAAATAAAATAAAATAA

ATATCTTTTAATTTACTAAAATATATGATTTTTTTAATTATACTTCTGTTAAGAAACCCAACAACCCCTATTAAAATTCT

TATACACATTTGTCTTACTTATTAGTTAATCAATGAAATTTATTTATTTCTCAATAAAGGAGAAAACCCGGATTAATTAG

TGGATAAACAGGTTGGACCACACAAAGAAAGGTGGGCCCCATTTTTTATTTTTATTTTTCTTAAAAAATACTAACATTTC

TTCCATTTTAATTTATTTGTCGTGATTATTATACATATACTAATCTCCATTTCGTATTATTTCATTGTTATTACTGCTCT

CATTTTTGTATTTTCTTTTTCGAATTACTAGACATGCATAGTTAGATAAAGATTTTATAAAAAATAATCTCTCTATTTAT

TTGAGTTAACAACTATCATATCTTTTTAGATTCCACTAGATAGATNNNNNNNNNNNNNNNNNNNNNNNNNNNNNNNNNNN

NNNNNNNNNNNNNNNNNNNNNNNNNNNNNNNNNNNNNNNNNNNNNNNNNNNNNNNNNNNNNNNNNNNNNNNNNNNNNNNN

NNNNNNNNNNNNNNNNNNNNNNNNNNNNNNNNNNNNNNNNNNNNNNNNNNNNNNNNNNNNNNNNNNNNNNNNNNNNNNNN

NNNNNNNNNNNNNNNNNNNNNNNNNNNNNNNNNNNNNNNNNNNNNNNNNNNNNNNNNNNNNNNNNNNNNNNNNNNNNNNN

NNNNNNNNNNNAAAGCTTTATTAAGCGATGTCAAATAAATACTTGTCATTTAATATAAAACAAATACATTATACCATAGA

AATTAATATATTTTCCTTATATATATTACACTCCACCAAACAGAGCAGAGAAGAGGAAACACAACACCTGCTGATCTTCT

TCTCCATTCACATACTACACCCAAAAAAGAAAAAAAGAAAAAAAACTGAAAAAAAATTTCAACAATTTTTTTTTTCTGTT

GAATTTTATTCAATTTTGTTATAAACTTGGAGTTTTATAATTATTATCATTTTTGTGAGTATG

**>CaCIPK14**

TGCGGTCTGAGGAAGGGTAGAGTACTCTGACCTTAAATGTTGTTTTCAATAGACCCCCAGCTCAATGAAAGCAAAAAACT

ATTTATGAATATATAAGCAATAGCAGGATGGTAAAAAAGTAGAGGTCCTTCCCCTACCCCCAATTTTTAAAAGAAAATGA

TTATTTTTTCTTTGTTGCTGTTGATATCTATTGTAGTTTAAGTCAATGCCCCATTGCAGGATTTCTTTTTTGTTTTTGCA

TGGTGGGATATTGCTGAAGGAACTTTACAGCTATTTTTTCAGACAAAAAAAGGGCTTTACAGCTATTGTTGATGGAAAGA

TAATTTATTTTAGCGTCAATTTGACTTTTATATTAATTTCAATCAGATTTGAGTGTACTAGATGTGGCTCTTAGTTTGAA

TAACAGCTAAAATGTCTAATATAACAAACACTAACTATATCTTTGATGGTCTACAATCAGAATATGTTCTTCTTGATGTA

TCTACCATCTTAGCCAAATTTGAAGTAGGAACTATCACTTGTTTTATGTTTGTCCTTTTCCATACTGCTCGAATATGCGA

TACTTAAGCCGGTCTTTCAGAAACATTCTCTCTAATTCTACGAGGTAGGGTAAGGTATACGTACACTCCACTTTCCCCAG

ACCCCACTGATGTAGGATTTCGCTGAATCTGTTGTTGTTGTTGTTGAACAATCACTTGTTTCCTTGGAATTAGTAAGTTC

AAGTAAGCGTCATTATGATTCTCGATTCTTCATTGGAATTTGTGCATTTATTAAGTGTCATTATGATTCTCGAGTCTTCT

TCGAAATTTGTGTGTATCTAGGTGCACAAAGCATCTCATTGTTGCAGATTGGAGCGGGATTATATTGGGGTTGTTGTATG

CAATCTTACTTTACATTTTGCGAGAGGTTGCAAAATGTATGGTATGGCACCTTGCTATATAGGTCTCTTGCAGTAGAATT

TCTCAGTTAATAATTGTTTGAATACAAACACATATACTATATTCGTGGTAAATACACTGCTAATATATGATCAATCAATA

ACTACTTCCAATTTTGCTCAAGATGCATTTCTTTACCTTTCAAATTTTATTTCTATTGTTGGAAGCATAATCTGTCTAGC

TACTGAAATCTTTTCCTCTGATTTCACCTTTTCCAGGGGGTCAGTGGAAGTTCATTTAGTTGTTTGACTACACACTTTTT

CGGGGAGTCCTAGGAATGCTGAAAGATGTGTTTGTGTGTGTGGAGTTCTAGAAGTGATCGTCTTGAGGCTTTGGGCTTAA

TAGAGTTGCCAGAGATTGAGCCCAGGTGCTGCGACCATTGCATCATTGTCTGCGCCAAGATGCAAATTGCGCGGAGTAGC

CGTACTTTTGCATTAATAAATGATAATTTCTGCTCGGATGGTTGAGCGGTGTTAATTTATAAGATTTTGCATCTAAGTTC

TGCTAGAAGGAGAGATATAGCTTTGCTTAGCTAGTAGTACTAGGTTTGGTAGATTTTTTTATG

**>CaCIPK15**

AATGATGCTTTTTATTTGAACATATCCCTTAAAAAGATCATTTATTTTCATAAAGTAAGATATATTTTTACTAACTTATC

TTTAATAAATATCTTGAAAAAAATAATATAAATAATATANNNAGCTCTTTAATAATTTTTTGATAATAAAAAAATTTCTT

CATTTAATATAAGCAAAATCAAAAGATATACTTTTATTAACCTACCCTTGATAAATATCTTGAAATTTTTTTAGCTCTTT

AATAAATATCCTGAAAAGGATTTAGCTCTTTAATAATTTCTTGATTACAAATTTTTTCTTTATTTAATATAGGCAAAGTT

AAAGCAATCATTAATTTCTTCTTATTTATGTGAAACATTATTTTTTTAAATATAAAATAACCACTATTTCAAAAGGAAGG

AGGTGTGAATTTTAACACTATAATTAATTATTTGAAAATCCACTTTACTAGTTGGATCCACAATTACTTATCCCTTCAAA

ATAAATAAATAAATTTAAAAATGATTTTTGACACAAAAAAGTAAAAAAATAAACATAAACATACCAAGTGGTAGGAACTA

GACACGCTAAAAAACAATAAAATCCAACTGTCCAATATGTCCCCATTTGTTGACTTACAAAATTGAAACACACAACTTTG

TTGTTTTTTGATAAAAAAAAAAAACTAAATATACATCTTTTAACTTATTATATTTACACAAATTCTTATCCTTATAAAAT

AATTATAAAAATTTCAATTAATTATGTATTTATGTAAAATAATTACAAAGATCTAAATTAATTATGTATATTTCTTAAAT

ACTCTGGAGAGCTAATTATTTATATTACAAGGAAAAAATATATCTTCATTAGATATATTATGTATCTCGATAGATTCAAA

AAATCGAAAAAATACATCATGGTGCTAATTTTATATCTTTATAAAAAAATGTATTTTTATTAGATGTATCAGGATCTAAT

TATATATTCCGACAAATTCAACATCAAAATTTTCAATAATTATGTAGAAATATCATAATTTTCTCTAATTAATTTCAAGC

AGTTGAAATTTATATTATTTGTCATATAATAAATTAGAAGAAACGTTTTTTTAAAAATAAAATAAAATGTGGTGTCTCGA

CATAGATTAGTGGACTCTTATCTAAAAGCTAACACGTAAATTTGGACGGATGGAGTTGTAGAAATGATGATATAATGACG

TGTAGGGATAGACATTTCACACCCACCAATTAAAATATTACTACTTTTTTTTAAGTATGTTAATGTGGAAAAAAAATAAA

AAATAAAAAAATTGCAGTATATATATATATTGTCTAGAAAGGCGAAAAAGTAATAGCCATTGGGGGGGATTCATTTTCTA

TAAGCCCCTTTTCAACAACTCCAAAAAATTTTCTATCATTCTACCAATTTTTCTTCTTTCTCATCAGCTAGTTCCATCTT

GATTATCAAGATAGAACGTTTTTCATTGGCCTCTAGTCGGCGCATACACGTCGACTATTATG

**>CaCIPK16**

CTATATCTGTCAAAATTCAAACCCAACTTATTGATGAAAAATAATGGATTATGCTTCTCTAGCTTATTAAGAATGTACAA

TAATATGTATATAATAAAAAATATTTATATTTAATTTATATACTTAAAAAAAAATTTCAACGAAAAATATTCATTTGACC

ACCCTTCATTAACTGTGGCTCCGCCACTTATCTCAACTGCACCAACAATAAGGAGCCTAGAAACTTTGACACCCTAGTAT

TTAATTAGACCGACTTTATAGATTACGTGTTGGACTTATACCGTCCTTATTTATAAAAAATAATAGTATTAAGTCACGTC

TCACGTGTACTGTCTTTCATTGAATAAACTAATCCTGTTACTTTCTATTTGATTTTGTTGTAACGTTGCTGTAATTTTTA

GACAAAATTGTATGGAGTATATAGCTGAATAATAATCAATTATACAAATTATAAATATATATATTCATACTACTAGCTAG

TAAGTCTGTACAAATAGTGGCGGAGCATTACTTATTAAGAGTGTCGATTGAAAACTTTTGTTTTCTAAAGTTATATTATA

TAGAGAGGTAATTCTTTTATATGTATATTATTACTTTATGTTTTGATTGCCTTTAGTAAATATTGAAGTGAATGGTCGCA

AATTAGAACTACATATTAGTGGACCAAGATTGTGAGTTAAAACGATAAGGAAACACAGATAAGTGAGGGGAAAACTTTTT

TGTTTGGATAATATAATTAAGCAATAATCGGTTTAAGCAAAGAGAAAAATAATAATATGTACTTCATTGAAATTTGTTAG

ATGACTGAAACGTGTACAGCACTCTGCCAAAACCAACTTTTAATTGTATTGACTGAAACCATTGAATTAAATCATACCAT

TTTACTCGTTCCAAATTTTTTAATATTTTNNNNNNNNNNNNNNNNNNNNNNNNNNNNNNNNNNNNNNNNNNNNNNNNNNN

NNNNNNNNNNNNNNNNNNNNNNNNNNNNNNNNNNNNNNNNNNNNNNNNNNNNNNNNNNNNNNNNNNNNNNNNNNNNNNNT

AAAATGTAAACATCATTTAATAGGAGTACTATGATAAATTAGATATATTATTAATTATTTTTTTAATTAATTAATATATA

ATATCAAATTGAAACGAATAAAATGAAACAAGTGGATTAAATTAAAACAAGTGCCTAATTCAAAAATTCAAAAATTTACA

ATTCACGTGGAAACAAAAAATTCTTCGACTGGACCAAATAAAAAAAGGAAAGCATTTGAAAACAAAGACGTCCTATATAA

CACAAGTACGCTGTTAAGGACAACGAAAACAAAATAAACTTAAACAAGAAGTTTCCCTATTTTGAACTATTCCTATATAT

ACAACAACAAAATTAGAAAAAACCAAAAAAAAAAAAAAAGAAAACTATATATACCAACCCTTTAGCTTCTTCTTCATTAA

TCATAATTCGTATCTTATAAACCAAAACACAACATTAATTGCCCCCGGCATCTTTCACCGATG

**>CaCIPK17**

ATGAATGACAAACAAGATATAACAGTGAAGCAAGTAATTGACTTGATCGCGATTCAACATGTTGTGTGCGGGTTCATGAA

AATCTAATAATTTTTGCTTAAACTCTCTATATACATTAAAATACTACCCAAATGTTTGGCTCTAATTTAATTACTTAGTA

ATTTATATTAACCTTAGTCATTATAAATGTATAACCTTCAATTTCTGAAATCACTTTGACTTGAACCAGGTAATGCATGA

TTCAAATTCCCATATAAGCTATCGCTTTTCTTAATATTTTTTATTTAACATTTTTAATATTCTTTAATACACAATTATAA

GAAAACAAAAAGAAAAGTATGCAATCACCAAAACAAGGGTGAAATACTAAGAAAGCTTATCATTAAATAACAATAATTGA

ACGCTTTAGTTTGAATTATTCTCCTTAATTTGTCGGGAGATATGATTGCCAAAATTTTCACAATAATACTCCATAATGGA

GTATGTATGTAATCACAGACAGCTCTTTCTAATCAATTAGAGTTGTTACTAATAACATTTCTAGAGATAAGATTAAAAAT

TCTTTTGAATTATCGCAAATTTTTTTATTTTTTTTTTTAATATTACTTATACGCCTAAAATCATGCGTAGGTGTAGTAAA

AGCGAGTTCGACCAGCCTAATCCGCCCAATTCCTTTAAGTTTAGACGTGTTGCTGATCTGCCCATTCATTAGCTCAACCT

ATATTCTAGTTCATTAAAAATTAGGTTGATAAGTAGCCCAAATCGATTTATAAGAAATCTTGTCAAAATATTTAAAAAGA

TATTTTTTAAATTAGATATGTTATATATATTTAACAAAGAAGAAAATAATTTTATAAAGTATTAAAAATTTATAAAAAAA

TTTAAAAAAAAAAAAGCAATTAAAACTCGTAAGAATAGGGATTAAGTTATAACCCACATTTTAGTTATTTGAACACAAAT

AGCTTTTGAGCGAGTCAATAACTCATTCATTTATAACTCAAATCTATTTTAATTCATATAAACTCAGCTCAGCTGCCCAT

CTAACACCTCTAATAATGCGGGATCTAAGGATATTAAGAATAAAACATATTCCTAATACGATCTCATTTGTTAGAAATAT

ATTTCATTCGTAAGATTAGATTTTTTATATTTAAATTTAGTCGAATATCAATACAAATATTGAACCCTGCTTGATATTTG

CAAAATATGATGGGCTTTGCCGCTCCTTGCACTACTGCCCAAACAATGTTCTCCAATAACTAGCAAACAGCCCACTGCTT

TCTCACTTGCCCCACACTGCCCCCACTCCGTCCGCCTCCTCTTATGTGTGTCTCTTCTTCCTCCTTCACATTTTTGCAAT

CACACAAATCTCCATCATCTCTCAATCAACTACAAGAATCTCAAATATATATATATATATATATATGTTCCATTTTAAAC

TTTTAGCCTAGTTCAAAATATTAAGATTCAGTCCTTATTAAACTTCAGCAGAAACCATTTATG

**>CaCIPK18**

ATATATATTGTACGCAGTTTTACATTGTATTTCTGTCATAGACTGTTTTCAAGATTCGAATTCGTAATCTTTTGATCACA

TGACACCAACTTTACCGATTATCAATTTCATGAAATATTCTTATTTATTTCATTTAATGTACCCATAATAATCAAATCAT

AGTTATTTTAGTAACTCAATAGTCTATATTTAGCTAATTAACCAAAACTACTTGAAATTACTAATTAAGATTATACCAAA

TGTGGCAAATTCTCACCGCAAGGGGCAAAATAGTCATAAAAATTACCAAACTTCAAAGTGACCAAATTCTTCACGTCAAT

TATTAATCAAACTGACCAGAATCCTAGCTGTCCTTACACGTGTCACTCTGTGATTGCCTAAAATATCCTCAAGATTCCTT

TTTAATCCCTCGTGATTTTGACACGTGTCATATCAACAAAACGATGGTCGTTGGATCATGACAACTCAATGTCTAAGTAA

ATTGACTATTTTGCCCTTGAGCCCACTATAAAATGACTATTTTGCCCTTGAACCCAGTATAAATTAATGCAACAACAACA

TATCCAGTGAAATCTCATAAGCAAATTTATGGAGGGTATAAGTGTACGCATACCTTTCCACTATCTCGTAAAAGGTAGAG

AGACTGTTTCCGAAATATCCTGACTGAAGTGAGTAAATTTATGATAATTGCCTAAAATATCCTCAAGATTCCTTTTTAAT

CCCTCATGATCTAGACACGTGTCATATCAACAAAACCATGGTCGTTGGATCATAACAACTCTATGTCTAGGTAAAATAAC

TATTTTGCCCTTGAACCAACTATCAAATTTGTGCTGCAAAAACGTATTCAGTGAAATCTCCACAAGTTTTCGCTACCTCG

TGAAAGATAGAAAGACTGTTTTCGAAAGACCTTTAGCGTGAATAAATTTACGGTAATTGCCTAAAATATCCTCAAGATTC

CTTTTTAATCCCTCATGATCTAGACACGTGTCATATCAACAAAACCATGGTCGTTGGATCATAAAAACTCATGTCTAGGT

AAAATGACCATTTTACCCTTCAACCCACTATAAATTTATGCTACTTGCCTTCATTCAGAAAGATTAGTGAAACAACAAAA

AAGCCCATTCATTCTCTTGTTGTTCTCTTTTATTTTTTTCTTCACCAAACCCTAAATTAATTCGAGAATAAAATCTCCAA

TTTTATTTTTTTTCTGAGAGGAAATTTATCTAGGGAATGCAGAGAGATGAGAGGCTCTCGGTTTTCTAATCCCCTTTTTG

GATTCGGAAAAATCCAAAAATCTGTTACCGGCGGCAGAAACTGCCATCGGAAACACGTTGAAGACGGGTGAACCAATCAC

CCGTTTTCGCCTTTTTCGTCCTCCACTTTTTNNNNNNNNNNNNNNNNNNNNNNNNNNNNNNNNNNNNNNNNNNNNNNNNN

NNNNNTTTCAACTCAAAAAGCAAAAAAACGAAAAAAACCTTCTAGTCAGTCCCGAAAAGAATG

**>CaCIPK19**

ACTTTTGATTTAGTAAAGTTGGAAGAATGAAGAAATTAAACTAACCTCAAAAATAAATTGAAGAAGATGATGATCTTCAA

CAATTTGATAAGATAAGGTTTGTAAATTCAAACCTTATAATTCCTTAATTATGGTTATTTGATTTTAGGCATAAATTGTG

GAGGTGTGATATGTATATGGAGGTTGGAGAAAGAAAGTGTAAAAAATAAGATATTTGTAATTAGTTTTGGTAGATGAAAT

TTTATATAATAATAGAAAGGTAGGATGTATATTTATGTAATTTTTTCTAATTTAATTGCCTATTTCTCAGAGCATGTCTT

ATACTGCTATGGATATCATCCAATTCAATCAATCACTCAAATTCAATTAAATCTAGTACTTACCCATTTTTCAAACATGA

CTAAAAAAAAAGAGTGAGAAAAGTCTCATGCAACAATCGACTTTTGAATAAACGAATAACTAAAATTATATTATATATTT

CTTTCCCAACAGATCATACAGGACAGATACGCGAAACTCAAATCATTGCTGGATAAAACAAAAGGAGATTAAACAATAAA

AAAATAGTTGGCAATGGAAGAAATAATGAGGTTCATCCACCAAATCTGAACCAAATATTGTGAAGAAATAAATTCTTAAA

AAGAAGGCCATTTTATTCATTCTCATGCCTGTTAAACTCCTTTCTTAATCACTGCAAAGGAACTAACAATACGAATAAGA

TTACTTCATTCTTAACCACATCTCTCAAATTCTAAAGTGATTCTCCTTGAATGAACCTCACATAACACGAATTTGGAATA

ATCAATCTCGTTGATACCGAATACCAAGATGATTATTAAACAAAAAATCTTATTTCATCACATGAATTCGTGGCCCTCCA

CTACAGCACCTCGCATCATTTCCTATTTAATGTTGGTTTTATCTCAAAAACTAGAATAAGTAATAATAATTGCTACTATC

CTCTAATTTTAAATACAATTTACTTAATTATCACTATTGTCGTTATTTCGGTGAAGGTAGCATCAAACAACTTGAGACTT

TGAAAATTCAACTTGAAGCCACTTTGAGGCACTCAAATTAGGATAGAAAATGTCATGCTAAGGACAGCAAGCTGTCTTTT

GCCTTACTCAAATTTCTACTACTTATTGAATTATTCAAGAAACTTCTTTTTATGTGATATTAAAAGGGCAATAGACATTT

TTGAAATTGTAACATCCTCTATTATATATTAGCTGCTTCTTGAAAATCTTCGTTCCCCTCTACTTCTTAATTTGCTTATA

GTCTCTCTCAACATTCCACTTGAAAACAACTTTTTAAATGTGCCCATTTTCTTGATTACTAGCATCTTTGAAATCCCTCC

CTCGCTCCCCCCCCCCACCCAAAGGAAAGAAAAAAGAAATAAGGATTTCCCCTTTTGTTTTTACAAAAAAAAATAAACCC

CCCCCCCCCCCCCCCCCCCAAAAAAAAAAAGTATCTTTTTGAAAGATCTAATATAGTAATATG

**>CaCIPK20**

TTTGATAAGTAGTTTAGGGAGGAAAAATGAATAATTGATAGTTGAAAGCGCATTTTGATAAATAGTTTAGCTAGGAAAAG

TGAACAGTTGATAGTTGAAAGTGACAACAACAACAACGTACCCAATATATTCCCATCAAGTGGGGTCTGGGGAAGGTAAG

TGTACGCAGTCCATACCACTATCTCATATGTGAGATAGAGAGGCTGTTTTCGATATATCCCAACTCAAAATAAAACAATC

TAGACTGATAGTTGAAAGCGTATTTTGATAAATAGTTTAGGTAGGAAAAGTGAACAATTGGTAGTTGAAAGCGTATTTTG

ATAAATAGTTTAGGTAGGAAAAGTGAATAATTGATAGTTGAAAGCGTATTTTGATAAATAGTATAGGTAGGAAAAGTGAA

CAATTGATAGATGAAAGTGTATTTTGATAAATAGTTTAGGCAGGAAAAGTGAACAATTGTTAGTTGAGAGCGTATTTTGA

TAAATAGTTTAGGTAGGAAAACTGAATGATTCATAGTTGAGAGCGTATTTTGATAAATAGTTTATGTAGGAAAAGTGAAC

AATTGATAGTTGAAAGCGTATTTTGATAAATAGTTTAGGTAGAAAACTCACACATTTGATAGTTGTGGTATGAAATTAAA

AAAAAAAATAAAATCATATTTTGATCATTAACTCTTTCTTAAGTCATATGAGTATGTAAAGGTCATAAGATTTTTTATAG

GCTTAATATATAATCAACAACTTAAACTTGTTAGGATATTCCATTTAGACGCTCGAACTAAGCCTTGTTTCAATTGAATG

CCTCGACTCCTAATAAAGTATTCCAATTAGACACTTTCCTTTCGAATTGTGATAATCTTTTCTCTTATGTTCTTACCGTT

TATTTAGTAGTTAAGTTGACCATGTAAAATATGCGATTTTCTTTAATTATATACGCATTTGCAAGCCGTAAAACCCCAAG

CATTTTCTACTTGAAGTATAATTAAAGGAGATGACATATTTTGTTTTAATATACGGATGAAAACACGTGCAGAAGTTTTT

CAAAAATTTGAATCGCAAGTGTCTAATTGGAATAATTTATTAGGAGTTTGAGCGTTCAATTGGAACAATACTTAGTTCGA

TTTGTCCAAATGAAATATGTTGACAAGTTTAAGAGACTGACTATATATTAAACCATTTTTTTACCTATGAAAATTGCATA

TTTGTTATATGTGTGCATTTAAACATGTTATACATGGTCAATGTGCTTTGATCTAGATTATTGATTAGTTATATTATGAT

TCAATTGGACAGGTTTTTGATGAGCTATATTAAGCCTTTTTTTTTACCTATGAAAATTGCATATTTATTATATTTGTGCA

TTTAAACATGTTATACATGGTCAATGTGCCTTGATCTAGATTATTGATTAGTTCTATTATGATTCAATTGACAATTTTTT

GATGAGCTATAGTTAAAGGTTGCTAACTTTTGTGAATCCGTGGGAAATGAGTATTTAGAGATG

**>CaCIPK21**

GGAAAAATAGAGCGTACGTAAATCTAGANTTTTCAATAAAAACATATTCGGTGTAATTTCACAAACGTATCTGGAAAAAT

AGAGCGTACGTAAATCTAGAGGCGGAGCCAAGATTTCAAGGAAGGGAGTTCGATATTCAAAGAAAACTAAGTCGAAGGGG

GTTCAACACCAACTATAATCACATAAAAAATAATTTTAATCATGTATAAATAATATATTTTTCCGTCGAAGGGGTTCGTT

CGAACCCCCAAAAGAGGGCTGACTCCGCCTCTACGTAAATCATATCTCTACCTTAGAGGTGATAACGAGAAAATAATTTA

TTGTCGTTCACAAATACAAGCAAGTACAAACGTATCTGAAAAAATTAAGAGTGTACATCGTATTTTTCTATATGAAAATC

ACAGGGGCAATATAGTCATTTAAAATACCAATAATACACCGAACTAACCTATTCCATGCTACTTATGCCACGTGTCCGAT

TTTTATTGCTAAATATATTAACCAGATTTACATGCAACCCGTAAAAAAAGGACACGTGTCCATCATCAAAACCCTCATGC

AATCTCTCAAAATGACAATAATACCCTTCAGCCGACTATAAATCCACCTTCATTGCCACCATTTTGTAGAAATTTGCAGA

AAATTAAGCAAAAAAAGCTCCGAAATTCGAAAAAAAAATTGAGATTTTTGTTAAGATTTTTGGGAATGCAGAGAGATGAG

AAGTAATTCGTTTGTTTATAATTTTGTTATTGGTGGCTTAAAGCCCCAGATTTCAGTTGCCGGAGTTTGCCGGAATTGTC

ATCGGAATTGTCACCGGAAGCACGTTGAAGACGGGTGAACGTATACAACGCTTTCAACGAGGCACATCATCGATTTTTTT

CAGCGCACCCATCAATTTTTTTGCGTAAAAGGTACACTTTTTTCGAATTTCCTTCGCCGGTGGCCGGAATTTCGCCGGGA

TTGTCACCGTAGACGGTGAACCCATCGACCTATTTGCTTTTTCTGAACAAAAAGGACATACCCTTTTCGAATTTTATTCA

CCGGTGACCGGGATTGTTACTTTAAACTTGCCGGAAACCTGAATTCTACTGGAATCTCGCCGTATACTACGCCGGAAACG

GTGAACCGATCCACCCACCCCAATTTTTCAGCTCAAATACAAAGCCATTCGGTTTTTTTAGCAAAAAAAACAAAGACCCA

ATTCGAAATTTCTTTGCCGGCTCCGGAATTGTTACCTTAAACTCGCCAGAGATGGTGAACCCACCCACCTACCCCAATTT

TTTCAGCACCGAGATGAACCCTTCTGTTTTCTCTTAGGAAAAAACAACACCCTTTTCAAATTTCACTCGCCGGAATTGTC

ACCGGAATCTCATCACCCACCCCCATCAATCTTTTTTTCCATATTCGAACACCCTTTTCAATCTTGCTTCAAGAATCTTG

CTCAAAAAAGATCTGTTTTTTTTTCCTGTGGTGTCTGAGCCAGCTTGCGATAATTTTCTAATG

**>CaCIPK22**

TCATCCTCATTATTGATTACAACCGTTGCTTGATTTGCTCTAGTCGTCACTAATTATTTCCTGCTTAATAATTTACATTT

TTATTTATAATATCAAAATCATCTTGATACTTTCAAACACTTTATACCCAATTTTCTTAAACTAAAGATTGAAATAATTT

CTAATTTCTTGATCCTCGTGGGAACGATATCAGACTATCTAAGTCATTATATTACTTGCACGATCGCGTACACTTGCGTG

TGCGTAGAGACAAAATAATTTGATATATAGTAGACCCTCTAACTCGTCATATTCTAGAAGTAATAGTCGATGAAAGTTGA

CCTAAATAAGAGCATCTGCTAAAACTCTATTGATAACAAGTGCTCTACTCATCTTTTAGTGAAGAGACCTCTATGATCTC

AATTCACGTACAAATAGATTCCCACACTACTTAATTGATAAACATACTTATATTCACATAGGAGTCATTGGTTTAGGGAC

GGAATGACGATTTAAGCTCTTGCCTGAATAAGCGGGTTCTTACAATGACATAGAATAAAGTGGGTATTTGACAAATTTTT

CTCTAAATGGCAAAATAAGCTTTAAAAGTAGGTATTTTACTAACTTTACCTAGAAATTTATGGGTTTTCCACATCACACA

AAAATAATCAAACGGTAACAAAGGAAATTTGAACCAAGAATCAAGAGGACAATAAAATTTTTACAAGTCCCTTTACTTCA

CTATACCAAACAGCACACTTTGTTTATGACATCCCAACTTATAATTATATTATATATTTAATATATTTCTCTATATAAAT

ATCAAATCCGCAAGAAAGTTTTTGATTTTTCGAGAACTCCCATCCAACCCCTGGTCTGGCCGTGGTGTTTAGAAAATGAA

TTATTTCATTATACAAAGATAAATTATATTGGATAAAGTGAACGTGCATGTATTCACCTGCTACTGGTACATTATTGTGT

ATATTTTATCCATCGCTAGCTGGAGATCATCCATTAGTTATCAGTGCTTACTTGTTCTGTTTCATTCCCTAGATTATCTG

GTCTATAAATAATCCTAATATTTATTCTTAGGTACGAAACTTCCTCTGACCAAAAAAAAAAAAAGAGATGCGAAACTCCG

TAGAAAAATGGTAGGTAAACAAAGTCTCTTTGTACTATAATACCTGTAGAAAGGGTATAAGAATTCCTCCGTATATCTGA

ATCTTTTTCCTCTTCTCTCCTCTCCTCAAGTCTTTGCTTCAAGAAAAGCCAAATATTATCTTCTTTGTTTTGCAGTTCTA

TTAATCGAATACCCCCATTAGCATCTTTAGCAAGTTCCCCTCATTCCTAAGTGATTTTTTTTTTATTTCTGGTTTTGATT

GACGAAAAGGAGTACCGGAAAGATTGAAGTGTTAACAATTCAGATATCAAGCAAATTAGGTTTGCTCGGTGTTTTAATGC

AAAGGGTAGAGAACATTCACAAAGCAGGGGTTACTGTTTTACTGGTCTGTGGGTATAGTTATG

**>CaCIPK23**

CATCGGTCAAGATTAATCGATGCCTTTGGATCGAACTAGATAGTATGTCTATTTCTTTGAATGATGTCGGTGAACAAGTA

AAAGGAGGAGCAGGAAGCTCGAAAATAGCAAATGGGAATGGTTACCTATTTATTTGTTGATGTTGTAGAAACGCTTCAAT

CACAAAGGTATACTACGAATTTAGCGGCTTTGTTTGGTATATATAATATAGGGGGGCCTTTCATTTTTAACTTTTTATTG

TTTAAATAAGTAAATGATTAATTATATCCACACAACTCCCACTTATCTCTTTTTCACTCCTTTGCTTACTAGTGGATGAG

AATTTTGTTATATTTTTTTACTATTTCTTTAAGTCAAACAGATGGAAAGTTTTCATTTTCAAGTTATTTATACATGCGCG

TCATTGTTTCATCCATTTTTAACTTTGGACAAAATCACTTATACCCCCAATTTTGTTTAAAAAAGAGAAAATCGTCAAAA

ACCTCCAATCTTTAGCCCAAATTTTAACTATATATTTATATTTTGCGAGGGTCCTATGACCCCTTGAGCTATTTTAAAGT

GAAATTATTACCTCCAAAAAATCTGATAACCAGTCATTTTGGATAAGGTGTGATGCGTGCAAACAGTATTTTGACACGTG

TTATTTTTGTATAAAATTAATTTTTATTTACTTTCTTTTCCATTTTTATCACTTTTTATCATTTTTTTTCATTTTTTTAC

CATTATTTTTTTTCACCATTACTTTTTATCTCTTTTCCTTTTCTAACTTTACTATTCCTACTTCTATGCAAATAATTTTT

CGACGACCTTGAAGTGGAAAATAATGAACAAGACCGAATTTGAAATTTTCAACGATCTTGAAGTCGAAAATAATAAACAA

CATCAAATTTAAAAATTTTCGACAACCTTGAAGTGGAAAACAATGACCATATCAATTATTAAATTTTCGATGATCTTAAA

TTCGAAAACAACGAACAATACCAATTTTAAAATTTTCAACGACCTTGAAGCCGGAAACAATGAACAATATAGAATTTAAA

ATTTTTGATGACCTTAAAATCGAAAACAATAGAGAATTTTAAAATTTTCGATGACCGAAGTAGAAAATAATGGACAATAC

ACATTTTAAAATTTACGACCTTGAAGTCAGAAATAATGGATAATATCGATTTTAAAATTTTCGATGATCTTGAAGTCGAA

AATAATATACATCGAATTTAAATTTTCGACAATCTTGAAGTGCGAAATAATGAACAATATCGATTTTTAAATTTTTGATG

ATTTTAAATTCGGAAACAACAAAGAATACCAATTTCAAAATTTTCGACGACCTTGAAGCCGGAAAAAATGAACAATACCG

AATTTAAAATTTTTAATAACCTTAAAGTCGGAAAAAATAGAGAATTTTGAAATTTTCAACGACCTTCAAGTCGAAAACAA

TGGACAATACTCATTTTAAAATTTATGACGACTTTGAAGTCGTAAAGAATTGATAATATCAAT

**>CaCIPK24**

ATAAAAATATATAAATATTTGATTAAAATAAATAATGTATAATATAAGAAGATATTGTAAATATCTATGTTAAAATAAAT

ATTATCAAAGTAAAAGAAGGAAGTGTGCTTATTACTTAAAATAAAAAACGAAGTTTGATTTTTAAAATAAAGTTGAGGGT

GAAAAATATTTTCACACCAGACATTTATCACAAAGTAGTACAATAGCTATTAAGTCCCTCTATTTATTACATTTATTTAG

AACTTTCTAGTTTAATCATTTCTGTTAACACCTCATGATCGATGTGATAAGCAATTAATTTGTGTTTGTTAGATCTGCAG

TTGGCAGAACATCGTCTTTTAACTTGTCTTTATTTTTTGTTCCAACTAAAATAAAATGAGTAACGTGGGTAATTTGAACA

CGTCGGTACCCATTGCCCATTCTCGCCTACCTATAATTTTTCTCCTCCAATCCTTTTTTTAACAAAAAAAGTATCAAACC

TTTCCGCGTTGACCCATCAAATTGATACTTTCTTTGTTCATTTTTACATTTTAATGTATTAAAATTTTTGCCAAATCAAG

ATTAACAAAAATCAAACTATAAGAATCTATTATATATCAGCATTATCTAATAATTTTGTCAAAAGTTTATTTACACAACT

TAAATTTATGATCTTTTTATCCATTTCTATTTATCCGGCATATAAAAAATAATTGTATAACTTTATTTATCAAATTTTAG

AAAACATGAGATAATTTATTATTTTATATTTATTTTATTTATGTGGTTAACCACTATTCAGTTCTAATTTTTAAATTATT

TATAATTAACAAAGGCAATATAATAAATAATTCTTTTACTCCCTCCGTTTCATAATAAATAAATTGTTGAATTTTGACAC

ACATATTAAGGAAAAAACATTAAAGACATAAATTTAACATAACTTTTCATTTTTATCTCAAAAAAGAAAGAATTGACCTT

GTAATACCTTTTCAAAAGTTAATTGGTACTCAAATCATAAGGACAAATTTGAAAAAAAATTCAATGATTCACTTATTTTG

AAATATCAATAAATATTCCAATAATTCACTTATTTCGAAACGGAGGTAAGTGAGTATTATTTTTTGAGAAGTATGTCAAA

TTAAATATGAAGCAAGTAATGATTATATAGAATTACAACTCTCCCCCAATTTATGTTACCAATTAAAATATAGAAATTTA

AACTTCTGAATTTTAATAATGAATTCTGACAAAAAAAACTTTAATAGTGACTTTGTAAATAAAAACTTAAAAGTATTTAT

ACCAAAAAAAATGTAGAAATTGAGGGCATCAAAATTCACACTATACGTACCTTCCTGGACCAATCCCCATTTAGAATCTT

CTCTCTTCTTGCGTAATAGCTGCTATTACGATTCTTCTGGAAATCATTCAAATTGAATCTTTATGCTTCTTCATATTTCT

CGTCATACACCAATTTGATGCAAAACGAAGAAAAACATCCCGAGGAATTTGCTACTAGTAATG

**>CaCIPK25**

TTTCTTGAAACAGTGAATTTGGGATTCTGAATCCCATTAGTTTTGAGATCTTTCCTTCTTTTTGAGGCTATCTATCCTTT

TTCTATTTGTTGGTTCTCAGGGTCATTTGTGTTGTTTCTATTTGTTTAATTGGACTTCAAATTTGGCCCCTTTGTGGAGC

TTCTCTCCCTCTCTCTCCCTCTCTCTCTCCCTCCTCTTTTAAATCAAGAAAGTTTATATTTTAATGCTGATTTTGGTTTG

AAGTTTGTTTTGTCTTTTTTTTTTTTTTTTTTCCTGTAGACAATTAGCTTATCCTGTTTGCATTTGTTTGTGGAATAGAT

AGTTCCCGAAGTGATGCTACAAGTTAAGCTATAGTTTAACTAGCTGCATTTTGCTGGGATCTGTTGCTCATTTAGCCGCA

ATAATGTTTAGGTAGTGGATCTCTCTCCCTCGCTCTCTTTCACGGGCACGCACACCGATACAAATGTCTTTTGGTCAGAT

TCAACATTTCTTTGTTTTTAATTCATTCACACCTAGTTTAATCATTTTCTTCAAATGTAGATGCTATTATAATTGGCCTC

TTTAATTAAATGATGATGTGCCTTGATTCTCCCTCCTCCTCCTTAAACAAATAACACAACTAAATAAACTAACCTTTTTT

TTGTTATTCCATTTCTCTTACTATTTAGTGCTACCTCTTTAATTAAATGATGATGTGCCTTGATTCTCCCTCCTCCTTAA

ACAAATAACTCAACTAAATAAACTAACTTTTTTTATGTTATTCCATTTCTCTTACTATTTAGTGCTTCTTTTTTAGATAA

CTGCCAGCTTTCGTGCACCTAGACTAATTCTGTTGAGGCTAGGATAGATGGAAAAATATCACCTAGGCTAAGTTGGAATT

TGAACCTCATCCACTTCATTGACCACTAGGTCACACCCTTGGGTGTGTTAATATTTAGTGTTTCTTGCACTTTGGCTATC

TATTATTTTGTTGTAGTGATTGCTTCTCTTTTAGTATGCTTTGTCATGCTTTCTTAGCATTTTGTCTTGGCATCTTCACT

CCCATTGTTTTCTTTTTTCCTGAACTGCTTTTACTTGAGCCAAGAGTCTATCAGAAACAACCTCTCTTCTCCAAAGGTAG

GAGTAAGGTCTGCGTTCACACTACCCTCCCCAAACTCCACTGTGGGATTACACTGGGTATGCTGCTGTTGTTGGTGGTGG

AGAAAGTGATTATGATGGAAATTTCCATTTGGAATAAAATCCTTCACTCATTCCATTGGCAATTTGCAAGCTGGGGTGTC

TTTTTATGGTGCAGTTTGTCAAGTTGTATTGGAATATCAATTTTATCTTATTTTTGTAATTGGTCTTAGATCTTTGTGGG

TGCCCGGAATGAACGTAAAGACTAGTCTTCTTATGTTAGGAACTCAGATGTCTTACAGAAGTTTTAATCATTGTGCAGTA

AAAAGCTCTTAAGAAGGATTACAACTGCACTTTTTATAGGATTCTGCTAGTATCTCAATGATG

**>CaCIPK26**

GTAGGCAAAAAATATCCAATTAAATCAAAATTCAGGAGGTTTAGGAGTCTGATAGGTACATGCCATAATTGAGGTGTCTA

AGTGAATTTTGACAATATAGAGATGTATTCGTTTTTATTTTATGTGGCACTCTTTTTTATTCATCACATTTTTTTATTCT

CTTTTTTTTTTTACTTGTCACATTTTGGTTTGACACACTTATTAAGAAAATAATTAATAGCATAGTTATTTTATCATAAT

ACCCCTATTAAATGATGTTCGCATTGTGTTTTGAAATTAATTTGGAGAAAATATAATATATTGATGCGAAGGGCTAGAGA

AAAACAAAGTTGTCTTTTCTTGATATGTCAAAATACAATTAGAAAATTAGTGACGAGCAAAAGCAAACGGAGGGAATATT

TGATAACTTTTTAAAAGCACAATTACTCTTTTATCCTTATTGATCTCACTTATAAGGTCTTACTCAATTAAAGATAATAG

TAGTACATTTTTTTTAATATAAGGTAATTTGATAAACATCACTAAATTTTTTCTTATTTCTTAAATTTCATGTACGATCA

AAATATGCCACGTAAAATTGTACTGGGAGAAGATCATAAACTTGAAAATAATAGTATAGTTTGGTTTGTACTTCGTAGCT

GTAAGATGGGAAAGACAGACATATTATGAAATTATTTTAGTTAACCCAGCTTACATAGCGGATTACTTATTCCATCATTT

TAGTAAAAATAATGTAATTAAATAATTGTGAGATTAAATAATATTCATTATTATATACTTTATTTCAGTCGAGTGTTTAT

CGAAAATGATTTTTCTATCTCTACGAAGTAGAGGTCGTAGCGCATGCATATATTCTTGATTCTACCAATGTTATCATAAG

TAATAGGGCAAACCACATAAATTCAGACAGTTTGAAGCTTAATTATAGAAAATTTCTATATTTTGCATAATTATTAAAAA

TCTCAAATTTGAGTCTATCGAAATACATAATTAGCTCCCGTTATATGTTTTTTATTTTATTTTGGGTCTGTTACATCCAA

CGAAGATATAATTTTTTCTAAATAAAAATACATAATTAGCTCCCGATACATTTCGATTTTGTATCTGTCGAGATACGTAA

TTAACTCCCCGATACATCTAATAAAGATACATAATTAATTAAAATTTTTATAATTATTTTATAAAGATGAAAATTTGTGT

AAATATGATAAGTTAAGATGGAGGTTTATATTATTTTTTCATAAAATAATCTCATAAAAGTTATTATCCTTGCCACACCC

TTAAATACTTCTTTAGAGATTCCGATTCCGTAGAAAAAGTCAGCACGTGTTTTCACGTAGTCCAAGTGGCCCAATTAATA

AATAAACTACTTATAAAAGCCAAATATCCATCAAAGAAAACTTCAAACTTCAAACTTCAAAAATCACAAATTCTACCTTC

CCTAAAGACACATACCAAAAAAAATCTTATAAGTTTTTCTTTTATCAAATTTAGTGACAAATG
